# Mitigating tin ion migration and reinforcing buried interface via synergistic polymer-modified tin oxide

**DOI:** 10.1038/s41467-026-77624-8

**Published:** 2026-09-11

**Authors:** Wenxin Yang, Yepin Zhao, Zhen-Yang Suo, Yu-Che Lin, Minhuan Wang, Weixuan Huang, Jian-Guo Zheng, Joo-Hong Lee, Mingjie Xu, Yiushun Tong, Zhichao Shen, Dong Meng, Ran Zheng, Ming Gong, Yingke Zhu, Ying Zhang, Haoxiang Duan, Elizabeth Zhang, Yuran Shi, Chia-Yu Lin, Aman Dhillon, Jinsung Kim, Tso-An Yang, Anatoly I. Frenkel, Xiaoqing Pan, Ilhan Yavuz, Jing Cao, Jin-Wook Lee, Yang Yang

**Affiliations:** 1 Department of Materials Science and Engineering, University of California, Los Angeles, CA USA; 2 State Key Laboratory of Natural Product Chemistry, Key Laboratory of Nonferrous Metal Chemistry and Resources Utilization of Gansu Province, College of Chemistry and Chemical Engineering, Lanzhou University, Lanzhou, China; 3 Key Laboratory of Materials Modification by Laser, Ion, and Electron Beams (Dalian University of Technology), Ministry of Education, School of Physics, Dalian, China; 4 Department of Materials Science and Chemical Engineering, Stony Brook University, Stony Brook, NY USA; 5 Irvine Materials Research Institute, University of California, Irvine, Irvine, CA USA; 6 Department of Integrated Display Engineering, Yonsei University, Seoul, Republic of Korea; 7 Department of Energy Systems Engineering, Seoul National University, Seoul, Republic of Korea; 8 Department of Chemical Engineering, Stanford University, Stanford, CA USA; 9 Department of Physics, Marmara University, Ziverbey, Istanbul, Turkey; 10 School of Transdisciplinary Innovations, Seoul National University, Seoul, Republic of Korea

**Keywords:** Devices for energy harvesting, Solar cells

## Abstract

The buried interface between the charge transport layer and the overlying perovskite absorber is critical yet difficult to probe in perovskite solar cells. Because light enters through the transparent substrate and is predominantly absorbed near this region, photo-induced degradation processes are partially initiated at the hidden buried interface. Here, we reveal that instability at the buried SnO_2_/perovskite interface triggers structural and chemical decomposition of the perovskite layer. To address this issue, we introduce polymeric interlayers that simultaneously reinforce interfacial bonding and suppress tin-ion migration. Poly(1-ethenylpyrrolidine-2,5-dione) (PED) forms a robust and chemically compatible interface, yielding enhanced phase purity, reduced defect density, and significantly mitigated tin diffusion. These findings identify the buried SnO_2_/perovskite interface as a key degradation-sensitive region under light aging and highlight interfacial polymer design as an effective strategy for improving the intrinsic stability of perovskite photovoltaics.

## Abstract (web summary)

The buried interface in perovskite solar cells is vulnerable to light-induced degradation but difficult to probe. Yang et al. reveal a role of tin migration in accelerating degradation and use polymer interlayers to reinforce the interface, improving device performance and stability.

## Introduction

Perovskite solar cells (PSCs) have rapidly emerged as one of the most promising next-generation photovoltaic technologies, achieving power conversion efficiencies (PCEs) that now rival those of crystalline silicon^1,2^. Despite this remarkable progress, their operational stability remains a major obstacle to commercialization^3,4^. Among the multiple interfaces within the device architecture, the buried interface between the charge transport layer (CTL) and the perovskite absorber plays a crucial role yet remains relatively underexplored^5,6^. Unlike the readily accessible top surface, this buried interface is physically enclosed and challenging to probe, resulting in its frequent neglect in both mechanistic studies and materials optimization.

Furthermore, the top and bottom surfaces of a perovskite film exhibit intrinsic asymmetry^7,8^, a factor that has been largely overlooked. Differences in nucleation kinetics, substrate interactions, and illumination exposure produce variations in morphology and defect density between the two surfaces. This structural disparity implies that treating the perovskite layer as a homogeneous bulk material oversimplifies its degradation behavior and limits the effectiveness of conventional stabilization strategies. Understanding and stabilizing this hidden interface is therefore essential for achieving intrinsically durable perovskite photovoltaics.

In the conventional n–i–p device configuration, light enters through the transparent conductive substrate and is predominantly absorbed near the buried ETL/perovskite interface, particularly for high energy photons, owing to the high optical absorption coefficient of metal halide perovskites (Supplementary Fig. 8). Consequently, this region experiences the highest photon flux, carrier density, and thus possible local heating, forming a hotspot where photochemical and electrochemical reactions are likely to initiate^9,10^. Growing evidence indicates that degradation of perovskite films often originates from this buried interface rather than from the exposed top surface, as interfacial defects^11,12^, trapped charges^13,14^, and ion migration^5,15,16^ collectively accelerate structural decomposition and chemical instability. Previous efforts to improve the buried interface have explored self-assembled monolayers^17,18^, fullerene derivatives^19^, two-dimensional perovskite passivation layers^20,21^, and post-treatments such as plasma or molecular doping^22,23^. While these methods have yielded partial improvements in charge extraction and surface passivation, they often suffer from limited chemical robustness, non-uniform coverage, or instability under prolonged illumination and thermal stress. Moreover, few strategies have directly addressed the issue of tin-ion migration from the SnO₂ ETL, a subtle but critical degradation pathway that compromises both the electronic and structural integrity of the perovskite layer.

Here, we introduce a series of polymeric interlayers designed to simultaneously strengthen interfacial bonding, improve perovskite film morphology, and suppress tin-ion diffusion across the SnO₂/perovskite boundary. Among them, PED forms a stable and chemically compatible interface, leading to markedly enhanced efficiency and long-term operational stability. This work identifies the buried interface as a key origin of perovskite degradation and establishes interfacial polymer engineering as an effective strategy for realizing intrinsically stable, high-performance perovskite solar cells.

## Results

### Optimized perovskite quality at buried interface

**Fig. 1:**
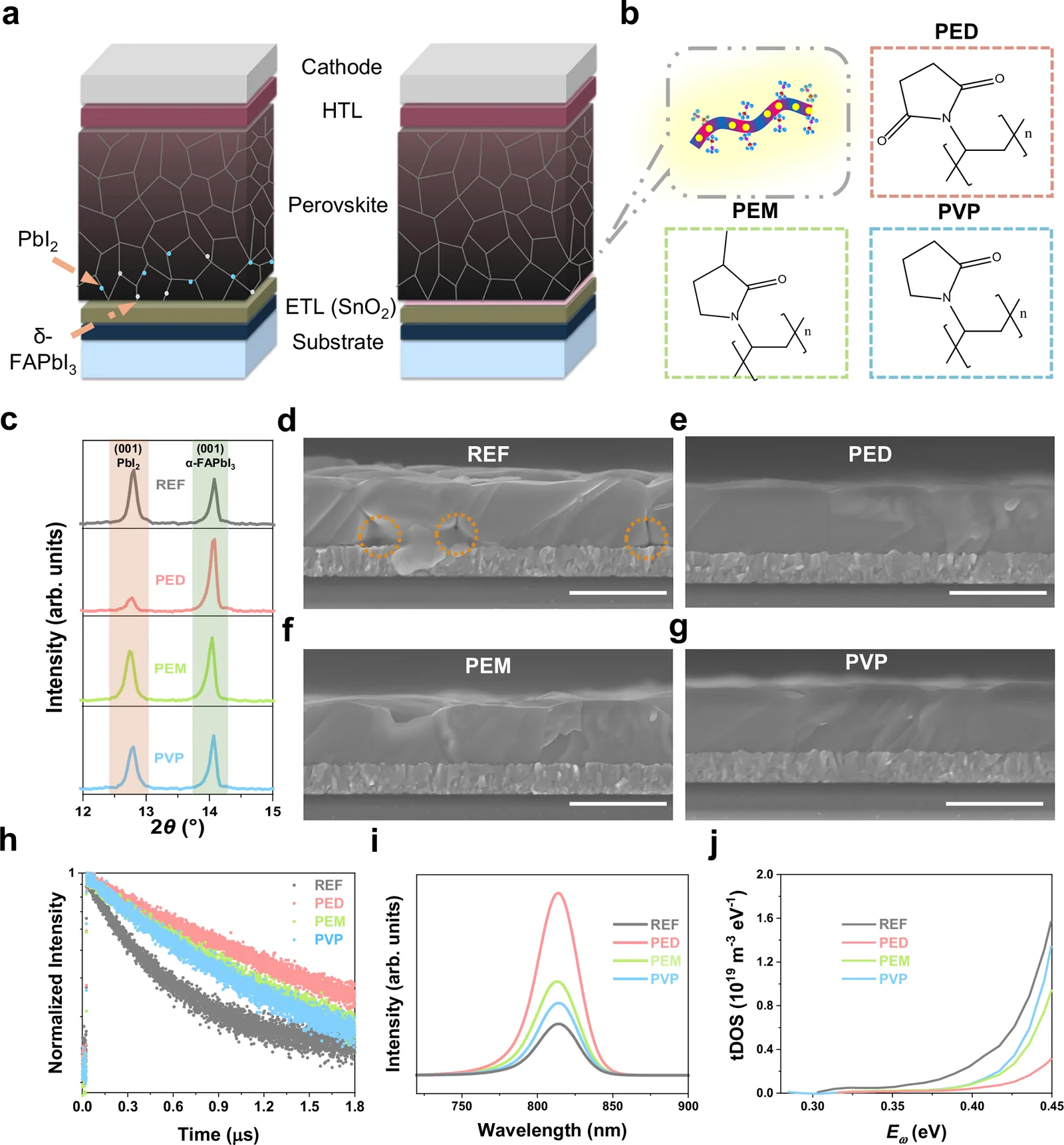
Polymer interlayer design and its effects on perovskite film quality. **a** Schematic device illustration. **b** Polymer structures of PED, PEM and PVP. **c** XRD patterns of buried perovskite interface with and without polymeric interlayers. Cross-sectional SEM (scale bar, 1 μm) images of the fresh perovskite film: **d** without a polymeric interlayer; **e** with PED; **f** with PEM; **g** with PVP. Orange dashed circles highlight interfacial voids. **h** TRPL decay profiles without or with the polymeric interlayers. The testing structure was glass/ITO/SnO_2_/perovskite. **i** Steady-state PL spectra. **j** tDOS in perovskite solar cells with or without polymeric interlayers.

To systematically address the buried interface issue, we rationally selected polymeric interlayers, taking advantage of their uniform film-forming capabilities and tunable side chains without severe aggregation^24,25^. Specifically, we introduced three structurally related polymers, including polyvinylpyrrolidone (PVP), poly(1-ethenyl-3-methylpyrrolidin-2-one) (PEM), and PED as model systems. Building upon PVP, which has been widely utilized in prior literature as a standard Lewis-base passivator^26,27^, we systematically varied the functional groups. PEM introduces steric hindrance via an additional methyl group. Most importantly, PED, our ultimate design, features dual-carbonyl groups specifically chosen to enable strong, bidentate-like chelation. These three polymers were introduced between the SnO₂ ETL and the perovskite absorber, and their effects were compared with control samples without interlayers (Figs. 1a and 1b). To evaluate the influence of these polymers on perovskite crystallinity, the perovskite films were peeled from the substrates using epoxy (Supplementary Fig. 39), and X-ray diffraction (XRD) measurements were performed from the bottom side on as-fabricated samples. The XRD results revealed that the intensity ratio between the (001) α-FAPbI₃ and (001) PbI₂ diffraction peaks increased substantially upon incorporation of the polymeric interlayers. Among them, the PED-modified film exhibited the highest ratio, indicating enhanced perovskite phase purity and crystallinity. The corresponding ratios were 3.18, 1.20, 1.20, and 0.87 for PED, PEM, PVP and REF, respectively (Fig. 1c, Supplementary Fig. 11, Table 1). Cross-sectional scanning electron microscopy (SEM) images of the buried interface (Fig. 1d–g) revealed that the reference perovskite films contained numerous voids and grooves along grain boundaries. In contrast, perovskite films incorporating polymeric interlayers exhibited denser microstructures with significantly reduced grain boundary grooves, as further confirmed by atomic force microscopy (AFM) (Supplementary Fig. 12). To elucidate the root cause of interfacial voids, we investigated the interfacial wetting and surface energy after the polymer treatment. Contact angle measurements (Supplementary Fig. 13) revealed enhanced hydrophilicity on the polymer-modified substrates compared to bare SnO_2_. The increase of the surface energy helps flatten the grain boundary grooves and largely eliminate the voids^28–30^. These morphological defects serve as pathways for oxygen and moisture ingress, promote ion migration, and induce localized electric fields, all of which accelerate phase segregation and non-radiative recombination^31,32^.

The improved interface quality was further reflected in the optoelectronic properties of the perovskite films. Time-resolved photoluminescence (TRPL) decay profiles (Fig. 1h), fitted using a bi-exponential model, revealed prolonged carrier lifetimes of 1.43 μs (PED), 1.10 μs (PEM), and 0.96 μs (PVP), compared to 0.52 μs for the control. Steady-state photoluminescence (PL) spectra (Fig. 1i) showed that polymer-treated samples exhibited markedly higher PL intensity than the reference film. In addition, trap density analysis based on trap density of states (tDOS) measurements (Fig. 1j) demonstrated a reduced density of in-gap states in polymer-modified films, indicating suppressed trap-assisted recombination and improved charge carrier dynamics. These results suggest improved perovskite film quality at the buried interface by introducing the polymeric interlayers.

### Degradation behavior and interfacial stabilization

To verify the difference in degradation rates between the top and bottom regions of the perovskite layer, perovskite films identical to those used in PSC devices were deposited on glass substrates using a two-step deposition process. The films were continuously illuminated under xenon light in ambient air at a relative humidity (RH) of 40% for 24 h without encapsulation. Optical microscopy (OM) images revealed a more pronounced color change at the buried side than at the top surface after aging (Supplementary Fig. 14), suggesting stronger degradation near the buried interface. This observation is further supported by depth-profile XPS of fresh and aged devices. Compared with the fresh device, the aged device shows pronounced Pb/I/N compositional decoupling near the buried SnO₂/perovskite interface, where the N-containing organic component is depleted relative to Pb/I-containing inorganic species in part of the buried-interface region (Supplementary Figs. 9 and 10).

**Fig. 2:**
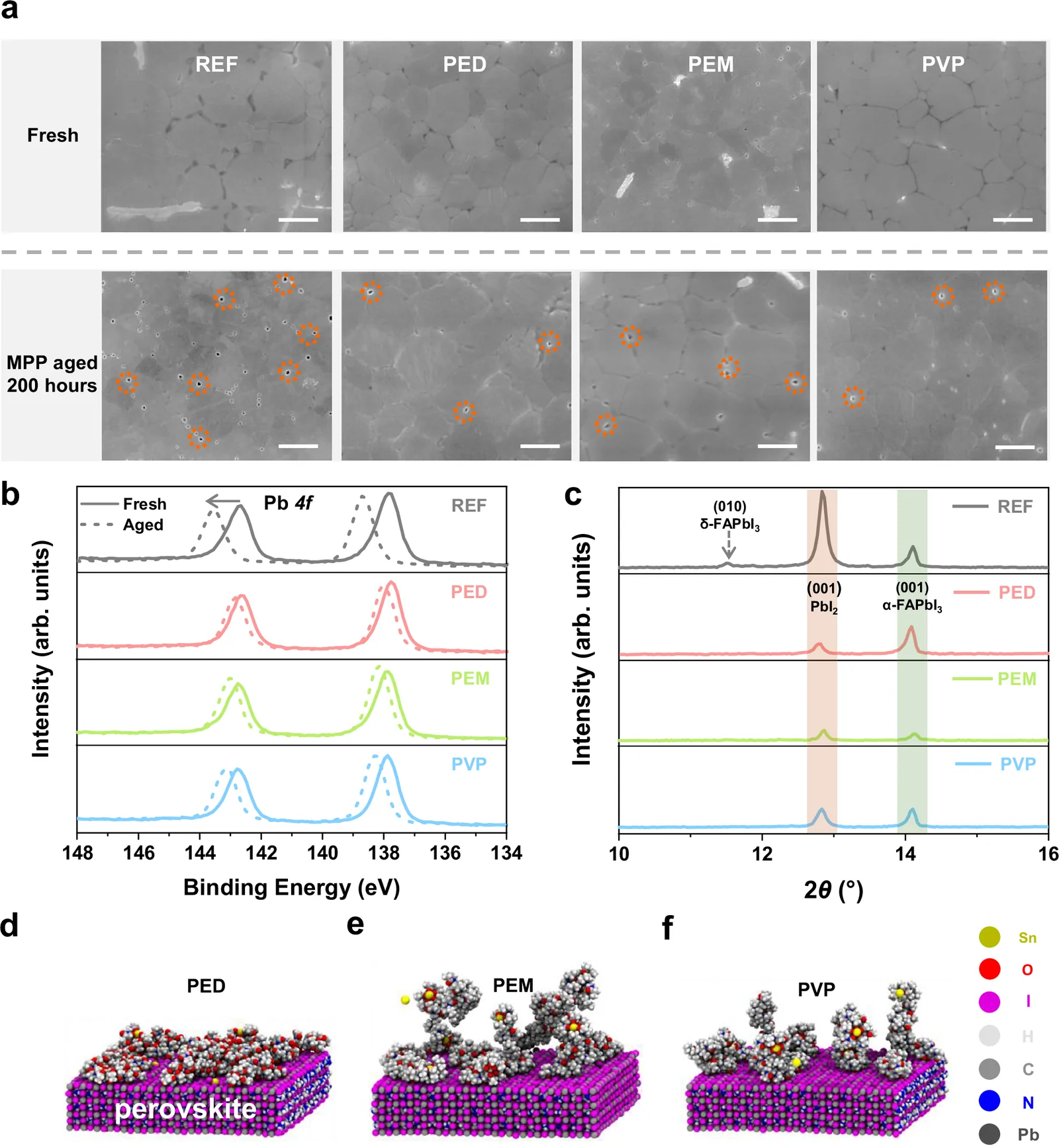
Polymer interlayers mitigate buried interface degradation. **a** SEM images (scale bar, 1 μm) of the buried perovskite surface with and without polymeric interlayers. Orange dashed circles highlight voids formed after aging. **b** Pb *4 f* XPS spectra with and without polymeric interlayers before and after operation. The arrow indicates the binding-energy shift after aging. **c** XRD patterns of buried perovskite surface after operation. MD simulation structures of PED (**d**); PEM (**e**); PVP (**f**) on the perovskite surface.

To further evaluate the effect of polymeric interlayers on the stability of this buried interface, we conducted a series of characterizations on aged films extracted from devices with the structure: substrate /ITO/ETL (SnO_2_)/ (polymeric interlayer)/perovskite/HTL/Au. The perovskite layer exhibited a bandgap of 1.56 eV (Supplementary Fig. 15). The perovskite layers were peeled off after operation under maximum power point tracking (MPPT) for 200 hours. SEM analysis (Fig. 2a) showed that the aged perovskite bottom surfaces of reference devices developed pronounced grain boundary grooves and voids, consistent with structural degradation. In contrast, films incorporating polymeric interlayers exhibited smoother, denser morphologies.

X-ray photoelectron spectroscopy (XPS) provided additional insights into the chemical stability of the buried interfaces (Fig. 2b). In fresh samples, the Pb *4 f* core-level spectra confirmed the expected oxidation state of Pb²⁺. After aging, however, the reference sample displayed peak broadening and an evident positive binding energy shift (0.81 eV), signifying oxidation and the formation of lead-containing degradation species. The incorporation of polymeric interlayers did not significantly affect the chemical state of fresh samples. After aging, the PED-modified sample showed a much smaller Pb *4f* binding-energy shift (0.15 eV) and largely preserved line shapes, indicating reduced oxidative degradation. Although PVP- and PEM-treated samples also exhibited improved interfacial stability compared to the control, PED provided the most effective suppression of chemical deterioration.

To further examine phase evolution upon aging, perovskite films were subjected to simultaneous light and thermal stress under one-sun illumination at 65 °C. As shown in Fig. 2c, XRD patterns of the reference film revealed an increased PbI_2_ peak intensity and the emergence of the δ-FAPbI₃ phase, both indicative of decomposition and phase transformation. In contrast, the PED-treated film retained a stable α-FAPbI_3_ diffraction pattern with negligible PbI_2_ formation and no detectable δ-phase, indicating improved phase stability. While PVP and PEM interlayers also mitigated phase degradation to some extent, PED proved the most effective in maintaining crystallinity and suppressing structural transition (Supplementary Fig. 16, Table 2). This increased α-FAPbI_3_/PbI_2_ ratio also suggests that the strong Lewis acid-base coordination between the polymer’s C=O groups and Pb^2+^ ions stabilizes intermediate phases during annealing. This kinetic modulation prevents premature PbI_2_ precipitation, thereby ensuring a more complete conversion to the pure α-FAPbI_3_ phase.

To elucidate the distinct interfacial behaviors of the three polymers, molecular dynamics (MD) simulations were performed to investigate their interactions with the perovskite surface. As illustrated in Fig. 2d–f and Supplementary Fig. 17 and 18, PED molecules exhibited strong adhesion and close packing at the perovskite interface, forming a compact and robust interfacial configuration. In contrast, a significant fraction of PEM and PVP molecules detached from the surface, suggesting weaker interfacial interactions and a higher tendency for nonuniform coverage. In practical device structures, such weak adhesion may promote polymer aggregation and result in incomplete interlayer formation, thereby reducing passivation effectiveness and allowing morphological defects to persist^33^.

### Mitigation of tin ion migration

**Fig. 3:**
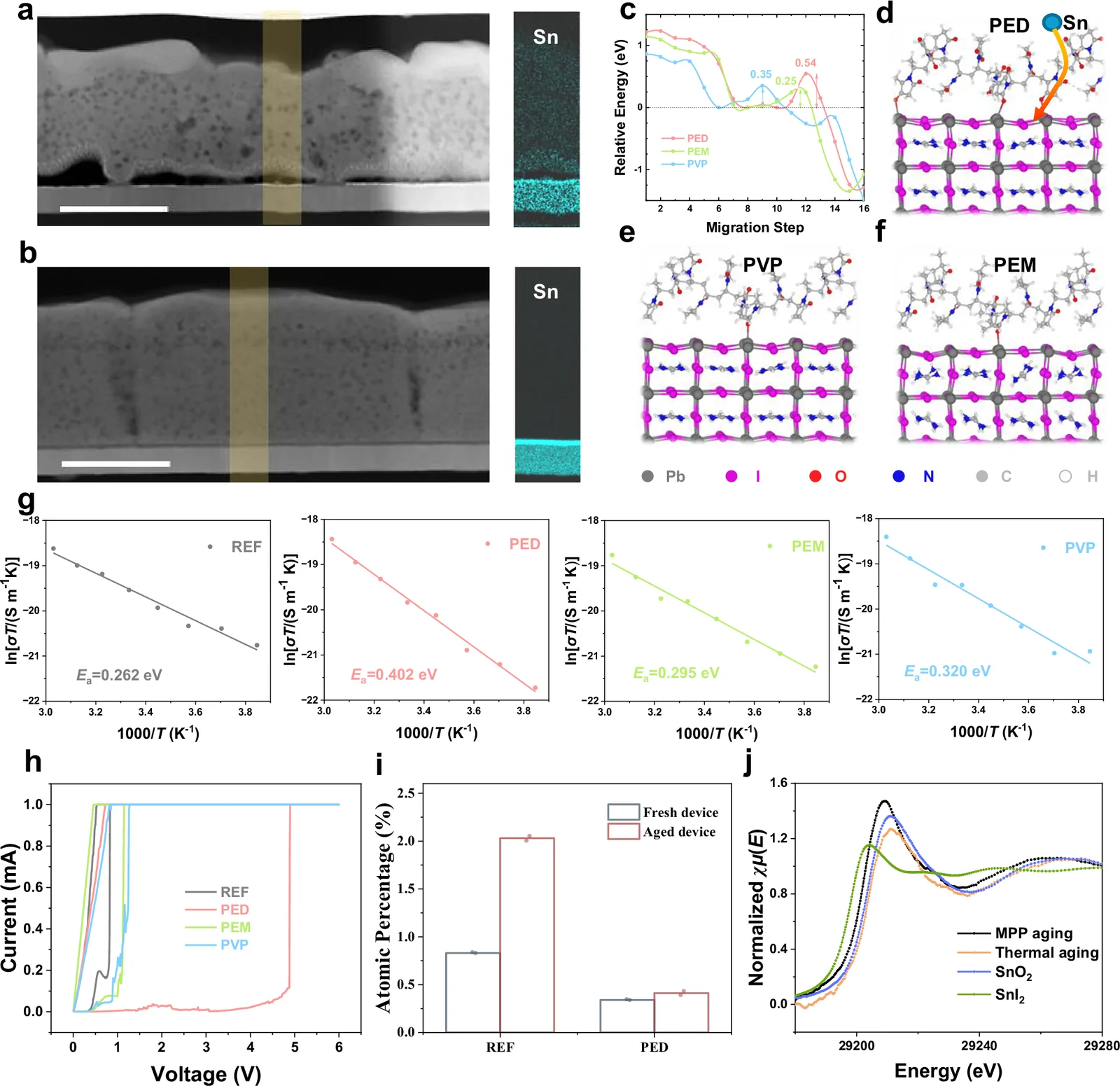
Suppression of tin migration by polymer interlayers. **a** Cross-sectional STEM image (scale bar, 500 nm) and Sn EDS elemental map of the highlighted area in the reference sample after 1000 hours of MPPT aging. The yellow shaded regions indicate the areas corresponding to the Sn EDS analysis. **b** Cross-sectional STEM image and Sn EDS elemental map of the highlighted area in the PED-treated sample after MPPT aging for 1000 hours. The yellow shaded regions indicate the areas corresponding to the Sn EDS analysis. **c** Calculated Sn migration energy profiles for the PED, PEM, and PVP interlayers. Corresponding atomic models of the interfaces with PED (**d**), PVP (**e**), and PEM (**f**) interlayers. In (**d**), the orange arrow indicates the simulated Sn migration pathway. **g** Temperature-dependent conductivity of devices without and with PED, PEM, and PVP polymeric interlayers. *E*_a_ is the activation energy. Symbols represent measured data and lines represent linear fits. **h** Resistive switching measurements of perovskite solar cells with or without polymeric interlayers. **i** ICP-AES measurements of Sn content in aged and fresh samples. Bars represent the mean of two independent results (*n* = 2), with individual data points shown. **j** Sn K-edge X-ray absorption near-edge spectra showing an intermediate oxidation state (between those of SnI_2_ and SnO_2_) for the aged sample.

Cross-sectional scanning transmission electron microscopy (STEM) images of reference devices operated under MPPT for 1000 hours revealed clear evidence of perovskite degradation initiating from the buried interface (Fig. 3a, Supplementary Fig. 19)^34,35^. Energy-dispersive X-ray spectroscopy (EDS) elemental maps confirmed that tin ions originating from the SnO_2_ ETL infiltrated into the lower region of the perovskite film, triggering early-stage interfacial decomposition. These observations demonstrate that tin migration from the SnO₂ ETL into the perovskite active layer constitutes a significant yet often overlooked degradation pathway, posing a critical threat to device efficiency and operational lifetime. In contrast, devices incorporating a PED interlayer between the SnO_2_ and perovskite layers (Fig. 3b, Supplementary Fig. 20) exhibited minimal interfacial degradation and no discernible Sn penetration into the absorber, highlighting the effectiveness of the polymeric barrier in mitigating ion diffusion.

To elucidate the underlying mechanisms of Sn-ion suppression, density functional theory (DFT) calculations were performed to examine the interaction between tin ions and the three polymeric interlayers. All polymers introduced additional energy barriers that impeded tin migration (Fig. 3c–3f). Among them, PED produced the highest migration barrier of 0.54 eV, followed by PVP (0.35 eV) and PEM (0.25 eV). Further investigations for overall ion migrations, including halide and oxygen ion migration, were conducted through temperature-dependent conductivity measurements on devices with the structure of FTO/SnO_2_/perovskite/Au (Supplementary Fig. 21). The activation energy (*E*_a_) values for ion migration were extracted from linear fits to the Nernst-Einstein relation^36^:

1 $$\sigma \left(T\right)=\frac{{\sigma }_{0}}{T}\exp \left(\frac{-{E}_{{{{\rm{a}}}}}}{{k}_{{{{\rm{B}}}}}T}\right)$$

where *σ*(*T*) is the temperature-dependent conductivity, *σ*₀ is a pre-exponential constant, and *k*_B_ is the Boltzmann constant. The extracted activation energies for PED-, PEM-, and PVP-modified films were 0.402 eV, 0.295 eV, and 0.320 eV, respectively, significantly higher than that of the reference film (0.262 eV) (Fig. 3g). The experimental trend in *E*ₐ values was consistent with the DFT results for Sn-ion in Fig. 3c, confirming the enhanced energy barrier to effectively suppress ion migration by PED.

Resistive switching measurements were further conducted to assess ion mobility under an applied electric field (Fig. 3h, Supplementary Figs. 22–24). The transition from high resistance state (HRS) to low resistance state (LRS) is mainly determined by conductive filament formation via mobile ion migration (Supplementary Fig. 22). Under the applied electric field, the reference device exhibited an abrupt increase in current (set transitions) below 1 V, indicative of rapid conductive filament formation between top and bottom electrodes driven by mobile ions. In contrast, devices with a polymer interlayer, particularly those incorporating PED, showed a markedly enhanced threshold voltage of ~5 V for the transition from HRS to LRS (Fig. 3h and Supplementary Fig. 23). Notably, the device with only PED without SnO_2_ demonstrated relatively lower threshold voltage compared to that for REF device (Supplementary Fig. 24). These results imply that the synergistic effects between SnO_2_ and PED can effectively suppress the mobile ion migrations in PED-treated devices. Collectively, both computational and experimental analyses confirm that polymeric interlayers, especially PED, play a vital role in retarding migration of mobile ions, including tin ions, and stabilizing the buried interface.

Tin ion migration becomes more pronounced under external stresses such as prolonged illumination and elevated temperature. To quantify tin diffusion and evaluate the barrier efficacy of PED, inductively coupled plasma atomic emission spectrometry (ICP-AES) was performed on perovskite films that were dissolved from the substrate after MPPT operation (Fig. 3i, Supplementary Table 3). After 100 hours of MPPT operation, the tin content in the reference device increased from 0.83 at% to 2.03 at%, whereas the PED-modified device showed only a minimal increase from 0.34 at% to 0.41 at%. XPS corroborated these findings, revealing a detectable Sn signal (0.25 at%) on the bottom surface of aged reference devices but no measurable Sn in polymer-interlayer-treated samples (Supplementary Figs. 25, 26, Table 4). These results confirm the strong barrier capability of PED against tin diffusion, consistent with STEM/EDS observations.

To further probe the chemical state of migrated tin species, X-ray absorption near-edge structure (XANES) spectroscopy was performed on aged devices (Fig. 3j, Supplementary Fig. 27). The Sn K-edge spectra revealed that the oxidation state of diffused tin lies between that of SnI_2_ and SnO_2_, indicating partial oxidation within the perovskite matrix. This intermediate oxidation state suggests that tin migration introduces chemical perturbations that alter the local electronic structure and promote defect formation, unintentional doping, and interfacial recombination^37,38^. The inability of migrated tin to revert to its original oxidation state implies irreversible modification of the perovskite environment, with long-term consequences for both device stability and efficiency. These findings underscore the importance of suppressing not only tin migration but also its subsequent oxidation, providing a mechanistic basis for the improved durability achieved through PED-based interfacial engineering.

### Enhanced photovoltaic performance and long-term operational stability

**Fig. 4:**
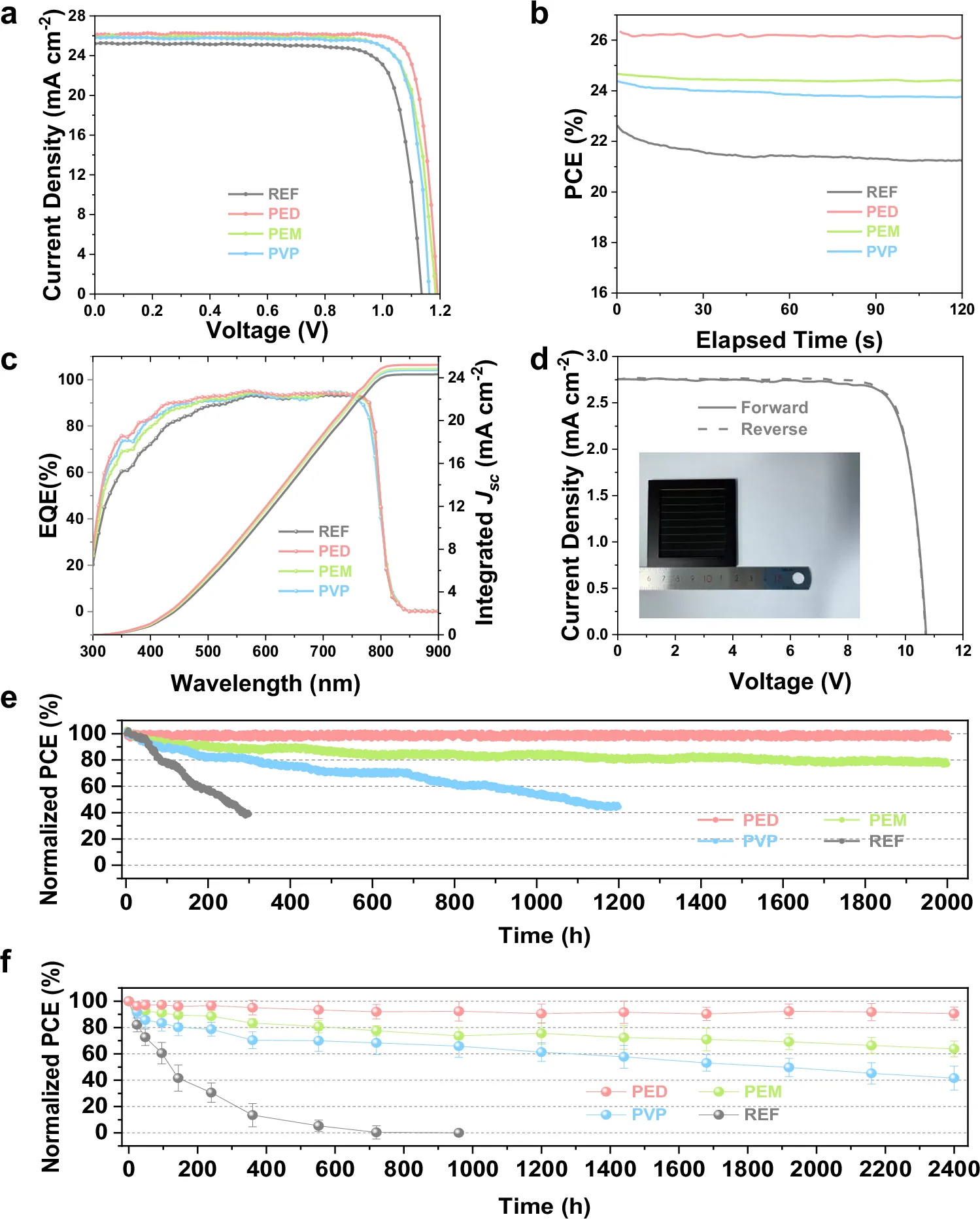
Photovoltaic performance and stability of polymer-modified perovskite solar cells and modules. **a** *J*–*V* curves of devices with and without polymeric interlayers. **b** Stabilized power output measured under maximum power point tracking. **c**, EQE spectra and integrated *J*_sc_ values of the best-performing devices. **d** Forward and reverse *J*–*V* curves of module with a PED interlayer. **e** MPPT stability of PTAA-based devices under one-sun illumination at 65 °C with and without polymer interlayers. The initial PCEs of the reference, PED, PEM, and PVP devices were 21.88%, 24.89%, 24.08%, and 23.58%, respectively. **f** Thermal-light stability of PTAA-based devices under one-sun illumination at 85 °C with and without polymer interlayers. The initial PCEs of the reference, PED, PEM, and PVP devices were 22.57%, 25.28%, 24.31%, and 23.88%, respectively. Data points indicate the median values, and error bars represent the standard deviation across ten independent devices (*n* = 10).

The photovoltaic performance of devices incorporating different polymeric interlayers was systematically evaluated. The introduction of a PED interlayer resulted in a pronounced enhancement in average PCE, increasing from 23.43% for the reference device to 26.74%. Devices incorporating PEM and PVP interlayers also exhibited improved performance, with average PCEs of 25.23% and 25.12%, respectively. The addition of polymeric interlayers led to simultaneous increases in open-circuit voltage (*V*_oc_), short-circuit current density (*J*_sc_), and fill factor (FF), reflecting enhanced charge extraction and reduced interfacial recombination (Fig. 4a and Supplementary Fig. 31, Table 5). The PED-modified device achieved a certified PCE of 26.58%, with a *V*_oc_ of 1.194 V, *J*_sc_ of 25.87 mA cm^−2^, and FF of 86.06% (Supplementary Fig. 28). The stabilized PCE values also improved markedly, from 21.3% in the reference device to 26.2%, 24.4%, and 23.8% for PED-, PEM-, and PVP-modified devices, respectively (Fig. 4b). External quantum efficiency (EQE) spectra (Fig. 4c) confirmed these results, showing excellent agreement between the integrated *J*_sc_ values and those obtained from *J*–*V* measurements, with less than 5% deviation for the PED-treated device (integrated *J*_sc_ = 25.2 mA cm^−2^). While the EQE spectra displayed negligible differences above 500 nm, the PED-modified device exhibited a distinctly higher EQE in the 350-400 nm region, indicating improved photocarrier generation and extraction near the buried interface. As short-wavelength photons possess a large absorption coefficient and a shallow penetration depth in perovskites (Supplementary Fig. 8)^39^, they are predominantly absorbed adjacent to the bottom heterojunction. Therefore, this localized EQE enhancement supports improved suppression of interfacial non-radiative recombination and more efficient charge collection^40^. These results demonstrate that polymeric interlayers effectively enhance interfacial charge transport and overall photovoltaic performance, with PED yielding the most significant improvement. Building on the improvements observed in small-area devices, we further examined the scalability of the polymer-interlayer strategy by fabricating large-area perovskite modules with an active area of 21.54 cm². The PED-interlayer-based module achieved a certified power conversion efficiency of 24.17% (Fig. 4d, Supplementary Fig. 29), demonstrating that the interfacial advantages introduced by the PED interlayer are well preserved during scale-up. These results underscore the effectiveness of polymeric interlayers in enabling high-efficiency, large-area perovskite modules.

To gain further insight into the origin of the improved device efficiency, the interfacial energy level alignment was examined using ultraviolet photoelectron spectroscopy (UPS) and Kelvin probe force microscopy (KPFM). Since the polymer layers function as interfacial modifiers rather than standalone charge-transport layers, UPS measurements were performed on SnO₂/polymer stacks to probe the device-relevant surface energetics at the buried interface. Polymer-only UPS spectra were also measured as references and are provided in Supplementary Fig. 35. As shown in Supplementary Fig. 34 and Supplementary Table 8, the PED-modified SnO₂ exhibited a shallower work function compared with pristine SnO₂, while the valence-band onset remained nearly unchanged. These results indicate that PED modifies the surface electronic structure of SnO₂ without substantially changing its occupied-state onset. Such interfacial energy modulation is expected to improve energetic compatibility at the SnO₂/perovskite interface, contributing to more efficient electron extraction and reduced interfacial recombination. Crucially, this tailored energy level alignment provides a clear physical link between the material energetics and the observed device performance. The minimized energy offset, likely driven by the interfacial dipoles of the polar polymer chains, is expected to increase the built-in potential and effectively suppress non-radiative carrier recombination at the buried interface. Together, this optimized interfacial energy alignment likely contributes to the substantial enhancement in *V*_oc_ observed in the *J*-*V* curves (Fig. 4a). The KPFM mapping also revealed a more homogeneous surface potential distribution for the PED-treated film, implying reduced surface trap density and a more uniform interface.

The operational stability of the encapsulated solar cell devices was further examined under MPPT using continuous 1-sun-equivalent white-light LED illumination at 65 °C in the N_2_-filled glovebox (Fig. 4e). The PED-treated device exhibited long-term stability, maintaining 96 % of its initial PCE after 2000 hours of continuous operation, whereas the reference device degraded completely. Devices with PEM and PVP interlayers also showed improved retention of performance over time. To assess thermal-light durability, unencapsulated devices were further tested under accelerated ISOS-L-2I conditions at 85 °C and 1-sun-equivalent illumination in a nitrogen-filled glovebox (Fig. 4f). After 2400 hours, the reference device exhibited complete degradation, while devices containing PED, PEM, and PVP interlayers retained 90.5 %, 63.7 %, and 41.5 % of their initial PCEs, respectively. Furthermore, the perovskite film incorporating the PED interlayer exhibited significantly enhanced stability under 40% relative humidity and continuous light illumination compared to the reference sample (Supplementary Fig. 38). Collectively, these results underscore the critical role of polymeric interlayers in stabilizing the buried interface and suppressing ion migration, thereby enabling substantial improvements in both efficiency and long-term operational durability of perovskite solar cells.

## Discussion

This work identifies the buried interface between the SnO₂ electron transport layer and the perovskite absorber as a key degradation-sensitive region in perovskite solar cells. Owing to its high photon flux and carrier density, this hidden interface is particularly vulnerable to light-induced decomposition and tin-ion migration. By introducing polymeric interlayers, we strengthened interfacial bonding, improved film morphology, and effectively suppressed tin diffusion. Among the investigated polymers, PED exhibited the strongest adhesion and chemical stability, leading to enhanced crystallinity, higher activation barriers for ion migration, and suppressed phase transformation under stress. Consequently, PED-modified devices achieved a power conversion efficiency of 26.58% and retained 96% of their initial performance after 2000 hours of operation. These findings highlight interfacial polymer engineering as an effective approach to achieving intrinsically stable and high-efficiency perovskite photovoltaics.

## Methods

### Materials and small-area device fabrication

Dimethylsulfoxide (DMSO, 99.7%), isopropanol (IPA, 99.7%), dimethylformamide (DMF, 99.8%), acetone (99.9%), chlorobenzene (99.8%), acetonitrile (99.8%), octylammonium iodide (OAI), 4-*tert*-butyl pyridine (98%), lithium bis(trifluoromethanesulfonyl)imide (99.95%), 2,2′-Azobis(2-methylpropionitrile) (AIBN, 98%) and PVP10 (average Mw~10000) were all purchased from Sigma-Aldrich. Lead iodide beads (PbI_2_, 99.999%) and SnO_2_ colloidal solution (15% in water) were purchased from Alfa Aesar. Formamidine iodide (FAI), methylammonium bromide (MABr), and methylammonium iodide (MAI) were purchased from GreatCell Solar. Spiro-MeOTAD (99.8%) and methylammonium chloride (MACl) were purchased from Xi’an Polymer Light Technology. 1-ethenylpyrrolidine-2,5-dione and 1-ethenyl-3-methylpyrrolidin-2-one were purchased from Enamine. All the materials were used without further purification.

ITO glass slides were sequentially cleaned with detergent, deionized water, acetone, and IPA for 30 minutes each. Then, the ITO substrates were dried with a nitrogen gun and treated by UV-ozone for 20 minutes to remove organic residue from the surface. Some 0.2 ml of SnO_2_ colloidal solution was diluted in 1.2 ml of deionized water and dropped on the pre-cleaned ITO glass slides at a rate of 3,000 r.p.m. for 30 s, followed by annealing at 150 °C for 30 minutes in air. After cooling to room temperature, the substrates were treated by UV-ozone for another 10 minutes and transferred into the glove box for the following steps. For the polymer interlayers, PED was dissolved in chloroform with a concentration of 1 mg/ml. PEM and PVP were dissolved in chlorobenzene with a concentration of 1 mg/ml. The PED concentration was optimized through concentration-dependent device screening from 0.1 to 10 mg mL⁻¹, with 1 mg mL⁻¹ providing the best representative photovoltaic performance and the most balanced improvement in *V*_oc_, *J*_sc_, and FF (Supplementary Fig. 33 and Supplementary Table 7). PED was used as the representative polymer for concentration-dependent optimization because it showed the best overall device performance and buried-interface stabilization. After identifying 1 mg mL⁻¹ as the optimized PED concentration, the same concentration was applied to PEM and PVP to ensure a fair comparison under identical processing conditions. The solutions were spin-coated at a speed of 3000 r.p.m. for 30 s and annealed at 75 °C for 1 minute. This spin-coating condition was selected based on spin-speed-dependent device-performance screening using PED as the representative polymer interlayer (Supplementary Fig. 42). Additional attenuated total reflectance Fourier-transform infrared spectroscopy (ATR-FTIR) and proton nuclear magnetic resonance spectroscopy (¹H NMR) characterizations indicate that the polymer interlayers remain largely stable under the 150 °C perovskite annealing condition, with no obvious evidence of thermal decomposition or further polymerization (Supplementary Figs. 2 and 6). For the fabrication of perovskite films, a typical two-step method was employed: 1.5 M PbI_2_ in DMF and DMSO (volume ratio, 9.5:0.5) was spin-coated onto the SnO_2_ films at a rate of 1500 r.p.m. for 30 s and annealed at 80 °C for 30 seconds. Organic salts of FAI/MACl/MABr (70 mg:10 mg:4 mg in 1 ml IPA) were spin-coated on PbI_2_ films at a rate of 1800 r.p.m. for 30 s, followed by thermal annealing at 150 °C for 13 min in ambient air with a controlled humidity (30–40%). The OAI solution (3 mg OAI was dissolved in 1 mL IPA) as a passivation procedure was spin-coated on perovskite films at 4000 rpm for 30 seconds. After being cooled to room temperature, the perovskite films were transferred into a glove box for spiro-MeOTAD deposition. The spiro-MeOTAD solution consisted of 72.3 mg spiro-MeOTAD, 28.8 μl of 4-*tert*-butyl pyridine and 17.5 μl of lithium bis(trifluoromethanesulfonyl) imide solution (520 mg lithium bis(trifluoromethanesulfonyl) imide in 1 ml acetonitrile) in 1 ml of chlorobenzene. Some 40 μl of the spiro-OMeTAD solution was spin-coated on perovskite films at a rate of 3000 r.p.m. for 30 s. For the stability test devices, we employed poly(bis(4-phenyl)(2,4,6-trimethylphenyl)amine) (PTAA) doped with 4-isopropyl-4′-methyldiphenyliodonium tetrakis (pentafluorophenyl) borate as the hole-transporting layer. Finally, 80 nm of Ag or Au was thermally evaporated as the electrode under high vacuum (<5.0 × 10^−6^ Pa).

### Module Fabrication

Perovskite solar modules, consisting of nine sub-cells connected in series, were fabricated on FTO glass substrates with dimensions of 6 × 6 cm^2^. The series interconnection of the module was achieved by patterning P1, P2, and P3 lines using a laser scribing system with a wavelength of 1064 nm and a power output of 20 W. The FTO substrate was pre-patterned for P1 (20 μm width) utilizing 40% laser power at a speed of 500 mm/s, a frequency of 200 kHz, and a pulse width of 50 ns. The subsequent processes were identical to those used for small-area devices.

The P2 lines (80 μm width) were etched prior to the Au evaporation step, using an average laser power of 33%, a speed of 500 mm/s, a frequency of 50 kHz, and a pulse duration of 50 ns. The distance between P1 and P2 was approximately 60 μm. Following the deposition of a 100 nm thick gold layer, the P3 line (10 μm width) was fabricated under the same scribing conditions as the P2 line. The distance between P1 and P3 was approximately 180 μm, and the geometric fill factor (GFF) was around 96.5%. Additionally, laser edge cleaning was applied to the perovskite components, resulting in a light-receiving area of 21.54 cm² for the perovskite component. The certified test was conducted using a Mask with an aperture area of 21.5 cm^2^.

The module geometric fill factor (GFF) was determined from the designed and experimentally verified interconnection geometry. The sub-cell width was approximately 5200 μm, while the interconnection dead zone defined by the P1/P2/P3 scribing region was approximately 180 μm, corresponding to a GFF of approximately 96.5%. The scribing quality and interconnection dimensions were further verified by SEM/optical microscopy images (Supplementary Fig. 30).

### Material characterization

Resistive switching tests were measured using voltage sweep mode (CC = 1 mA) on a Keithley 4200 SCS under vacuum around 10^−2^ torr.

XPS measurements were carried out on an XPS AXIS Ultra DLD (Kratos Analytical). An Al Kα (1,486.6 eV) X-ray was used as the excitation source. UPS measurements were carried out using a helium discharge lamp, emitting ultraviolet energy at 21.2 eV. All UPS measurements were performed using standard procedures with a −9 V bias applied between the samples and the detectors.

PL spectroscopy was performed with a Horiba Jobin Yvon system, with a 532-nm xenon lamp or a 640-nm monochromatic laser excitation source. Time-resolved PL decay profiles were measured using a Picoharp 300 with time-correlated single-photon counting capabilities (PLD 800B, PicoQuant) with a repetition frequency of 100 kHz.

X-ray absorption spectroscopy measurements were performed at the QAS (7-BM) beamline of the National Synchrotron Light Source – II at Brookhaven National Laboratory. The measurements were performed in fluorescence mode, and up to 50 scans for each sample were merged to improve the signal-to-noise ratio.

SEM (Nova Nano 230) was used to characterize the morphology of the perovskite films. X-ray diffraction measurements were carried out by an X-ray diffractometer (PANalytical) with Cu Kα radiation at a scan rate of 4° min^−1^.

*J*–*V* characterizations of the solar cells were carried out with a Keithley 2401 source meter, under simulated one-sun illumination (AM 1.5 G, 100 mW cm^−2^; Oriel Sol3A with class AAA solar simulator, Newport). The intensity calibration of the light was done by a National Renewable Energy Laboratory (NREL)-certified Si photodiode with a KG-5 filter. For each condition, at least eight devices were fabricated and measured for statistical analysis. *J*–*V* characterizations were conducted from −0.1 V to 1.22 V at a scan rate of 0.1 V s^−1^ using a mask with an area of 0.0735 cm^2^. The device area is defined by the overlap patterned bottom ITO electrode and top metal electrode. The measurement of solar cells was carried out in an ambient atmosphere without pre-conditioning such as voltage bias and light soaking. The steady-state power output of the solar cells was calculated from the photocurrent, measured under constant bias voltage operation, that corresponds to a maximum power output. The IPCE measurement was carried out using a specially designed system (Enli Tech) in a.c. mode (chopping frequency, 210 Hz) without bias light.

Cross-sectional TEM samples were prepared using Tescan Gaia3 Dual Beam FIB. STEM and EDS experiments were carried out by using a JEOL JEM-2800 S/TEM operated at 200 kV with dual 100 mm^2^ silicon drift detector X-ray detectors.

AFM/KPFM was performed on a Dimension Icon Scanning Probe Microscope (Bruker Nano, Inc.).

ICP-AES was performed on a Shimadzu ICPE-9000. ICP-AES tuning solution and hydrochloric acid were from Alfa Aesar and Fisher Chemical, respectively. Standard element solution was purchased from the National Center of Analysis and Testing for Nonferrous and Electronic Materials. The standard solution was diluted to 1000.0, 500.0, 200.0, 100.0, and 20.0 ppb with 1% HCl for the calibration curve. Milli-Q water (18.2 MΩ) was used in all experiments. Collected perovskite powder was scraped from approximately 30 individual, fully fabricated devices (yielding about 0.2–0.5 mg per device to accumulate enough amount of powder for testing conditions).

FTIR spectra were collected in ATR mode using a Thermo Scientific Nicolet Summit X FTIR spectrometer equipped with an Everest ATR accessory and a ZnSe ATR crystal. The polymer-coated side was placed in contact with the ATR crystal during measurement. The spectra were plotted as normalized absorbance to compare peak positions and characteristic vibrational features. For thermal-stability characterization, polymer powders were heated at 150 °C for 13 min, matching the perovskite annealing condition used in device fabrication.

Size exclusion chromatography (SEC) analysis was performed using a Tosoh EcoSEC Ambient (Room Temperature)-GPC equipped with two TSKgel SuperAW GPC columns connected in series (two SuperAWM-H; 6.0 mm I.D. x 15 cm) calibrated with a conventional calibration curve using Sigma-Aldrich monodisperse polystyrene standards ranging from 1.0 MDa to 500 Da. Dimethylformamide (DMF) with 0.1 wt% lithium bromide (40 °C) was used as a carrier solvent at a flow rate of 0.6 mL/min. Samples were prepared at a concentration of around 1 mg/mL in DMF with 0.1% LiBr, with a sample injection volume of 50 μL. An instrument equipped RI detector was used as the main source of peak detection, and peak analysis was conducted using EcoSEC Elite GPC System Workstation Software.

### Molecular dynamics

All Molecular Dynamics (MD) simulations were carried out using the GPU-accelerated version of AMBER24^41^. For molecular mechanics parameters, the General AMBER Force Field (GAFF) was used for PED, PEM, and PVP and FA^+^, following a standard protocol described in previous studies^42^. For the remaining ions, force-field parameters designated within AMBER24 code were selected. A large 10 nm x 10 nm FAPbI_3_ perovskite slab, and a polymer layer on top with or without free Sn ions, were first generated. Initial geometries of the polymers (having few monomer units) were prepared by randomly placing 12 polymers on the surface using the PACKMOL code^43^, ensuring a minimum intermolecular distance of 2 Å. Partial charges were generated via the Merz-Singh-Kollman scheme using HF/6-31 G(d) as implemented in Gaussian09^44^ and incorporated as restrained electrostatic potential (RESP) charges^45,46^. Periodic boundary conditions were imposed. The whole structure was initially energy-minimized over 1000 MD steps, with positional restraints applied to all heavy atoms. Each system was then heated and equilibrated at 300 K for 4 ns, during which the temperature was controlled using a Langevin thermostat (collision frequency of 5.0 ps^−1^). This was followed by an additional 2 ns of relaxation using a Berendsen barostat to equilibrate the system at a pressure of 1 atm. Lastly, all restraints were removed, and a production run of 40 ns was performed, maintaining the system at a constant temperature of 300 K and a time-averaged pressure of 1 atm.

### Density functional theory calculations

We calculated the energy barrier of tin migration through polymer materials on the perovskite surface with DFT. All simulations were performed in VASP (Vienna Ab initio Simulation Package) with a plane-wave basis and the projector-augmented wave (PAW) method^47,48^. We used the Perdew–Burke–Ernzerhof (PBE) generalized-gradient functional for both the self-consistent field steps and the geometry optimizations. For the optimizations, we used a Γ-centered 2 × 2 × 1 k-point mesh and a 400 eV plane-wave cutoff. Atoms and cell parameters were relaxed with a conjugate-gradient algorithm until the residual forces were below 0.02 eV/Å. To avoid spurious interactions between periodic images, we added a 10–15 Å vacuum layer along the z direction. We placed the polymer atop the perovskite slab and made it laterally periodic, so its repeat matches the perovskite lattice.

We evaluated tin migration barriers by tracking how the energy changes as the tin ion moves along its migration pathway. First, we optimized the initial and final structures: a tin ion above the polymer and tin at the polymer-perovskite interface. We then generated intermediate geometries by linearly interpolating between them and mapped the pathway with the nudged elastic band (NEB) method and constrained minimization, using 16 images in total. The energy barrier height was taken as the energy difference between a minimum-energy state and the saddle point along the path.

## Supplementary information

Supplementary Information

Transparent Peer Review file

## Source data

Source data

## Data Availability

The data generated in this study are provided in the Supplementary Information and Source Data file. Source data are provided with this paper.
