# Supplementary material for: Mitigating tin ion migration and reinforcing buried interface via synergistic polymer-modified tin oxide
Source: Nat Commun. 2026 Sep 11;17:10723. doi: 10.1038/s41467-026-77624-8 (PMC13658243; doi:10.1038/s41467-026-77624-8)
Supplement: Supplementary file 1 [file 41467_2026_77624_MOESM1_ESM.pdf]

Supplementary Information for

Mitigating tin ion migration and reinforcing buried interface via synergistic polymer-modified tin oxide

Wenxin Yang[1†], Yepin Zhao[1†*], Zhen-Yang Suo[2†], Yu-Che Lin[1], Minhuan Wang[3], Weixuan Huang[4], Jian-Guo Zheng[5], Joo-Hong Lee[6,7], Mingjie Xu[5], Yiushun Tong[1], Zhichao Shen[1], Dong Meng[1], Ran Zheng[1], Ming Gong[1], Yingke Zhu[1], Ying Zhang[1], Haoxiang Duan[1], Elizabeth Zhang[8], Yuran Shi[8], Chia-Yu Lin[8], Aman Dhillon[8], Jinsung Kim[1], Tso-An Yang[1], Anatoly I. Frenkel[4], Xiaoqing Pan[5], Ilhan Yavuz[9*], Jing Cao[2*], Jin-Wook Lee[7,10], Yang Yang[1*]

Supplementary Figures

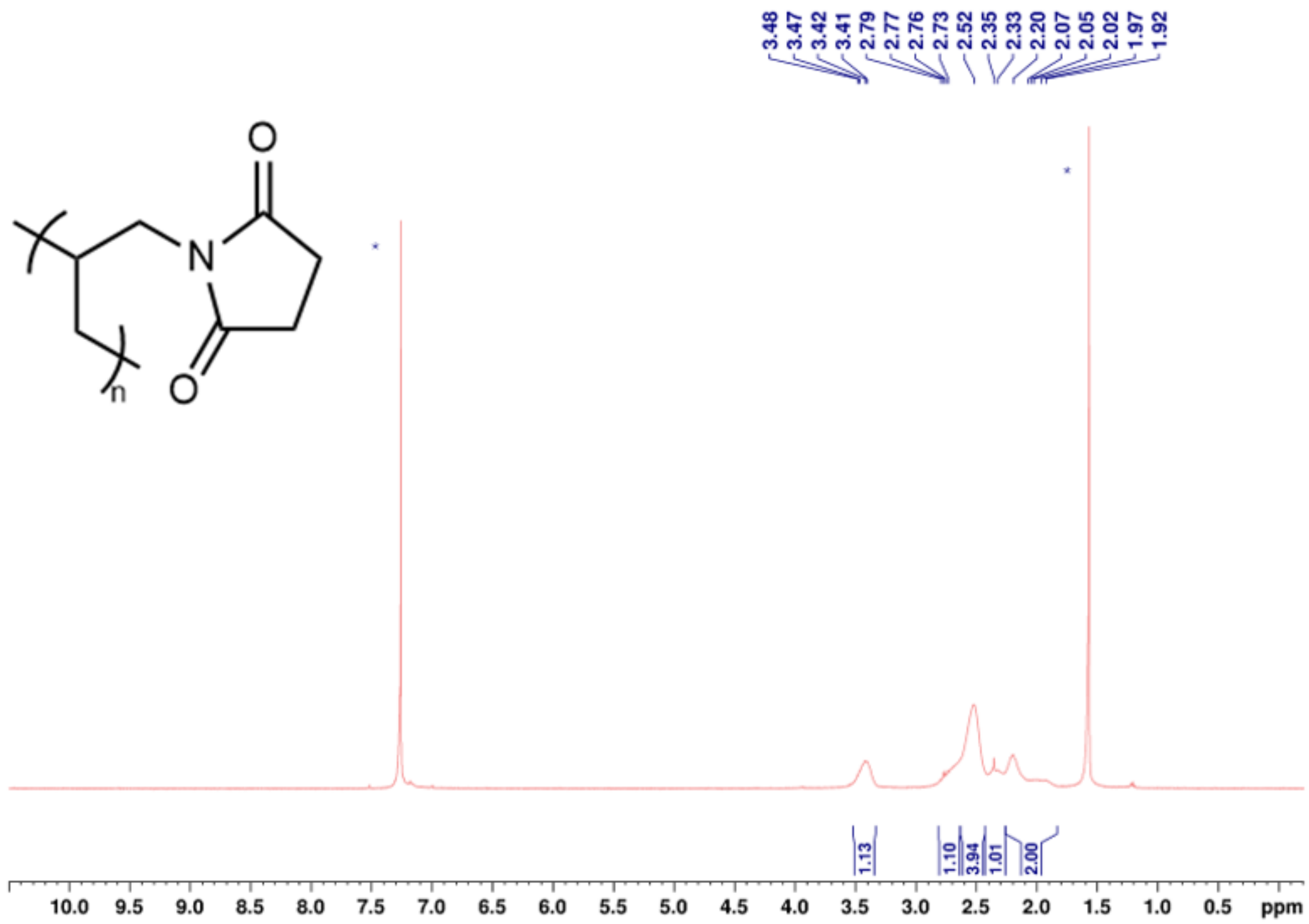

Supplementary Fig. 1 | ^{1}H NMR of PED

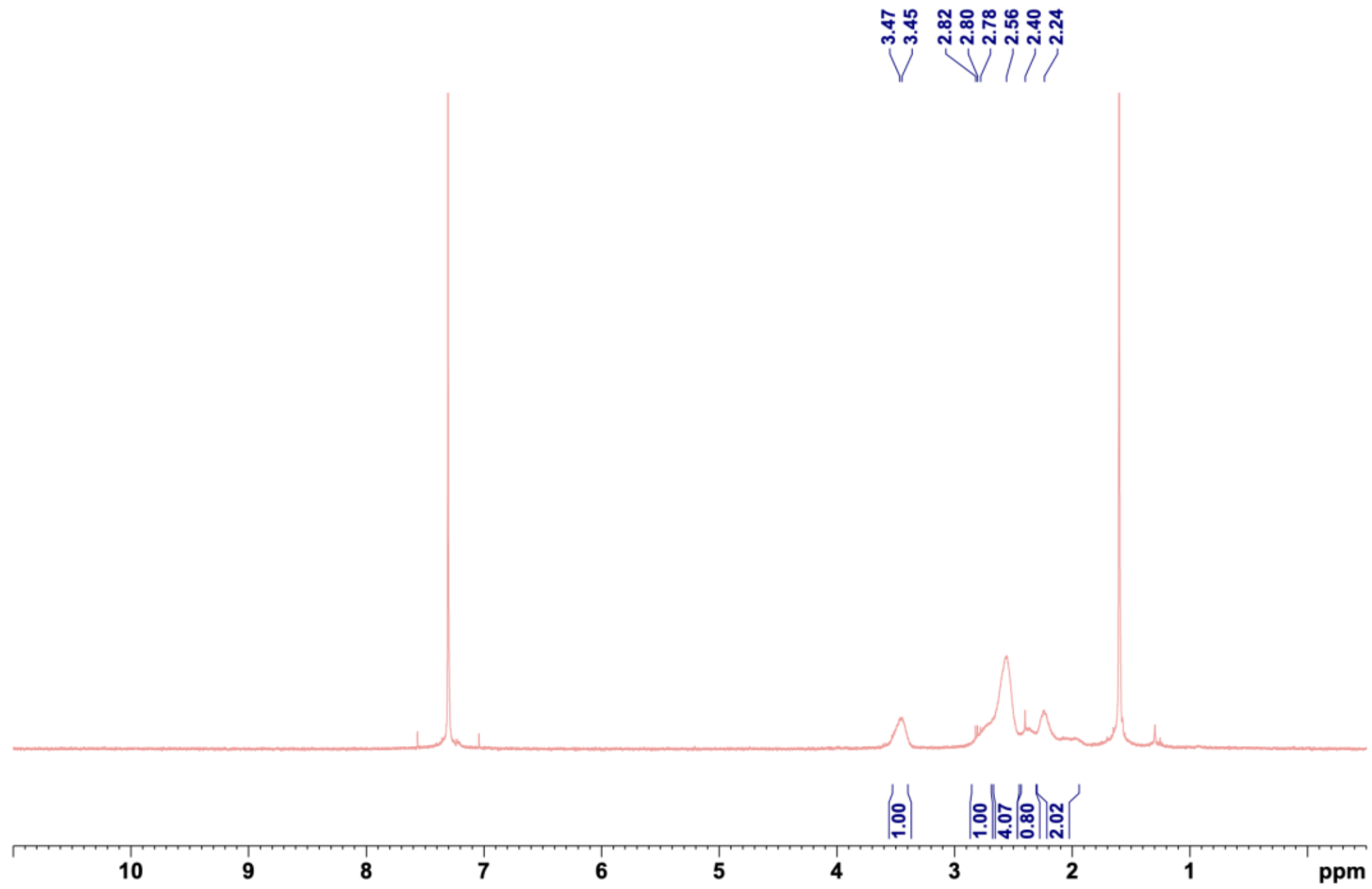

Supplementary Fig. 2 | ^{1}H NMR of PED after annealing at 150°C for 13 minutes

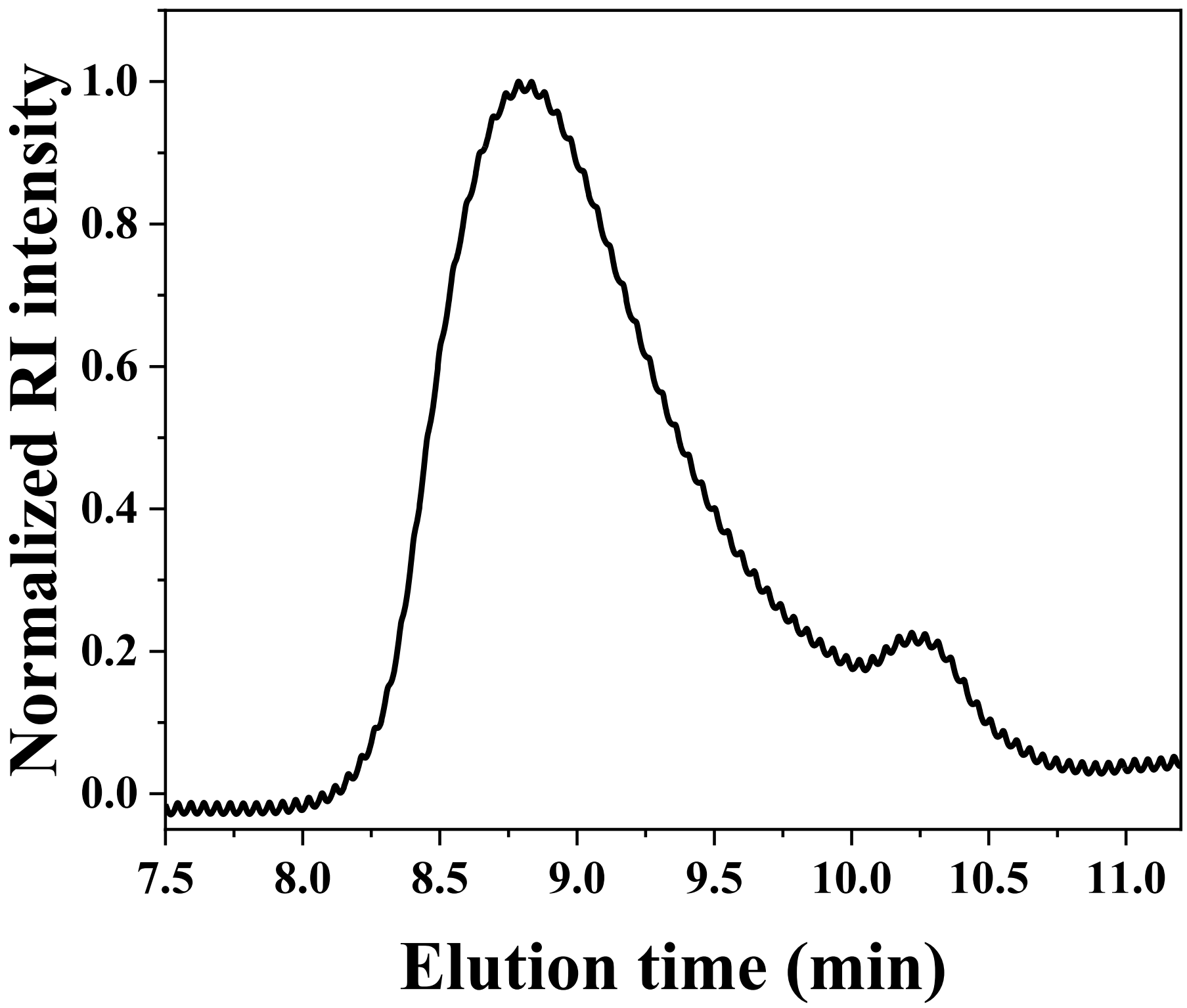

Supplementary Fig. 3 | GPC characterization of purified PED

The chromatogram is dominated by a main polymer peak. The purified PED exhibits M_n = 5,033, M_w = 7,465, and $Đ = M_w/M_n$ = 1.48. RI, refractive index.

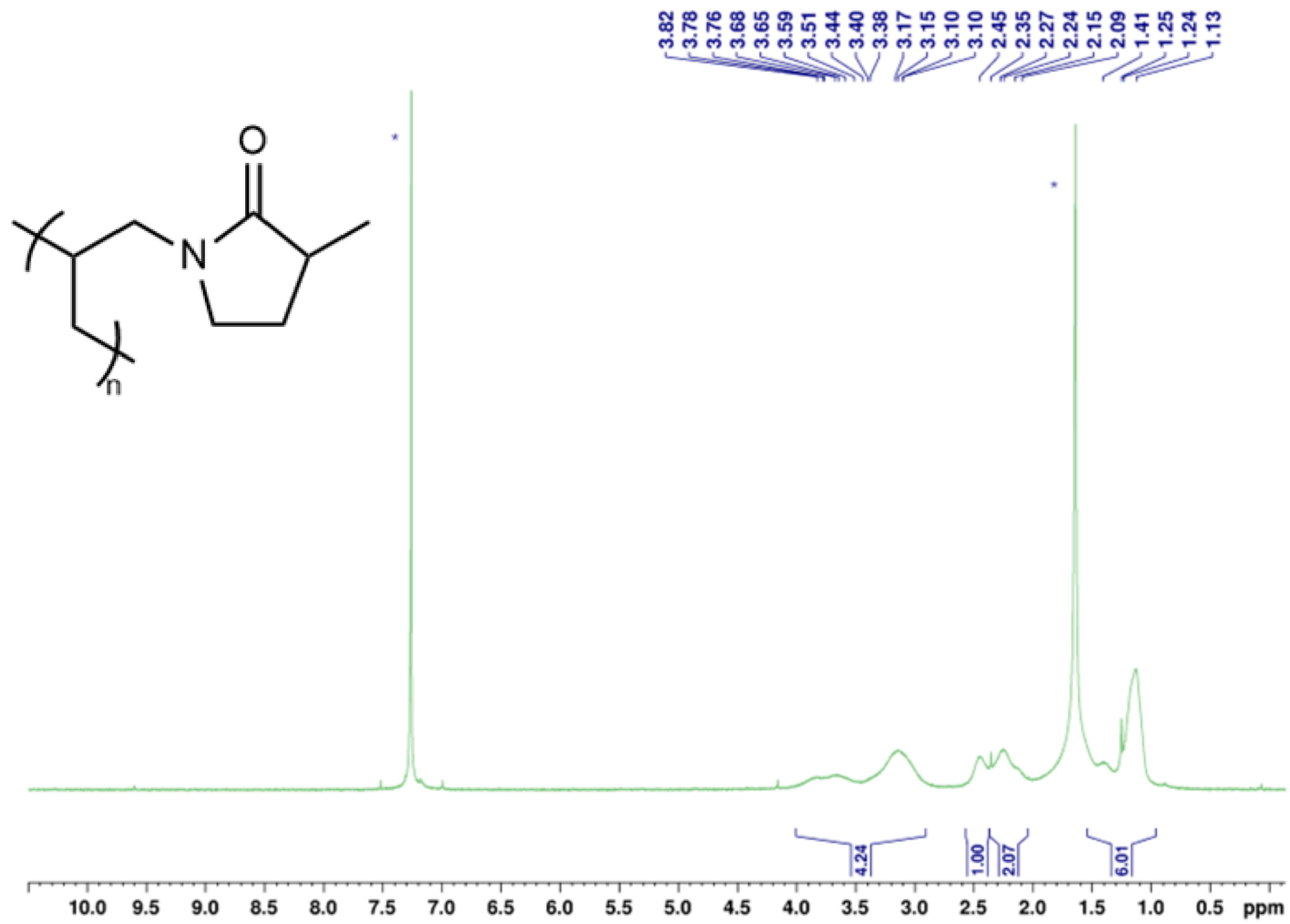

Supplementary Fig. 4 | ^{1}H NMR of PEM

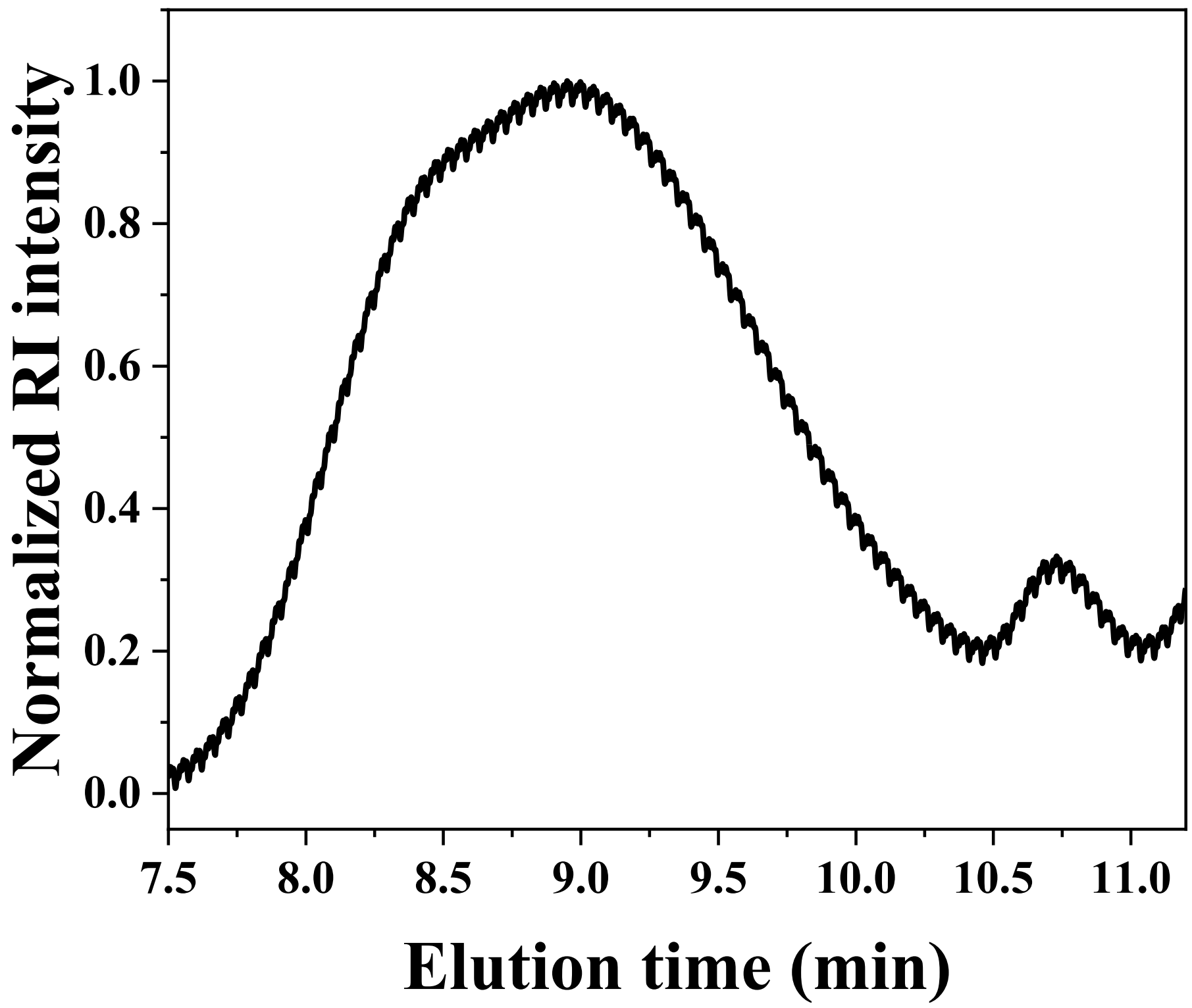

Supplementary Fig. 5 | GPC characterization of purified PEM

The chromatogram is dominated by a main polymer peak. The purified PEM exhibits M_n = 16304, M_w = 62018, and $Đ = M_w/M_n$ = 3.804.

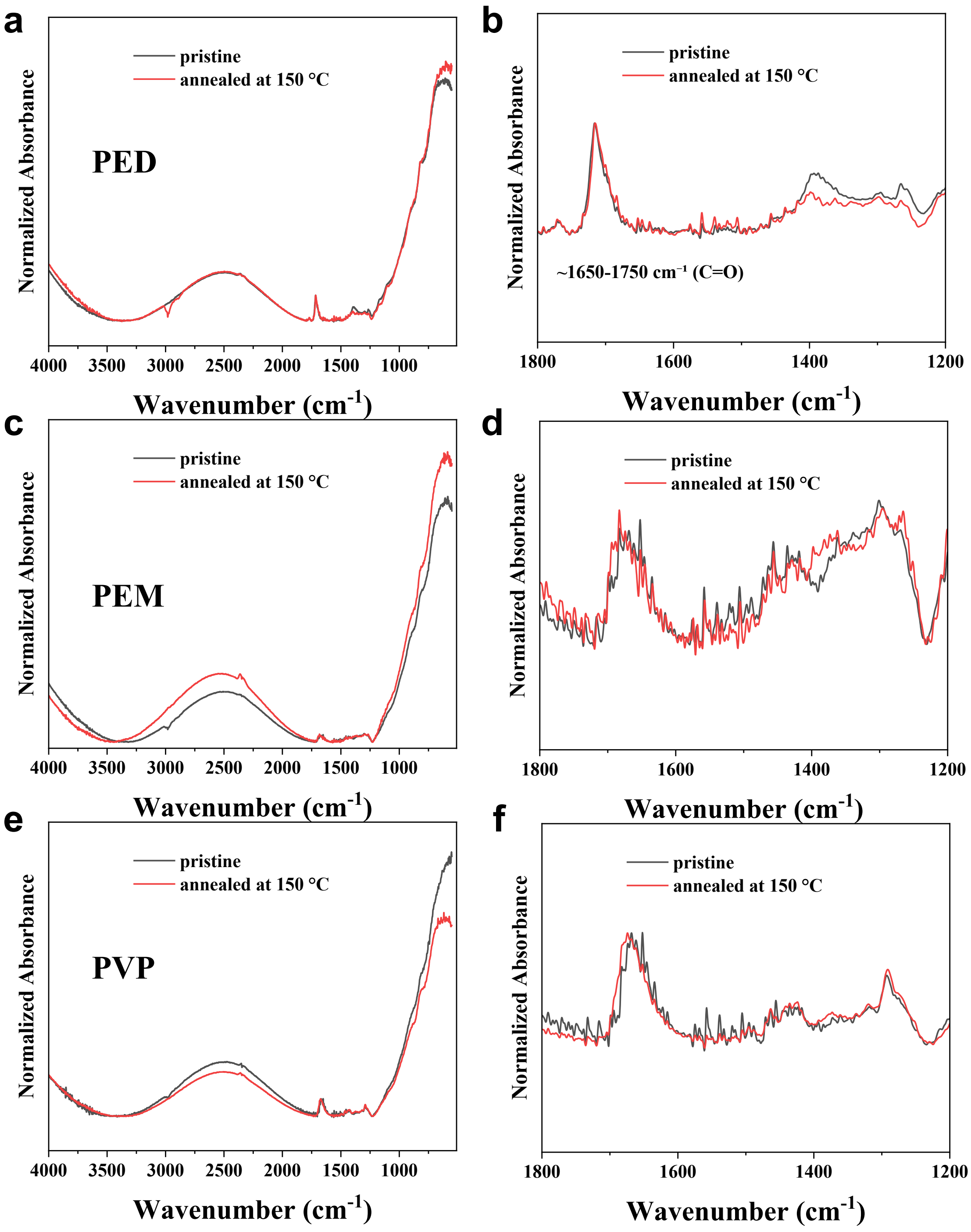

Supplementary Fig. 6 | ATR-FTIR characterization of polymer thermal stability under the perovskite annealing condition

a, c, e, Full-range normalized absorbance FTIR spectra of PED, PEM, and PVP, respectively, before and after annealing at 150 °C. **b, d, f,** Corresponding enlarged spectra in the 1800–1200 cm^{-1} region, covering the carbonyl-related and adjacent fingerprint regions. The spectra were baseline-corrected and normalized for comparison of peak positions and characteristic vibrational features.

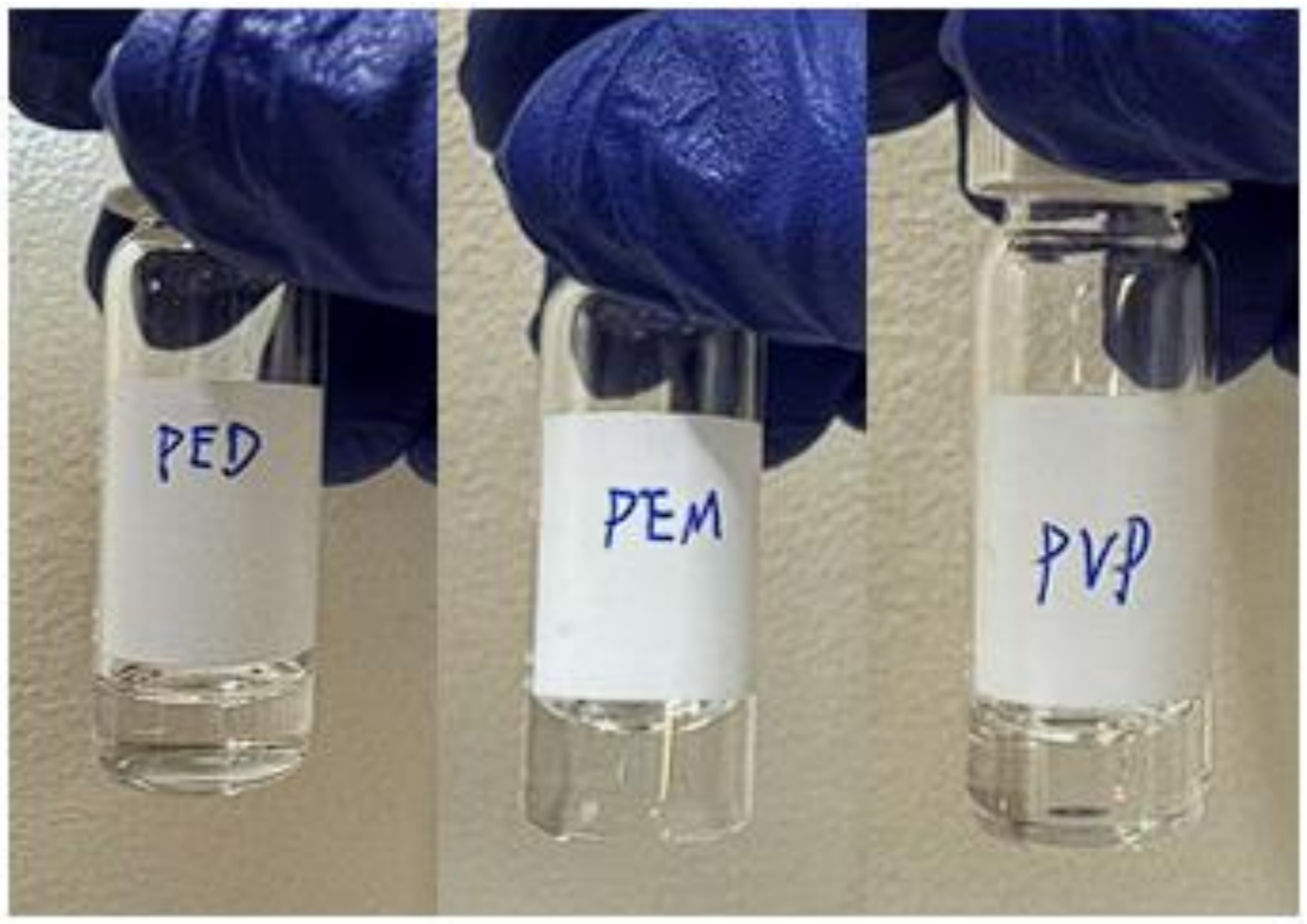

Supplementary Fig. 7 | Solution of PED, PEM and PVP with a concentration of 1mg ml^{-1}

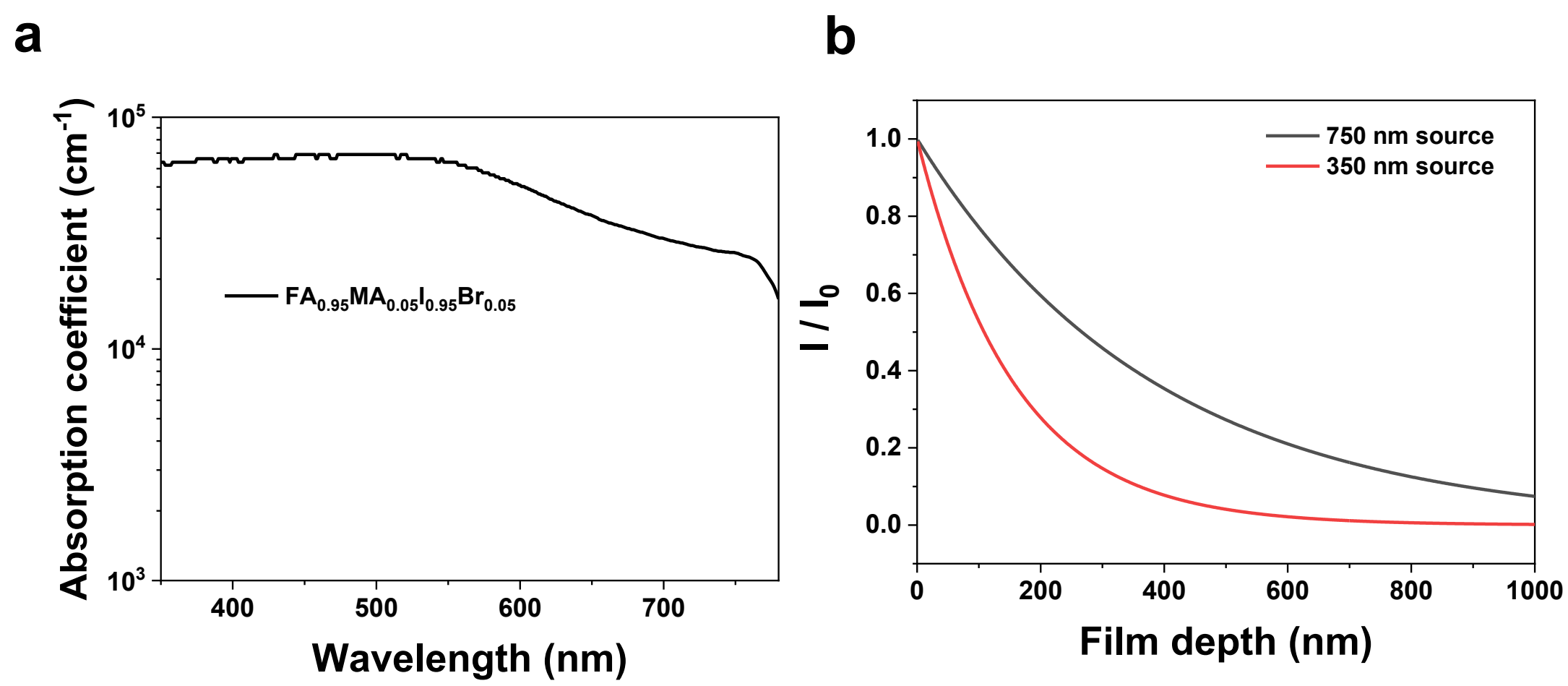

Supplementary Fig. 8 | Absorption spectra of perovskite film

a, Absorption coefficient of $FA_{0.95}MA_{0.05}I_{0.95}Br_{0.05}$. **b,** Depth dependent intensity attenuation of incident light (I_0) with different light source wavelength. I and I_0 denote the light intensity at a given film depth and the incident light intensity, respectively.

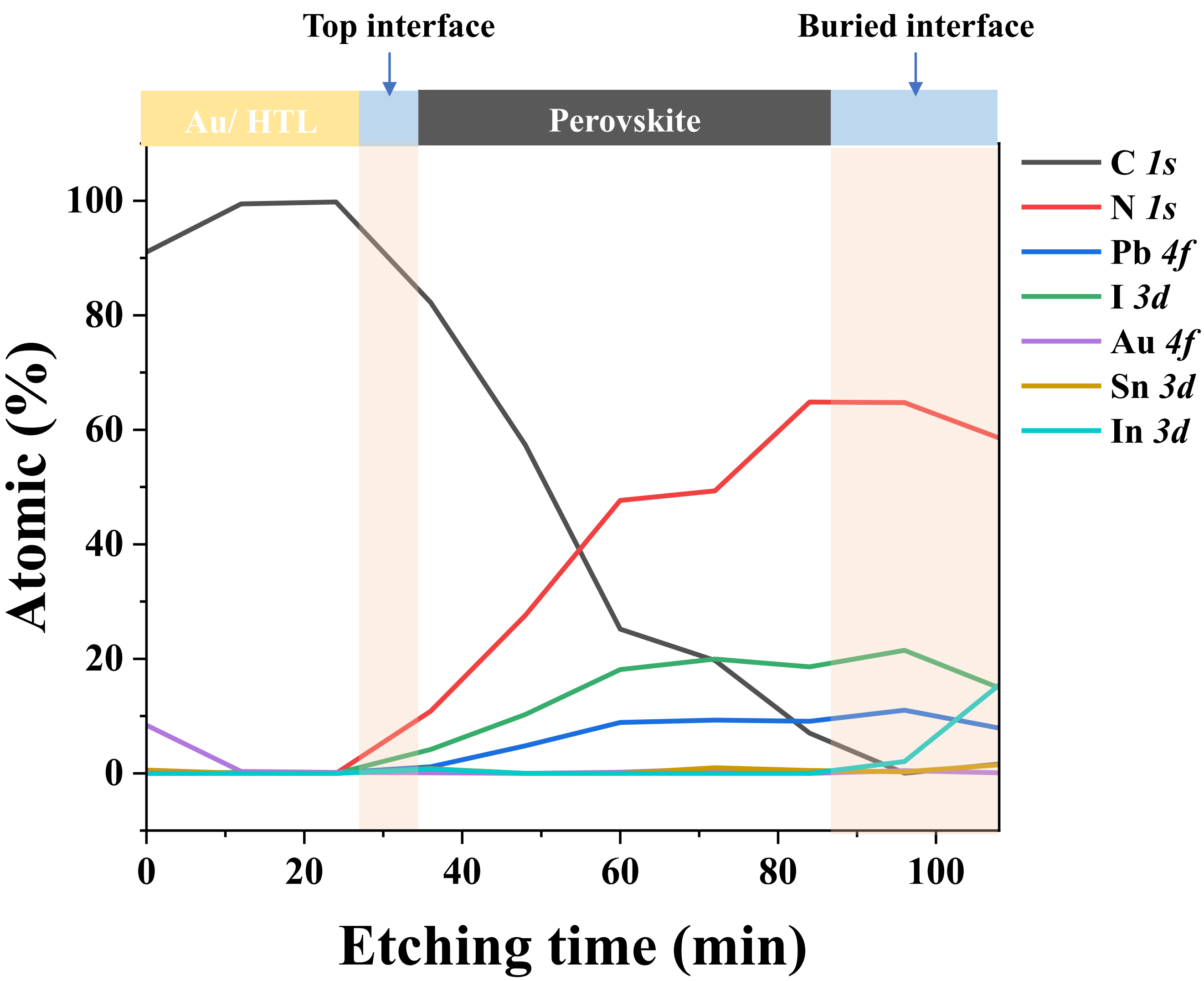

Supplementary Fig. 9 | Interface-focused XPS depth profile of the fresh device

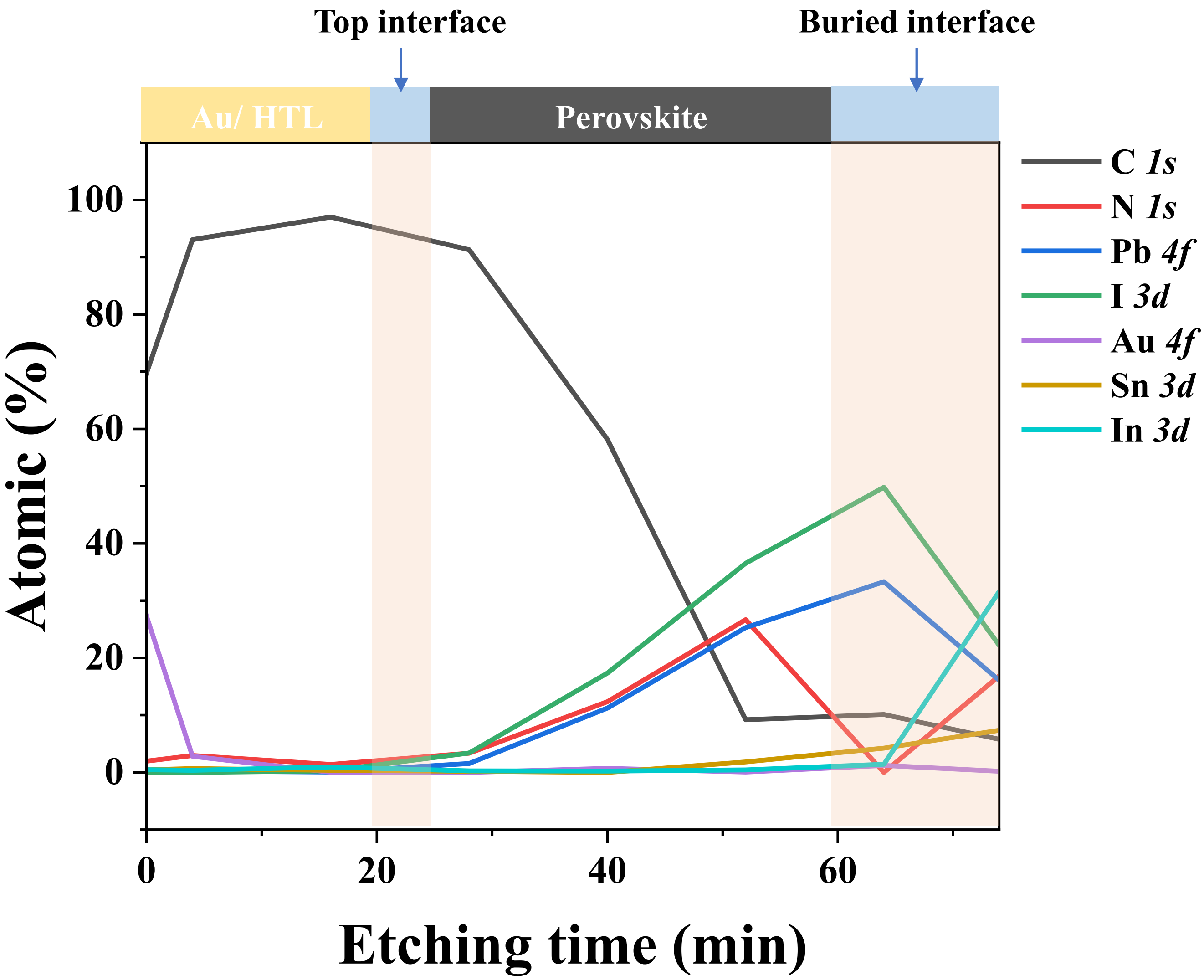

Supplementary Fig. 10 | Interface-focused XPS depth profile of the aged device

Aging condition: encapsulated device under one-sun illumination for 170 hours.

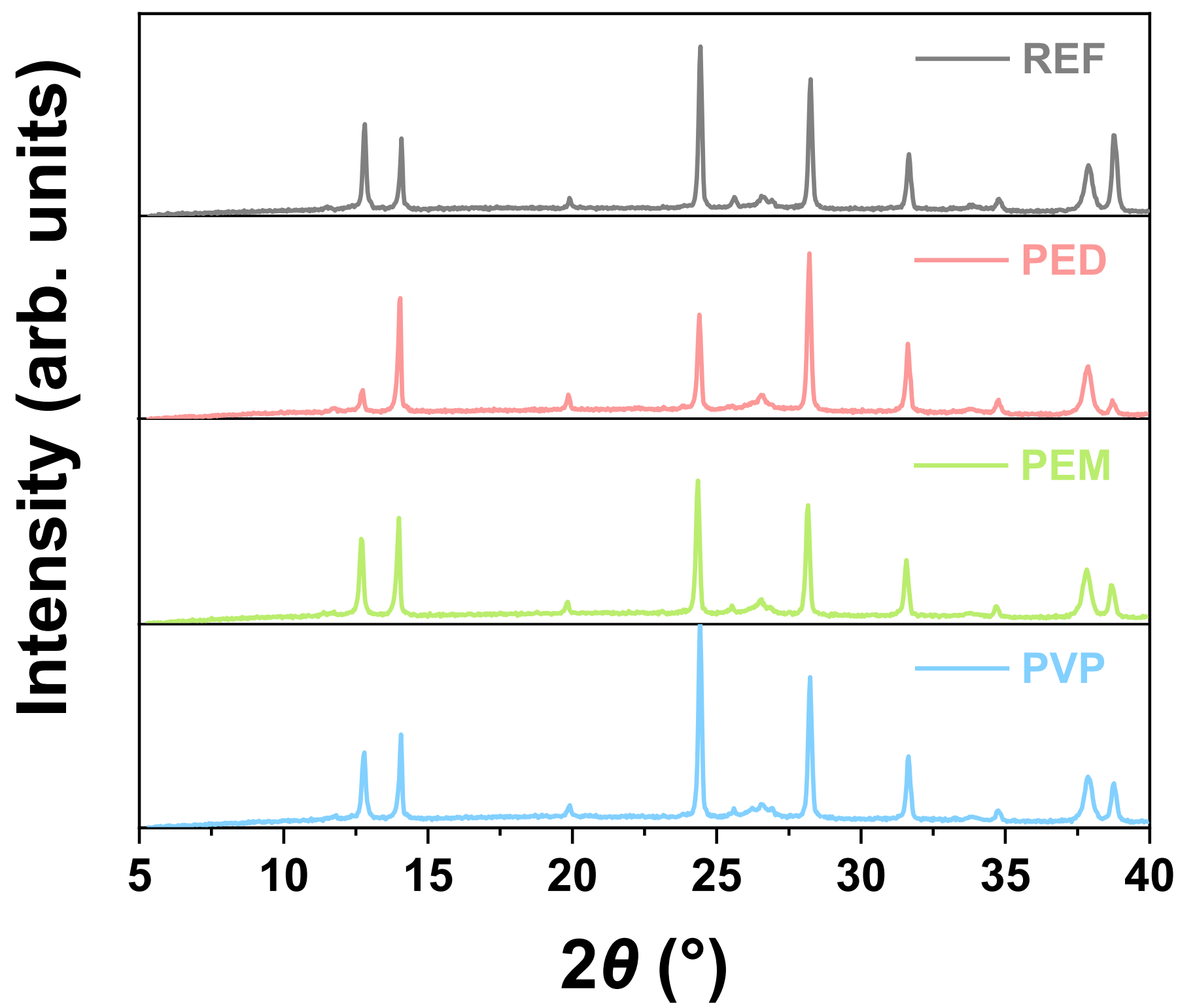

Supplementary Fig. 11 | XRD patterns of fresh samples

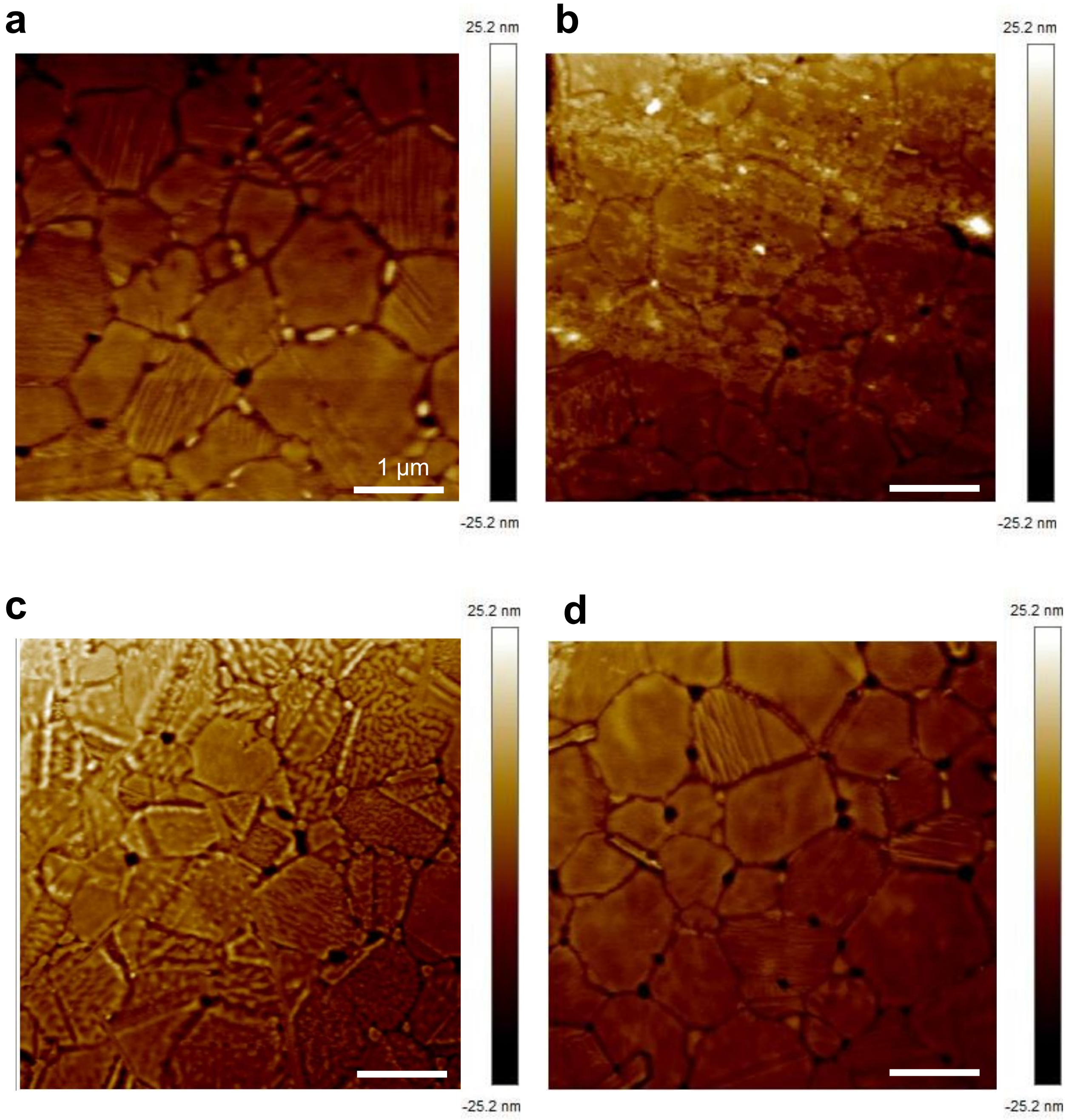

Supplementary Fig. 12 | AFM images for buried interface peeled from fresh device

a, REF; **b,** with PED interlayer; **c,** with PEM interlayer; **d,** with PVP interlayer (scale bar, 1 μm).

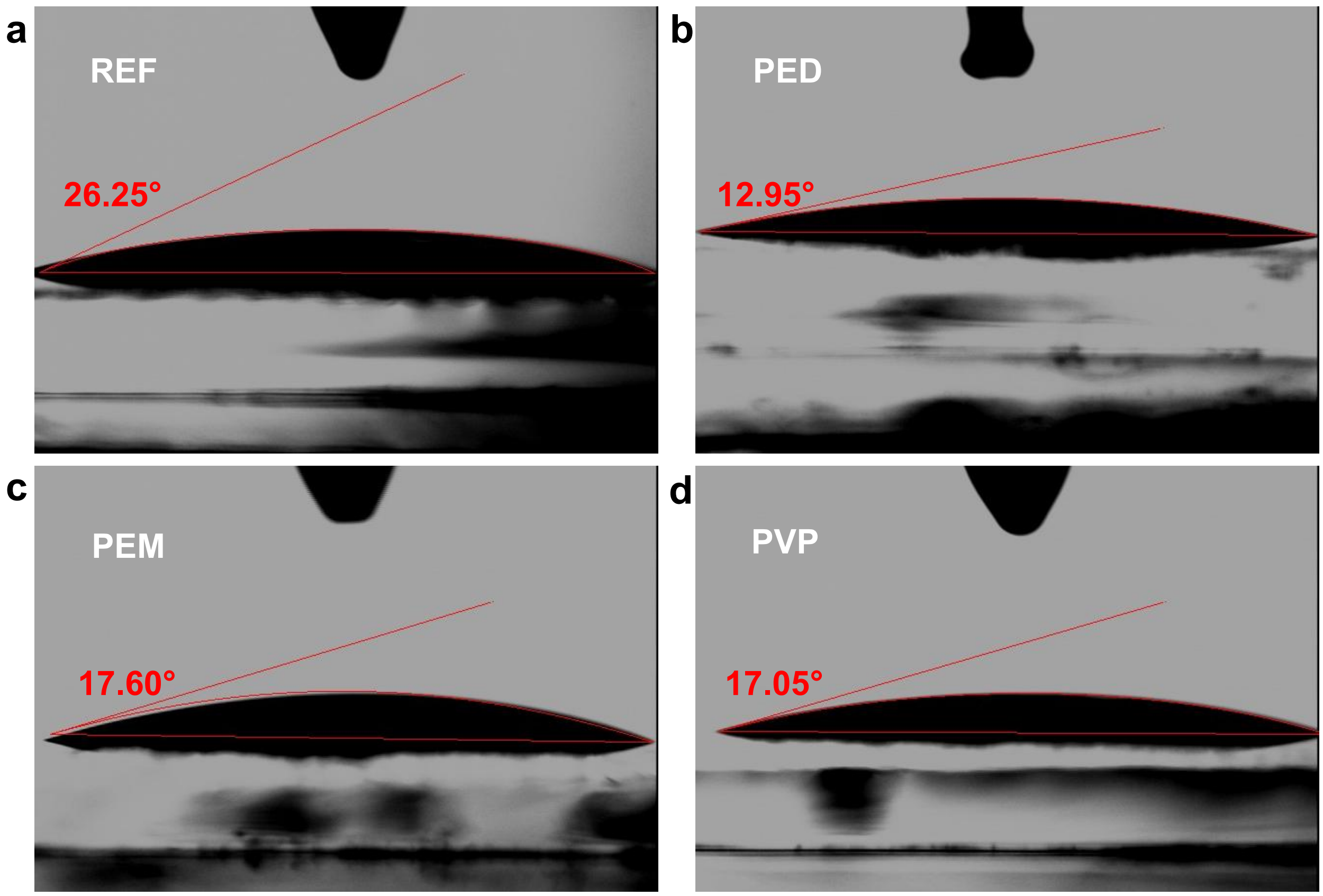

Supplementary Fig. 13 | Contact angle of water on different surfaces

The structure is Glass/ITO/SnO2 with/without polymer interlayers.

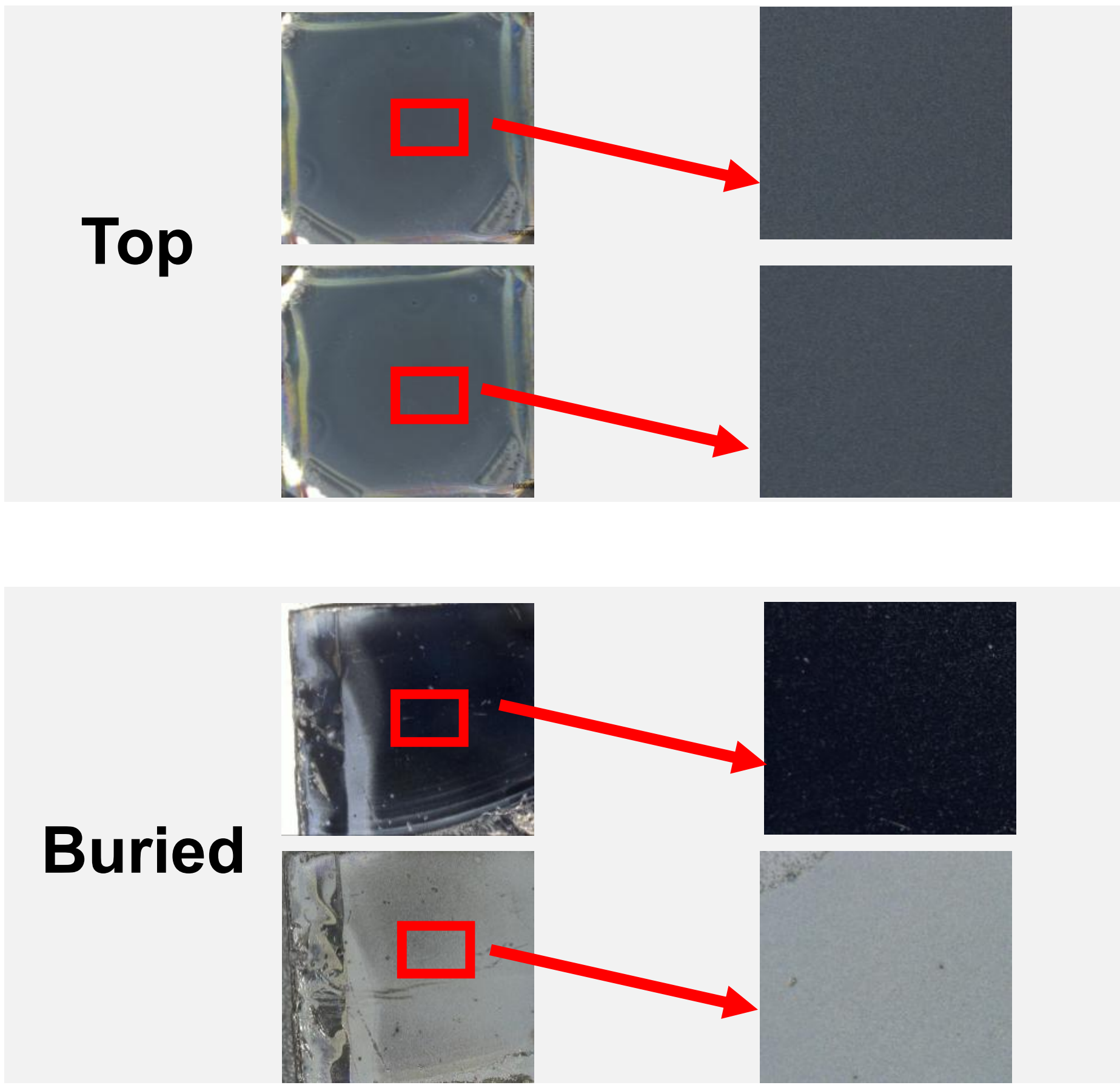

Supplementary Fig. 14 | Optical images of top and buried interface comparison before and after degradation

The aging condition is incandescent lamp illumination under ambient conditions (~40% humidity, room temperature).

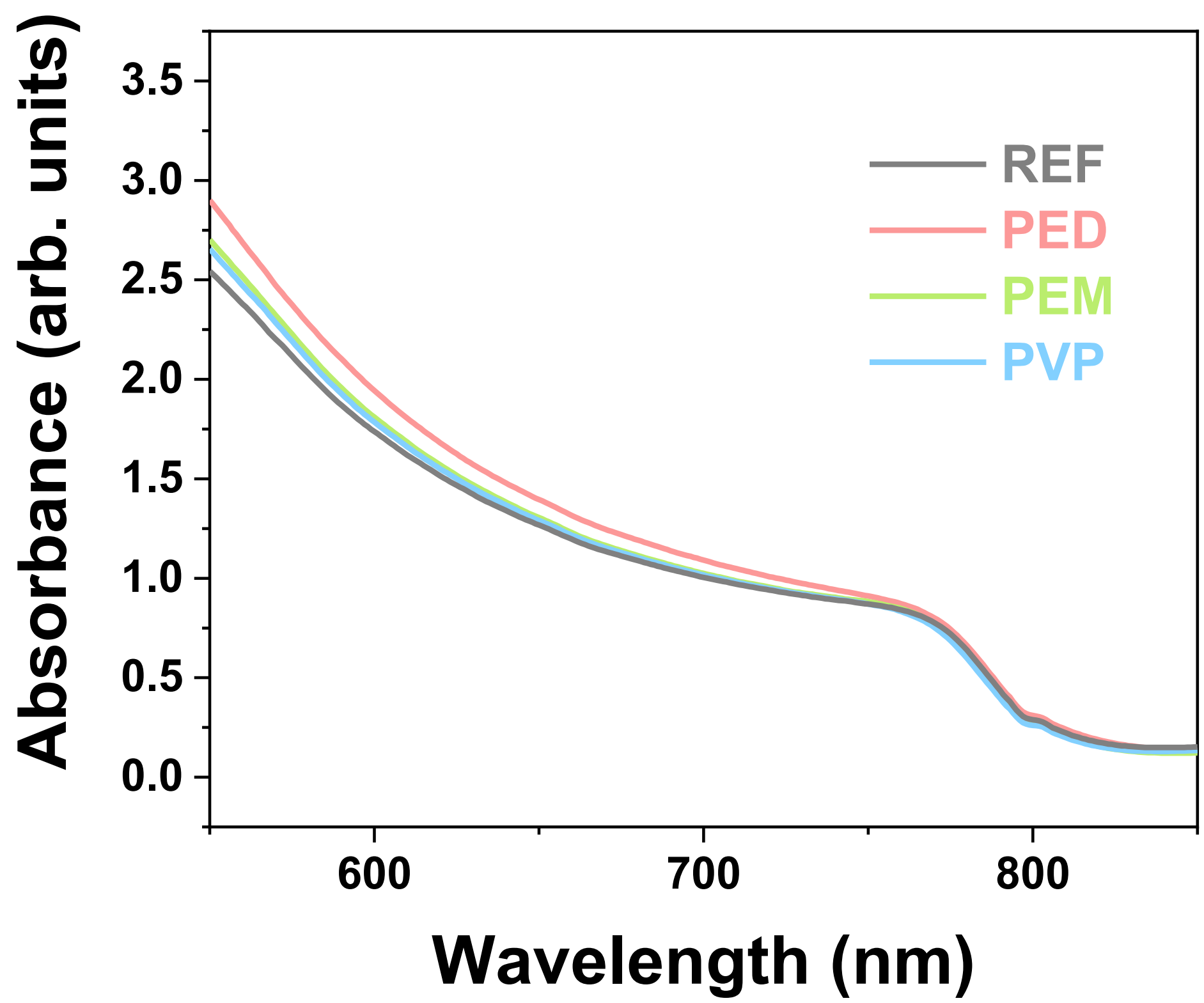

Supplementary Fig. 15 | UV-vis spectra of perovskite thin film

Films are deposited on ITO/ (polymeric interlayers) substrate.

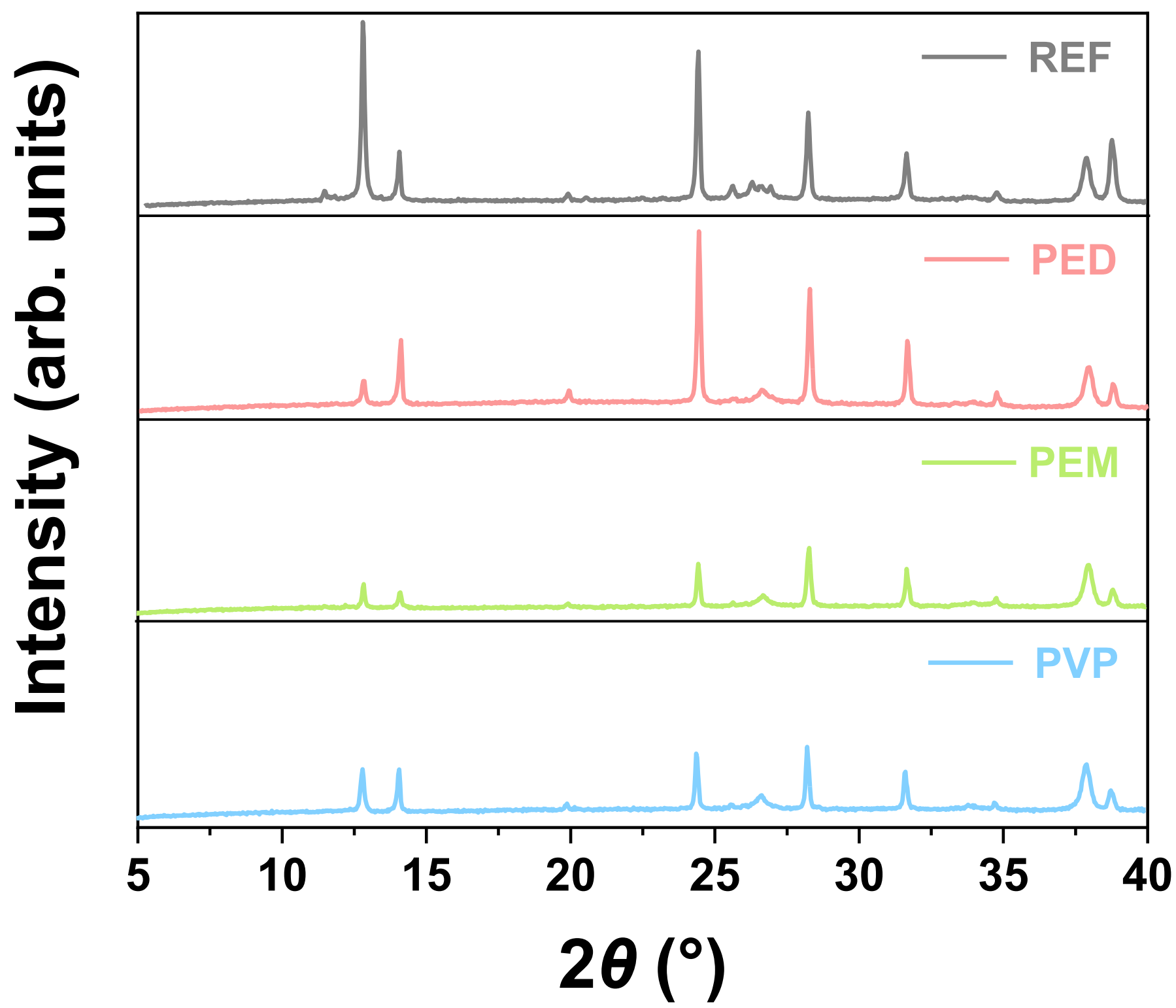

Supplementary Fig. 16 | XRD patterns of aged samples

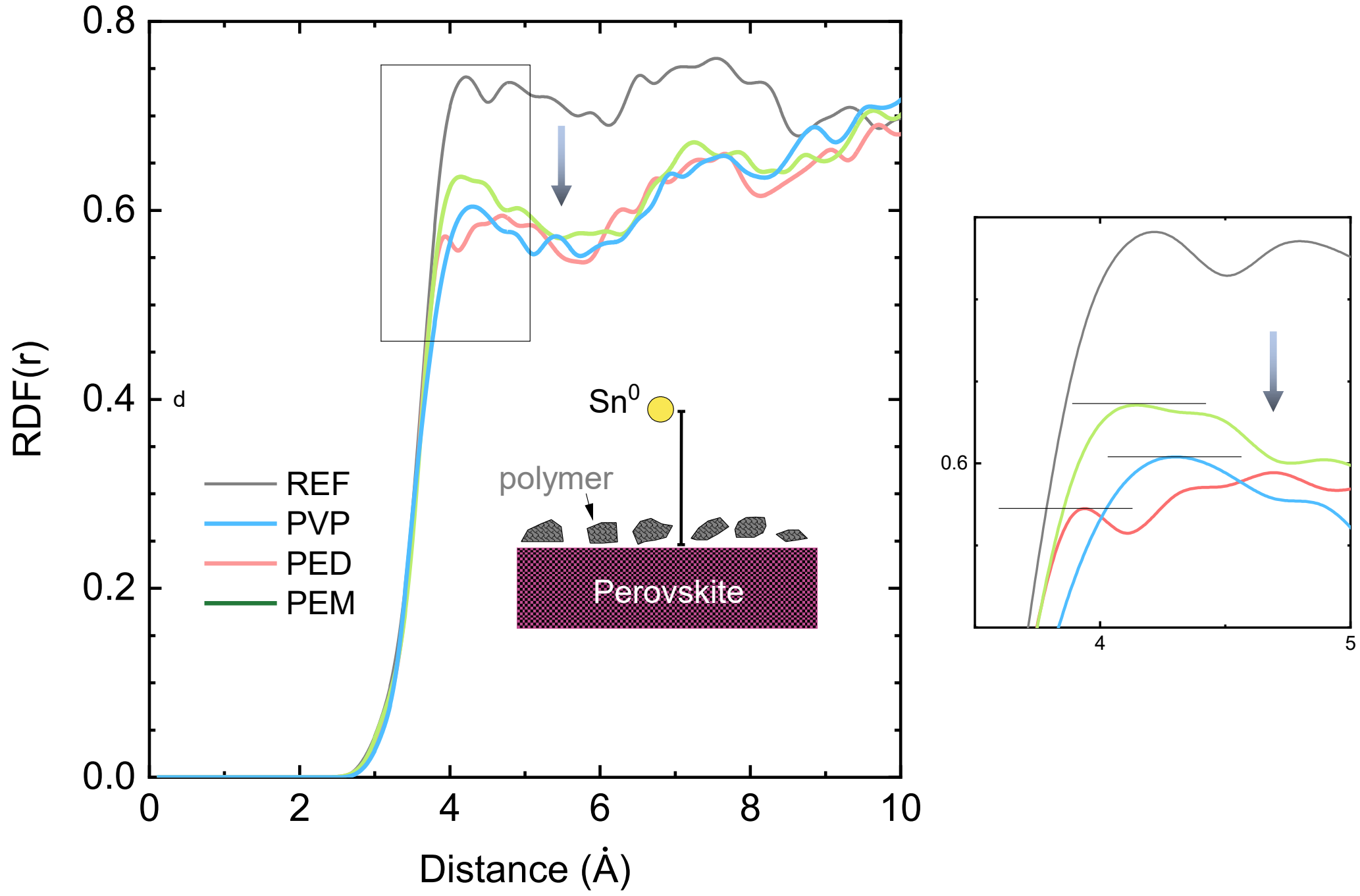

Supplementary Fig. 17 | MD simulations for Sn migration across different polymers

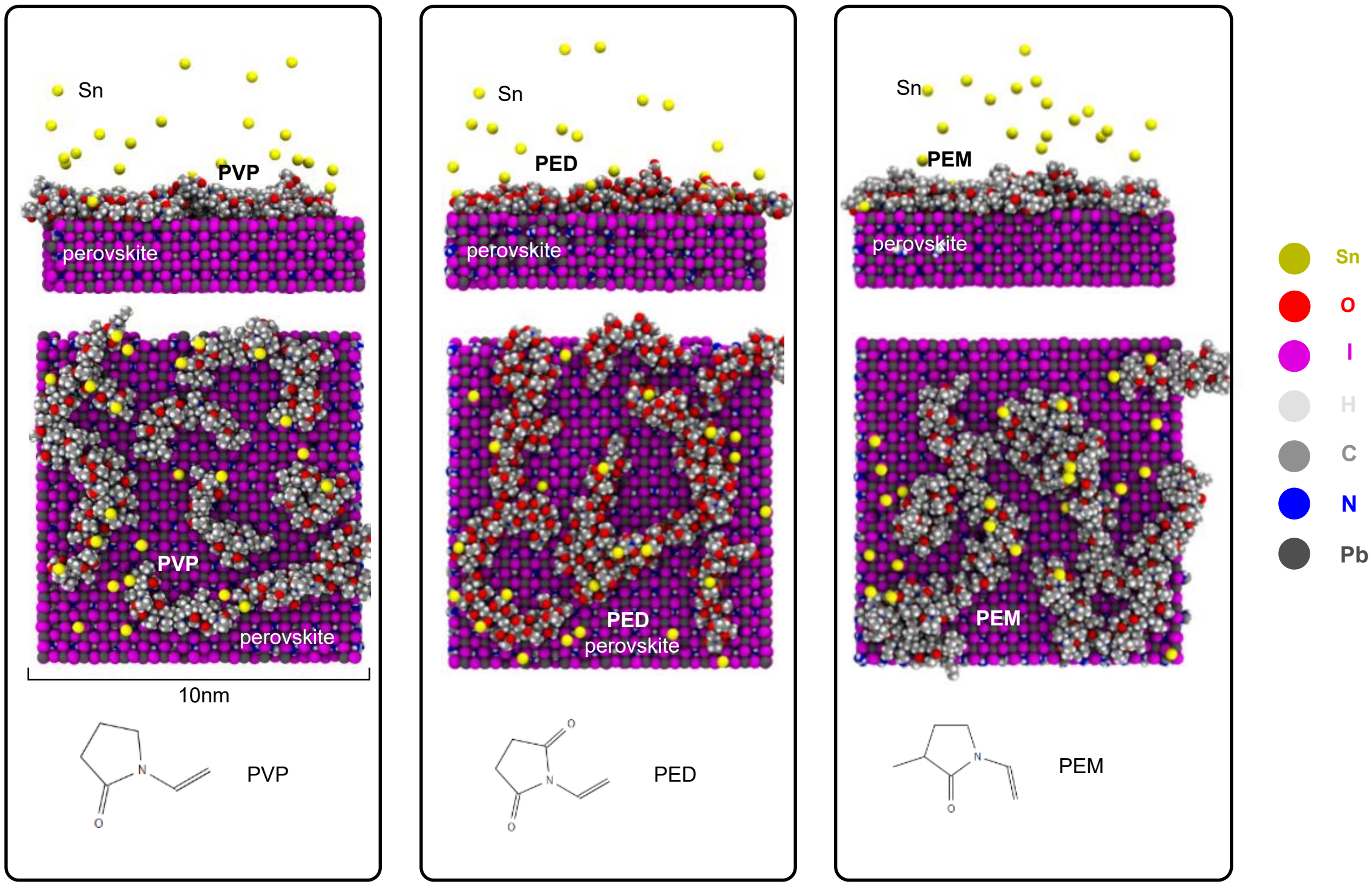

Supplementary Fig. 18 | MD simulations for Sn migration across different polymers

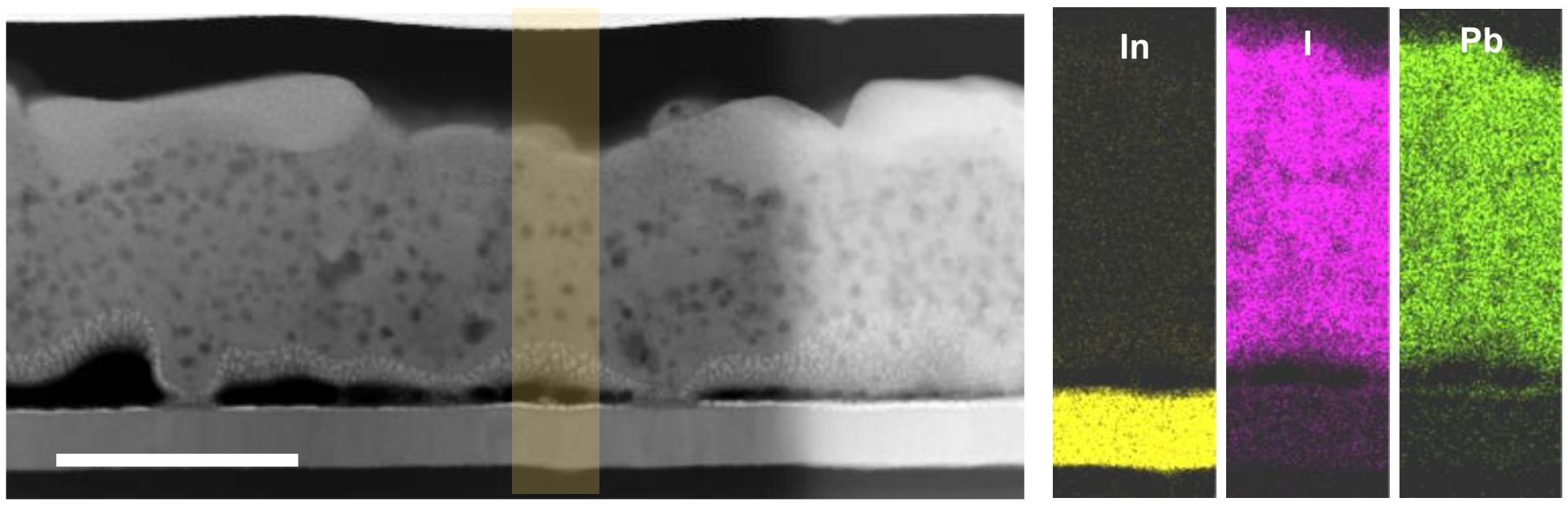

Supplementary Fig. 19 | STEM image and EDS elemental maps for an aging reference device EDS elemental maps of In, I and Pb are from the highlighted image area (scale bar, 500 nm).

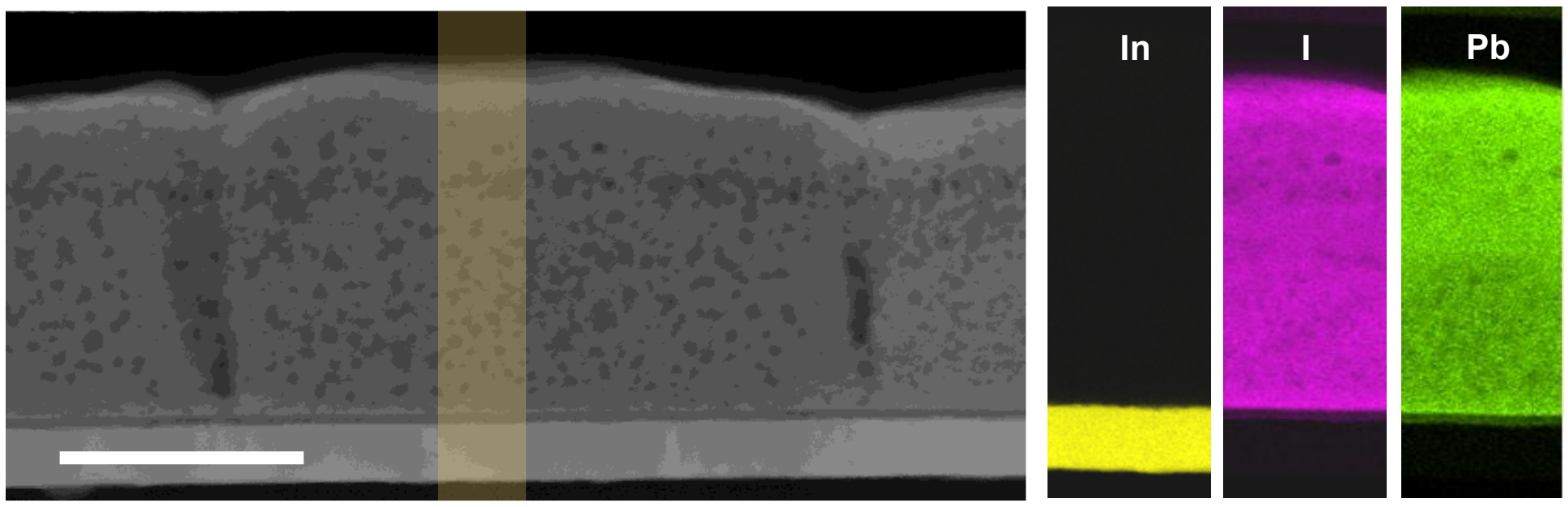

Supplementary Fig. 20 | STEM image and EDS elemental maps for an aging reference device with PED interlayer

EDS elemental maps of In, I and Pb are from the highlighted image area (scale bar, 500 nm).

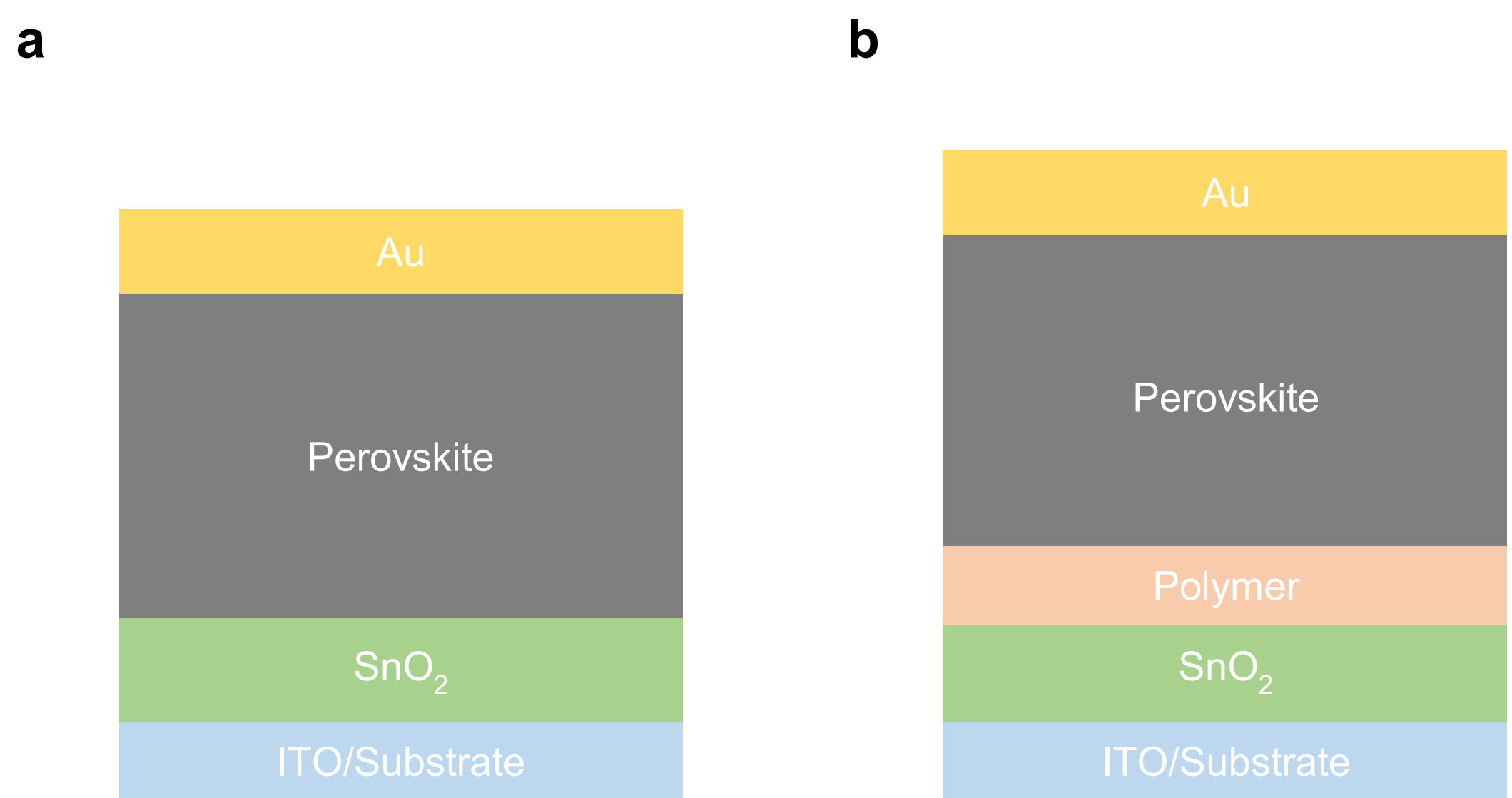

Supplementary Fig. 21 | Device illustration for temperature-dependent conductivity tests

a, reference device; **b,** device with different polymeric interlayers.

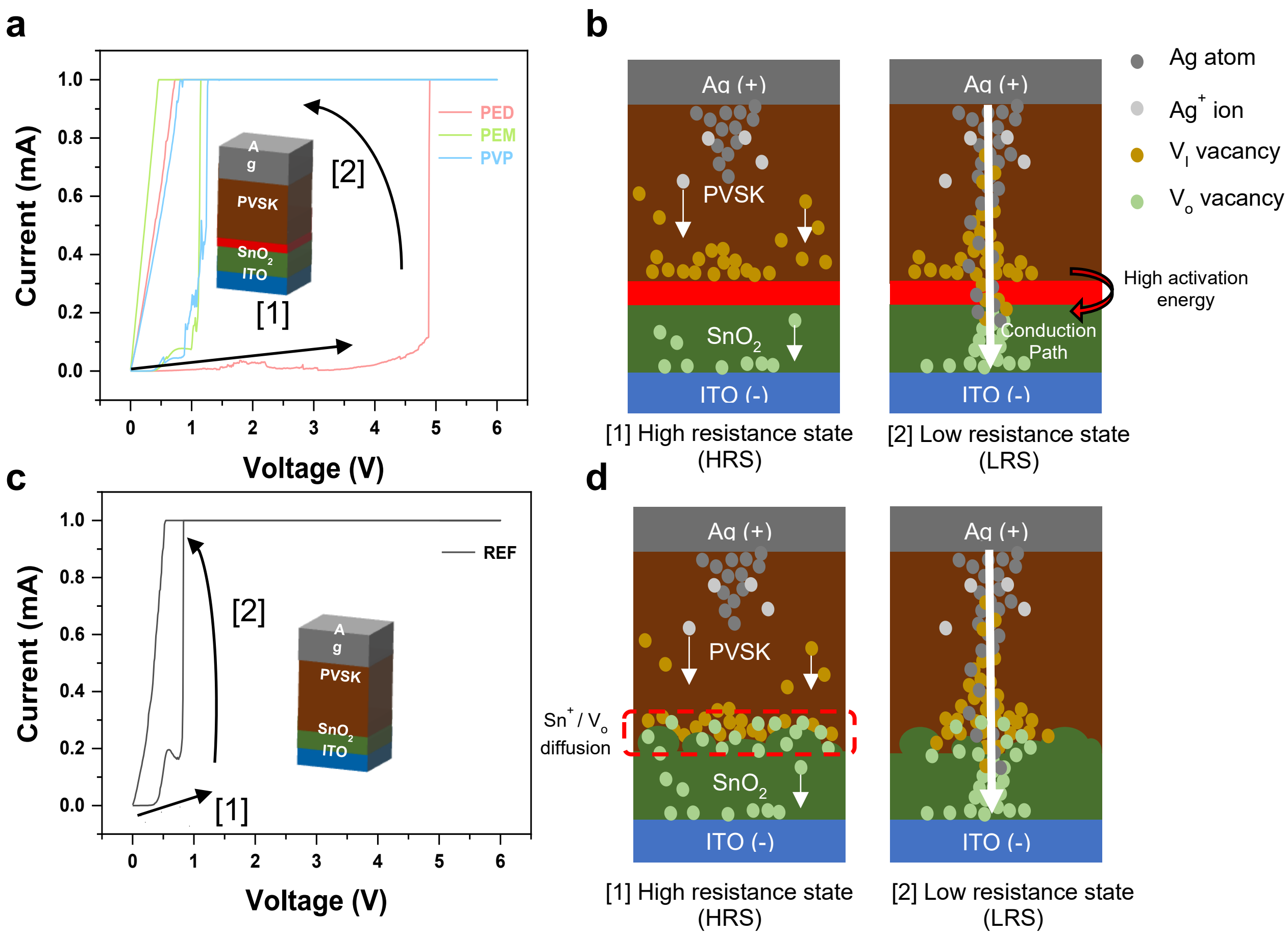

Supplementary Fig. 22 | *I-V* characteristics of resistivity switching measurement and corresponding schematics

a, b, with polymeric interlayers and **c, d** without layers. For the region of [1], the conductive filament is not fully formed, maintaining high resistance state (HRS). The resistance state is abruptly switched to low resistance state (LRS) at the region [2] due to the formation of conductive filaments via mobile ion migrations.

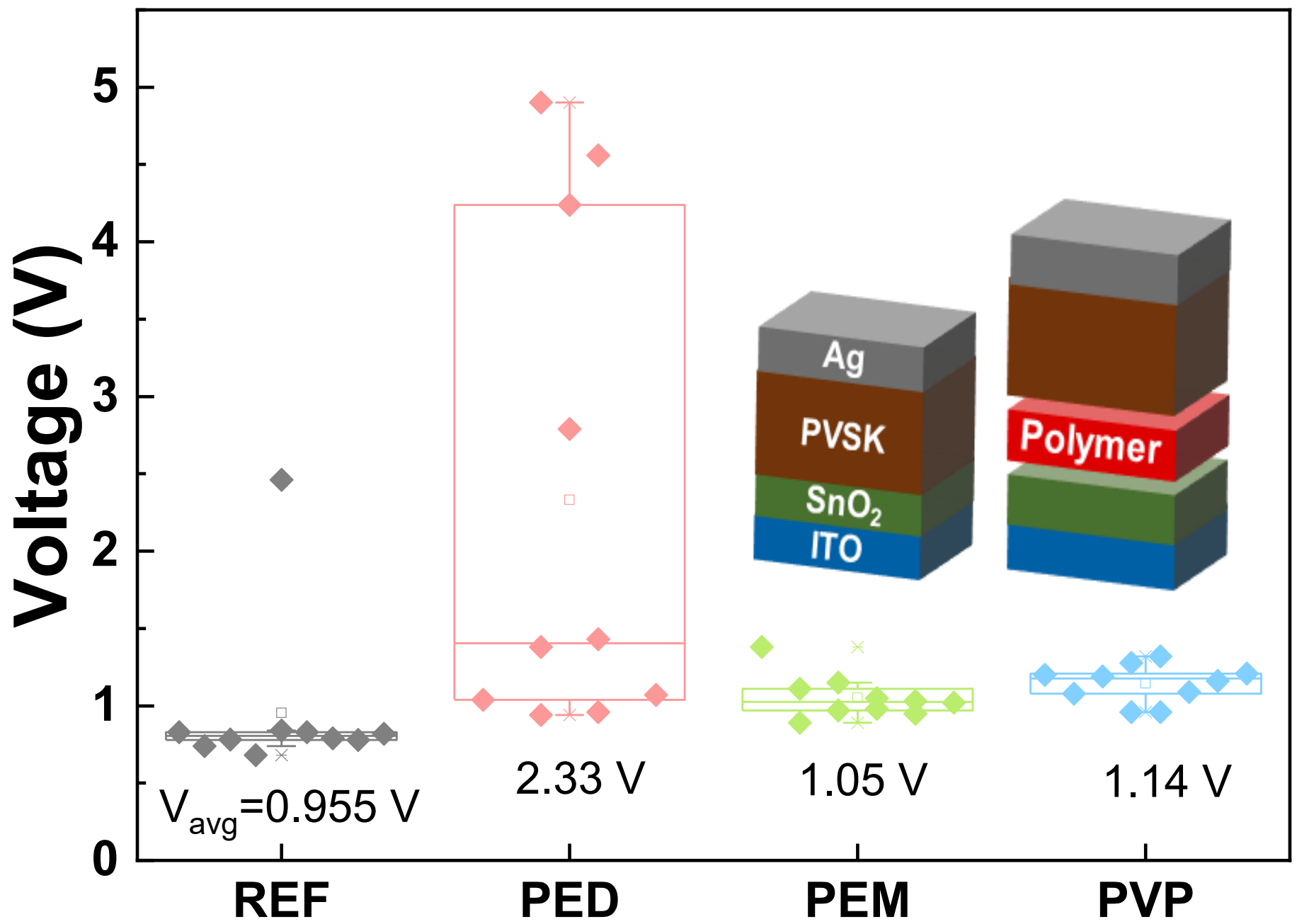

Supplementary Fig. 23 | Resistivity switching measurements for polymeric interlayers with SnO_2 Results are extracted from 10 different cells. The box plots show the median (center line), the 25th and 75th percentiles (box limits), the 1.5× the interquartile range (whiskers), the mean (open square), and the 1st and 99th percentiles (crosses). Individual data points are shown as diamonds.

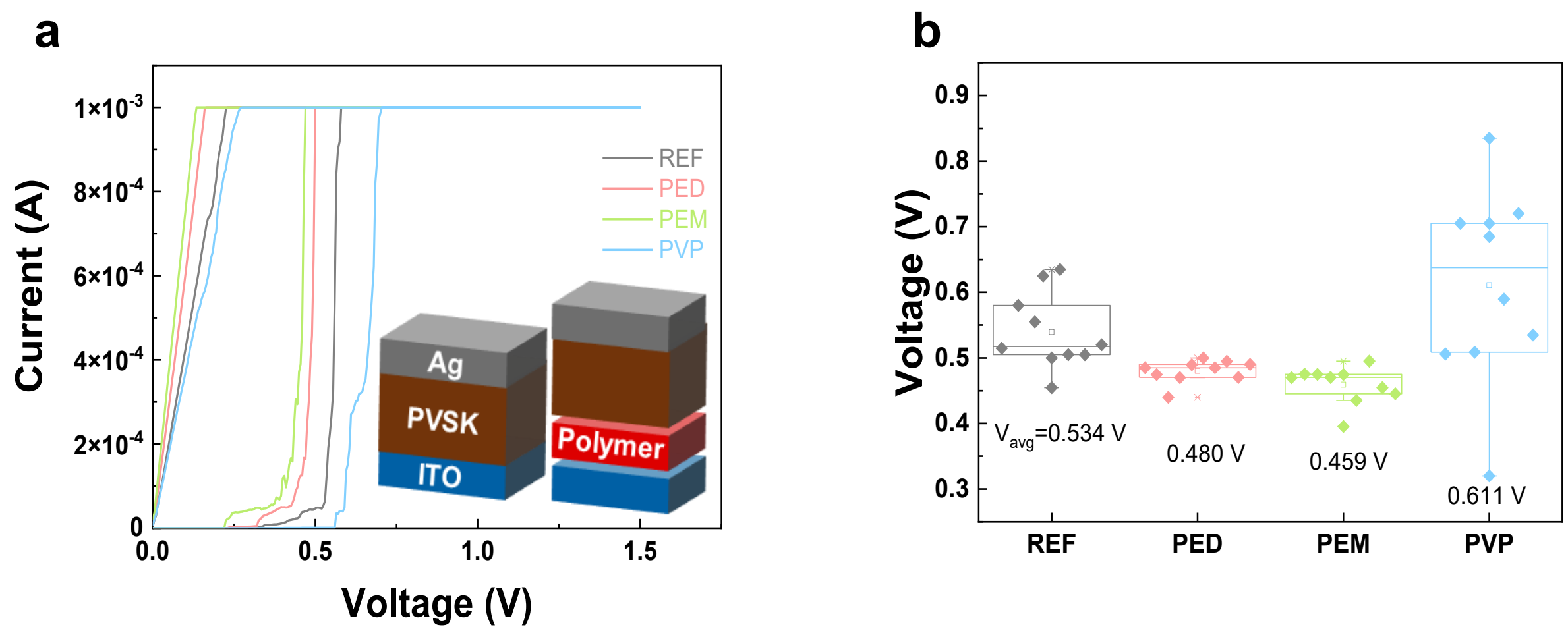

Supplementary Fig. 24 | Resistivity switching measurements for polymeric interlayers without SnO_2

Results are extracted from 10 different cells. The box plots show the median (center line), the 25th and 75th percentiles (box limits), the 1.5× the interquartile range (whiskers), the mean (open square), and the 1st and 99th percentiles (crosses). Individual data points are shown as diamonds.

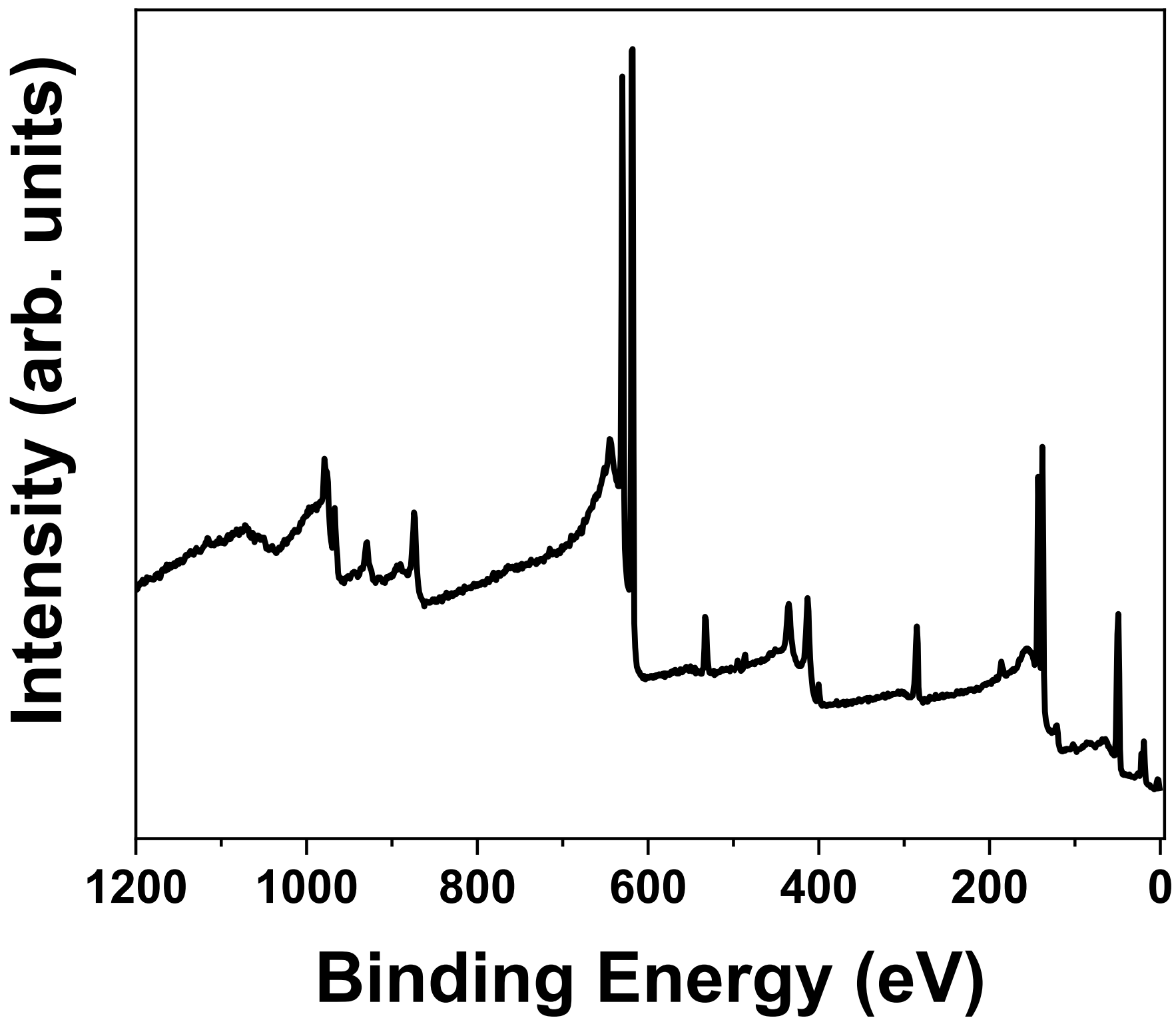

Supplementary Fig. 25 | XPS survey spectrum for aged reference sample

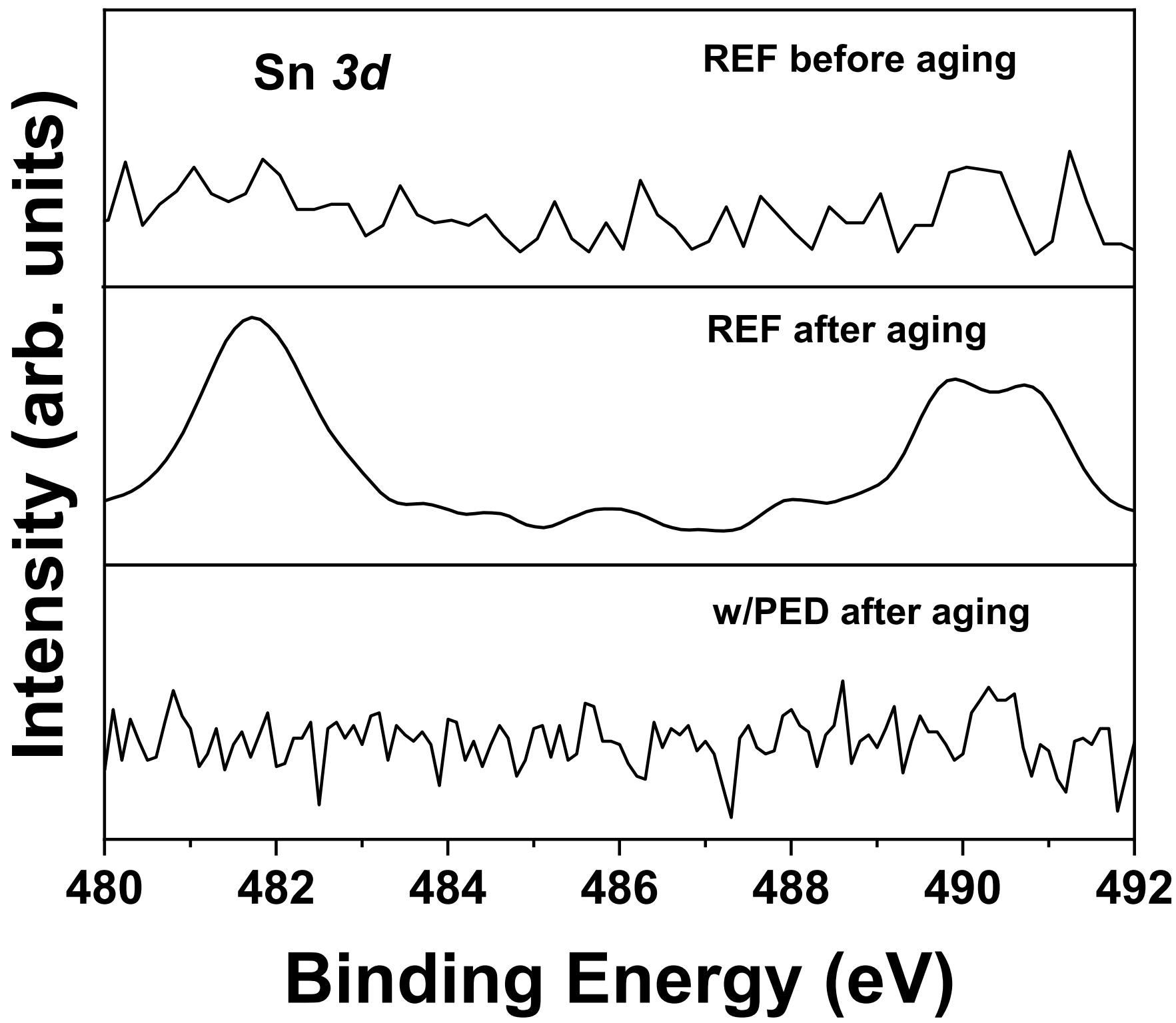

Supplementary Fig. 26 | XPS spectra for Sn *3d* of the buried interface before aging and after aging

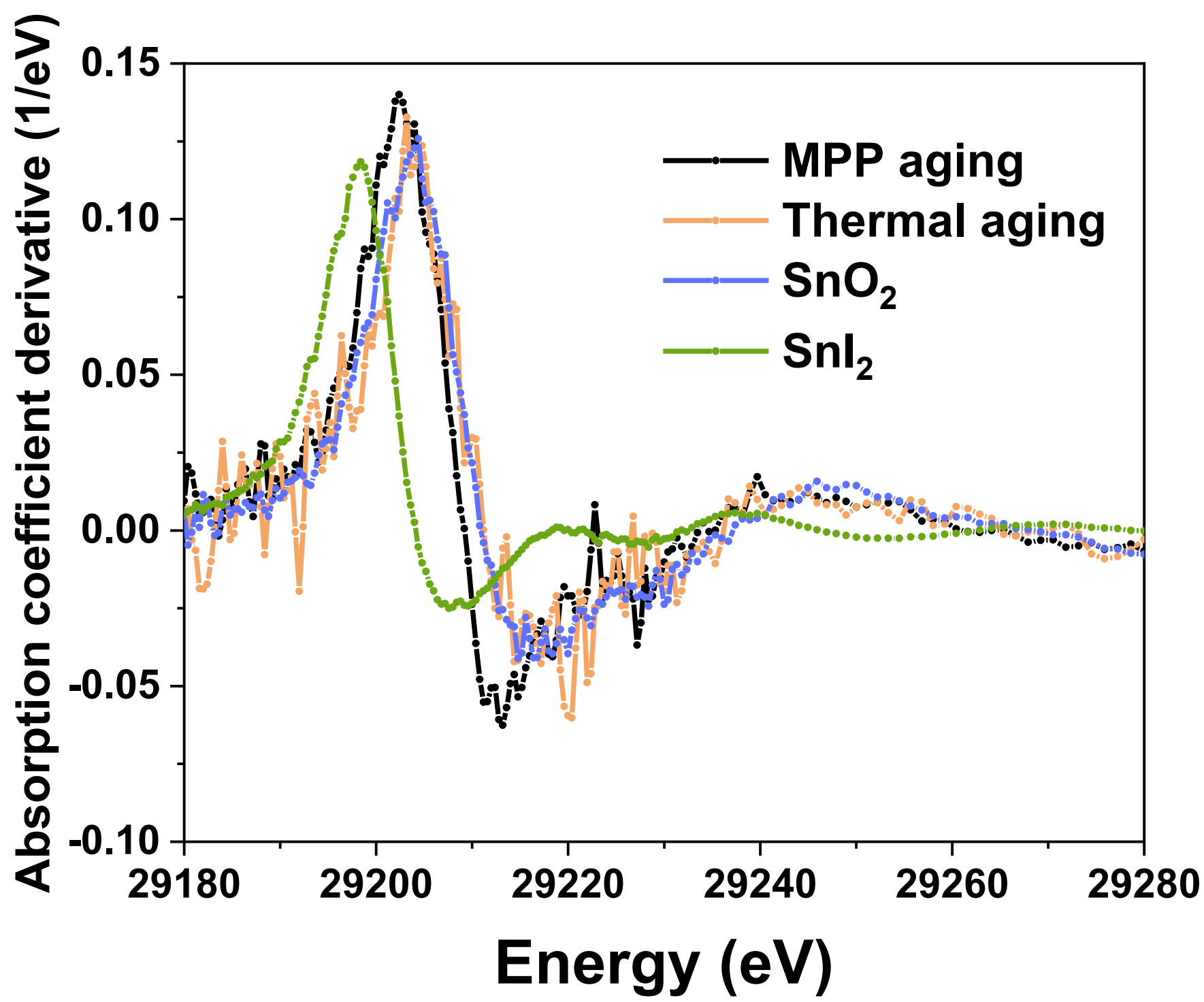

Supplementary Fig. 27 | Derivative of X-ray absorption coefficient in the Sn K-edge region for the MPP and thermal aging samples

SnO_2 and SnI_2 samples are used as references.

TÜVNORD

TÜV NORD (Hangzhou) Co., Ltd.
Room B409, Building 1, No.9, Jiuhuan Road, Shangcheng, District, Hangzhou City, Zhejiang Province, 310019, P.R. China
Phone: +86 (0) 571 8538 6989
Fax: +86 (0) 571 8538 6986
hzpcert@tuv-nord.com
www.tuv-nord.com/cn

Test Report
No. TRPVP01062/26P/01

Commission Testing
according to IEC 61215-2 / EN IEC 61215-2

Applicant: **Lanzhou University**
No. 222 Tianshui South Road, Lanzhou, Gansu Province, P. R. China, 730000

File No.: PVP01062/26P-01

Designed: (Project Engineer) by: Bella Liu (digital signature: 2026.01.23 12:35:20 +08'00')

Reviewed: (Technical Certifier) by: Henry Hang (digital signature: 2026.01.23 13:23:38 +08'00')

All copyright and joint copyrights with respect to studies, assessments, test results, calculations, presentations, etc., drafted by TÜV NORD (Hangzhou) Co., Ltd. shall remain the property of TÜV NORD (Hangzhou) Co., Ltd. TÜV NORD (Hangzhou)'s contractual partner may use assessments, studies, test results, calculations, presentations, etc., drafted within the scope of the contract only for the purpose agreed in the contract or agreement. It is not permissible to pass on to third parties the reports, assessments, test results, calculations, presentations, etc., drawn up by TÜV NORD (Hangzhou) Co., Ltd. or to publish them in abridged form, unless the parties to the contract have concluded a written agreement on the passing on, presentation or publication of extracts from them.

TÜVNORD

Test Report

File No.: PVP01062/26P-01 Test Report No.: TRPVP01062/26P/01

IEC 61215-2 / EN IEC 61215-2			
Clause	Requirement + Test	Result - Remark	Verdict

Test results of IEC 61215-2 / EN IEC 61215-2
Perovskite solar cell

**4.2 Maximum power determination - MQT 02**								—
Test date [YYYY-MM-DD] ......:	2026-01-20							—
Test method ......:	☒ Solar simulator / ☐ Natural sunlight							—
Module temperature [°C] ......:	25±5							—
Irradiance [W/m²] ......:	1000							—
Sample #	I_{sc} [mA]	V_{oc} [V]	I_{mp} [mA]	V_{mp} [V]	P_{max} [mW]	FF [%]	η [%]	
1-forward	1.598	1.192	1.490	1.060	1.58	82.94	25.64	
1-reverse	1.593	1.194	1.545	1.060	1.64	86.06	26.58	

Supplementary information:
The tests are performed according to client's application.
During this maximum power determination, the spectral mismatch was not considered. And the lab did not perform light soaking procedure prior to measurements.
η [%] = Pmax [W] / area [m²] / 1000 [W/m²] x 100% (designated illuminated area: 0.0616 cm²).
The measurements were performed with a steady state solar simulator class AAA according to IEC 60904-9:2020.
Sample#-forward: The electrical resistance of solar simulator was scanned from 0 to +∞.
Sample#-reverse: The electrical resistance of solar simulator was scanned from +∞ to 0.
And the IV measurement characteristic was listed as below:

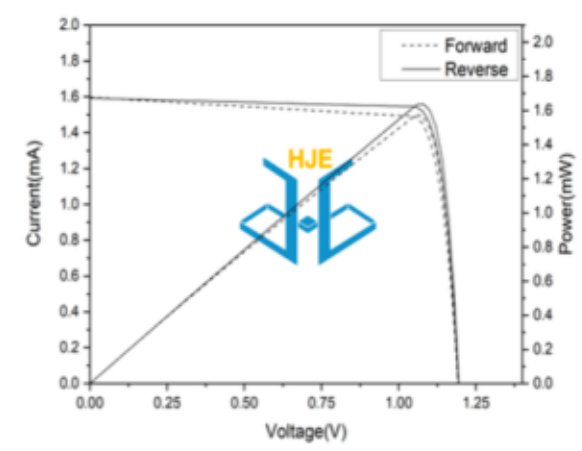

Supplementary Fig. 28 | Independent efficiency certification of PSC small-area device

TÜVNORD

TÜV NORD (Hangzhou) Co., Ltd.
Room B409, Building 1, No.9, Jiuhuan Road, Shangcheng, District, Hangzhou City, Zhejiang Province, 310019, P.R. China
Phone: +86 (0) 571 8538 6989
Fax: +86 (0) 571 8538 6986
hzpcert@tuv-nord.com
www.tuv-nord.com/cn

Test Report
No. TRPVP01062/26P/07

Commission Testing
according to IEC 61215-2 / EN IEC 61215-2

Applicant: **Lanzhou University**
No. 222 Tianshui South Road, Lanzhou, Gansu Province, P. R. China, 730000

File No.: PVP01062/26P-07

Designed: (Project Engineer) by: 数字签名者: bella liu 日期: 2026.01.23 12:39:18 +08'00'

Reviewed: (Technical Certifier) by: 数字签名者: Henry Huang 日期: 2026.01.23 13:25:10 +08'00'

All copyright and joint copyrights with respect to studies, assessments, test results, calculations, presentations, etc., drafted by TÜV NORD (Hangzhou) Co., Ltd. shall remain the property of TÜV NORD (Hangzhou) Co., Ltd. TÜV NORD (Hangzhou)'s contractual partner may use assessments, studies, test results, calculations, presentations, etc., drafted within the scope of the contract only for the purpose agreed in the contract or agreement. It is not permissible to pass on to third parties the reports, assessments, test results, calculations, presentations, etc., drawn up by TÜV NORD (Hangzhou) Co., Ltd. or to publish them in abridged form, unless the parties to the contract have concluded a written agreement on the passing on, presentation or publication of extracts from them.

TÜVNORD

Test Report

File No.: PVP01062/26P-07 Test Report No.: TRPVP01062/26P/07

IEC 61215-2 / EN IEC 61215-2			
Clause	Requirement + Test	Result - Remark	Verdict

Test results of IEC 61215-2 / EN IEC 61215-2
Perovskite solar module

4.2 Maximum power determination - MQT 02							—
Test date [YYYY-MM-DD] ......:			2026-01-20				—
Test method ......:			☒ Solar simulator / ☐ Natural sunlight				—
Module temperature [°C] ......:			25±5				—
Irradiance [W/m²] ......:			1000				—
Sample #	I_{sc} [mA]	V_{oc} [V]	I_{mp} [mA]	V_{mp} [V]	P_{max} [mW]	FF [%]	η [%]
1-forward	59.334	10.692	56.897	9.050	514.91	81.16	23.95
1-reverse	59.310	10.771	56.803	9.150	519.75	81.36	24.17

Supplementary information:
The tests are performed according to client's application.
During this maximum power determination, the spectral mismatch was not considered. And the lab did not perform light soaking procedure prior to measurements.
η [%] = Pmax [W] / area [m²] / 1000 [W/m²] x 100% (designated illuminated area: 21.5 cm²).
The measurements were performed with a steady state solar simulator class AAA according to IEC 60904-9:2020.
Sample#-forward: The electrical resistance of solar simulator was scanned from 0 to +∞.
Sample#-reverse: The electrical resistance of solar simulator was scanned from +∞ to 0.
And the IV measurement characteristic was listed as below:

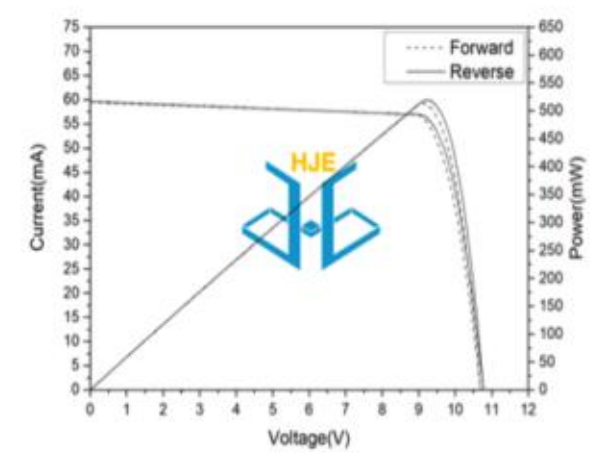

Supplementary Fig. 29 | Independent efficiency certification of PSC Module

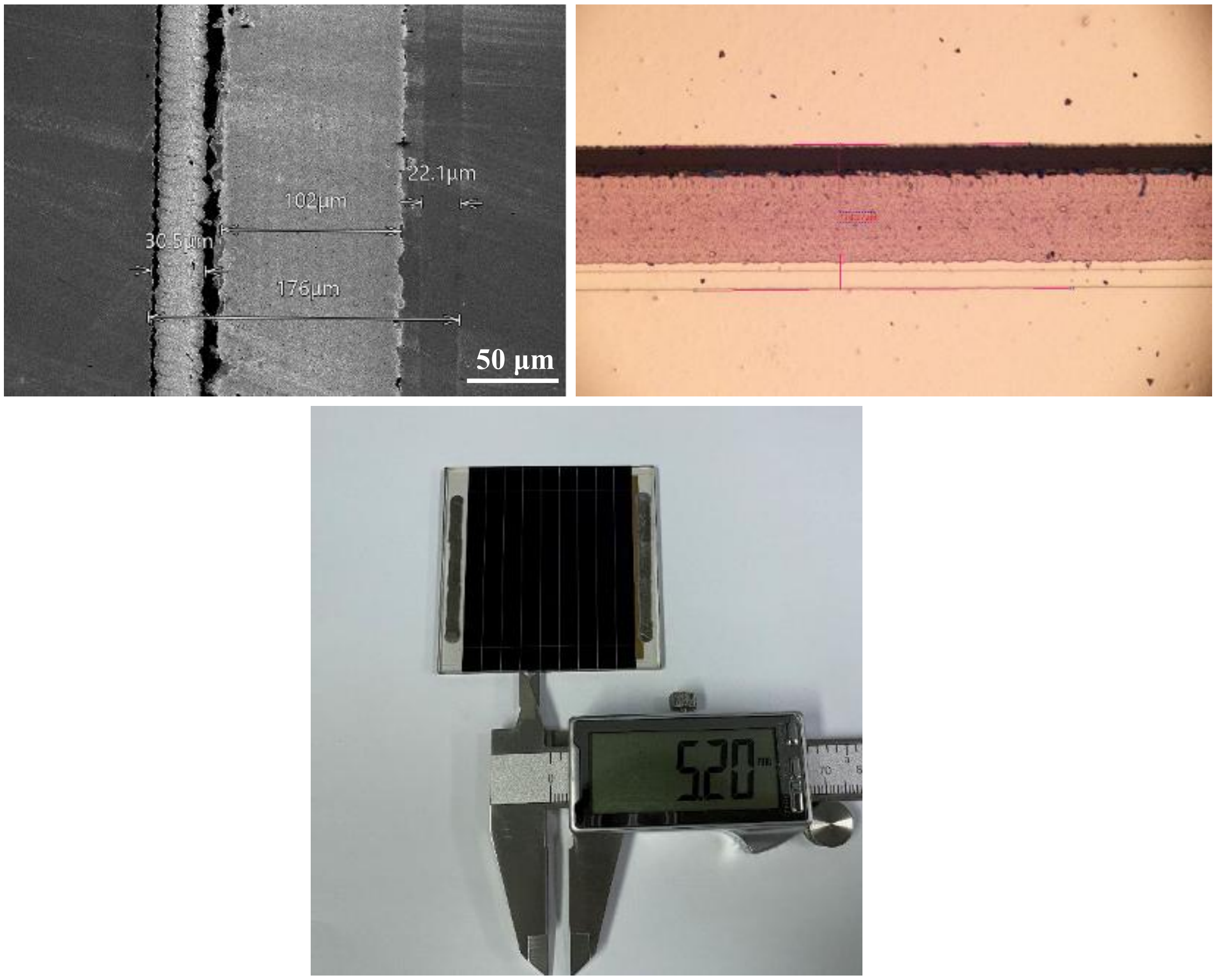

Supplementary Fig. 30 | Experimental verification of the module GFF and scribing quality

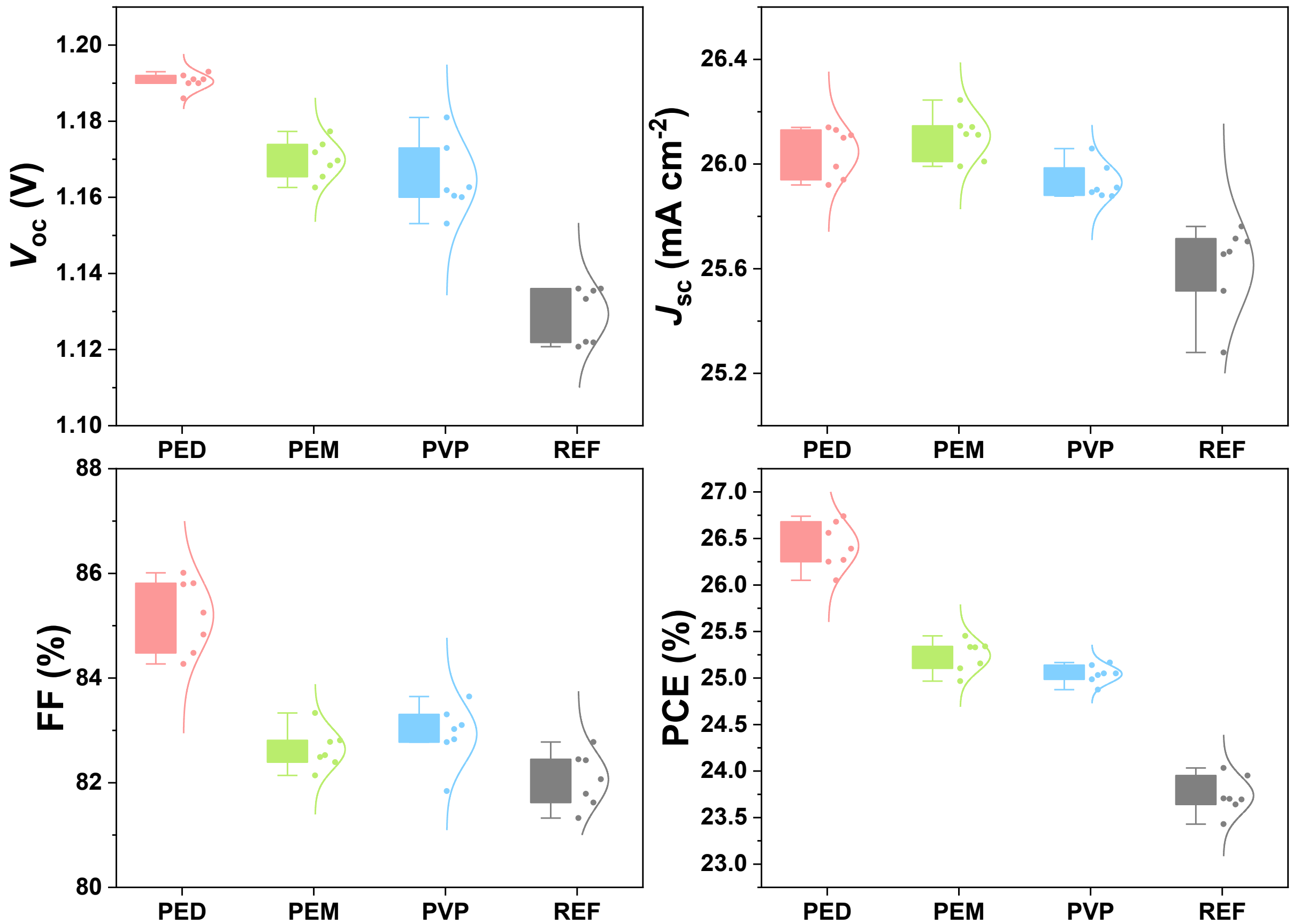

Supplementary Fig. 31 | Statistical photovoltaic parameters of devices with different polymeric interlayers and references

Results are extracted from 7 devices. Box plots show the median (center line), 25th–75th percentiles (box limits), and the 1.5× the interquartile range (whiskers). Individual data points are shown as diamonds, and the dashed curves represent the fitted normal distributions.

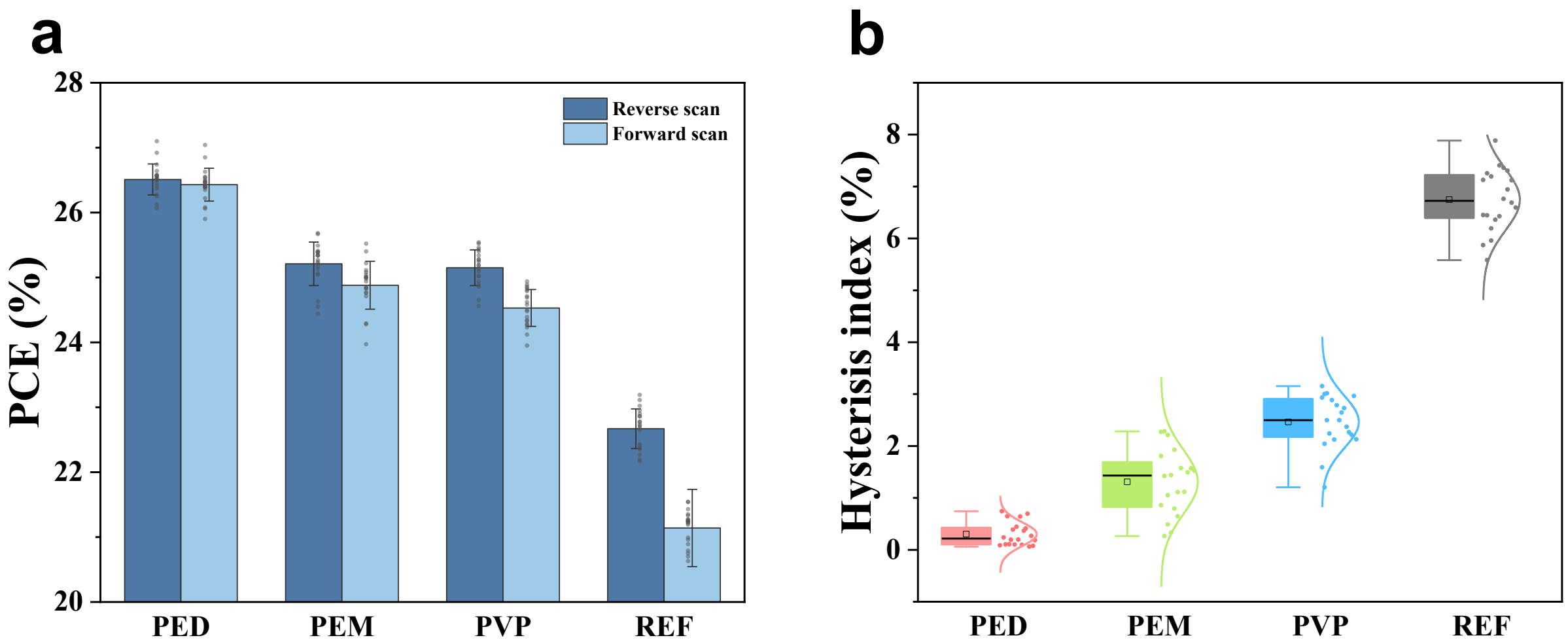

Supplementary Fig. 32 | Effects of polymer interlayers on device performance and hysteresis

a, Statistical comparison of reverse- and forward-scan PCEs for PED-, PEM-, PVP-modified and reference devices. Bars represent mean values, error bars represent standard deviation, and overlaid points represent individual devices (n = 20). **b,** Distribution of hysteresis index (HI) calculated from the same devices using $\mathbf{HI = (PCE_{reverse} - PCE_{forward})/PCE_{reverse} \times 100\%}$. Box-plots show the median and interquartile range, and overlaid points represent individual devices. Box plots show the median (center line), 25th and 75th percentiles (box limits), and the most extreme data points within 1.5× the interquartile range (whiskers). Squares represent the mean, individual data points are shown as dots, and the curves represent the fitted normal distributions.

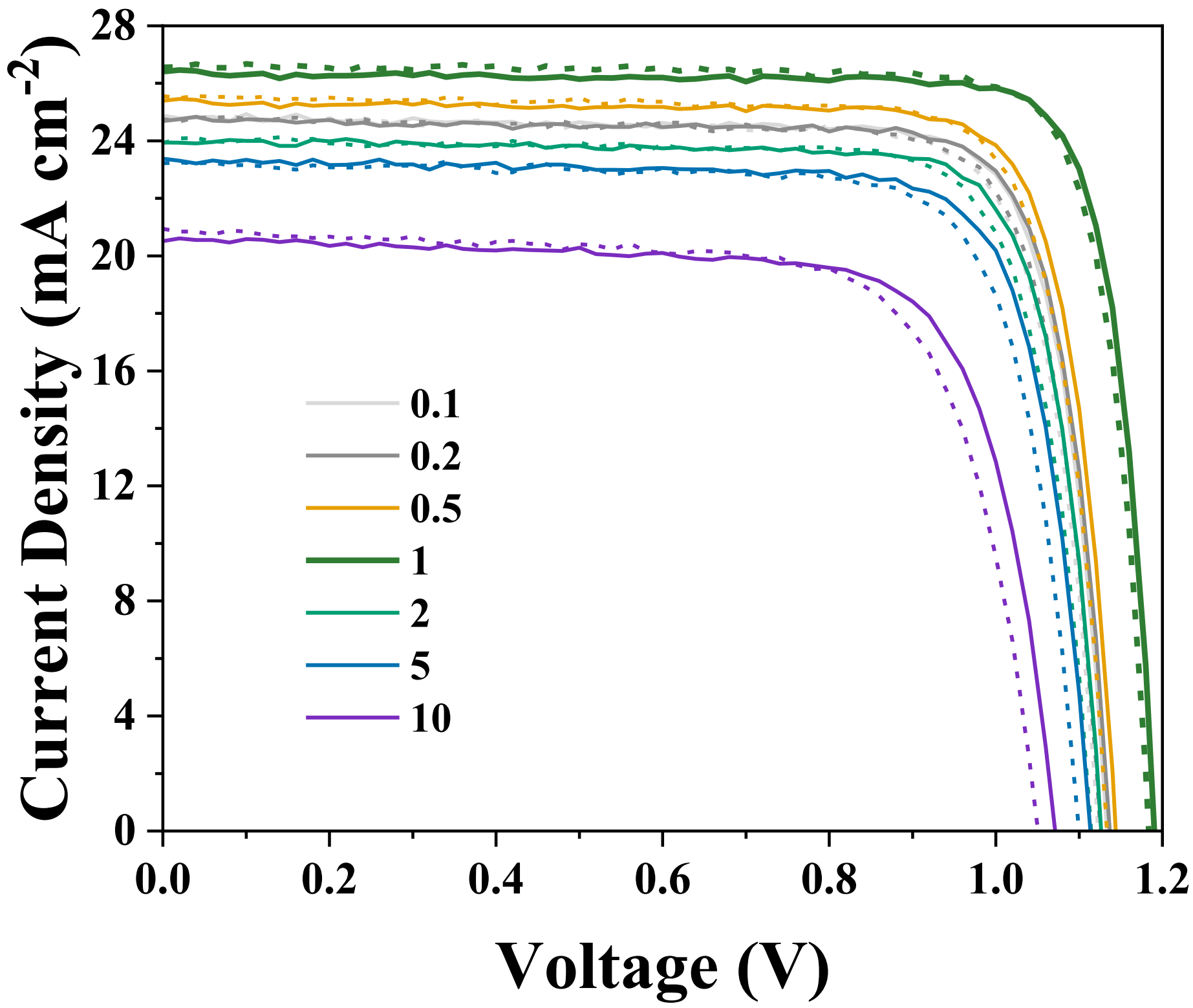

Supplementary Fig. 33 | Concentration-dependent device performance of PED-modified devices The unit of concentration is mg ml^{-1}.

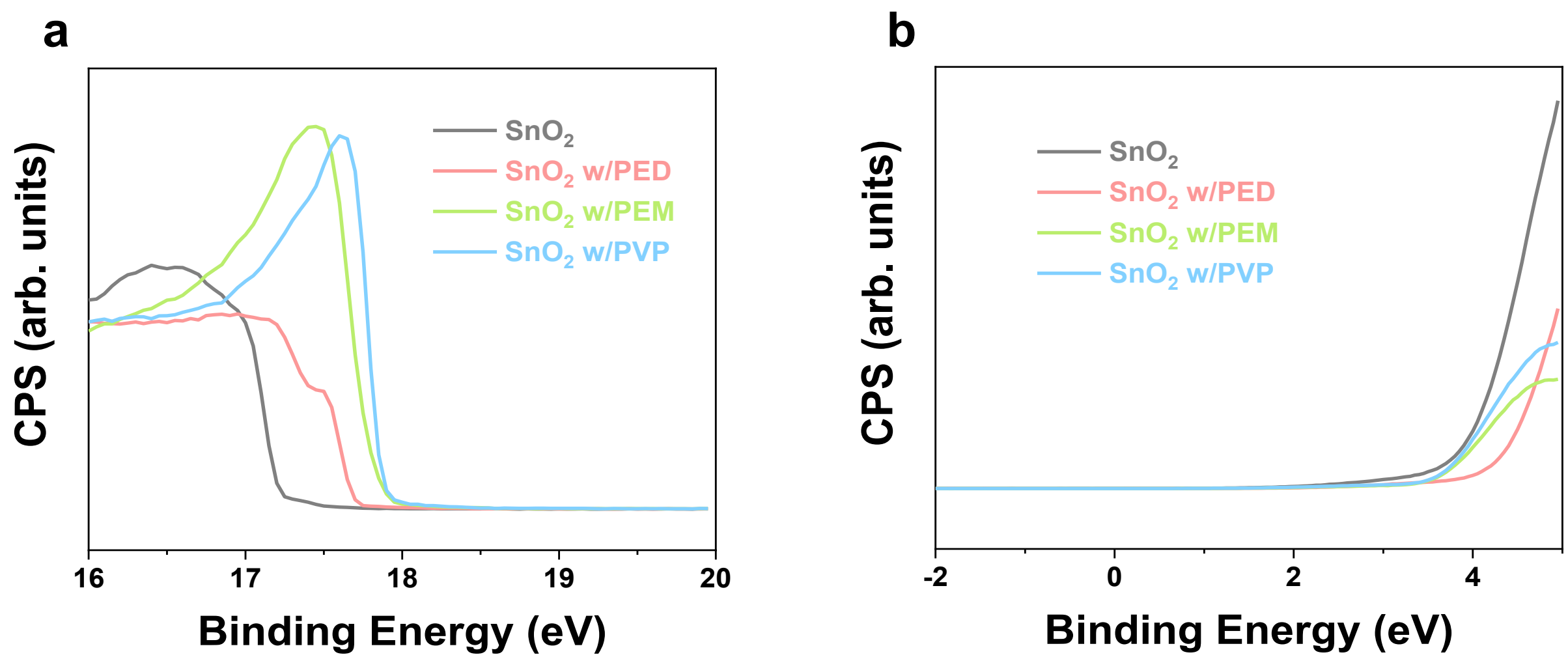

Supplementary Fig 34 | UPS spectra of SnO_2 and polymer-modified SnO_2 buried interfaces

a, Valence-band onset region near the Fermi level; b, secondary electron cutoff region.

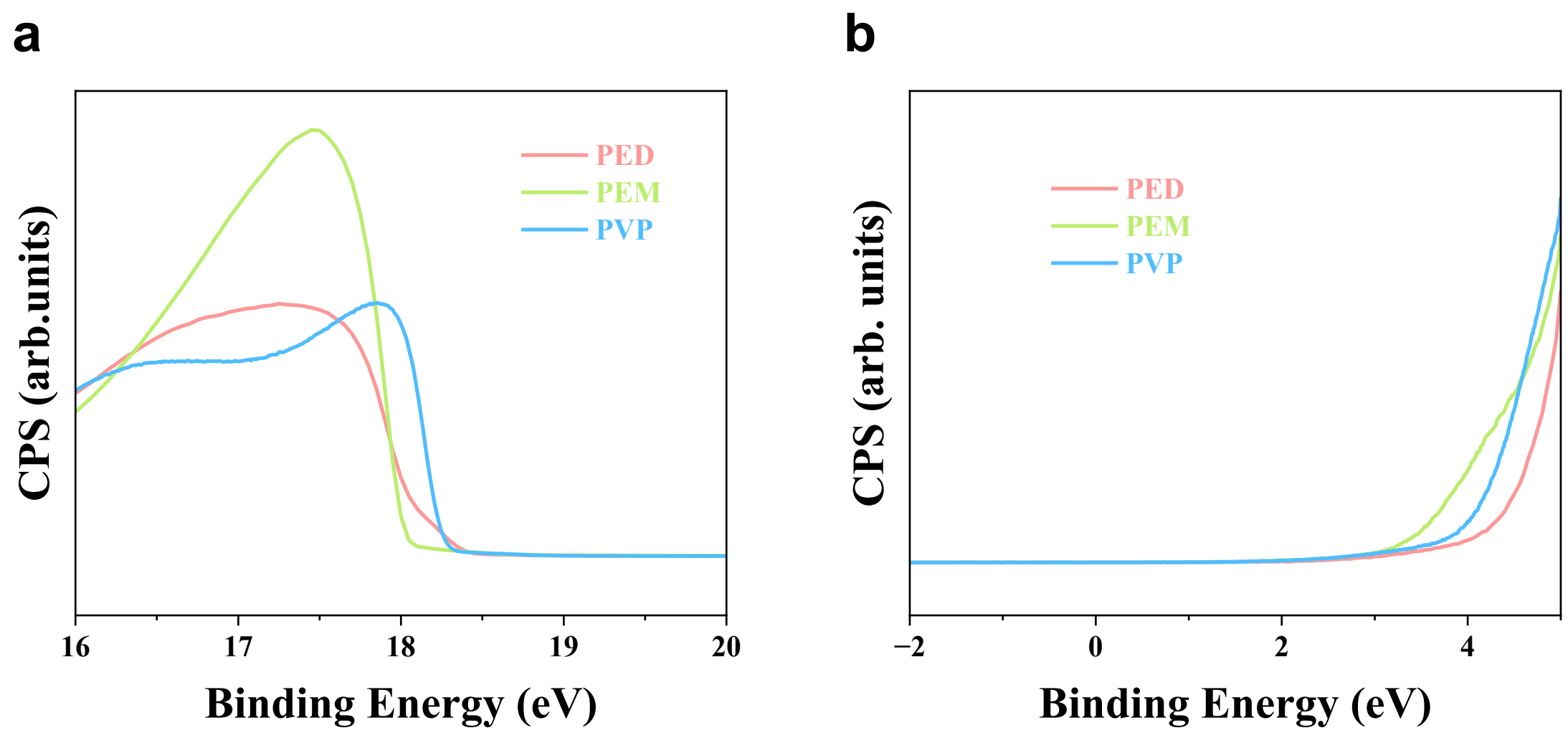

Supplementary Fig. 35 | UPS spectra of polymer only films

a, HOMO onset region near the Fermi level; b, secondary electron cutoff region.

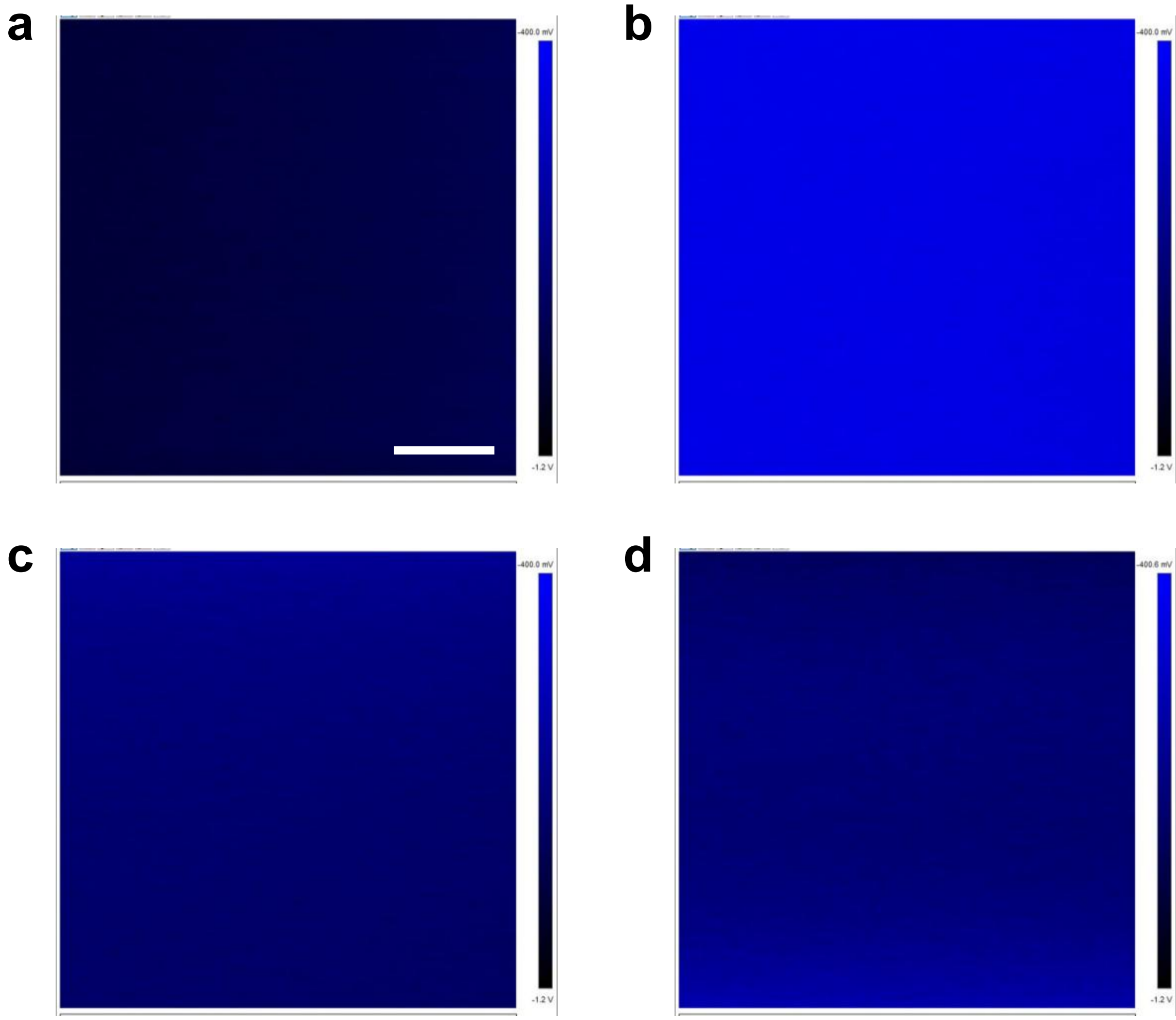

Supplementary Fig. 36 | KPFM for the buried interface work function

a, ITO/SnO_2; **b,** ITO/SnO_2/PED; **c,** ITO/SnO_2/PEM; **d,** ITO/SnO_2/PVP (scale bar, 1 μm).

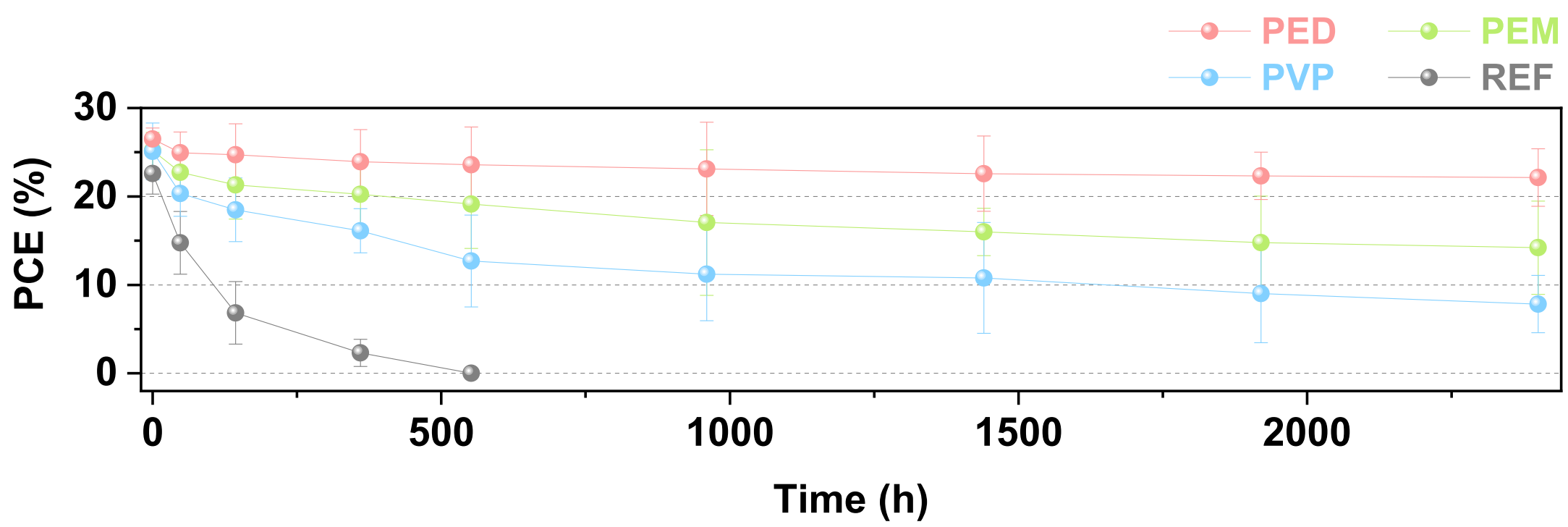

Supplementary Fig. 37 | 65 ℃ thermal-light stability test under one-sun illumination without or with the polymeric interlayers with spiro-OMeTAD as HTL

For each condition of the stability tests, ten devices were measured for statistical analysis. Centre is the median value. Error bars indicate the standard deviation from these samples.

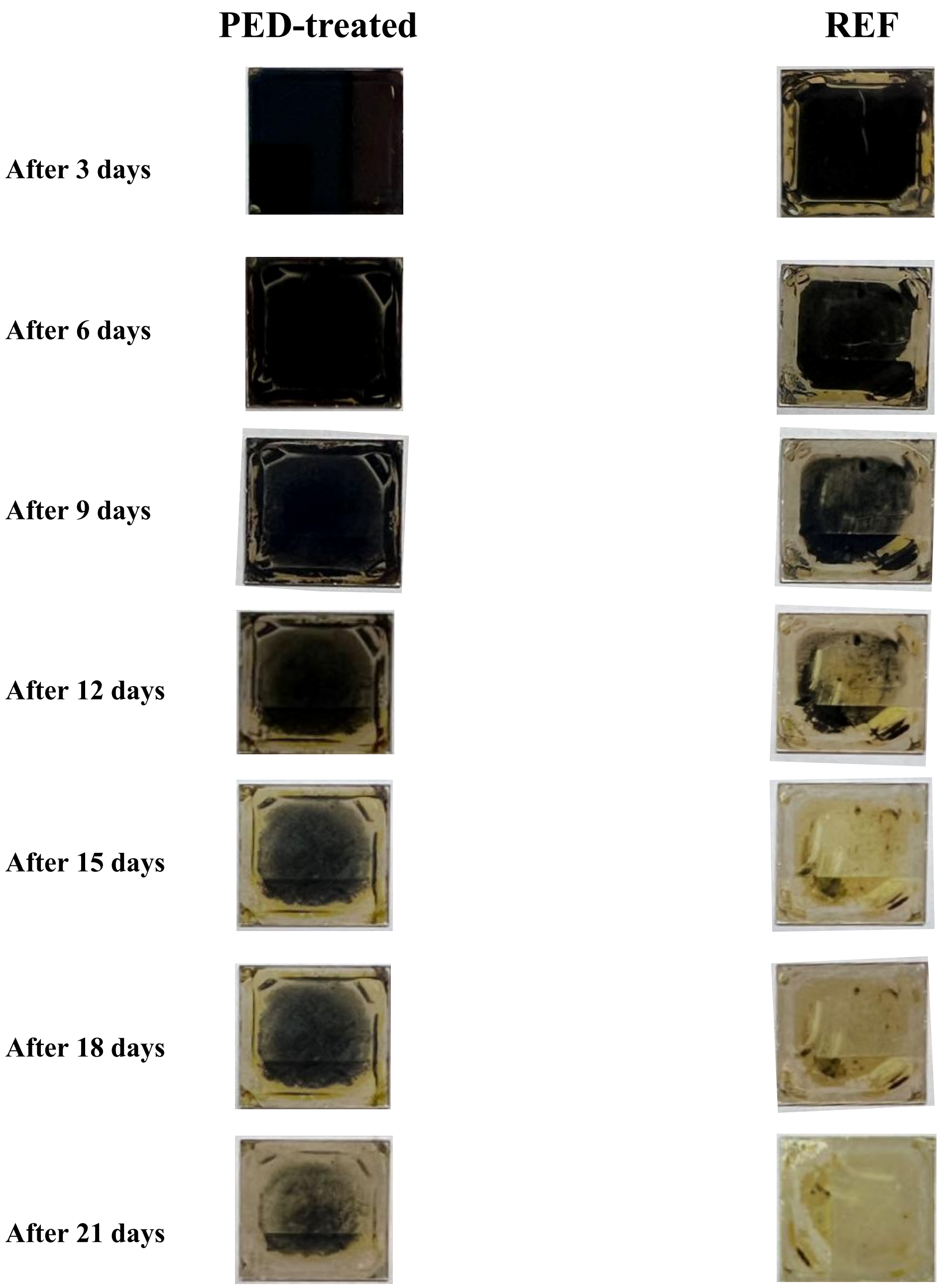

Supplementary Fig. 38 | Perovskite film under 40% RH with light halogen bulb illumination at 40°C

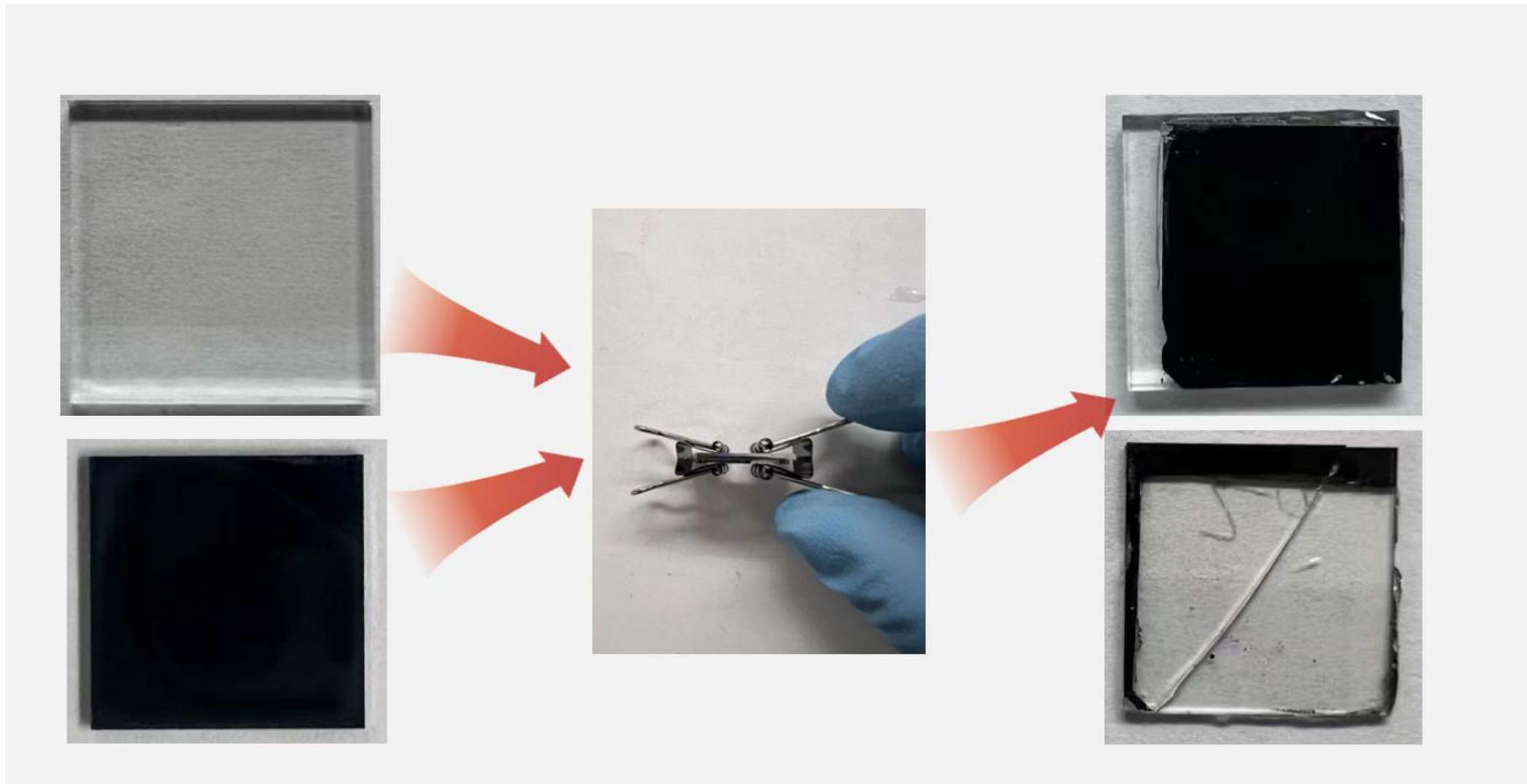

Supplementary Fig. 39 | Illustration of the Perovskite film peeling-off process

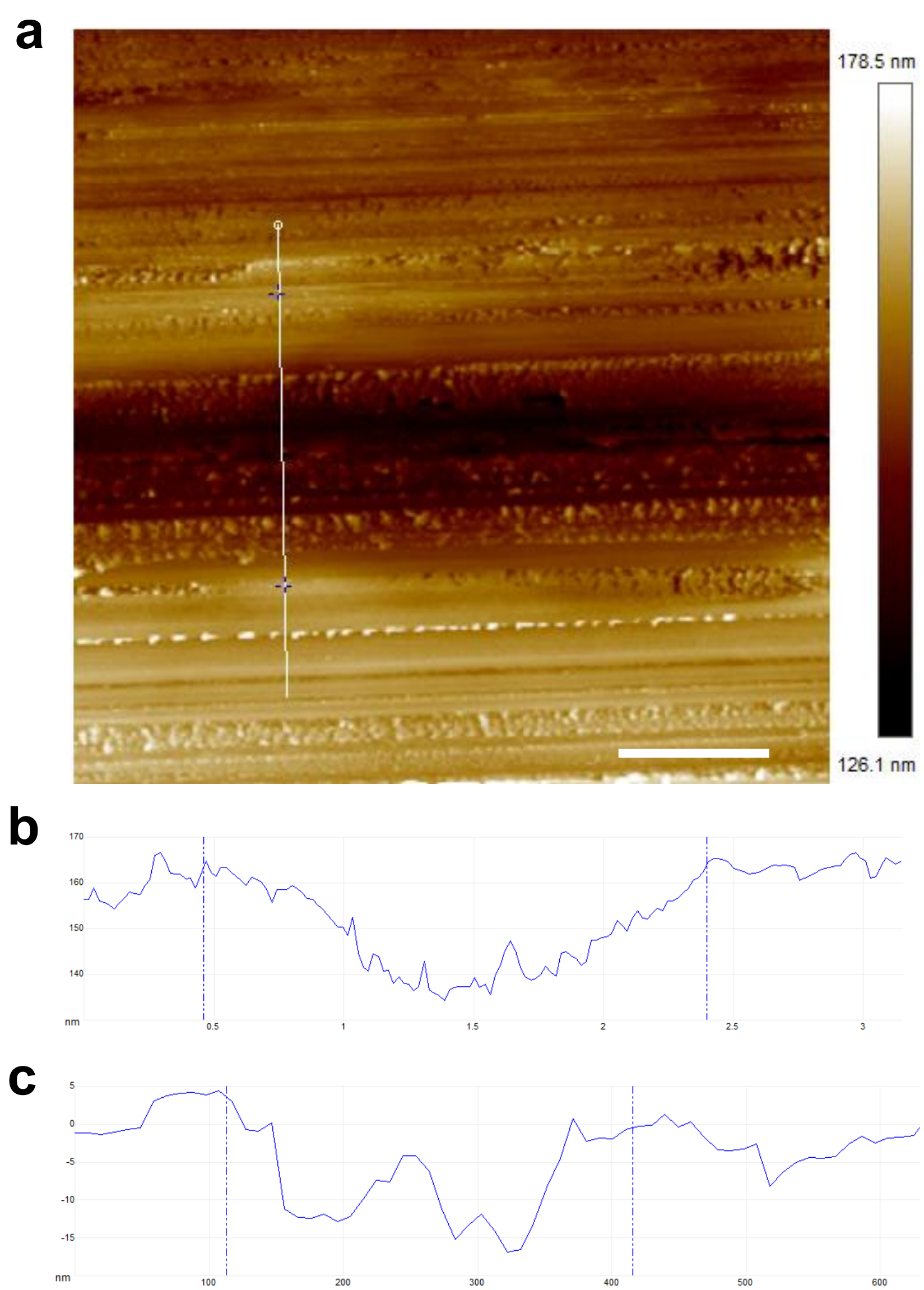

Supplementary Fig. 40 | Representative AFM scratch step-height analysis of PED-modified SnO_2

a, Representative AFM height image of a mechanically scratched PED-modified SnO_2 film prepared at 3000 rpm. The white line indicates the position used for line-profile analysis (scale bar, 1μm). **b** and **c**, Representative AFM line profiles extracted from PED-modified SnO_2 and pristine SnO_2 control samples. Because mechanical scratching can partially remove the underlying SnO_2 layer together with the polymer overlayer, the measured values are interpreted as effective scratch step heights rather than exact absolute polymer thicknesses.

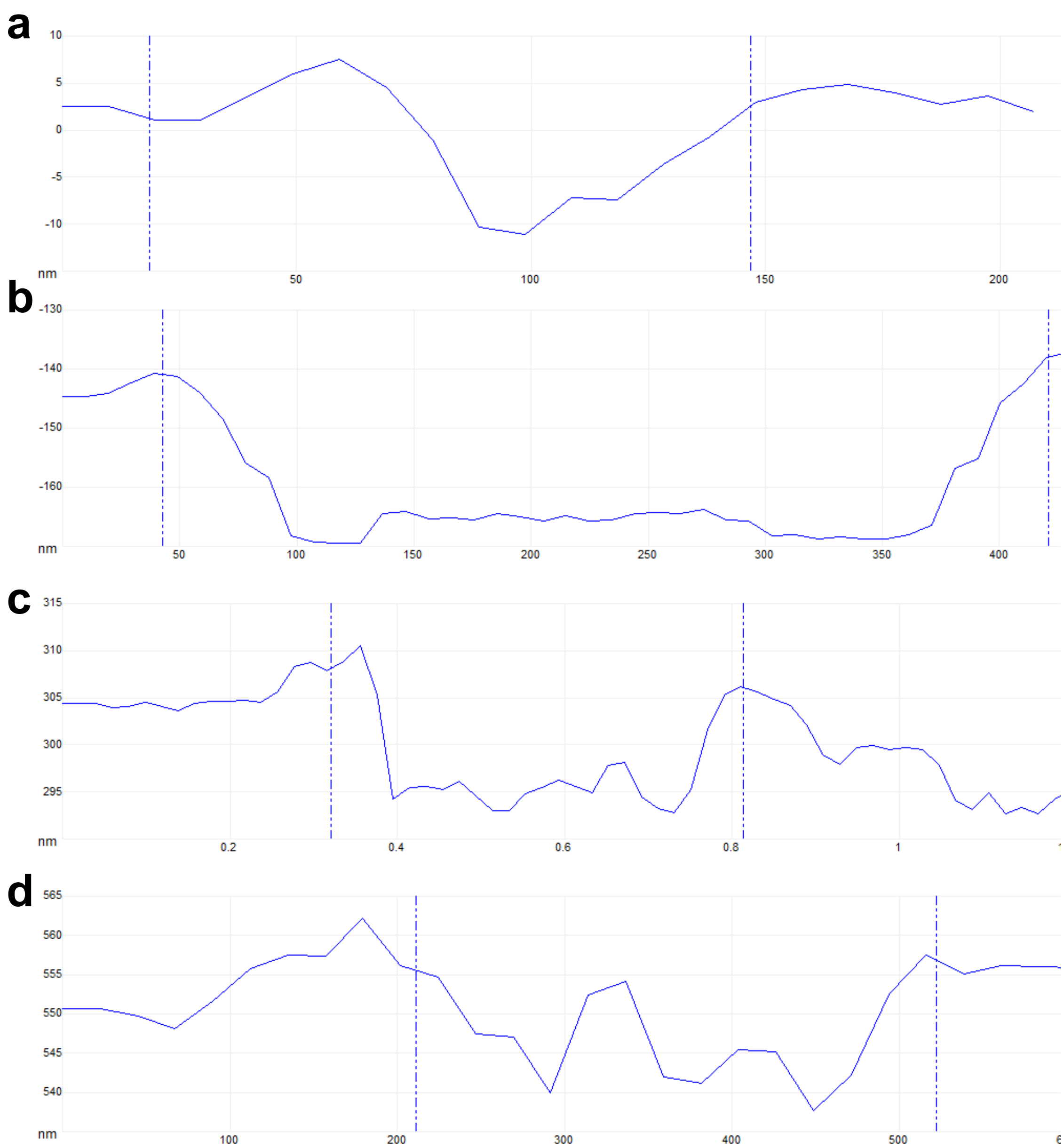

Supplementary Fig. 41 | AFM line-profile comparison of polymer-modified SnO_2 films prepared under different conditions

a, PEM at 3000 rpm; **b,** PVP at 3000 rpm; **c,** PED at 500 rpm; **d,** PED at 5000 rpm.

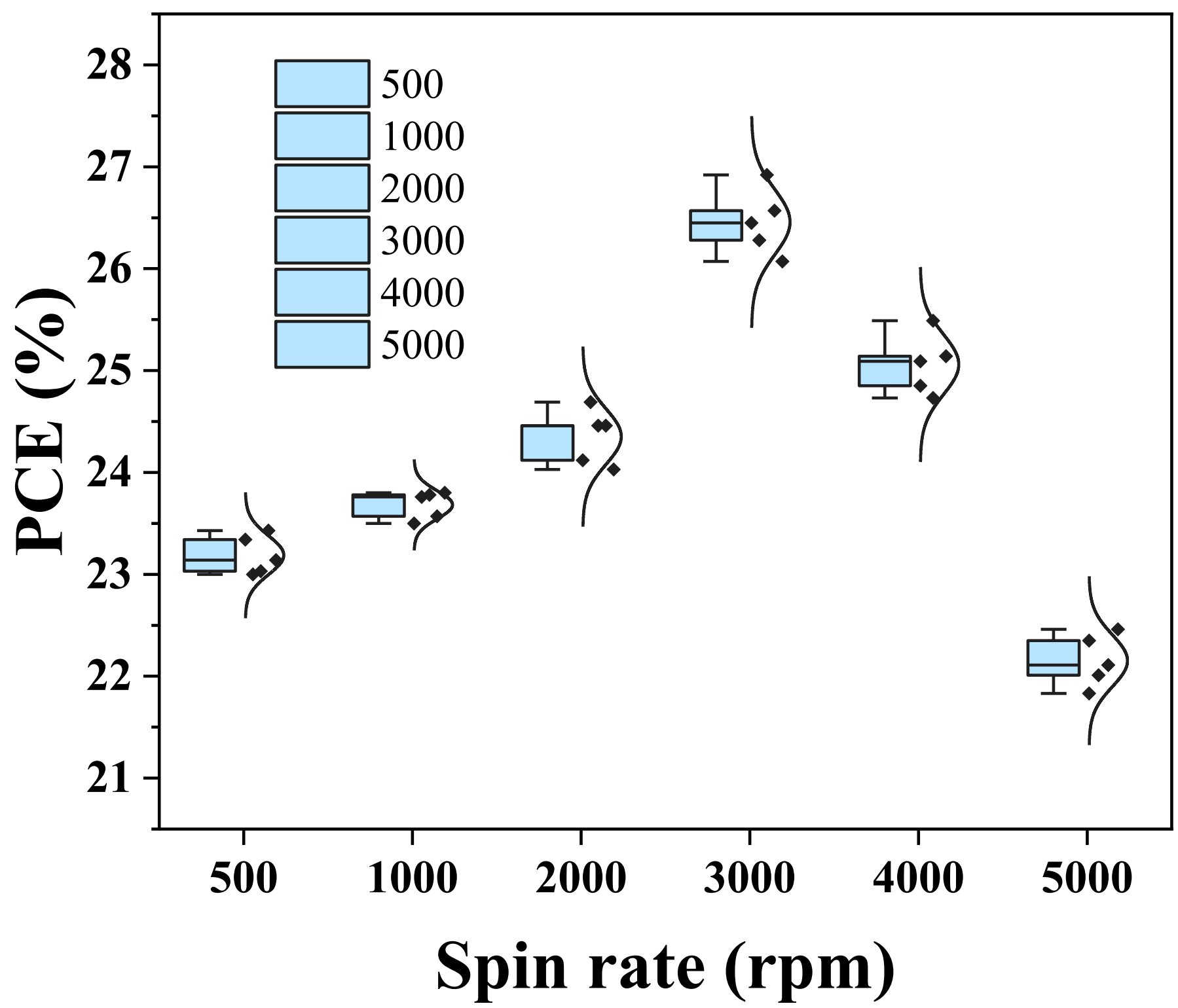

Supplementary Fig. 42 | Spin-speed-dependent device-performance screening of PED-modified devices

Results are extracted from 5 devices. Box plots show the median (center line), 25th and 75th percentiles (box limits), and the most extreme data points within 1.5× the interquartile range (whiskers). Diamonds represent individual data points, and the curves represent the fitted normal distributions.

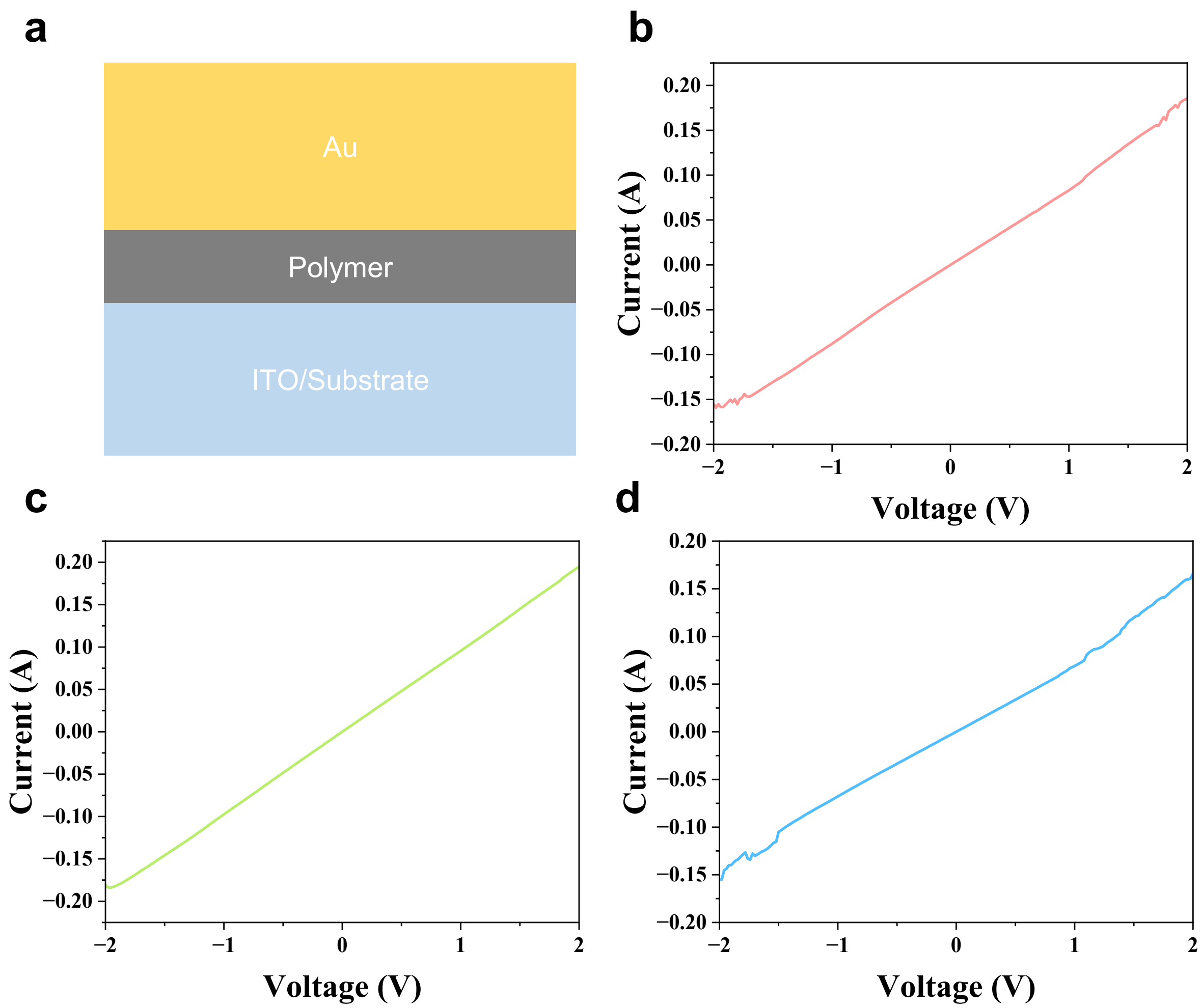

Supplementary Fig. 43 | Vertical electrical characterization of ultrathin polymer layers

a, Schematic illustration of the two-terminal vertical device structure used for electrical characterization of polymer films. **b - d**, Vertical current–voltage characteristics of **(b)** PED, **(c)** PEM, and **(d)** PVP films prepared under the optimized processing conditions.

Supplementary Tables

Supplementary Table 1 | Fresh sample XRD peak ratio of PbI_2 and α-$FaPbI_3$

Condition	Intensity of PbI_2 (arb. units)	Intensity of (001) α-$FAPbI_3$ (arb. units)	Ratio
REF	4865	4241	0.87
PED	1918	6091	3.18
PEM	4488	5372	1.20
PVP	4082	4917	1.20

Supplementary Table 2 | Aged sample XRD peak ratio of PbI_2 and α-$FaPbI_3$

Condition	Intensity of PbI_2 (arb. units)	Intensity of (001) α-$FAPbI_3$ (arb. units)	Ratio
REF	10533	3490	0.33
PED	2224	4312	1.94
PEM	2027	1739	0.86
PVP	3118	3155	1.01

Supplementary Table 3 | Data of the ICP-AES graph

Sample	Device condition	1st test atomic percentage(%)	2nd test atomic percentage(%)
REF	Fresh	0.82999	0.83874
	Aged	2.00494	2.05388
PED	Fresh	0.33986	0.34752
	Aged	0.39092	0.43194

Supplementary Table 4 | XPS survey spectrum for aged reference sample

Name	Pos.	FWHM	Area	At%
N *1s*	399.91	2.16	1473.86	4.92
C *1s*	284.91	3.19	9729.99	58.5
O *1s*	532.91	2.83	6427.29	13.19
Sn *3d*	485.91	2.54	1041.87	0.25
Pb *4f*	137.91	2.22	42939.04	11.35
I *3d*	617.91	2.41	53716.62	9.6
Br *3p*	185.91	3.03	1824.88	2.18

Supplementary Table 5 | Photovoltaic parameters of champions device for different conditions

Condition	V_{oc} (V)	J_{sc} (mA/cm^2)	FF (%)	Eff (%)
REF R	1.136	25.20	80.95	23.17
REF F	1.136	25.28	81.62	23.43
PED R	1.191	26.10	86.01	26.74
PED F	1.187	26.19	84.36	26.19
PEM R	1.184	25.89	81.95	25.12
PEM F	1.168	25.74	83.92	25.23
PVP R	1.163	25.80	83.65	25.12
PVP F	1.181	25.91	81.84	25.05

Supplementary Table 6 | Statistical summary of forward/reverse *J–V* measurements and hysteresis indices

Condition	Reverse PCE (%)	Forward PCE (%)	Hysteresis Index(%)
PED	26.51±0.24	26.43±0.25	0.302±0.228
PEM	25.21±0.34	24.88±0.37	1.309±0.622
PVP	25.15±0.27	24.53±0.28	2.465±0.505
REF	22.67±0.31	21.14±0.59	6.747±0.594

Supplementary Table 7 | Photovoltaic parameters of representative PED-modified devices prepared with different PED concentrations

Condition	V_{oc} (V)	J_{sc} (mA/cm^2)	FF (%)	Eff (%)
0.1 mg/ml F	1.123	24.82	80.52	22.44
0.1 mg/ml R	1.134	24.88	81.04	22.85
0.2 mg/ml F	1.125	24.75	81.24	22.63
0.2 mg/ml R	1.137	24.69	81.77	22.95
0.5 mg/ml F	1.133	25.56	81.49	23.61
0.5 mg/ml R	1.144	25.40	82.09	23.85
1 mg/ml F	1.183	26.56	84.07	26.42
1 mg/ml R	1.190	26.41	84.14	26.45
2 mg/ml F	1.114	23.92	80.77	21.53
2 mg/ml R	1.126	23.95	81.61	22.01
5 mg/ml F	1.100	23.22	78.76	20.11
5 mg/ml R	1.113	23.38	79.31	20.65
10 mg/ml F	1.051	20.94	72.73	16.01
10 mg/ml R	1.071	20.51	75.42	16.57

Supplementary Table 8 | Calculated effective valence/HOMO levels of SnO_2 and SnO_2/polymer stacks from UPS results

Condition	SnO_2	SnO_2 w/PED	SnO_2 w/PEM	SnO_2 w/PVP
Cut-off Energy (eV)	17.18	17.65	17.82	17.89
Work function Φ (eV)	4.03	3.56	3.39	3.32
Valence/HOMO Onset (eV)	3.92	4.38	3.61	3.67
Calculated effective valence/HOMO level vs vacuum (eV)	-7.95	-7.94	-7.00	-6.99

Supplementary Table 9 | Calculated HOMO for polymer from UPS results

Condition	PED	PEM	PVP
Cut-off Energy (eV)	18.13	18.02	18.26
Work function Φ (eV)	3.08	3.19	2.95
HOMO Onset (eV)	4.29	3.94	3.27
Calculated HOMO level vs vacuum (eV)	-7.37	-7.13	-6.22

Supplementary Table 10 | Apparent vertical conductivity of ultrathin polymer layers

Condition	dJ/dV (A cm^{-2} V^{-1})	R_A (Ω cm²)	σ (S cm^{-1})
PED	0.555	1.80	2.78×10^{-7}
PEM	0.643	1.55	3.22×10^{-7}
PVP	0.447	2.24	2.23×10^{-7}

Supplementary Notes

Supplementary Note 1 | Synthesis and characterization of PED, PEM

The detailed synthetic routes and characterizations of PED and PEM are shown in in Supplementary Scheme.

^{1}H NMR spectra were measured on Bruker AdvanceCore 400 MHz NMR spectrometers. Gel permeation chromatography (GPC) were measured on Shimadzu model LG-2030C 3D.

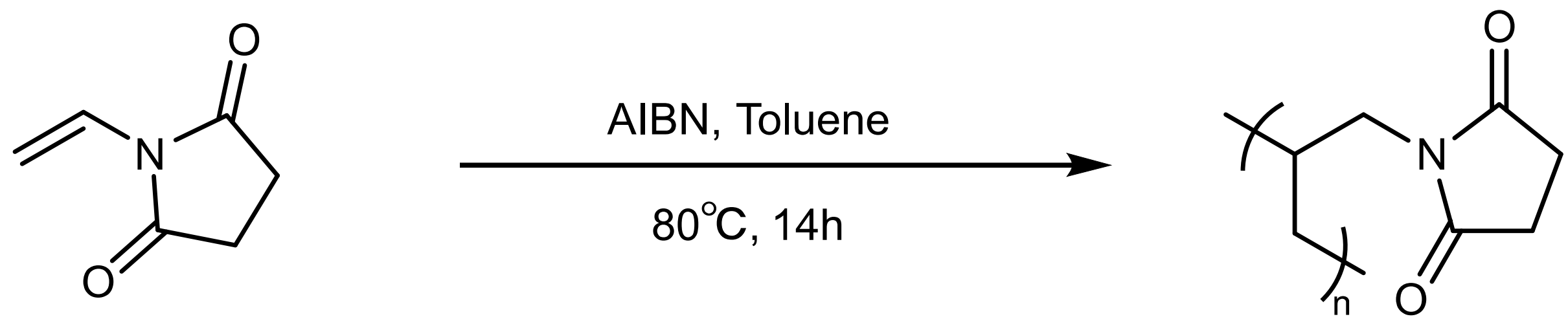

Supplementary Scheme 1 | Synthesis route of PED

Under a nitrogen atmosphere, 1-ethenylpyrrolidine-2,5-dione (ED) (0.2 mL) was dissolved in toluene (1 mL) together with 2,2′-azobis(2-methylpropionitrile) (AIBN, 0.002 g). The solution was heated and stirred at 80 °C for 14 hours to affect free-radical polymerization[1,2]. To improve the solubility of the product, 1mL chloroform (CF) was added to the solution. The mixture was then poured into diethyl ether (DE) to precipitate the polymer. The resulting precipitated polymer was collected by filtration, purified by three cycles of reprecipitation (dissolution in CF followed by precipitation with DE, and dried under vacuum to constant mass. The PED sample was obtained as flake-like solids (M_n=5033, M_w=7465).

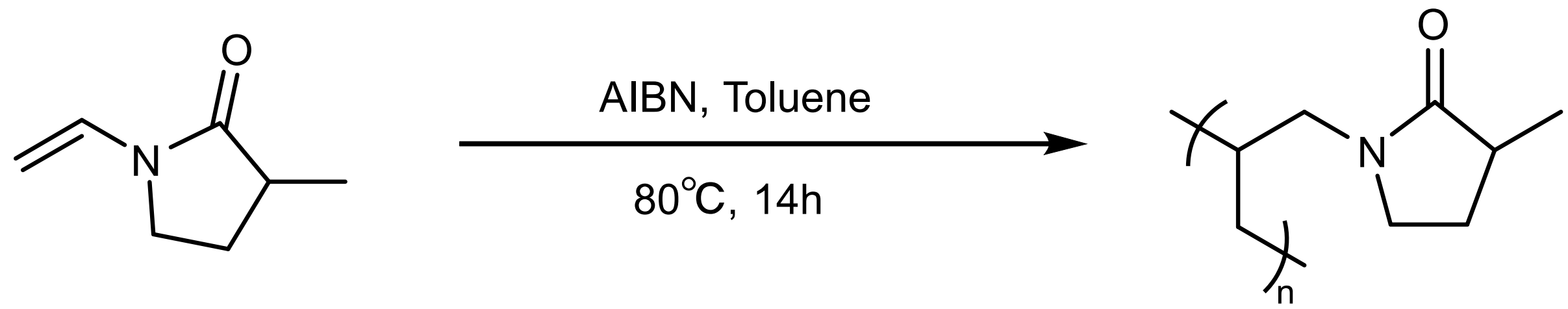

Supplementary Scheme 2 | Synthesis route of PEM

Similarly, under a nitrogen atmosphere, 1-ethenyl-3-methylpyrrolidin-2-one (EM) (0.2 mL) was dissolved was dissolved in toluene (1 mL) together with AIBN (0.002 g). The solution was heated and stirred at 80 °C for 14 hours to effect free-radical polymerization. To improve the solubility of the product, 1mL chlorobenzene (CB) was added to the solution. The mixture was then poured into diethyl ether (DE) to precipitate the polymer. The resulting precipitated polymer was collected by filtration, purified by three cycles of reprecipitation (dissolution in CB followed by precipitation with DE, and dried under vacuum to constant mass. The PEM sample appeared as a free-flowing granular powder (M_w=16304, M_n=62018).

Supplementary Note 2 | Perovskite thin-film peeling process

The perovskite thin-film peeling process was performed as follows (process provided in Supplementary Fig. 39. A uniform layer of A–B epoxy was first applied onto a clean glass substrate (Glass/FTO). The perovskite film was then placed with its top surface in direct contact with the epoxy layer and gently flipped or pressed to ensure complete adhesion. The assembly was left to cure for >8 h under ambient conditions. After curing, the two substrates were carefully separated, leaving the perovskite film adhered to the epoxy-coated glass. This configuration exposes the buried interface of the perovskite layer for subsequent characterization.

For complete device stacks, the top metal electrode was first removed using Kapton tape. The same peeling protocol described above was then applied to expose the buried perovskite/ETL interface.

To ensure a high yield and reproducible results for the mechanical peeling process, robust FTO substrates were employed to withstand the necessary stress without shattering. The success rate for obtaining macroscopically intact and undamaged peeled films is approximately 60%. Furthermore, high-resolution STEM characterization of the exposed buried interface (as shown in Fig.2a) reveals intrinsic grain boundary morphologies and well-preserved crystal facets. The absence of mechanical tearing patterns or stress-induced micro-cracks confirms that the characterized buried interface accurately reflects its original state within the intact device.

Supplementary References

1. Voit, B. Principles of polymerization. By George Odian. Wiley Online Library (2002).
2. Otsu, T., Matsumoto, A., Kubota, T. & Mori, S. Reactivity in radical polymerization of N-substituted maleimides and thermal stability of the resulting polymers. *Polymer Bulletin* **23**, 43–50 (1990).
