# Supplementary material for: Mitigating tin ion migration and reinforcing buried interface via synergistic polymer-modified tin oxide
Source: Nat Commun. 2026 Sep 11;17:10723. doi: 10.1038/s41467-026-77624-8 (PMC13658243; doi:10.1038/s41467-026-77624-8)
Supplement: Supplementary file 2 [file 41467_2026_77624_MOESM2_ESM.pdf]

nature portfolio

Peer Review File

Mitigating tin ion migration and reinforcing buried interface via synergistic polymer-modified tin oxide

Corresponding Author: Professor Yang Yang

This file contains all reviewer reports in order by version, followed by all author rebuttals in order by version.

Version 0:

Reviewer comments:

Reviewer #1

(Remarks to the Author)
Authors reports a strategy to stabilize the buried SnO_2/perovskite interface in n–i–p perovskite solar cells (PSCs) by introducing polymeric interlayers — poly(1-ethenylpyrrolidine-2,5-dione) (PED), poly(1-ethenyl-3-methylpyrrolidin-2-one) (PEM), and polyvinylpyrrolidone (PVP). The authors identify tin-ion migration from the SnO_2 electron transport layer as a key yet previously underappreciated degradation pathway and demonstrate that PED, in particular, forms a robust interfacial barrier that suppresses Sn diffusion, improves perovskite crystallinity, and significantly enhances both efficiency (certified PCE of 26.58%) and superior operational stability. This manuscript is timely and the experimental scope is broad. However, several concerns must be addressed before this work can be considered for publication.

1. In Fig. 1c–f, perovskite films on polymeric interlayers show fewer voids than those on bare SnO_2, despite SnO_2 being more hydrophilic and expected to provide better precursor wetting. The authors should explain this counterintuitive result with supporting evidence such as contact angle measurements and a discussion of how the polymer surface modifies perovskite nucleation kinetics.

2. The authors claim that the buried interface is the primary origin of perovskite degradation, yet the supporting evidence (e.g, optical microscopy (Supp. Fig. 5) and post-aging SEM/XPS) does not conclusively rule out co-contributions from the bulk or top surface. I recommend that the authors provide quantitative, depth-profiling evidence (e.g., depth-profiling XPS or SIMS).

3. In Fig. 3f, the fresh REF device already contains 0.83 at% Sn while the fresh PED device contains only 0.34 at% (a significant difference before any aging). If this reflects Sn incorporation during fabrication being blocked by PED, this is an important finding that deserves dedicated discussion; if it lies within measurement uncertainty, replicate measurements with error bars are required.

4. While the GFF of 96.5% is stated in the manuscript, no experimental verification is provided. I recommend that the authors include SEM images of the P1, P2, and P3 scribed lines to directly confirm the GFF and scribing quality.

5. Since the polymeric interlayer insertion may influence interfacial charge extraction and ion migration dynamics, J–V hysteresis behavior could vary significantly across different interlayer conditions. I recommend that the authors provide R/F JV and hysteresis indices for all device conditions.

Reviewer #2

(Remarks to the Author)
This manuscript presents an outstanding and highly impactful study on the buried SnO2/perovskite interface in perovskite solar cells. The authors identify tin-ion migration and interfacial instability as key origins of device degradation and introduce a synergistic polymer-modified tin oxide strategy that simultaneously strengthens interfacial bonding, improves perovskite crystallinity, suppresses ion migration, and delivers excellent photovoltaic performance and operational stability. The work is comprehensive, mechanistically convincing, and of broad interest to the perovskite photovoltaics community. The reviewer suggests some items that need to be improved before it becomes acceptable in Nature Communications:1. While the

effectiveness of PED, PEM, and PVP is well-demonstrated, the initial introduction of these specific polymers is somewhat abrupt. The authors should add a brief discussion in the introduction or early in the results section explaining the exact rationale for selecting these three specific materials. 2. Recent literature highlights that polymeric interlayers can act as an 'elastic cushion' to release residual tensile strain at the buried interface. Given that the authors observed improved crystallinity and stability, it would be insightful to briefly discuss whether the PED interlayer also contributes to strain relaxation in the perovskite film. The authors could refer to any subtle shifts in the XRD peaks (if present) or simply cite relevant literature to enrich the mechanistic discussion.3. The authors present UPS (Supplementary Fig. 24) and KPFM (Supplementary Fig. 25) data demonstrating shifts in the work function at the bottom interface. To strengthen the manuscript's narrative, it is recommended to explicitly correlate these energy level shifts with the specific improvements in open-circuit voltage (Voc) observed in the J-V curves (Fig. 4a), thereby bridging the gap between material characterization and device physics.4. While the 2000-hour Maximum Power Point Tracking (MPPT) stability is impressive, the exact environmental conditions are not explicitly detailed in the main text. The authors should specify whether these measurements were conducted in an N2-filled glovebox or ambient air, the approximate temperature during tracking, and whether the devices were encapsulated. Adding a brief sentence to clarify this will improve the reproducibility of the work.5. There is a noticeable inconsistency regarding the substrate used. The main text mentions the use of an "ITO" substrate (e.g., ITO/SnO2/...). However, in Supplementary Note 2 (Perovskite thin-film peeling process), it is described as being "applied onto a clean glass substrate (Glass/FTO)." The authors must carefully check and unify the substrate descriptions throughout the manuscript and the Supplementary Information.6. Supplementary Note 2 describes the clever perovskite thin-film peeling process used to expose the buried interface. However, mechanical peeling can sometimes induce microscopic cracks or stress. The authors should add a brief comment regarding the yield of this technique and whether any mechanical stress artifacts were observed, reassuring the reader that the characterized bottom interface accurately reflects the intact device.7. Supplementary Fig. 26 illustrates the film aged under 40% RH with 'light bulb illumination.' Since incandescent bulbs generate significant heat, the authors should briefly clarify the approximate temperature the film reached to validate that the degradation is primarily moisture/light-driven rather than purely thermal.Overall, this is an elegant, carefully executed, and highly convincing manuscript. The work provides important new insight into buried-interface degradation in perovskite photovoltaics and proposes a practically relevant strategy to mitigate it. I believe this study will be of substantial interest to researchers working on perovskite solar cells, interface engineering, and photovoltaic stability. After revising the points mentioned above, I view it as a strong contribution suitable for publication in Nature Communications.

Reviewer #3

(Remarks to the Author)
In the manuscript entitled “Mitigating tin ion migration and reinforcing buried interface via synergistic polymer-modified tin oxide”, et al. introduce polymeric interlayers to enhance interfacial bonding and suppress tin-ion migration, leading to improved efficiency and stability of perovskite solar cells. While the study is systematic and provides useful mechanistic insights, I believe the level of novelty is insufficient for publication in Nature Communications. My specific comments are as follows:

First, the concept of incorporating polymeric interlayers between the perovskite and SnOx has been widely reported in the literature. As such, the incremental advance presented here limits the overall novelty and impact of the work.

Second, although the devices demonstrate respectable efficiencies (up to 26.58% reverse scan / 25.64% forward scan for small-area devices), the use of Spiro-MeOTAD as the hole transport layer raises concerns regarding long-term stability and cost. Notably, the stability measurements are conducted using a different HTL system (polyTPD-based), making it difficult to directly correlate efficiency and stability within the same device architecture. Furthermore, the stability data (Figure 4e,f) are presented only as normalised efficiency without reporting absolute values, which limits the assessment of real performance degradation.

Third, the devices exhibit non-negligible hysteresis. Therefore, stabilised power output (maximum power point tracking) should be reported instead of relying primarily on reverse scan efficiencies.

Overall, while the work is technically sound, the limited novelty and inconsistencies in performance evaluation reduce its suitability for publication in a high-impact journal.

Reviewer #4

(Remarks to the Author)
This work employs a polymer interlayer to modify the interface between SnO2 and perovskite, achieving excellent device performance and stability, with scalable validation demonstrated in large-area solar cells. However, I believe the manuscript requires additional characterization to address gaps in the experimental procedure and strengthen the logical consistency. The authors should resolve the following issues to meet the high standards of Nature Communications, which are currently not fully satisfied.
My comments are as follows:

1.Are these polymers n-type? Their electrical conductivity must be experimentally verified. A thorough discussion and characterization of the polymer properties are also required.

2.How did the authors determine the optimal concentration of the polymer interlayer? Was it based on device efficiency, carrier extraction, or defect passivation capability? No relevant discussion or investigation is provided throughout the

manuscript. How do different concentrations influence interfacial charge extraction, defect passivation, and device performance? The authors should present a scientific screening process and justification for the polymer interlayer concentration, rather than arbitrarily fixing it at 1 mg/mL. Such logical rigor is expected for Nature Communications.

3.The authors pre-synthesized monomers into polymers before device fabrication. I have several questions regarding this procedure:
1)How did the authors aim for 80 °C as the optimal polymerization temperature? AIBN initiates radical decomposition at approximately 70 °C. Supporting evidence must be provided.
2)To what extent does polymerization proceed under stirring overnight at 80 °C? Is the reaction complete or incomplete? A specific optimal polymerization time should be identified, rather than using the vague term “stirred overnight”. The investigation and supporting data for these points should be included.
3)What is the thickness of the polymer layer spin-coated onto SnO_2 after stirring overnight at 80 °C? How was the spin condition of 3000 rpm for 30 s determined? Was it based on device performance, carrier extraction efficiency, or defect passivation capability? These details are missing. Please justify the choice of spin speed and establish its correlation with film thickness.
4)Please provide photographs demonstrating the solubility of PED, PEM, and PVP in the solvents used to confirm their solubility.

4.During perovskite annealing at 150 °C, does the process affect the polymer interlayer? Could further polymerization occur? What implications does this have on device performance? This requires investigation and supporting evidence.

5.Please replace the term “bottom interface” with “buried interface”, which is more accurate in this context.

6.The authors conclude that “these results confirm that the bottom interface is significantly more sensitive to light-induced degradation than the top surface”. However, no justification is provided for this conclusion beyond optical microscope observations. Please provide a thorough explanation to support this claim.

7.Please improve Figure S7 by adding a scale bar, clearly defining the measurement range, and experimental conditions, as the current presentation is disorganized. Please label the conditions and elements in Figures 3a and 3b for greater clarity.

8.How can the pronounced increase in hysteresis for PED-modified devices be reconciled with the claim that PED effectively suppresses ion migration? Please address this apparent contradiction.

9.Why does the EQE show a pronounced enhancement in the 350–400 nm region, indicating improved generation and extraction of photogenerated carriers near the bottom interface? Please provide a mechanistic explanation and relevant literature citations.

10.The entire study focuses on a polymer interlayer deposited on SnO_2. Why did the authors characterize the energy levels of polymer-doped SnO_2 using UPS, rather than measuring the energy levels of the polymer interlayer alone? This approach is inappropriate and reinforces concerns raised in Comment 1 regarding whether these polymers function as n-type semiconductors. Please address this issue critically and revise accordingly.

11.The line widths throughout the manuscript are inconsistent. Please unify them for improved presentation and readability.

12.The sources of the starting materials used for polymer synthesis, including the grade, specification, and supplier of AIBN, are not reported in the Experimental Section.

Version 1:

Reviewer comments:

Reviewer #1

(Remarks to the Author)
All of my concerns have been well addressed by the authors. I recommend this manuscript for publication.

Reviewer #2

(Remarks to the Author)
The authors have adequately addressed all reviewer comments, and the manuscript is now suitable for acceptance.

Reviewer #3

(Remarks to the Author)
In the revised version of the manuscript entitled "Mitigating Tin Ion Migration and Reinforcing the Buried Interface via Synergistic Polymer-Modified Tin Oxide," the authors have satisfactorily addressed my comments, as well as those raised by the other reviewers. The revisions are thorough, and the changes made to the manuscript are appropriate and well

justified.

Overall, I believe the manuscript has been significantly improved and now meets the publication standard of Nature Communications. I therefore recommend its acceptance for publication in its current form.

Reviewer #4

(Remarks to the Author)
I have thoroughly checked the authors'point-by-point responses to all reviewer comments. Every concern I raised, alongside those from the other referees, has been comprehensively and satisfactorily resolved. No further amendments are needed, and the manuscript is suitable for publication in Nature Communications in its current version.

Open Access This Peer Review File is licensed under a Creative Commons Attribution 4.0 International License, which permits use, sharing, adaptation, distribution and reproduction in any medium or format, as long as you give appropriate credit to the original author(s) and the source, provide a link to the Creative Commons license, and indicate if changes were made.
In cases where reviewers are anonymous, credit should be given to 'Anonymous Referee' and the source.
The images or other third party material in this Peer Review File are included in the article's Creative Commons license, unless indicated otherwise in a credit line to the material. If material is not included in the article's Creative Commons license and your intended use is not permitted by statutory regulation or exceeds the permitted use, you will need to obtain permission directly from the copyright holder.
To view a copy of this license, visit https://creativecommons.org/licenses/by/4.0/

A point-by-point response to reviewers' comments:

Reviewer 1:

Authors reports a strategy to stabilize the buried SnO_2/perovskite interface in n–i–p perovskite solar cells (PSCs) by introducing polymeric interlayers — poly(1-ethenylpyrrolidine-2,5-dione) (PED), poly(1-ethenyl-3-methylpyrrolidin-2-one) (PEM), and polyvinylpyrrolidone (PVP). The authors identify tin-ion migration from the SnO_2 electron transport layer as a key yet previously underappreciated degradation pathway and demonstrate that PED, in particular, forms a robust interfacial barrier that suppresses Sn diffusion, improves perovskite crystallinity, and significantly enhances both efficiency (certified PCE of 26.58%) and superior operational stability. This manuscript is timely and the experimental scope is broad. However, several concerns must be addressed before this work can be considered for publication.

Our response:

We sincerely thank the reviewer for the positive evaluation of our work and for recognizing the significance of stabilizing the buried SnO_2/perovskite interface through polymeric interlayers. We also appreciate the reviewer's constructive comments and suggestions, which have helped us improve the mechanistic rigor and clarity of the manuscript. In the revised manuscript, we have carefully addressed all the points raised by the reviewer, including additional depth-profiling evidence, clarification of Sn incorporation and migration, module scribing/GFF verification, and statistical analysis of forward/reverse-scan *J–V* hysteresis. Detailed point-by-point responses are provided below.

1. In Fig. 1c–f, perovskite films on polymeric interlayers show fewer voids than those on bare SnO_2, despite SnO_2 being more hydrophilic and expected to provide better precursor wetting. The authors should explain this counterintuitive result with supporting evidence such as contact angle measurements and a discussion of how the polymer surface modifies perovskite nucleation kinetics.

Our response:

We sincerely thank the reviewer for highlighting this critical point regarding interfacial wetting and morphological evolution.

The reviewer logically presumed that the bare SnO_2 would be more hydrophilic. However, to explicitly answer this question, we conducted contact angle measurements. The corresponding results are shown in **Fig. R1** and have been included in **Supplementary Fig. 13** in the revised manuscript. The experimental data reveals a different reality: the polymeric interlayers render the surface more hydrophilic than our bare SnO_2 substrate. The contact angle decreased from 26.25° on bare SnO_2 to 12.95°, 17.60°, 17.05°after PED, PEM and PVP treatment, respectively. Beyond this, we also conduct the UV-ozone treatment to the substrate before spin-coating the perovskite precursor.

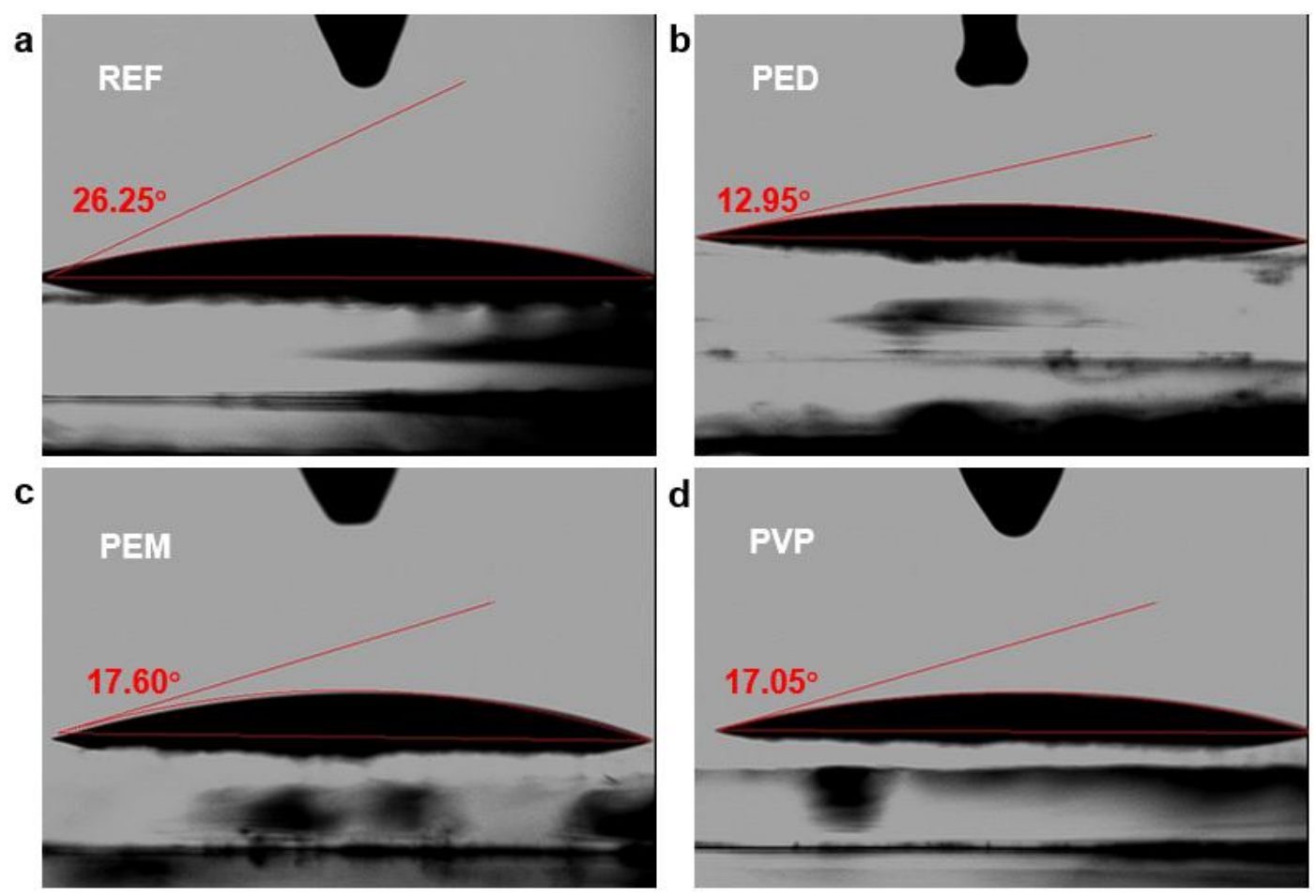

Fig. R1. Contact angle of Glass/ITO/SnO2 with/without polymer interlayers.

With this experimental evidence, morphological evolution is no longer counterintuitive but rather follows classical wetting theories perfectly. The smaller contact angle of the polymer interlayer treated substrate indicates a higher surface energy. Thermodynamically, the increase of the heterointerface (between the perovskite and underneath surface) energy can flatten the grain boundary grooves based on **Equation R1**[1, 2]. This aligns well with our cross-section SEM results.

$$\frac{\gamma_{GB}}{\gamma_{HI}} = 2\,cos\left(\frac{\varphi}{2}\right) = 2\,sin(\theta)$$

Equation R1

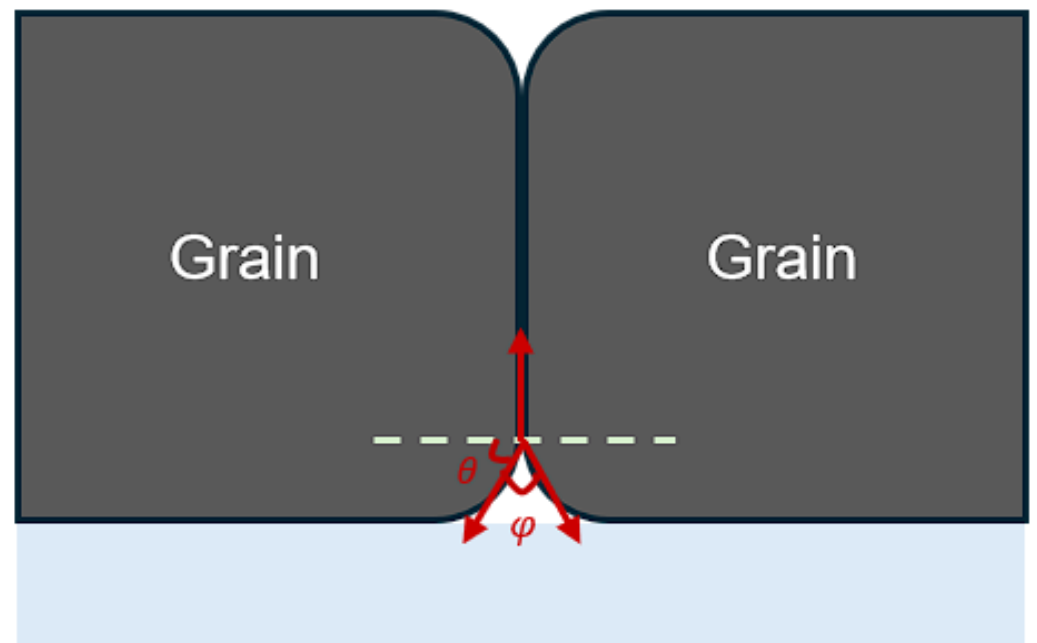

Figure. R2. Illustration of grain boundary groove.

where γ_{GB} is the grain boundary energy, γ_{HI} is the heterointerface energy, φ and θ is the dihedral and side angles of a grain boundary groove as illustrated in **Fig. R2**[3].

Furthermore, to fully address the reviewer's highly insightful question regarding how the polymer surface modifies the perovskite nucleation kinetics, we re-examined both our morphological and crystallographic data. As the reviewer insightfully prompted, we evaluated the nucleation kinetics. Importantly, our cross-sectional SEM and planar SEM reveals that the bulk grain size remains largely unchanged, while our standard XRD data shows no obvious peak shifts but a notable increase in the intensity ratio of the α-$FAPbI_3$ phase to the PbI_2 phase.

Based on these concrete experimental observations, we propose that the polymeric interlayer modifies the nucleation and crystallization kinetics not by reducing nucleation density (which would enlarge grains), but through homogenizing the nucleation process and promoting phase conversion, which synergistically eliminates interfacial voids:

1. Uniform Wetting and Homogeneous Nucleation Template:

Our contact angle measurements show that the polymer renders the surface more hydrophilic than the bare SnO_2. Bare SnO_2 often possesses heterogeneous surface states, leading to patchy precursor wetting and localized dewetting that spawn interfacial voids[4]. In contrast, the highly polar C=O groups on the polymer provide excellent and spatially uniform precursor wetting. This creates a homogeneous chemical template for nucleation, allowing the perovskite to crystallize conformally across the entire interface, thus eliminating bottom voids without necessarily altering the final bulk grain size.

2. Modulated Phase-Conversion Kinetics**:** The strong Lewis acid-base coordination between the polymer's C=O groups and the precursor ions (e.g., Pb^{2+}) profoundly modulates the crystallization kinetics[5, 6]. Rather than simply retarding the overall growth, this coordination stabilizes the intermediate phases during thermal annealing[7]. It prevents the rapid, premature precipitation of unreacted PbI_2 at the buried interface, ensuring a more thorough and complete chemical reaction to form the pure α-$FAPbI_3$ phase. This kinetic regulation perfectly corroborates our XRD results (**Fig 2C**), which exhibit an increased α-$FAPbI_3$/ PbI_2 ratio, indicating superior phase purity and suppressed interfacial PbI_2 segregation.

Following your highly constructive suggestion, we have incorporated the contact angle data and a rigorous discussion of this homogeneous nucleation and phase-conversion kinetics into the revised manuscript.

Revised parts in the manuscript:

In page 4 of the revised manuscript:

To elucidate the root cause of interfacial voids, we investigated the interfacial wetting and surface energy after the polymer treatment. Contact angle measurements (**Supplementary Fig.13**) reveal enhanced hydrophilicity on the polymer-modified substrates compared to bare SnO_2. The increase of the surface energy helps flatten the grain boundary grooves and eliminate the voids largely[28,29,30].

In page 6 of the revised manuscript:

This increased α-$FAPbI_3$/PbI_2 ratio also suggests that the strong Lewis acid-base coordination between the polymer's C=O groups and Pb^{2+} ions stabilizes intermediate phases during annealing. This kinetic modulation prevents premature PbI_2 precipitation, thereby ensuring a more complete conversion to the pure α-$FAPbI_3$ phase.

2. The authors claim that the buried interface is the primary origin of perovskite degradation, yet the supporting evidence (e.g, optical microscopy (Supp. Fig. 5) and post-aging SEM/XPS) does not conclusively rule out co-contributions from the bulk or top surface. I recommend that the authors provide quantitative, depth-profiling evidence (e.g., depth-profiling XPS or SIMS).

Our response:

We thank the reviewer for this important suggestion. We agree that optical microscopy and post-aging surface characterization alone cannot conclusively exclude contributions from the bulk or the top interface. Therefore, in the revised manuscript, we have softened the original wording and no longer describe the buried interface as the exclusive or sole origin of degradation. Instead, we refer to it as a major degradation-sensitive region and a key contributor to device degradation.

To provide quantitative depth-resolved evidence, we added XPS depth-profiling measurements of fresh and aged devices in **Fig. R3**, **Fig. R4**. The fresh device shows relatively well-separated elemental distributions corresponding to the Au/HTL, perovskite, buried interface, and SnO_2/ITO regions. After aging, however, the buried-interface region shows pronounced Pb/I/N compositional decoupling compared with the fresh device. In particular, the N-containing organic component is depleted relative to the Pb/I-containing inorganic species in part of the buried-interface region, suggesting preferential loss or redistribution of the organic perovskite components.

In addition, the aged depth profile shows increased overlap between Sn-related signals and the perovskite-related Pb/I region near the buried interface, suggesting Sn-related intermixing or migration from SnO_2 into the perovskite side during aging. This result is consistent with our post-aging bottom-side XPS, STEM-EDS, ICP-AES, and Sn K-edge XANES analyses, which collectively support Sn-related ion migration from SnO_2 as an important degradation pathway.

Importantly, we do not claim that degradation from the bulk or top surface is completely absent. Rather, the added depth-profiling XPS results show that the buried SnO_2/perovskite interface undergoes more pronounced chemical redistribution than the top interface under the same aging condition. These results strengthen our conclusion that the buried interface is a major degradation-sensitive region in these n–i–p devices.

We have modified the related discussion and incorporated the XPS depth-profile data in **Supplementary Fig. 9** and **10** of the revised manuscript.

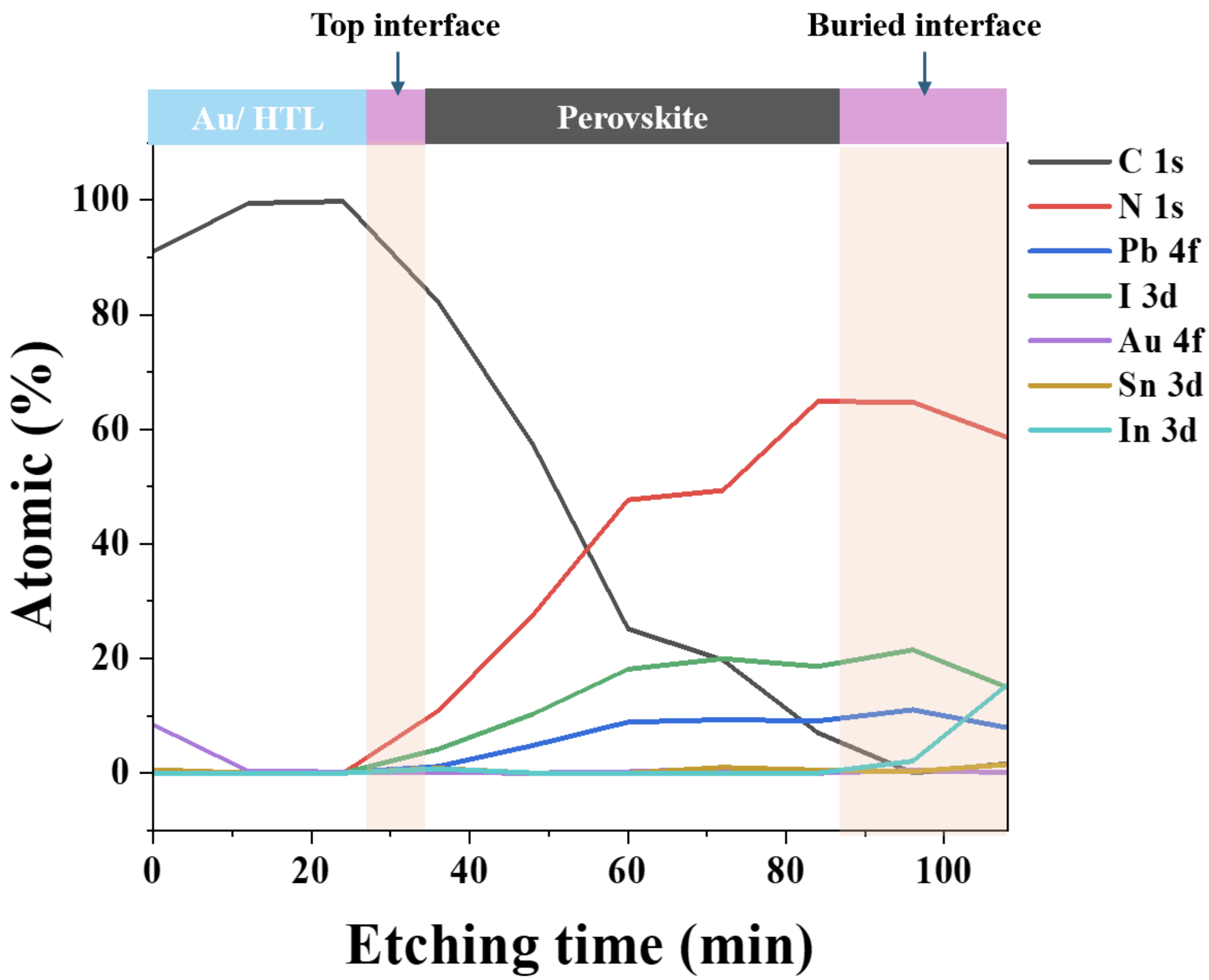

Fig. R3. Interface-focused XPS depth profile of the fresh device.

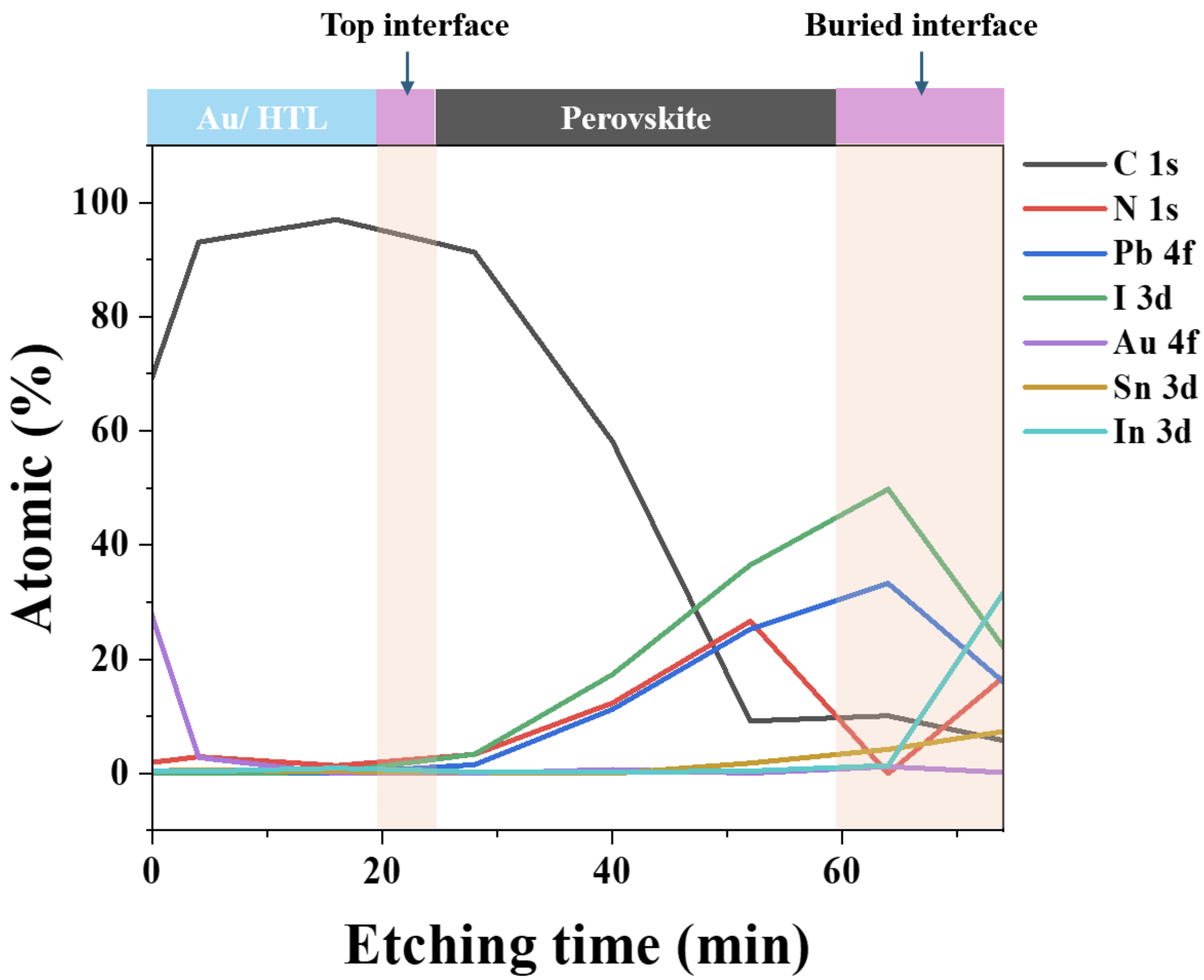

Fig. R4. Interface-focused XPS depth profile of the aged device (aging condition: encapsulated device under one-sun illumination for 170 hours).

Revised parts in the manuscript:

In page 2 of the revised manuscript:

Here, we reveal that instability at the buried SnO_2/perovskite interface triggers structural and chemical decomposition of the perovskite layer. To address this issue, we introduce polymeric interlayers that simultaneously reinforce interfacial bonding and suppress tin-ion migration. Poly(1-ethenylpyrrolidine-2,5-dione) (PED) forms the most robust and chemically compatible interface, yielding enhanced phase purity, reduced defect density, and significantly mitigated tin diffusion. These findings identify the buried SnO_2/perovskite interface as a key degradation-

sensitive region under light aging and highlight interfacial polymer design as an effective strategy for improving the intrinsic stability of perovskite photovoltaics.

In page 5 of the revised manuscript:

Optical microscopy (OM) images revealed a more pronounced color change at the buried side than at the top surface after aging (**Supplementary Fig. 14**), suggesting stronger degradation near the buried interface. This observation is further supported by depth-profile XPS of fresh and aged devices. Compared with the fresh device, the aged device shows pronounced Pb/I/N compositional decoupling near the buried SnO_2/perovskite interface, where the N-containing organic component is depleted relative to Pb/I-containing inorganic species in part of the buried-interface region (**Supplementary Figs. 9, 10**).

In page 10 of the revised manuscript:

This work identifies the buried interface between the SnO_2 electron transport layer and the perovskite absorber as a key degradation-sensitive region in perovskite solar cells.

3. In Fig. 3f, the fresh REF device already contains 0.83 at% Sn while the fresh PED device contains only 0.34 at% (a significant difference before any aging). If this reflects Sn incorporation during fabrication being blocked by PED, this is an important finding that deserves dedicated discussion; if it lies within measurement uncertainty, replicate measurements with error bars are required.

Our response:

We sincerely thank the reviewer for highlighting this critical detail. The reviewer is exceptionally perceptive in noticing the significant difference in Sn content between the fresh REF and PED devices.

We fully agree that distinguishing whether this difference originates from measurement uncertainty or intrinsic physical mechanisms is crucial. Therefore, we would first like to clarify the rigorousness of our ICP-AES methodology to confirm that this is a highly reliable data point rather than a measurement artifact:

1. Reliability and minimal measurement uncertainty: To achieve the required detection limits and ensure bulk-level accuracy for our ICP-AES measurements, we meticulously scraped and collected

perovskite powder from approximately 30 individual, fully fabricated devices (yielding about 0.2-0.5 mg per device to accumulate enough amount of powder for testing condition). Therefore, each data point inherently represents a statistical average of 30 devices, effectively eliminating random device-to-device variations. Furthermore, the values presented in **Fig. R5** are derived from two completely independent experimental batches, which required the destructive sampling of over 60 devices per condition. Given this extensive sampling scale, measurement uncertainty is substantially minimized.

We realize that the original manuscript lacked the necessary clarification that the plotted bars represented the average of these two independent measurements. Following your constructive suggestion, we have revised **Figure 3** to enhance its clarity and have provided the exact raw data in **Table R1** (which is added in **Supplementary Table 3** in the revised manuscript) to further demonstrate the excellent reproducibility of our results.

2. The physical finding: Suppression of initial Sn incorporation:

Having established the robustness of the data, we completely agree with your highly insightful deduction. The significantly lower Sn content in the fresh PED device (0.34 at%) compared to the fresh REF device (0.83 at%) indeed reflects that the PED interlayer acts as a strong physical and chemical barrier during the high-temperature thermal annealing step of fabrication. It successfully suppresses the initial diffusion and incorporation of Sn from the SnO_2 substrate into the perovskite lattice.

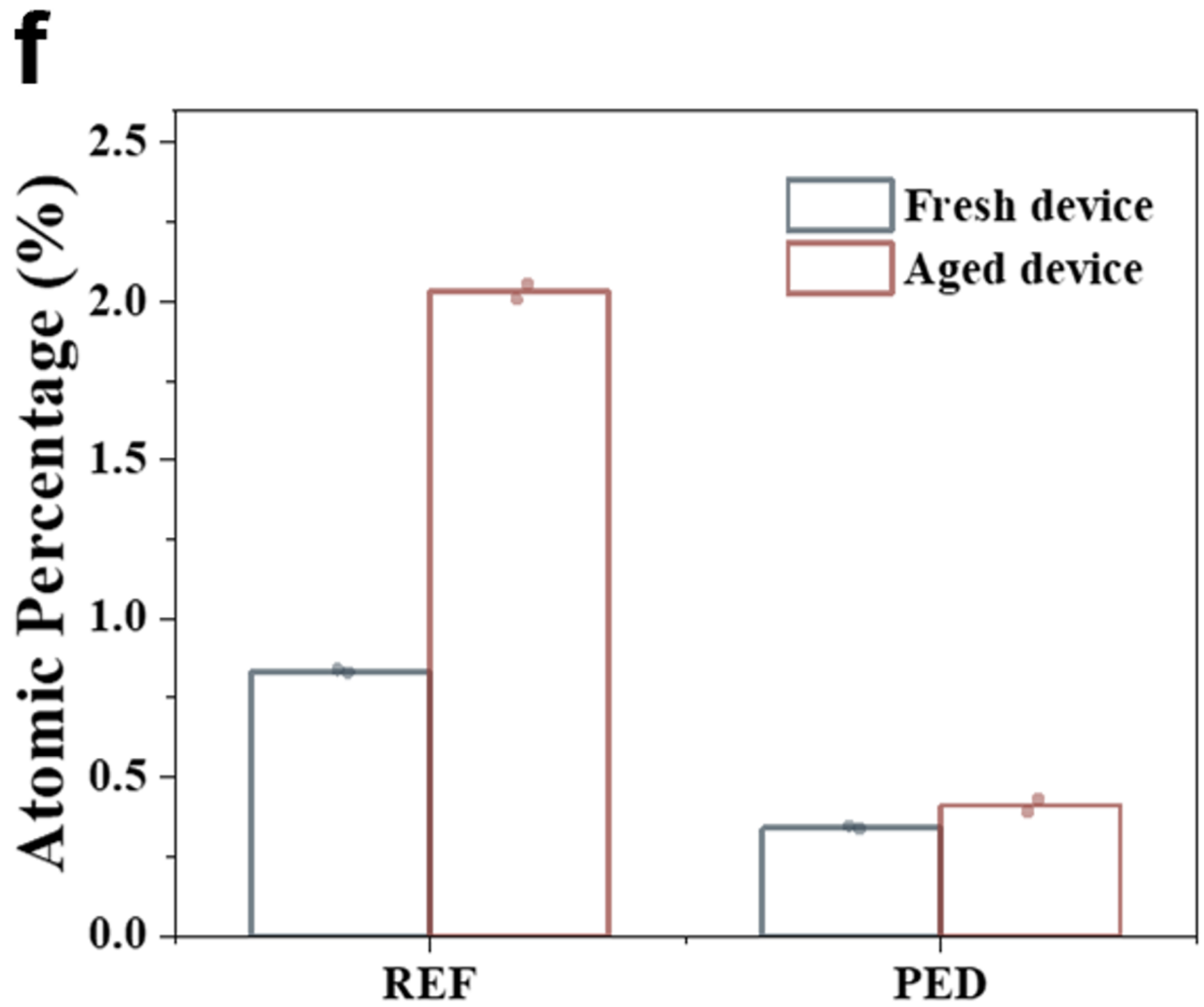

Fig. R5. ICP-AES results of tin content in aged and fresh samples.

Table R1. Data of the ICP-AES graph.

Sample	Device condition	1st test atomic percentage(%)	2nd test atomic percentage(%)
REF	Fresh	0.82999	0.83874
	Aged	2.00494	2.05388
PED	Fresh	0.33986	0.34752
	Aged	0.39092	0.43194

Revised parts in the manuscript:

In page 12 of the revised manuscript:

Remove the ICP-AES method introduction in the Materials and Methods section.

In page 15 of the revised manuscript:

Add the ICP-AES method introduction in the Materials Characterization part:

ICP-AES was performed on Shimadzu ICPE-9000. ICP-AES tuning solution and chloric acid (trace metal grade) were from Alfa Aesar and Fisher Chemical, respectively. Standard element solution was purchased from the National Center of Analysis and Testing for Nonferrous and Electronic Materials. The standard solution was diluted into 1,000.0, 500.0, 200.0, 100.0 and 20.0 ppb with 1% HCl for the calibration curve. Milli-Q water (18.2 MΩ) was used in all experiments. Collected perovskite powder was scraped from approximately 30 individual, fully fabricated devices (yielding about 0.2-0.5 mg per device to accumulate enough amount of powder for testing condition).

4. While the GFF of 96.5% is stated in the manuscript, no experimental verification is provided. I recommend that the authors include SEM images of the P1, P2, and P3 scribed lines to directly confirm the GFF and scribing quality.

Our response:

We thank the reviewer for this helpful suggestion. We agree that the geometric fill factor (GFF) and scribing quality should be experimentally verified rather than only stated as a design parameter. In the revised Supplementary Information, we have added microscopic/SEM characterization of the module interconnection region, including the scribed dead zone associated with the P1/P2/P3 interconnection lines, to directly verify the scribing quality and the geometric dimensions (**Supplementary Fig.31**).

As shown in **Fig. R6**, the designed sub-cell width of the perovskite module is 5200 μm, while the interconnection dead zone is approximately 180 μm, as confirmed from the microscopic/SEM measurements. Based on these values, the GFF is calculated as:

$$\mathrm{GFF} = \frac{5200}{5200 + 180} \times 100\% \approx 96.5\%.$$

The added SEM/microscopy images show a well-defined interconnection region without obvious over-scribing or severe damage to the adjacent active area. Therefore, the experimentally measured dimensions are consistent with the stated GFF of approximately 96.5%.

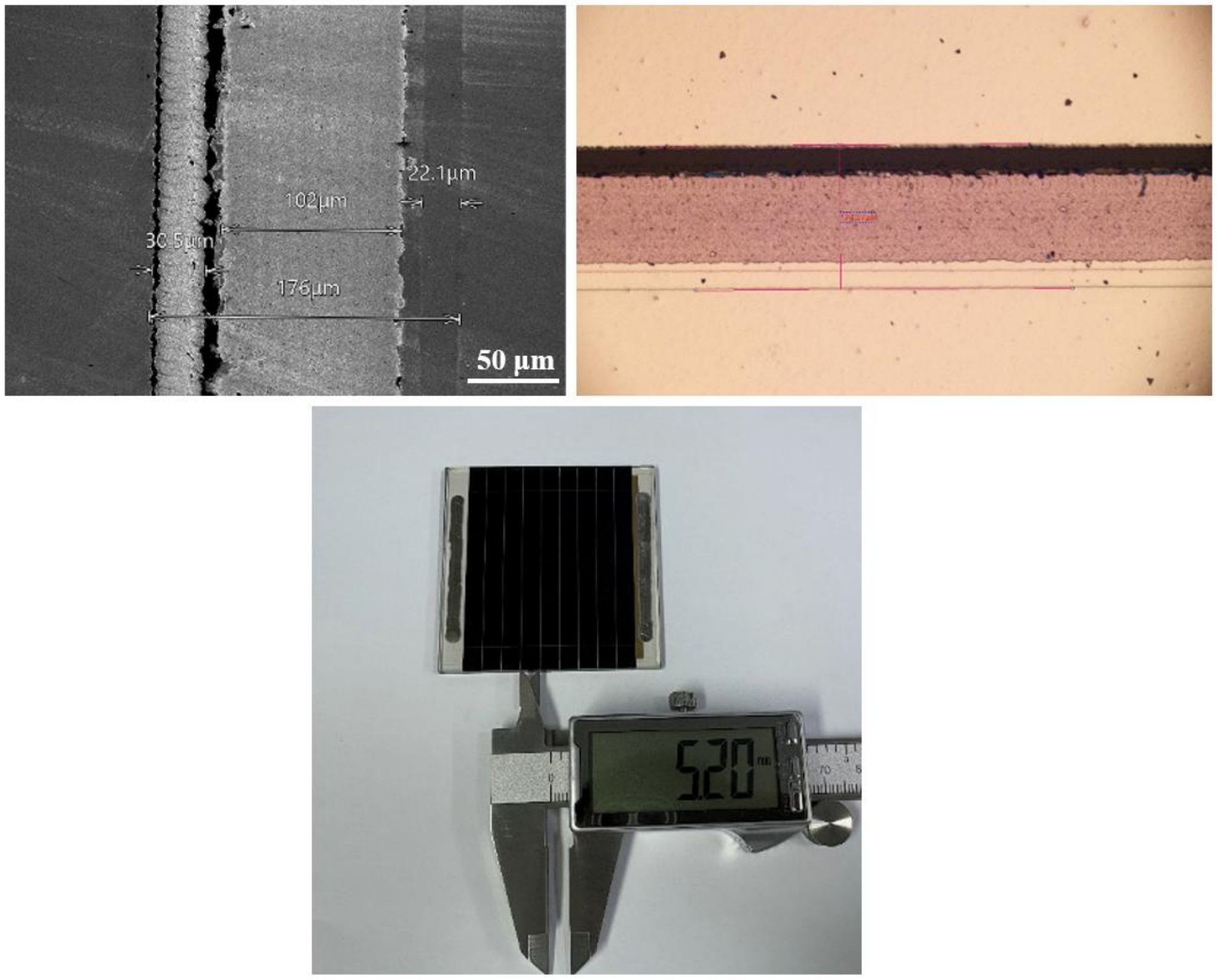

Fig. R6. Experimental verification of the module GFF and scribing quality.

<u>Revised parts in the manuscript:</u>

In page 14 of the revised manuscript:

The module geometric fill factor (GFF) was determined from the designed and experimentally verified interconnection geometry. The sub-cell width was approximately 5200 μm, while the interconnection dead zone defined by the P1/P2/P3 scribing region was approximately 180 μm, corresponding to a GFF of approximately 96.5%. The scribing quality and interconnection dimensions were further verified by SEM/optical microscopy images (**Supplementary Fig. 31**).

5. Since the polymeric interlayer insertion may influence interfacial charge extraction and ion migration dynamics, J–V hysteresis behavior could vary significantly across different interlayer

conditions. I recommend that the authors provide R/F JV and hysteresis indices for all device conditions.

Our response:

We thank the reviewer for this helpful suggestion. We agree that polymeric interlayers may affect both charge extraction and scan-dependent ion/charge dynamics, and therefore J–V hysteresis should be evaluated for all device conditions. To address this point, we have added statistical reverse- and forward-scan *J–V* analysis for REF, PED-, PEM-, and PVP-modified devices. The statistics were obtained from 20 devices for each condition. As shown in **Fig. R7a**, both reverse- and forward-scan PCEs are provided for all device groups. We further calculated the hysteresis index using $HI = (PCE_{\text{reverse}} - PCE_{\text{forward}})/PCE_{\text{reverse}} \times 100\%$. The corresponding HI distributions are shown in **Fig. R7b**, and the mean ± s.d. values are summarized in **Table R2**. The PED-modified devices exhibit the lowest average hysteresis index among all conditions, indicating that the PED interlayer does not aggravate J–V hysteresis while maintaining high device performance. These results have been added to **Supplementary Fig. 33** and **Supplementary Table 6.**

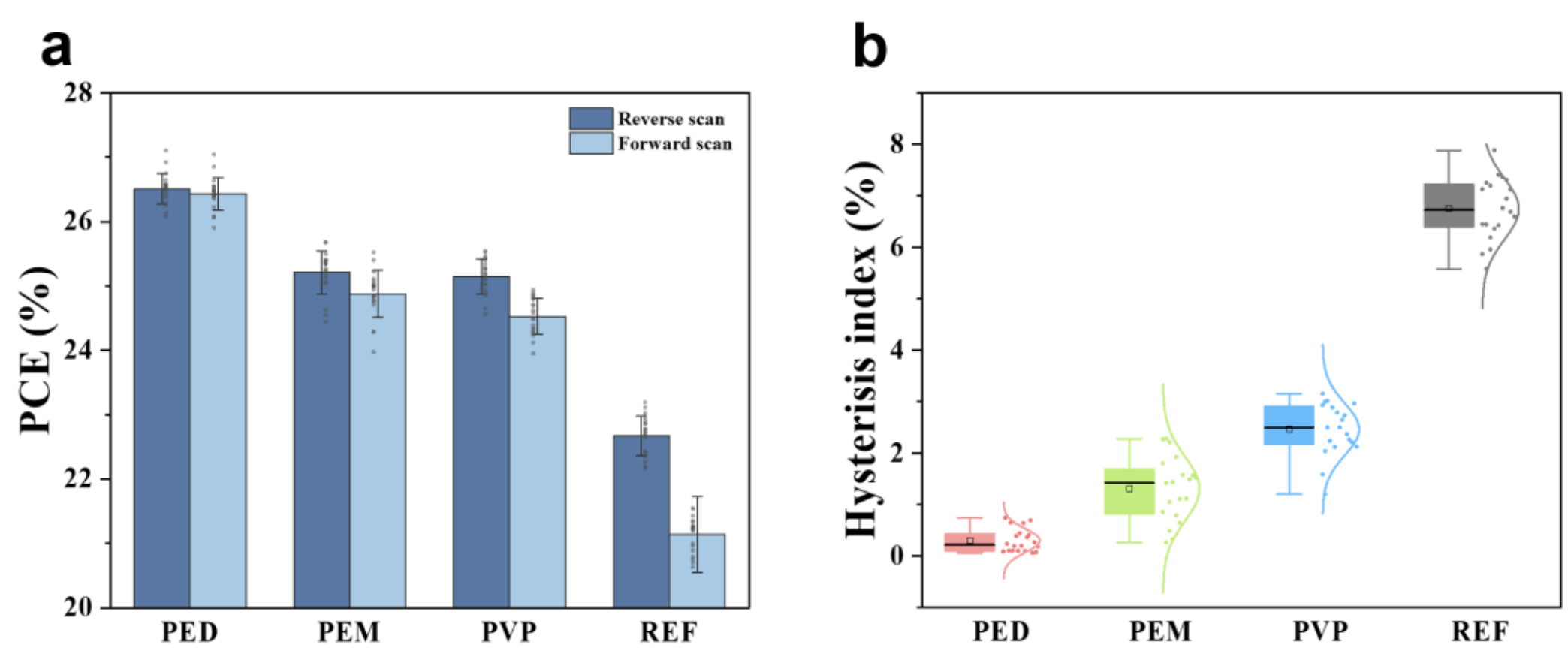

Fig. R7. (a) Statistical comparison of reverse- and forward-scan PCEs for PED-, PEM-, PVP-modified and reference devices. Bars represent mean values, error bars represent standard deviation, and overlaid points represent individual devices (n = 20). (b) Distribution of hysteresis index calculated from the same devices using $HI = (PCE_{\text{reverse}} - PCE_{\text{forward}})/PCE_{\text{reverse}} \times 100\%$. Box-plots show the median and interquartile range, and overlaid points represent individual devices.

Table R2. Statistical summary of forward/reverse J–V measurements and hysteresis indices.

Condtion	Reverse PCE (%)	Forward PCE (%)	Hysteresis Index
PED	26.51±0.24	26.43±0.25	0.302±0.228
PEM	25.21±0.34	24.88±0.37	1.309±0.622
PVP	25.15±0.27	24.53±0.28	2.465±0.505
REF	22.67±0.31	21.14±0.59	6.747±0.594

Reviewer 2:

This manuscript presents an outstanding and highly impactful study on the buried SnO2/perovskite interface in perovskite solar cells. The authors identify tin-ion migration and interfacial instability as key origins of device degradation and introduce a synergistic polymer-modified tin oxide strategy that simultaneously strengthens interfacial bonding, improves perovskite crystallinity, suppresses ion migration, and delivers excellent photovoltaic performance and operational stability. The work is comprehensive, mechanistically convincing, and of broad interest to the perovskite photovoltaics community. The reviewer suggests some items that need to be improved before it becomes acceptable in Nature Communications:

Our response:

We sincerely thank the reviewer for the very positive assessment of our work and for the constructive suggestions. In the revised manuscript, we have carefully addressed all the points raised by the reviewer, including the polymer-selection rationale, possible strain-relaxation effect, correlation between interfacial energetics and V_{oc}, stability-testing conditions, substrate descriptions, and peeling-process clarification. Detailed point-by-point responses are provided below.

1. While the effectiveness of PED, PEM, and PVP is well-demonstrated, the initial introduction of these specific polymers is somewhat abrupt. The authors should add a brief discussion in the introduction or early in the results section explaining the exact rationale for selecting these three specific materials.

Our response:

We sincerely thank the reviewer for pointing out this structural omission. We completely agree that the initial introduction of the polymers was somewhat abrupt and that the exact rationale should be explicitly stated.

Our selection process was based on a systematic, target-oriented design:

First, why polymeric materials over small molecules?

To effectively modify the buried interface, we specifically chose polymeric interlayers because they exhibit superior film-forming properties compared to small organic molecules. Polymers can form ultra-thin, highly uniform, and pinhole-free conformal layers on the SnO_2 substrate without severe aggregation[4,5]. Furthermore, the polymeric backbone provides high structural plasticity, allowing us to precisely tune the side-chain functional groups for targeted interactions.

Second, why the specific selection of PVP, PEM, and PED?

Rather than an arbitrary selection, these three polymers were chosen as ideal model systems to investigate how side-chain functional group variations affect Sn-ion trapping:

(1) PVP (Baseline Reference based on prior literature): Polyvinylpyrrolidone (PVP), featuring a single carbonyl (C=O) group, has been widely reported in previous literature as a standard Lewis-base passivator in PSCs[8,9]. We deliberately selected it as our baseline to represent conventional interfacial passivation.

(2) PEM (Steric/Hydrophobic Tuning): Building upon the classic PVP structure, PEM incorporates an additional methyl group ($–CH_3$). This was strategically selected to investigate whether increasing the hydrophobicity and altering the steric hindrance of the polymer layer could further enhance interfacial stability.

(3) PED (Optimal Design - Dual Coordination): As the core of our innovation, PED features a succinimide-like side chain with dual carbonyl groups. Recognizing from our initial tests that the single C=O in conventional PVP is insufficient to firmly trap the highly mobile Sn^{2+}/Sn^{4+} ions, we hypothesized and verified that this dual-carbonyl configuration enables an exceptionally strong, bidentate-like chelation. This specific structure completely locks the undercoordinated Sn/Pb ions, successfully shutting down the migration pathway.

<u>Revised parts in the manuscript:</u>

In page 3 of the revised manuscript:

To systematically address the buried interface issue, we rationally selected polymeric interlayers rather than small molecules, taking advantage of their superior uniform film-forming capabilities and highly tunable side-chains without severe aggregation[24,25]. Specifically, we introduced three structurally related polymers including polyvinylpyrrolidone (PVP), poly(1-ethenyl-3-

methylpyrrolidin-2-one (PEM), and PED as model systems. Building upon PVP, which has been widely utilized in prior literature as a standard Lewis-base passivator[26, 27], we systematically varied the functional groups. PEM introduces steric hindrance via an additional methyl group. Most importantly, PED, our ultimate design, features dual-carbonyl groups specifically chosen to enable strong, bidentate-like chelation.

2. Recent literature highlights that polymeric interlayers can act as an 'elastic cushion' to release residual tensile strain at the buried interface. Given that the authors observed improved crystallinity and stability, it would be insightful to briefly discuss whether the PED interlayer also contributes to strain relaxation in the perovskite film. The authors could refer to any subtle shifts in the XRD peaks (if present) or simply cite relevant literature to enrich the mechanistic discussion.

Our response:

We sincerely thank the reviewer for highlighting this critical point regarding interfacial wetting and morphological evolution.

The reviewer logically presumed that the bare SnO_2 would be more hydrophilic. However, to explicitly answer this question, we conducted contact angle measurements. The corresponding results are shown in **Fig. R1** and have been included in **Supplementary Fig. 13** in the revised manuscript. The experimental data reveals a different reality: the polymeric interlayers render the surface more hydrophilic than our bare SnO_2 substrate. The contact angle decreased from 26.25° on bare SnO_2 to 12.95°, 17.60°, 17.05°after PED, PEM and PVP treatment, respectively. Beyond this, we also conduct the UV-ozone treatment to the substrate before spin-coating the perovskite precursor.

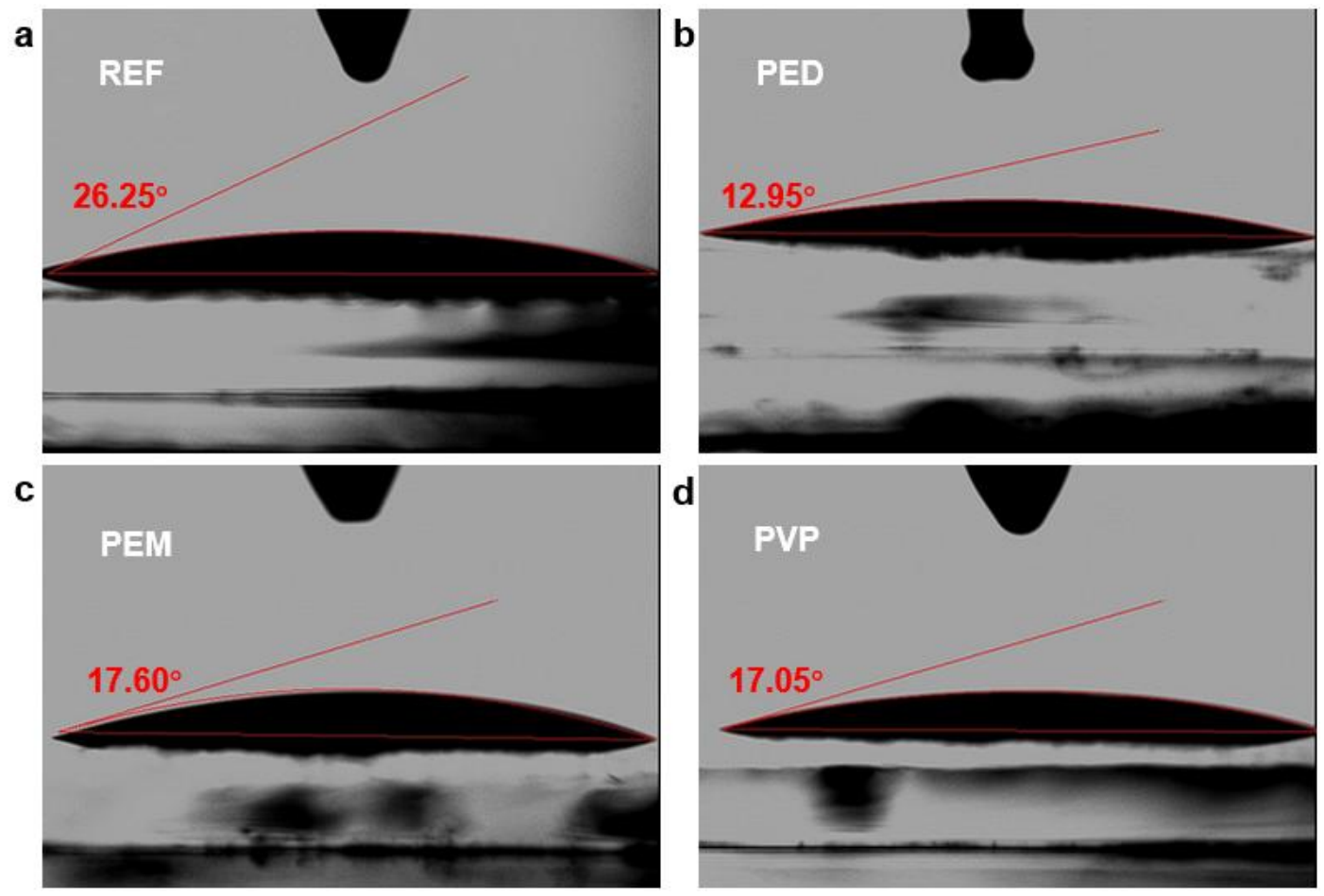

Fig. R1. Contact angle of Glass/ITO/SnO2 with/without polymer interlayers.

With this experimental evidence, morphological evolution is no longer counterintuitive but rather follows classical wetting theories perfectly. The smaller contact angle of the polymer interlayer treated substrate indicates a higher surface energy. Thermodynamically, the increase of the heterointerface (between the perovskite and underneath surface) energy can flatten the grain boundary grooves based on **Equation R1**[1, 2]. This aligns well with our cross-section SEM results.

$$\frac{\gamma_{GB}}{\gamma_{HI}} = 2\cos\left(\frac{\varphi}{2}\right) = 2\sin(\theta)$$

Equation R1

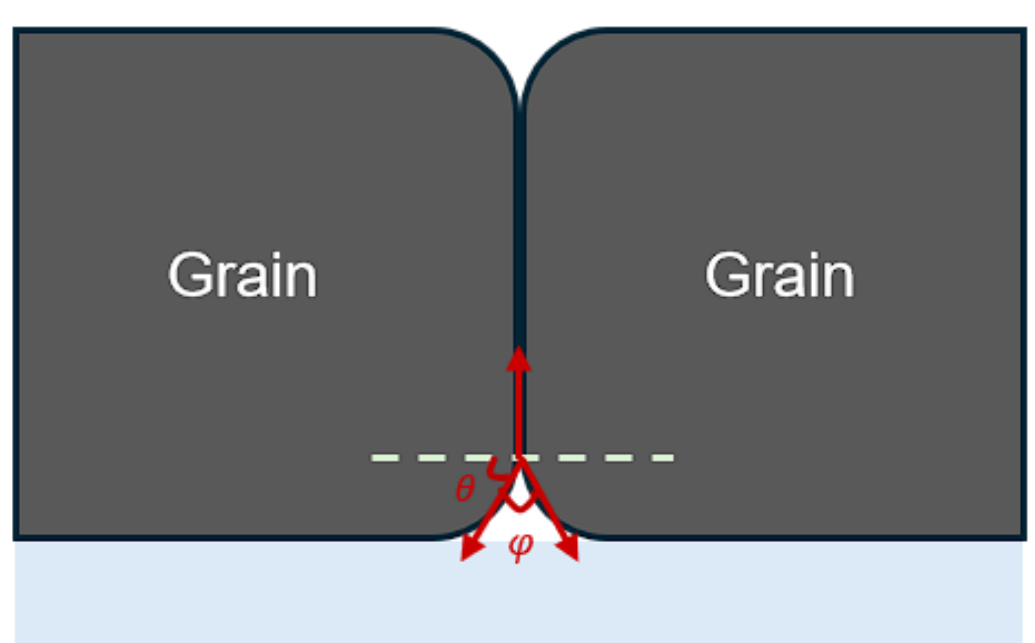

Figure. R2. Illustration of grain boundary groove.

where γ_{GB} is the grain boundary energy, γ_{HI} is the heterointerface energy, φ and θ is the dihedral and side angles of a grain boundary groove as illustrated in **Fig. R2**[3].

Furthermore, to fully address the reviewer's highly insightful question regarding how the polymer surface modifies the perovskite nucleation kinetics, we re-examined both our morphological and crystallographic data. As the reviewer insightfully prompted, we evaluated the nucleation kinetics. Importantly, our cross-sectional SEM and planar SEM reveals that the bulk grain size remains largely unchanged, while our standard XRD data shows no obvious peak shifts but a notable increase in the intensity ratio of the α-$FAPbI_3$ phase to the PbI_2 phase.

Based on these concrete experimental observations, we propose that the polymeric interlayer modifies the nucleation and crystallization kinetics not by reducing nucleation density (which would enlarge grains), but through homogenizing the nucleation process and promoting phase conversion, which synergistically eliminates interfacial voids:

1. Uniform Wetting and Homogeneous Nucleation Template:

Our contact angle measurements show that the polymer renders the surface more hydrophilic than the bare SnO_2. Bare SnO_2 often possesses heterogeneous surface states, leading to patchy precursor wetting and localized dewetting that spawn interfacial voids[4]. In contrast, the highly polar C=O groups on the polymer provide excellent and spatially uniform precursor wetting. This creates a homogeneous chemical template for nucleation, allowing the perovskite to crystallize conformally across the entire interface, thus eliminating bottom voids without necessarily altering the final bulk grain size.

2. Modulated Phase-Conversion Kinetics**:** The strong Lewis acid-base coordination between the polymer's C=O groups and the precursor ions (e.g., Pb^{2+}) profoundly modulates the crystallization kinetics[5, 6]. Rather than simply retarding the overall growth, this coordination stabilizes the intermediate phases during thermal annealing[7]. It prevents the rapid, premature precipitation of unreacted PbI_2 at the buried interface, ensuring a more thorough and complete chemical reaction to form the pure α-$FAPbI_3$ phase. This kinetic regulation perfectly corroborates our XRD results (**Fig 2C**), which exhibit an increased α-$FAPbI_3$/ PbI_2 ratio, indicating superior phase purity and suppressed interfacial PbI_2 segregation.

Following your highly constructive suggestion, we have incorporated the contact angle data and a rigorous discussion of this homogeneous nucleation and phase-conversion kinetics into the revised manuscript.

Revised parts in the manuscript:

In page 4 of the revised manuscript:

To elucidate the root cause of interfacial voids, we investigated the interfacial wetting and surface energy after the polymer treatment. Contact angle measurements (**Supplementary Fig. 13**) reveal enhanced hydrophilicity on the polymer-modified substrates compared to bare SnO_2. The increase of the surface energy helps flatten the grain boundary grooves and eliminate the voids largely[28,29,30].

In page 6 of the revised manuscript:

This increased α-$FAPbI_3$/PbI_2 ratio also suggests that the strong Lewis acid-base coordination between the polymer's C=O groups and Pb^{2+} ions stabilizes intermediate phases during annealing. This kinetic modulation prevents premature PbI_2 precipitation, thereby ensuring a more complete conversion to the pure α-$FAPbI_3$ phase.

3. The authors present UPS (Supplementary Fig. 24) and KPFM (Supplementary Fig. 25) data demonstrating shifts in the work function at the bottom interface. To strengthen the manuscript's narrative, it is recommended to explicitly correlate these energy level shifts with the specific improvements in open-circuit voltage (Voc) observed in the J-V curves (Fig. 4a), thereby bridging the gap between material characterization and device physics.

Our response:

We highly appreciate the reviewer for this extremely constructive suggestion. The reviewer is absolutely correct that bridging the material characterizations with the device physics significantly strengthens the narrative of our manuscript.

Following your insightful guidance, we have explicitly correlated the work function shifts observed in our UPS and KPFM measurements with the enhanced V_{oc} in our revised manuscript. The physical correlation is straightforward and robust:

The UPS and KPFM data (**Fig. R8** and **R9**) clearly demonstrate a shift in the work function at the buried interface upon polymeric modification. This shift may originate from the strong interfacial dipoles induced by the highly polar carbonyl (C=O) groups on the polymer chains. This dipole-induced work function shift effectively optimizes the energy level alignment between the SnO_2 electron transport layer and the perovskite absorber.

Consequently, this optimized energy alignment profoundly impacts the device physics in two ways, directly leading to the enhanced V_{oc} observed in Figure 4a: First, it increases the built-in potential (V_{bi}) across the device, providing a stronger driving force for carrier extraction. Second, the minimized energy offset significantly suppresses carrier accumulation and non-radiative recombination at the buried interface which is corroborated by our PL results (**Fig. 1G** and **H**). Together, these effects minimize the interfacial energy loss, thereby translating the modified material energetics directly into a boosted macroscopic V_{oc}.

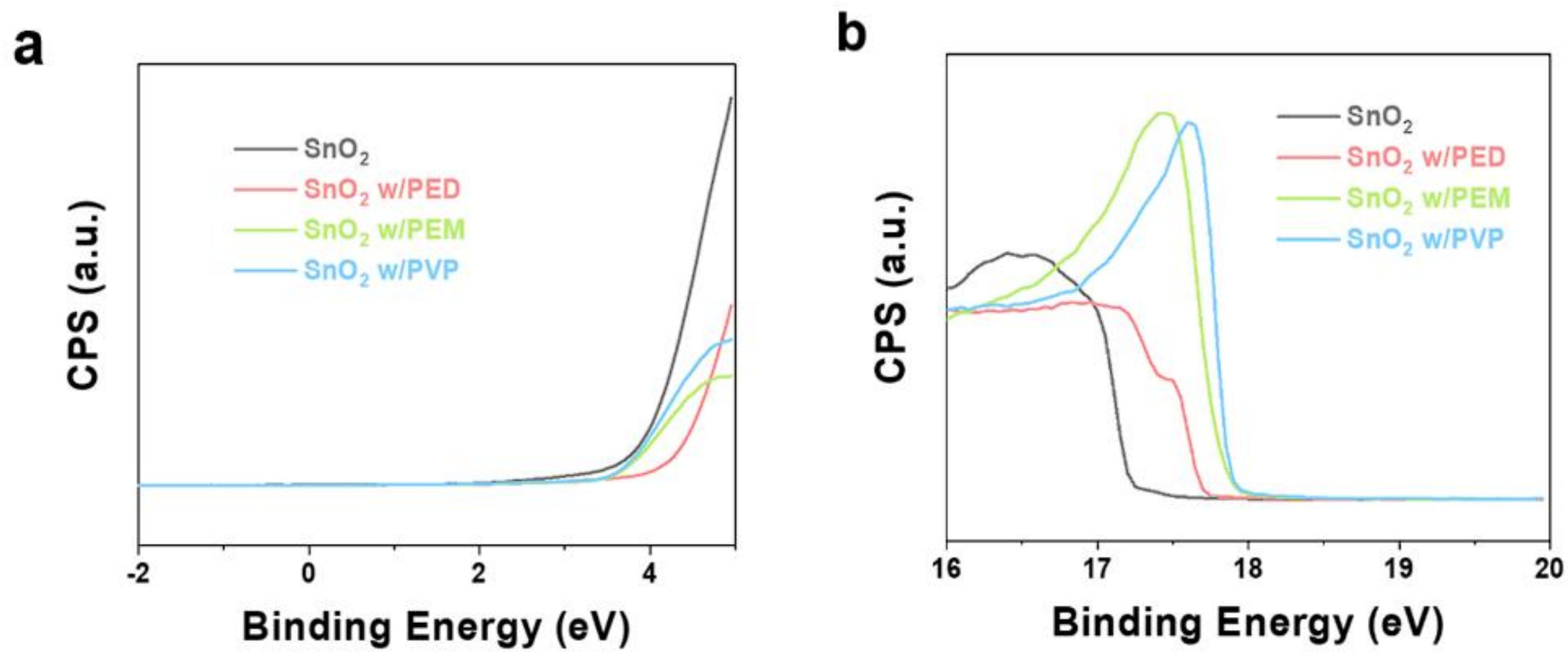

Fig. R8. UPS spectra of SnO_2 and polymer-modified SnO_2 buried interfaces: (a) valence-band onset region near the Fermi level and (b) secondary electron cutoff region.

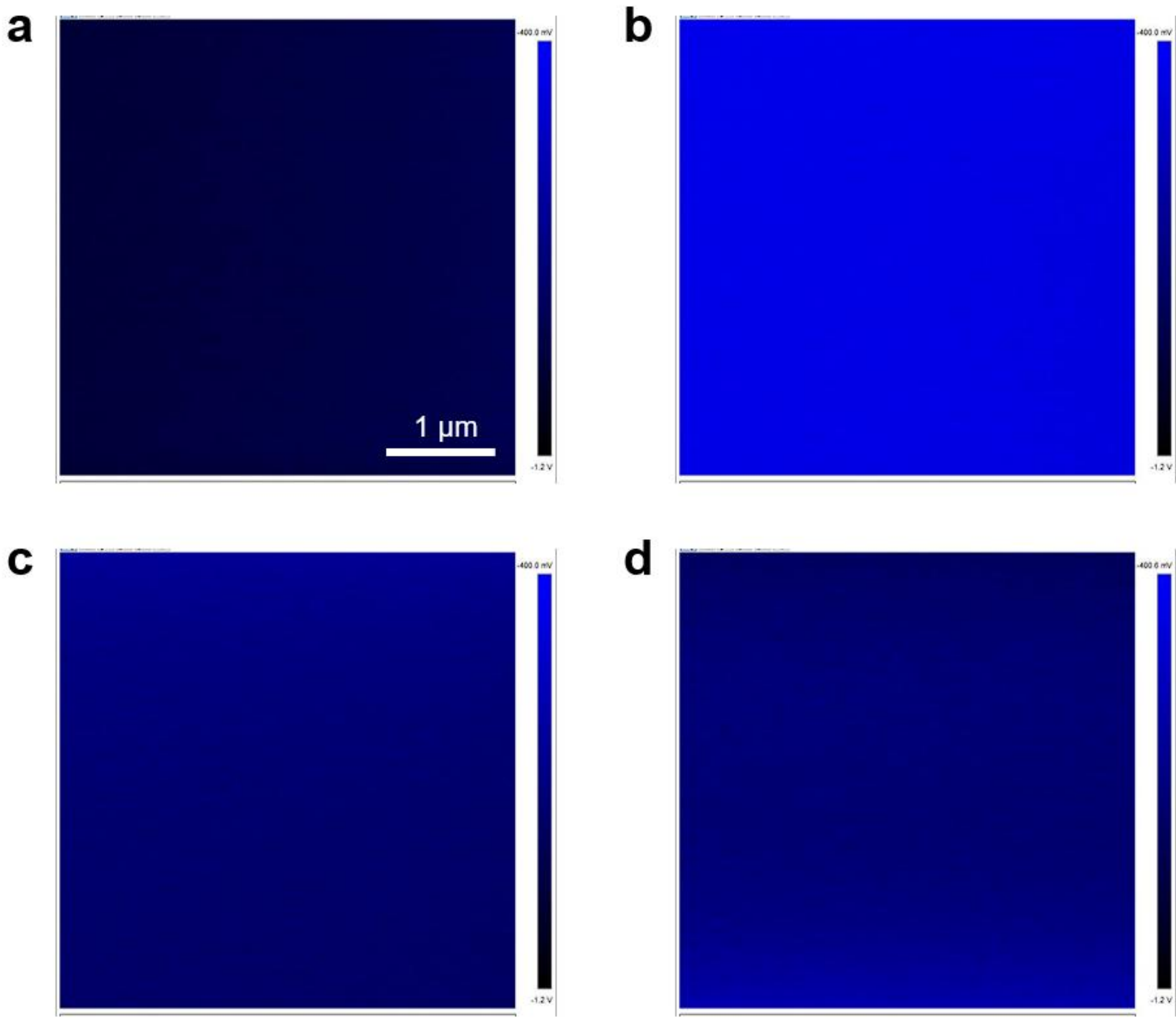

Fig. R9. KPFM for the buried interface work function (a) REF; (b) PED; (c) PEM; (d) PVP.

Revised parts in the manuscript:

In page 10 of the revised manuscript:

Crucially, this tailored energy level alignment provides a clear physical link between the material energetics and the observed device performance. The minimized energy offset, likely driven by the interfacial dipoles of the polar polymer chains, is expected to increase the built-in potential and effectively suppress non-radiative carrier recombination at the buried interface. Together, this optimized interfacial energetic largely contributes to the substantial enhancement in *Voc* observed in the *J*-*V* curves (**Fig. 4a**).

4. While the 2000-hour Maximum Power Point Tracking (MPPT) stability is impressive, the exact environmental conditions are not explicitly detailed in the main text. The authors should specify whether these measurements were conducted in an N2-filled glovebox or ambient air, the approximate temperature during tracking, and whether the devices were encapsulated. Adding a brief sentence to clarify this will improve the reproducibility of the work.

Our response:

We sincerely thank the reviewer for pointing out this important omission. The MPPT testing was conducted on encapsulated devices in an N_2-filled glovebox at 65 °C. We have now incorporated this information into the revised manuscript.

Revised parts in the manuscript:

In page 10 of the revised manuscript:

The operational stability of the encapsulated solar cell devices was further examined under MPPT using continuous 1-sun-equivalent white-light LED illumination at 65 °C in the N_2-filled glovebox (**Fig.4e**).

5. There is a noticeable inconsistency regarding the substrate used. The main text mentions the use of an "ITO" substrate (e.g., ITO/SnO2/...). However, in Supplementary Note 2 (Perovskite thin-film peeling process), it is described as being "applied onto a clean glass substrate (Glass/FTO)." The authors must carefully check and unify the substrate descriptions throughout the manuscript and the Supplementary Information.

Our response:

We appreciate the reviewer for pointing out this detail. While our standard devices are built on ITO substrates, the mechanical peeling process used to expose the buried interface (detailed in **Supplementary Note 2**) consistently shatters the relatively fragile ITO glass. To overcome this, we employed thicker and stronger FTO substrates to ensure a viable success rate for the peeling process. We expect this purely mechanical substitution to have a negligible impact on the underlying interfacial chemistry and the corresponding characterization results.

6. Supplementary Note 2 describes the clever perovskite thin-film peeling process used to expose the buried interface. However, mechanical peeling can sometimes induce microscopic cracks or

stress. The authors should add a brief comment regarding the yield of this technique and whether any mechanical stress artifacts were observed, reassuring the reader that the characterized bottom interface accurately reflects the intact device.

Our response:

We thank the reviewer for the insightful comment regarding the reliability of our film peeling technique. We have addressed the concerns regarding the yield and potential mechanical artifacts as follows:

1. Technique Yield**:** As specified in the revised **Supplementary Note 2**, by utilizing mechanically robust FTO substrates instead of thinner ITO glass, we significantly improved the robustness of the peeling process. We achieved a success rate of approximately 60% in obtaining complete, intact, and macroscopically damage-free perovskite films, which is sufficient for reliable and reproducible interface characterization.

2. Absence of Mechanical Artifacts**:** To confirm that the characterized buried interface accurately reflects the intrinsic morphology of the intact device, we refer the reviewer to the high-resolution SEM images of the buried interface (**Fig. 2a**). The morphology exhibits well-defined grain boundaries, clear crystal facets, and the absence of transgranular fractures or sharp, stress-induced micro-cracks that would typically result from mechanical tearing. The preservation of these delicate crystalline features directly demonstrates that our peeling method does not introduce significant mechanical artifacts.

Revised parts in the manuscript:

In page 8 of the revised supplementary information:

To ensure a high yield and reproducible results for the mechanical peeling process, robust FTO substrates were employed to withstand the necessary stress without shattering. The success rate for obtaining macroscopically intact and undamaged peeled films is approximately 60%. Furthermore, high-resolution SEM characterization of the exposed buried interface (as shown in **Fig. 2a**) reveals intrinsic grain boundary morphologies and well-preserved crystal facets. The absence of mechanical tearing patterns or stress-induced micro-cracks confirms that the characterized buried interface accurately reflects its original state within the intact device.\

7. Supplementary Fig. 26 illustrates the film aged under 40% RH with 'light bulb illumination.' Since incandescent bulbs generate significant heat, the authors should briefly clarify the approximate temperature the film reached to validate that the degradation is primarily moisture/light-driven rather than purely thermal.

Our response:

We sincerely thank the reviewer for pointing out this critical experimental detail. We completely agree that thermal effects must be decoupled from moisture/light-driven degradation when using heat-generating light sources like halogen bulbs.

In our setup, the illumination was provided by two 250 W halogen bulbs positioned approximately 40 cm away from the samples. Due to the inherent infrared emission of halogen lamps, we roughly test that the steady-state surface temperature of the perovskite films using a infrared thermometer, the temperature reached approximately 40°C during the aging process.

While this represents a mild thermal stress, it is important to note that the intrinsic thermal degradation threshold for our perovskite films typically requires sustained heating at >85 °C to induce pure thermal decomposition. Therefore, the severe structural breakdown observed in **Supplementary Fig.38** cannot be attributed solely to thermal degradation. Rather, it is fundamentally driven by the severe synergistic effects of light exposure and moisture (40% RH), accelerated by the mild local heating.

Per the reviewer's constructive suggestion, we have now explicitly detailed the light source type, working distance, and the estimated film temperature in the revised caption of **Supplementary Fig. 38** to ensure complete transparency.

Revised parts in the manuscript:

In page 48 of the revised supplementary information:

Supplementary Fig. 39. Perovskite film under 40% RH with light halogen bulb illumination at 40°C.

Reviewer 3:

In the manuscript entitled "Mitigating tin ion migration and reinforcing buried interface via synergistic polymer-modified tin oxide", Yang et al. introduce polymeric interlayers to enhance interfacial bonding and suppress tin-ion migration, leading to improved efficiency and stability of perovskite solar cells. While the study is systematic and provides useful mechanistic insights, I believe the level of novelty is insufficient for publication in Nature Communications.

Our response:

We thank the reviewer for the careful evaluation of our manuscript and for recognizing that the study is systematic and provides useful mechanistic insights. We also appreciate the reviewer's critical comments regarding novelty and performance evaluation. In the revised manuscript, we have substantially strengthened the discussion of the conceptual advance of our work relative to previous polymer-interlayer studies, emphasizing that our strategy is not only a polymer insertion approach but a polymer-modified SnO_2 platform that simultaneously reinforces the buried interface, suppresses Sn-related ion migration from SnO_2, modulates interfacial energetics, reduces interfacial defects, and improves both small-area and large-area device performance. We have also added and clarified stability data, absolute PCE values, stabilized power-output information, and hysteresis analysis to present the device performance more consistently. Detailed point-by-point responses are provided below.

First, the concept of incorporating polymeric interlayers between the perovskite and SnOx has been widely reported in the literature. As such, the incremental advance presented here limits the overall novelty and impact of the work.

Our response:

We understand the reviewer's concern regarding the prevalence of polymer interlayers in the literature; however, we respectfully emphasize that the significance and core novelty of our work extend far beyond conventional or incremental interlayer optimization. Instead, this study delivers a pioneering mechanistic discovery and a targeted, synergistic material design that addresses a critical, long-neglected degradation pathway:

1. Discovery of a hidden degradation mechanism (tin-ion Migration): While prior studies primarily focus on general defect passivation or energy level alignment using polymers, our work reveals for the first time that tin ions from the SnO_2 electron transport layer (ETL) migrate into the perovskite active layer under operational stress, directly catalyzing chemical decomposition and structural degradation at the buried interface. We have provided comprehensive and direct physical evidence for this phenomenon using cross-sectional STEM-EDS (**Fig. 3a, 3b**), quantitative ICP-AES (**Fig. 3f**) and Sn K-edge XANES (**Fig. 3g**). Uncovering this detrimental pathway fundamentally shifts the understanding of buried interface instability.

2. Synergistic and Targeted Polymer Design: Based on this newly uncovered mechanism, the introduced polymer interlayers, particularly PED, were not chosen randomly but rationally engineered to possess dual functionality. The dual-carbonyl groups in PED enable a unique bidentate-like chelation that simultaneously: (1) Reinforces the buried interface: Eliminates interfacial voids and grain boundary grooves while reducing trap density, leading to prolonged carrier lifetimes. (2) Suppresses tin migration: Establishes a remarkably high energy barrier (0.54 eV via DFT calculations and confirmed by an elevated experimental activation energy of 0.402 eV) that effectively blocks tin ion diffusion and prevents its irreversible oxidation within the perovskite matrix.

3. Breakthrough Performance and Scalability: Driven by this synergistic mechanism, our devices achieve a certified power conversion efficiency (PCE) of 26.58% (one of the highest reported to date) and an exceptional operational stability, retaining 96% of the initial PCE after 2000 hours of continuous MPPT testing. Furthermore, this strategy shows excellent scalability, enabling large-area modules (21.54 cm^2) to achieve a certified PCE of 24.17%.

In summary, we do not present an incremental polymer treatment, but rather a vital mechanistic revelation regarding tin-mediated degradation along with a highly effective, dual-functional strategy to mitigate it. We believe these findings provide indispensable insights into the intrinsic stabilization of perovskite photovoltaics, representing a substantial contribution to the field.

Second, although the devices demonstrate respectable efficiencies (up to 26.58% reverse scan / 25.64% forward scan for small-area devices), the use of Spiro-MeOTAD as the hole transport layer raises concerns regarding long-term stability and cost. Notably, the stability measurements are conducted using a different HTL system (polyTPD-based), making it difficult to directly correlate

efficiency and stability within the same device architecture. Furthermore, the stability data (Figure 4e, f) are presented only as normalised efficiency without reporting absolute values, which limits the assessment of real performance degradation.

Our response:

We thank the reviewer for this important comment. We agree that the use of different HTLs for efficiency and stability measurements should be clearly explained, and that absolute PCE values should be provided in addition to normalized stability traces.

In the original manuscript, spiro-OMeTAD was used for the highest-efficiency small-area devices because it remains a widely used HTL for achieving high certified efficiencies in n–i–p perovskite solar cells. In contrast, PTAA-based devices were used for the long-term stability measurements in **Fig. 4e**, **f** to minimize the well-known instability contributions from doped spiro-OMeTAD and to better isolate the effect of the buried SnO_2/perovskite interface. However, we agree that this distinction was not sufficiently clear in the original manuscript.

To directly address the reviewer's concern, we have added an additional stability test using the same spiro-OMeTAD HTL architecture as that used for the high-efficiency devices. The new data are included in **Fig. R10** and we also added it to the supplementary information in **Supplementary Fig. 38**, showing the thermal-light stability of devices with spiro-OMeTAD as the HTL under one-sun illumination at 65 °C. The PED-modified device maintains the highest absolute PCE throughout the test, while the reference device rapidly degrades, confirming that the stabilizing effect of the PED interlayer is also observed in the spiro-OMeTAD-based architecture.

In addition, we revised the captions of **Fig. 4e** and **4f** to explicitly report the initial absolute PCE values of the PTAA-based stability devices. For the MPPT stability test in **Fig. 4e**, the initial PCEs of the pristine reference device, devices with PED/PEM/PVP interlayer were 21.88%, 24.89%, 24.08%, and 23.58%, respectively. For the thermal-light stability test in **Fig. 4f**, the initial PCEs of the pristine reference device, devices with PED/PEM/PVP interlayer were 22.57%, 25.28%, 24.31%, and 23.88%, respectively. These values allow direct assessment of the absolute device performance at the beginning of the stability tests, while the normalized traces show the relative degradation behavior during aging.

Accordingly, the revised manuscript now clarifies the role of PTAA-based stability measurements as a platform to evaluate buried-interface stabilization with reduced interference from doped spiro-OMeTAD degradation. Meanwhile, the newly added spiro-OMeTAD-based stability test confirms that the PED interlayer also improves stability in the same HTL architecture used for high-efficiency device operation.

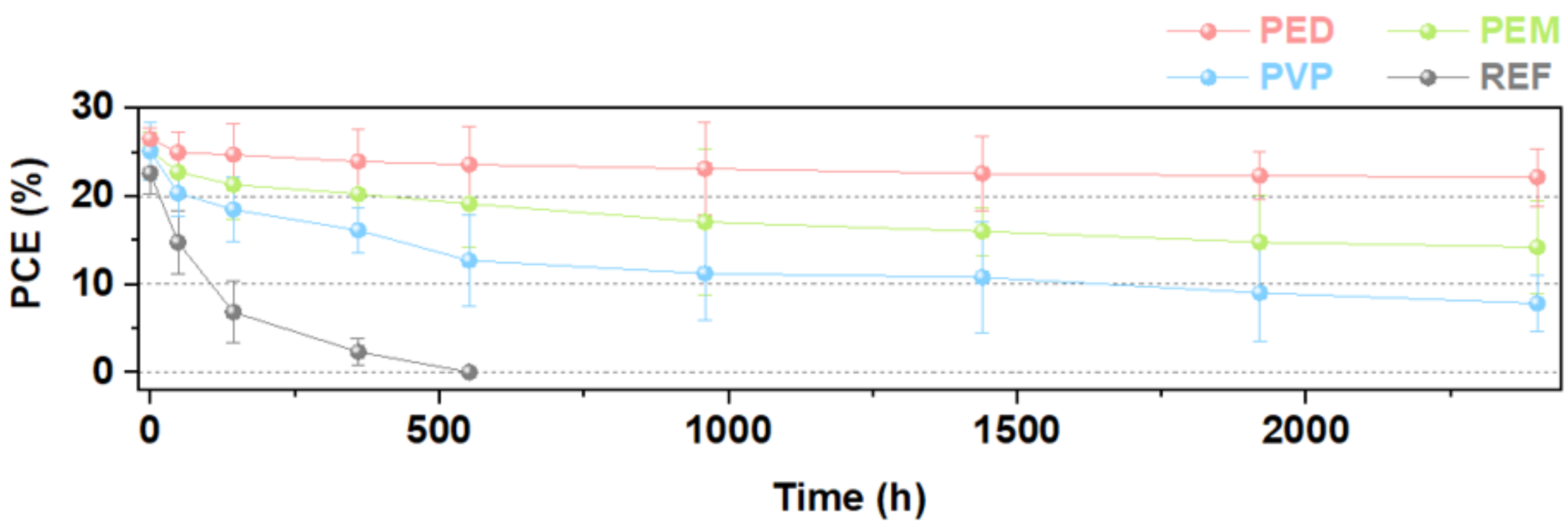

Fig. R10. 65 °C thermal-light stability test under one-sun illumination without or with the polymeric interlayers with spiro-OMeTAD as HTL.

Revised parts in the manuscript:

In page 24 and 25 of the revised manuscript:

(e) MPPT stability test of PTAA-based devices under one-sun illumination at 65 °C with or without polymer interlayers. The initial PCEs of the reference, PED, PEM, and PVP devices were 21.88%, 24.89%, 24.08%, and 23.58%, respectively. **(f)** Thermal-light stability test of PTAA-based devices under one-sun illumination at 85 °C with or without polymer interlayers. The initial PCEs of the reference, PED, PEM, and PVP devices were 22.57%, 25.28%, 24.31%, and 23.88%, respectively.

Third, the devices exhibit non-negligible hysteresis. Therefore, stabilised power output (maximum power point tracking) should be reported instead of relying primarily on reverse scan efficiencies.

Our response:

We thank the reviewer for this comment. We agree that, given the presence of *J*–*V* hysteresis in perovskite solar cells, stabilized power output is essential for accurately evaluating device

performance. We would like to clarify that our performance evaluation does not rely solely on reverse-scan efficiencies. In addition to forward and reverse *J–V* scans, we have reported stabilized power output under maximum power point tracking.

We would like to clarify that the device performance in our manuscript was not evaluated solely based on reverse-scan efficiencies. In the original manuscript, we already reported the stabilized power output under maximum power point tracking in **Fig. 4b**. The PED-modified device shows a stabilized PCE of 26.2%, which is consistent with the J–V and certified efficiency results, confirming that the high device performance is not an artifact of reverse-scan measurement. We have also added/clarified the forward/reverse *J–V* statistics and hysteresis-index analysis in **Fig. R7** and **Table R2**. These data show that PED-modified devices maintain high performance under both scan directions and exhibit the lowest average hysteresis index among all device groups.

To avoid possible ambiguity, we have revised the relevant text and figure caption to explicitly describe **Fig. 4b** as "stabilized power output measured under maximum power point tracking." In addition, we have added statistical forward/reverse *J–V* measurements and hysteresis-index analysis for all device conditions in **Supplementary Fig. 33** and **Supplementary Table 6** of the revised manuscript.

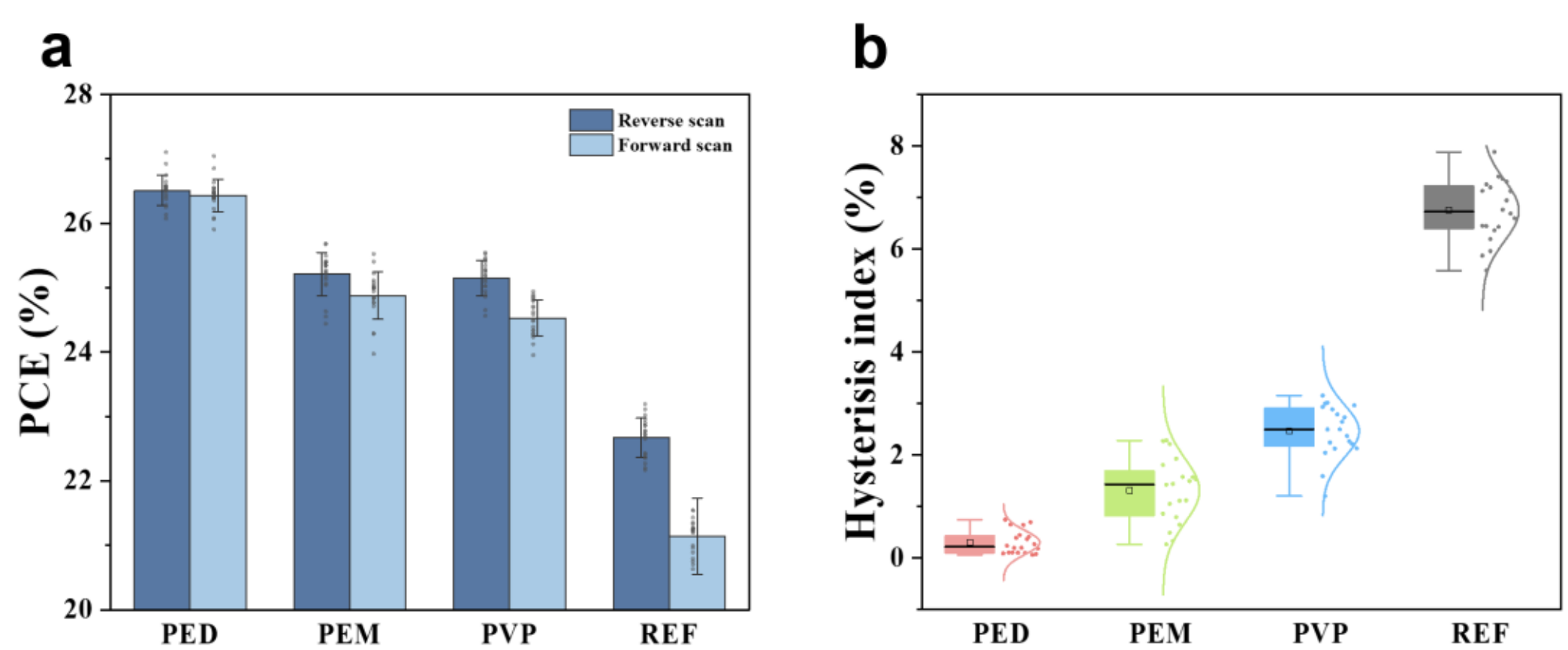

Fig. R7. (a) Statistical comparison of reverse- and forward-scan PCEs for PED-, PEM-, PVP-modified and reference devices. Bars represent mean values, error bars represent standard deviation, and overlaid points represent individual devices (n = 20). (b) Distribution of hysteresis index calculated from the same devices using $HI = (PCE_{\mathrm{reverse}} - PCE_{\mathrm{forward}})/PCE_{\mathrm{reverse}} \times$

100%. Box-plots show the median and interquartile range, and overlaid points represent individual devices.

Table R2. Statistical summary of forward/reverse J–V measurements and hysteresis indices.

Condtion	Reverse PCE (%)	Forward PCE (%)	Hysteresis Index
PED	26.51±0.24	26.43±0.25	0.302±0.228
PEM	25.21±0.34	24.88±0.37	1.309±0.622
PVP	25.15±0.27	24.53±0.28	2.465±0.505
REF	22.67±0.31	21.14±0.59	6.747±0.594

Revised parts in the manuscript:

In page 24 of the revised manuscript:

Fig. 4 | (a) *J–V* curves of devices with and without polymeric interlayers. **(b)** stabilized power output measured under maximum power point tracking.

Reviewer 4:

This work employs a polymer interlayer to modify the interface between SnO2 and perovskite, achieving excellent device performance and stability, with scalable validation demonstrated in large-area solar cells. However, I believe the manuscript requires additional characterization to address gaps in the experimental procedure and strengthen the logical consistency. The authors should resolve the following issues to meet the high standards of Nature Communications, which are currently not fully satisfied.

Our response:

We thank the reviewer for the careful evaluation of our manuscript and for recognizing the excellent device performance, stability, and scalable validation of our polymer-interlayer strategy. We also appreciate the reviewer's constructive comments regarding the need for additional characterization, clearer experimental details, and stronger logical consistency. In the revised manuscript, we have carefully addressed all the points raised by the reviewer by adding polymer-property characterizations, conductivity measurements, concentration and spin-speed optimization, GPC, ATR-FTIR, ^{1}H NMR, AFM scratch step-height analysis, solubility information, depth-profile XPS analysis, hysteresis statistics, and additional experimental details. We have also revised the terminology and presentation throughout the manuscript to improve clarity and consistency. Detailed point-by-point responses are provided below.

1.Are these polymers n-type? Their electrical conductivity must be experimentally verified. A thorough discussion and characterization of the polymer properties are also required.

Our response:

We thank the reviewer for this important comment. We clarify that PED, PEM, and PVP are not assigned as n-type semiconductors in this work. The polymer layers are used as ultrathin interfacial modifiers at the SnO_2/perovskite buried interface, while SnO_2 remains the primary electron-transport layer. Electrical conductivity alone does not determine carrier type; assigning n-type behavior would require carrier-type-specific measurements such as Hall, Seebeck, field-effect, or Mott–Schottky-type characterization. Therefore, we do not claim that these polymers are n-type materials.

To experimentally verify the electrical response of the polymers, we performed two-terminal vertical *I–V* measurements on polymer-only films prepared under the optimized processing conditions. The device structure is schematically shown in **Fig. R11a**. The low-bias linear region from −0.2 to 0.2 V was fitted to extract the slope d*J*/d*V*, and the area-specific resistance was calculated as $R_A = 1/(dJ/dV)$, where *J* is in A cm^{-2}. The effective electrode area was 0.15 cm^2. The measured current was normalized by this effective area to obtain the current density. The apparent vertical conductivity was then estimated using $\sigma_{app} = L/R_A$, where *L* is the effective polymer thickness. Because the absolute thickness of these ultrathin polymer layers is difficult to determine accurately as we will later discuss in the comment 3-3, we used an estimated effective thickness of approximately 5 nm for this calculation. Based on this estimation, the apparent vertical conductivities of PED, PEM, and PVP are 2.78×10^{-7} S cm^{-1}, 3.22×10^{-7} S cm^{-1}, and 2.23×10^{-7} S cm^{-1}, respectively (**Fig. R11b,c,d and Table R3**). These values should be interpreted as apparent vertical conductivities rather than intrinsic bulk conductivities, because the films are ultrathin and the measurement can be affected by nanoscale thickness variation, substrate roughness, contact resistance, and electrode/polymer/substrate interfacial contributions.

Nevertheless, the measurable vertical current confirms that the optimized polymer layers do not behave as thick insulating barriers. This conclusion is further supported by the AFM scratch step-height analysis discussed below in our response to comment 3-3, which shows that the polymer interlayers are ultrathin and do not form thick overlayers on SnO_2. In addition, the UPS characterization discussed below in our response to comment 10 indicates that the polymers modify the surface electronic structure of SnO_2, but does not provide evidence to assign them as n-type transport materials. Therefore, these polymers function primarily as ultrathin, electrically compatible interfacial modifiers rather than independent n-type charge-transport layers.

Accordingly, we have added the vertical *I–V* data, apparent-conductivity calculation, and the corresponding clarification of the polymer role to the revised Supplementary Information in **Supplementary Fig. 44** and **Supplementary Table 10**. We have also revised the related discussion in the main text to avoid the phrase "enhanced n-type character" and to clarify that the UPS results indicate surface electronic modification of SnO_2 rather than n-type behavior of the polymer interlayers.

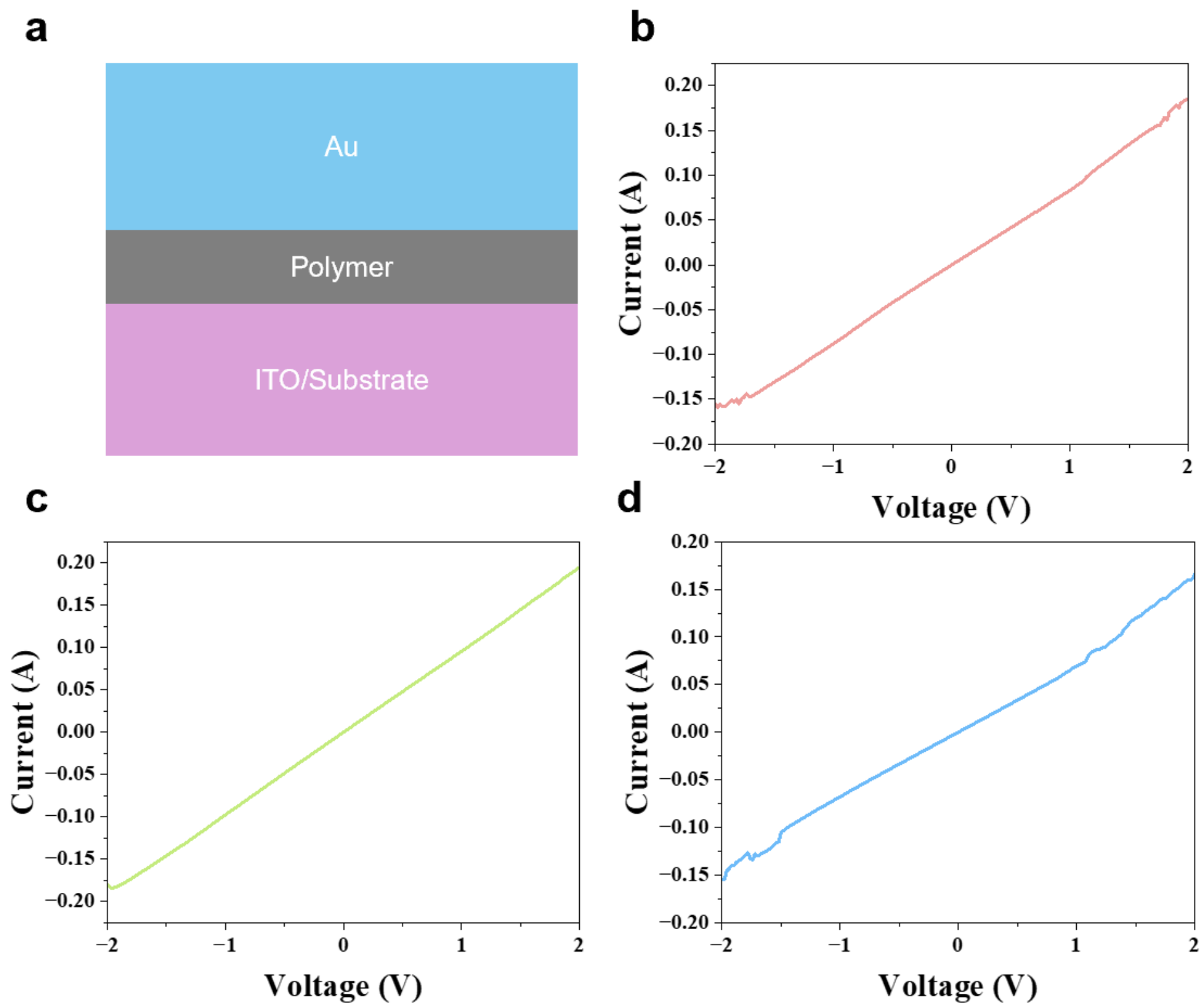

Fig. R11. Vertical electrical characterization of ultrathin polymer layers. (a) Schematic illustration of the two-terminal vertical device structure used for electrical characterization of polymer films. (b) to (d), Vertical current–voltage characteristics of (b) PED, (c) PEM, and (d) PVP films prepared under the optimized processing conditions.

Table R3. Apparent vertical conductivity of ultrathin polymer layers.

Condition	d*J*/d*V* (A cm^{-2} V^{-1})	R_A (Ω cm²)	σ (S cm^{-1})
PED	0.555	1.80	2.78×10^{-7}
PEM	0.643	1.55	3.22×10^{-7}
PVP	0.447	2.24	2.23×10^{-7}

Revised parts in the manuscript:

In page 9, 10 of the revised manuscripts:

As shown in **Supplementary Figures 35** and **Supplementary Table 8**, the PED-modified SnO_2 exhibited a shallower work function compared with pristine SnO_2, while the valence-band onset remained nearly unchanged. These results indicate that PED modifies the surface electronic structure of SnO_2 without substantially changing its occupied-state onset. Such interfacial energy modulation is expected to improve energetic compatibility at the SnO_2/perovskite interface, contributing to more efficient electron extraction and reduced interfacial recombination.

2.How did the authors determine the optimal concentration of the polymer interlayer? Was it based on device efficiency, carrier extraction, or defect passivation capability? No relevant discussion or investigation is provided throughout the manuscript. How do different concentrations influence interfacial charge extraction, defect passivation, and device performance? The authors should present a scientific screening process and justification for the polymer interlayer concentration, rather than arbitrarily fixing it at 1 mg/mL. Such logical rigor is expected for Nature Communications.

Our response:

We thank the reviewer for this important comment. We agree that the selection of the polymer-interlayer concentration should be justified more clearly. In the revised manuscript, we have added concentration-dependent device screening of the PED interlayer in **Fig. R12** and **Table R4**.

PED was used for concentration-dependent screening because it gave the best overall device performance and buried-interface stabilization among the tested polymers. Devices were prepared with PED concentrations of 0.1, 0.2, 0.5, 1, 2, 5, and 10 mg mL^{-1}, while keeping the other device-fabrication conditions unchanged. The representative *J–V* curves and corresponding photovoltaic parameters show a clear concentration-dependent trend rather than an arbitrary selection of 1 mg mL^{-1}.

At lower PED concentrations, the devices showed lower photovoltaic performance. For example, the reverse-scan PCEs of devices prepared with 0.1, 0.2, and 0.5 mg mL^{-1} PED were 22.85%, 22.95%, and 23.85%, respectively. These lower efficiencies were accompanied by lower V_{oc} and FF, suggesting that insufficient polymer coverage may provide incomplete interfacial modification/passivation at the buried SnO_2/perovskite interface.

When the PED concentration was increased to 1 mg mL⁻¹, the device exhibited the highest representative performance, with reverse-scan values of 1.190 V for V_{oc}, 26.41 mA cm⁻² for J_{sc}, 84.14% for FF, and 26.45% for PCE. The corresponding forward-scan PCE was also very similar (26.42%), indicating efficient charge extraction and low scan-direction dependence under this optimized condition.

In contrast, further increasing the PED concentration led to a pronounced decrease in performance. The reverse-scan PCE decreased to 22.01%, 20.65%, and 16.57% for 2, 5, and 10 mg mL⁻¹ PED, respectively. This degradation was accompanied by reduced J_{sc} and FF, indicating that excessive polymer loading may form a thicker or less favorable interfacial layer, which can disturb charge extraction and/or perovskite film formation.

Therefore, the optimized concentration of 1 mg mL⁻¹ PED was selected because it provides the best balance between buried-interface modification/passivation and charge extraction, as reflected by the simultaneous improvement in V_{oc}, J_{sc}, FF, and PCE. PED was used as the representative polymer for concentration-dependent optimization because it showed the best overall device performance and buried-interface stabilization. After identifying 1 mg mL⁻¹ as the optimized PED concentration, the same concentration was applied to PEM and PVP to ensure a fair comparison under identical processing conditions. We have added this discussion to the revised manuscript and the corresponding supporting data to the Supplementary Information in **Supplementary Fig. 34** and **Supplementary Table 7**.

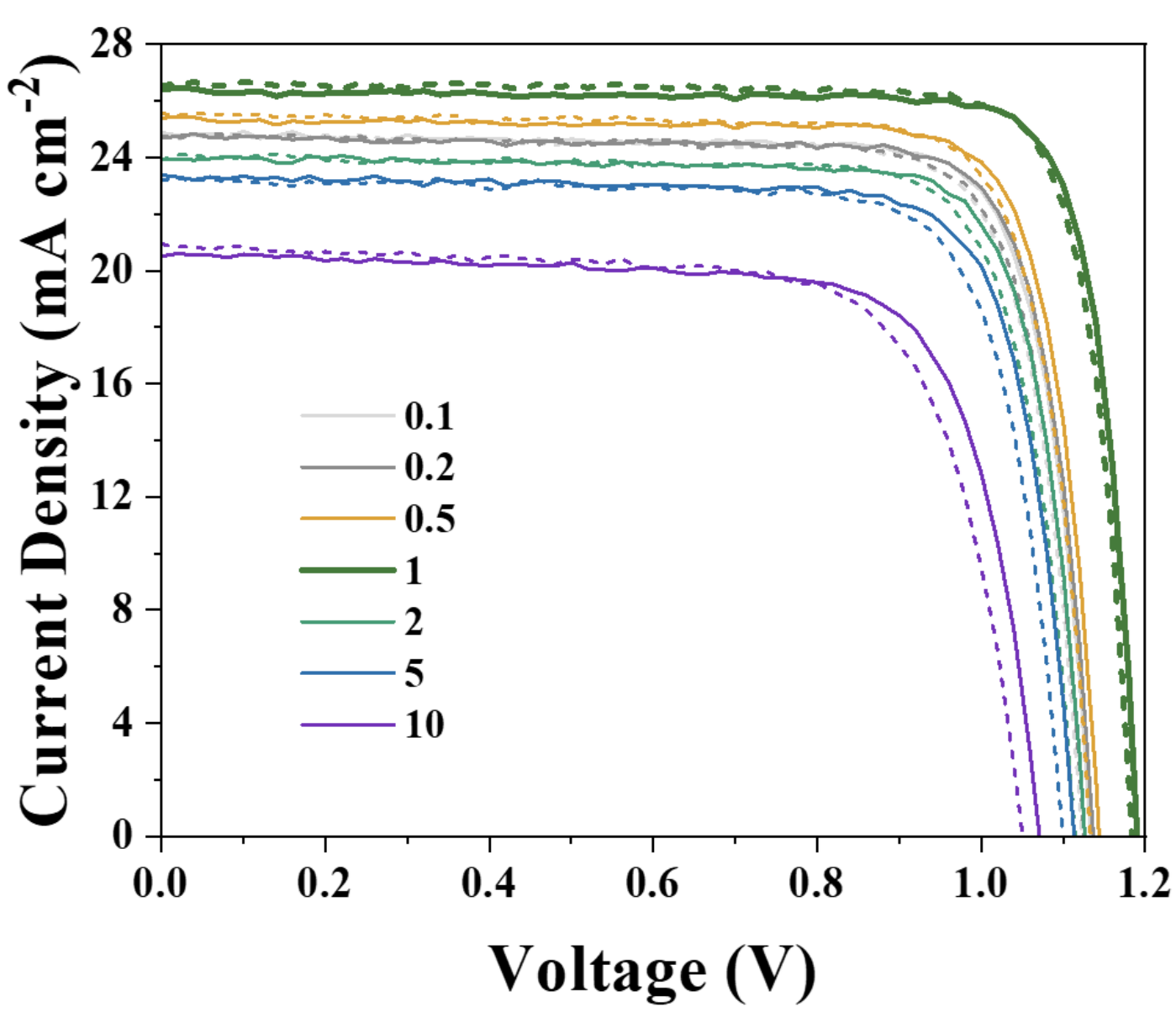

Fig. R12. Concentration-dependent (mg/ml) device performance of PED-modified devices.

Table R4. Photovoltaic parameters of representative PED-modified devices prepared with different PED concentrations.

Condition	V_{oc} (V)	J_{sc} (mA/cm^2)	FF (%)	Eff (%)
0.1 mg/ml F	1.123	24.82	80.52	22.44
0.1 mg/ml R	1.134	24.88	81.04	22.85
0.2 mg/ml F	1.125	24.75	81.24	22.63
0.2 mg/ml R	1.137	24.69	81.77	22.95
0.5 mg/ml F	1.133	25.56	81.49	23.61
0.5 mg/ml R	1.144	25.40	82.09	23.85
1 mg/ml F	1.183	26.56	84.07	26.42
1 mg/ml R	1.190	26.41	84.14	26.45
2 mg/ml F	1.114	23.92	80.77	21.53
2 mg/ml R	1.126	23.95	81.61	22.01
5 mg/ml F	1.100	23.22	78.76	20.11
5 mg/ml R	1.113	23.38	79.31	20.65
10 mg/ml F	1.051	20.94	72.73	16.01
10 mg/ml R	1.071	20.51	75.42	16.57

Revised parts in the manuscript:

In page 12, 13 of the revised manuscripts:

The PED concentration was optimized through concentration-dependent device screening from 0.1 to 10 mg mL^{-1}, with 1 mg mL^{-1} providing the best representative photovoltaic performance and the most balanced improvement in V_{oc}, J_{sc}, and FF (**Supplementary Fig. 34** and **Supplementary Table 7**). PED was used as the representative polymer for concentration-dependent optimization because it showed the best overall device performance and buried-interface stabilization. After identifying 1 mg mL^{-1} as the optimized PED concentration, the same concentration was applied to PEM and PVP to ensure a fair comparison under identical processing conditions.

3.The authors pre-synthesized monomers into polymers before device fabrication. I have several questions regarding this procedure:

1)How did the authors aim for 80 °C as the optimal polymerization temperature? AIBN initiates radical decomposition at approximately 70 °C. Supporting evidence must be provided.

Our response:

We greatly appreciate the reviewer for pointing out this. The polymerization temperature of 80°C was selected based on the well-established decomposition kinetics of AIBN ($t_{1/2}$ ≈ 75 min at 80°C)[10], which provides a steady radical flux suitable for efficient chain initiation while maintaining controllable reaction conditions. This temperature is widely adopted in conventional free-radical polymerization with AIBN as the initiator.

2)To what extent does polymerization proceed under stirring overnight at 80 °C? Is the reaction complete or incomplete? A specific optimal polymerization time should be identified, rather than using the vague term "stirred overnight". The investigation and supporting data for these points should be included.

Our response:

We thank the reviewer for this important comment. We agree that the polymerization condition should be described quantitatively rather than using the vague term "stirred overnight". In the revised supplementary information, we have specified the exact polymerization condition as stirring at 80 °C for 14 h.

This polymerization time was selected based on the decomposition kinetics of AIBN[10]. At 80 °C, 14 h corresponds to approximately 11 half-lives of AIBN, leading to near-complete decomposition of the initiator, with the residual AIBN concentration estimated to be below 0.05%. Therefore, after this reaction period, the radical source is essentially depleted, and further chain initiation is not expected to proceed effectively. Accordingly, 14 h at 80 °C was used as the defined polymerization condition for preparing the polymer interlayers, rather than an unspecified "overnight" process.

We also clarify that the isolated yield should not be interpreted as the monomer conversion. After polymerization, the crude polymer products were purified through three dissolution–reprecipitation cycles to remove residual monomers, initiator fragments, and low-molecular-weight oligomers. Specifically, $CHCl_3$/diethyl ether was used for PED purification, while

chlorobenzene/diethyl ether was used for PEM purification. The isolated yield of purified PED, PEM after three dissolution–reprecipitation cycles was 52.6%, 57.1%, respectively, which reflects the recovery of the purified polymer fraction after removal of soluble residual species and low-molecular-weight components.

To further support the formation of purified polymer products, we added gel permeation chromatography (GPC) characterization to the revised Supplementary Information. As shown in Supplementary Figs. 3 and 5, purified PED exhibits Mn = 5,033, Mw = 7,465, and Đ = Mw/Mn = 1.48 (**Fig. R13**). Because PEM has relatively limited solubility, we optimized the sample preparation condition and re-measured its GPC. The updated PEM GPC chromatogram shows a broader apparent molecular-weight distribution, with Mn = 16,304, Mw = 62,018, and Đ = Mw/Mn = 3.804 (**Fig. R14**). Molecular weights are reported as relative values based on polystyrene calibration. These results support the formation of purified polymeric products after specified polymerization and purification procedures.

Therefore, the revised supplementary now identifies 14 h at 80 °C as the defined polymerization time, provides the rationale based on AIBN decomposition kinetics, and includes the purification procedure, isolated yield, and GPC characterization as supporting evidence. We have revised the reaction scheme section and added the corresponding GPC data to the Supplementary Information in **Supplementary Fig. 3** and **Supplementary Fig. 5**. We also noticed a typo on the amount of AIBN usage and a labeling error in the Supplementary Scheme, where the structures/labels of PED and PEM were inadvertently interchanged. This has been corrected in the revised Supplementary Information.

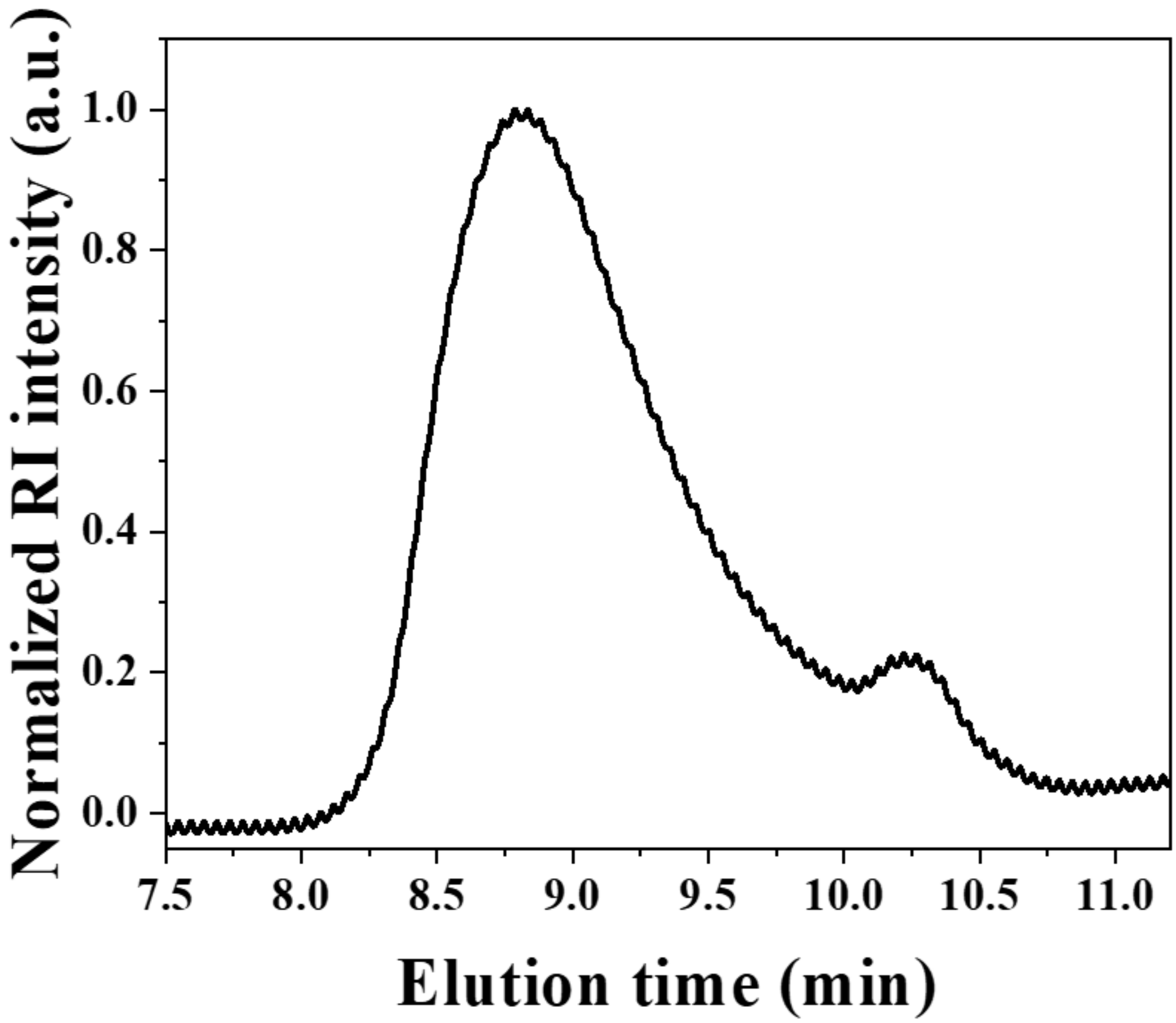

Fig. R13. GPC characterization of purified PED. The chromatogram is dominated by a main polymer peak. The purified PED exhibits Mn = 5,033, Mw = 7,465, and Đ = Mw/Mn = 1.48.

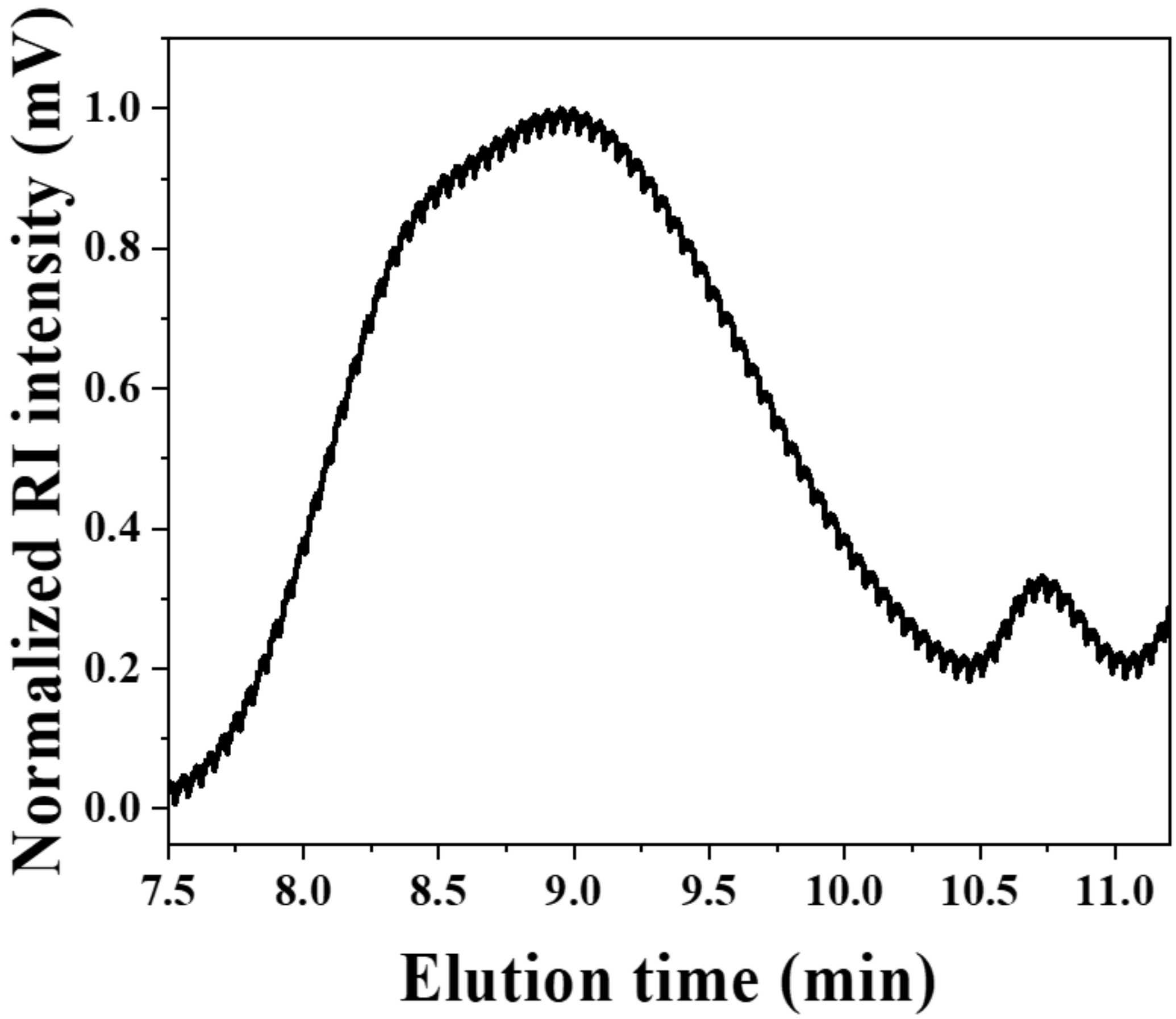

Fig. R14. GPC characterization of purified PEM. The chromatogram is dominated by a main polymer peak. The purified PEM exhibits Mn = 16304, Mw = 62018, and Đ = Mw/Mn = 3.804.

Revised parts in the manuscript:

In page 6 of the revised supplementary information:

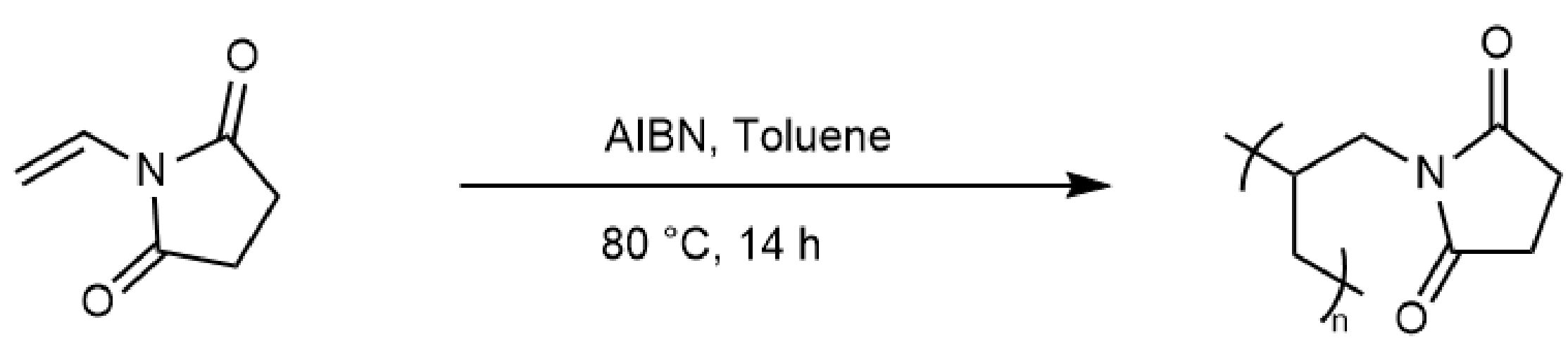

Supplementary Scheme. 1. Synthesis route of PED

Under a nitrogen atmosphere, 1-ethenylpyrrolidine-2,5-dione (ED) (0.2 mL) was dissolved in toluene (1 mL) together with 2,2′-azobis(2-methylpropionitrile) (AIBN, 0.002 ~~m~~g). The solution was heated and stirred at 80 °C for 14 hours to affect free-radical polymerization. To improve the solubility of the product, 1mL chloroform (CF) were added to the solution. The mixture was then poured into diethyl ether (DE) to precipitate the polymer. The resulting precipitated polymer was collected by filtration, purified by three cycles of reprecipitation (dissolution in CF followed by precipitation with DE, and dried under vacuum to constant mass. The PED sample was obtained as flake-like solids (Mn=5033, Mw=7465).

In page 7 of the revised supplementary information:

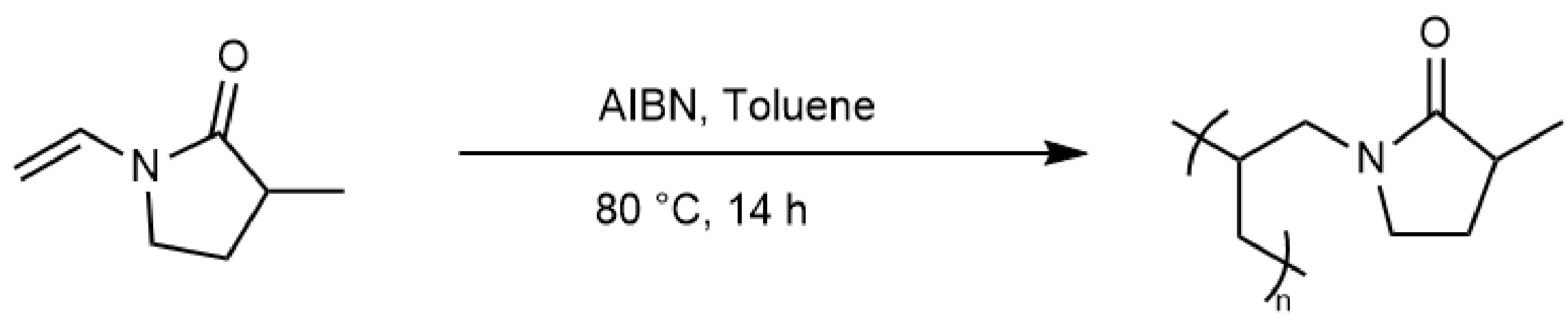

Supplementary Scheme 2. Synthesis route of PEM

Similarly, under a nitrogen atmosphere, 1-ethenyl-3-methylpyrrolidin-2-one (EM) (0.2 mL) was dissolved was dissolved in toluene (1 mL) together with AIBN (0.002 ~~m~~g). The solution was heated and stirred at 80 °C for 14 hours to effect free-radical polymerization. To improve the solubility of the product, 1mL chlorobenzene (CB) was added to the solution. The mixture was then poured into diethyl ether (DE) to precipitate the polymer. The resulting precipitated polymer was collected by filtration, purified by three cycles of reprecipitation (dissolution in CB followed by precipitation with DE, and dried under vacuum to constant mass. The PEM sample appeared as a free-flowing granular powder (Mw=16304, Mn=62018).

3)is the thickness of the polymer layer spin-coated onto SnO2 after stirring overnight at 80 °C? How was the spin condition of 3000 rpm for 30 s determined? Was it based on device performance, carrier extraction efficiency, or defect passivation capability? These details are missing. Please justify the choice of spin speed and establish its correlation with film thickness.

Our response:

We thank the reviewer for this helpful comment. We have now added AFM scratch step-height measurements and spin-speed-dependent device-performance screening to the revised Supplementary Information.

Because the polymer interlayers are ultrathin and deposited on rough SnO_2, direct determination of their absolute thickness is challenging. In addition, mechanical scratching can remove part of the underlying SnO_2 together with the polymer layer. Therefore, pristine SnO_2 was measured as a control, and the AFM results are reported as effective scratch step heights rather than exact absolute polymer thicknesses. The scratch step heights of polymer-modified SnO_2 films are comparable to that of pristine SnO_2, indicating that the polymer modification does not form a thick blocking overlayer (**Fig. R15** and **Fig. R16**).

The spin condition of 3000 rpm for 30 s was selected based primarily on device-performance screening using PED as the representative polymer interlayer. Devices prepared at 3000 rpm showed the highest PCE among the tested spin speeds, whereas both lower and higher spin speeds resulted in lower device performance (**Fig. R17**). This indicates that 3000 rpm provides the best balance between interfacial modification and charge extraction. After this condition was identified for PED, the same spin condition was applied to PEM and PVP to enable a fair comparison of polymer-structure-dependent interfacial effects under identical processing conditions.

We have added this discussion to the revised manuscript and the corresponding supporting data to the Supplementary Information in **Supplementary Fig. 41**, **Supplementary Fig. 42** and **Supplementary Fig. 43**.

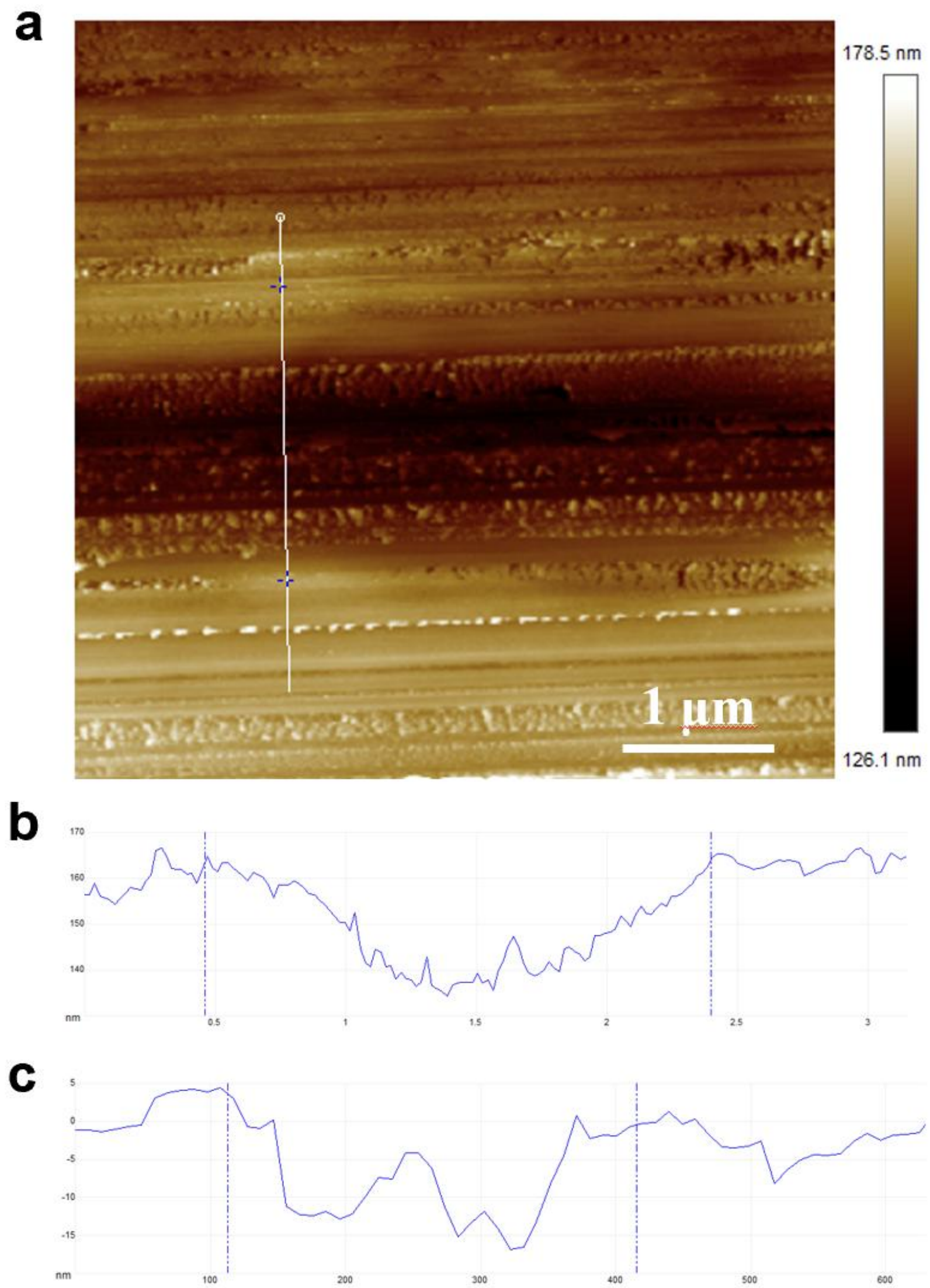

Fig. R15. Representative AFM scratch step-height analysis of PED-modified SnO_2. (a) Representative AFM height image of a mechanically scratched PED-modified SnO_2 film prepared at 3000 rpm. The white line indicates the position used for line-profile analysis. (b) (c) Representative AFM line profiles extracted from PED-modified SnO_2 and pristine SnO_2 control samples. Because mechanical scratching can partially remove the underlying SnO_2 layer together with the polymer overlayer, the measured values are interpreted as effective scratch step heights rather than exact absolute polymer thicknesses.

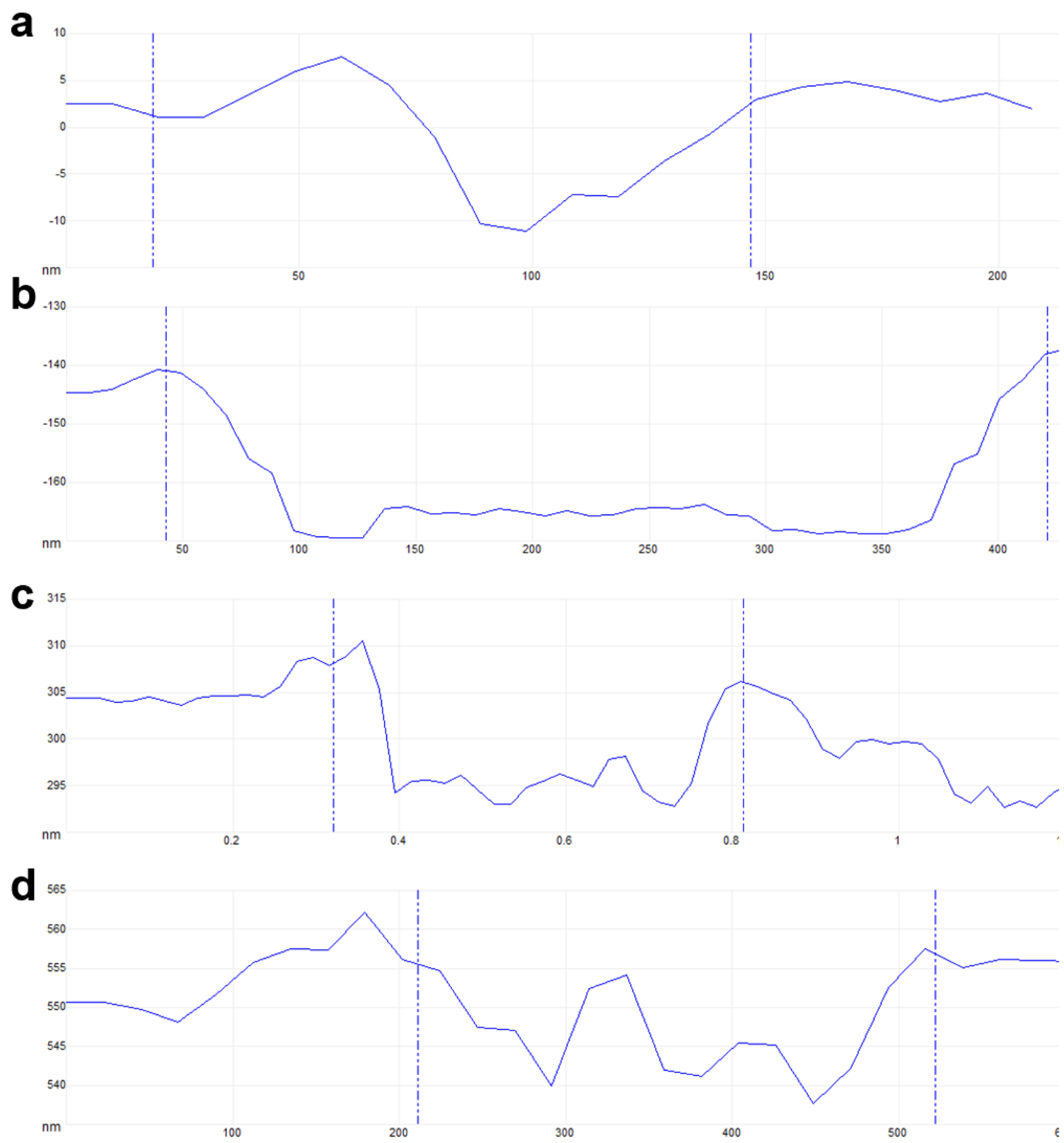

Fig. R16. AFM line-profile comparison of polymer-modified SnO_2 films prepared under different conditions. (a)PEM at 3000 rpm; (b) PVP at 3000 rpm; (c) PED at 500 rpm; (d) PED at 5000 rpm.

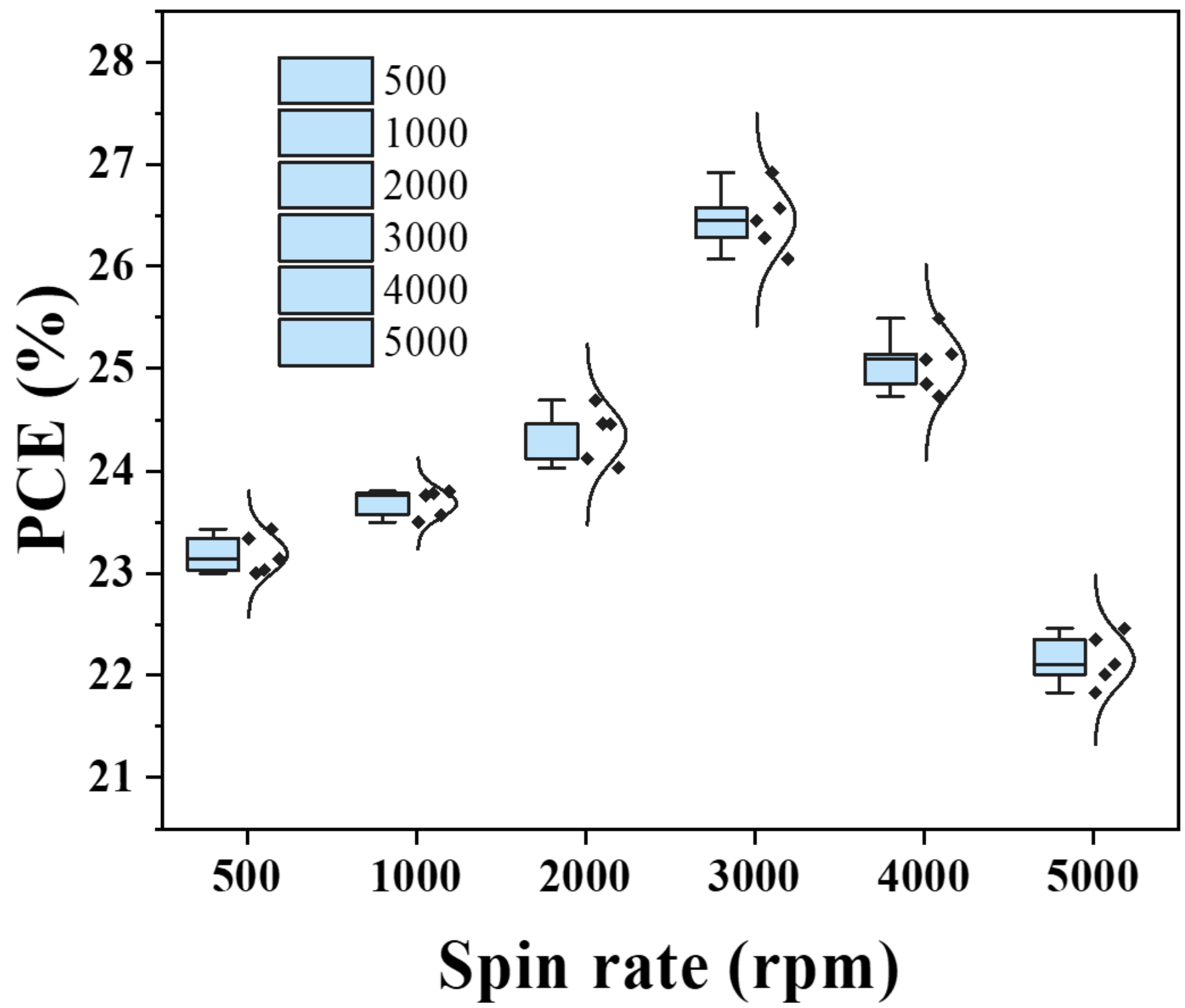

Fig. R17. Spin-speed-dependent device-performance screening of PED-modified devices.

Revised parts in the manuscript:

In page 13 of the revised manuscript:

The solutions were spin-coated at a speed of 3000 r.p.m. for 30 s and annealed at 75°C for 1 minute. This spin-coating condition was selected based on spin-speed-dependent device-performance screening using PED as the representative polymer interlayer (**Supplementary Fig. 43**).

4)Please provide photographs demonstrating the solubility of PED, PEM, and PVP in the solvents used to confirm their solubility.

Our response:

We greatly appreciate the reviewer for pointing out this. Photographs confirming the complete dissolution of PED (in $CHCl_3$), PEM (in chlorobenzene), and PVP (in chlorobenzene) at 1 mg/mL are now provided in **Fig. R18** and **Supplementary Fig. 7**.

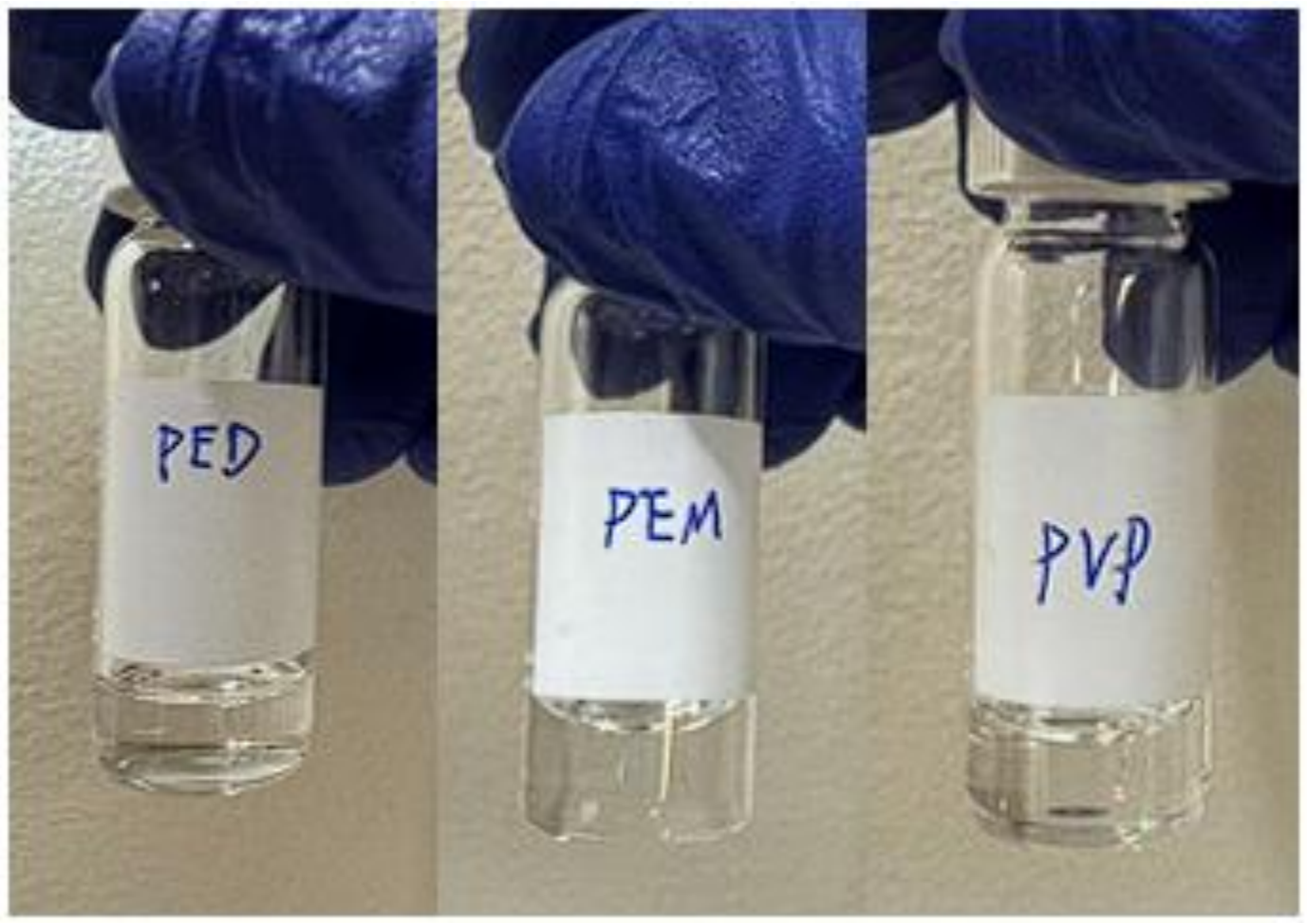

Fig. R18. Solution of 1mg/ml PED, PEM and PVP.

4.During perovskite annealing at 150 °C, does the process affect the polymer interlayer? Could further polymerization occur? What implications does this have on device performance? This requires investigation and supporting evidence.

Our response:

We appreciate the reviewer raising this important concern. We agree that the possible influence of the 150 °C perovskite annealing step on the polymer interlayers should be clarified. We addressed this issue from chemical, thermal-stability, and experimental perspectives.

First, from a chemical reaction standpoint, further radical polymerization during perovskite annealing is unlikely. The free-radical polymerization of the polymers was conducted at 80 °C for 14 h, corresponding to approximately 11 half-lives of AIBN. Under this condition, the residual initiator concentration is estimated to be below 0.05%. In addition, the polymer products were purified by repeated dissolution–reprecipitation cycles, which further removed residual monomers, initiator fragments, and low-molecular-weight species. Therefore, the key requirements for further radical polymerization, namely the simultaneous presence of active radical sources and free monomers, are not expected to exist during the subsequent perovskite annealing step.

Second, the annealing temperature used for perovskite formation is much lower than the typical decomposition temperature of related polymer backbones. Poly(N-substituted maleimide)-type polymers have been reported to exhibit high thermal stability, with no detectable weight loss below 300 °C and maximum decomposition temperatures around 400–440 °C under nitrogen

atmosphere[11]. These temperatures are far above the 150 °C annealing temperature used in our device fabrication process. This suggests that the polymer interlayers are not expected to undergo significant thermal decomposition during perovskite annealing.

Third, we performed direct experimental characterization to verify the structural stability of the polymers under the annealing condition. PED, PEM, and PVP thin films on ITO substrates were thermally treated at 150 °C using the same annealing condition as that used during perovskite film formation, followed by ATR-FTIR characterization. As shown in **Fig. R19**, the main characteristic vibrational features of all three polymers are largely retained after heating, and no obvious new absorption bands associated with thermal decomposition or further chemical reaction are observed. Minor relative-intensity and line-shape variations are visible in parts of the fingerprint region, particularly for PEM, which may arise from local structural rearrangement or changes in intermolecular interactions rather than clear evidence of further polymerization.

Because PED is the optimized polymer interlayer that delivers the best device performance and stability, we further performed ^{1}H NMR characterization on PED before and after 150 °C thermal treatment. As shown in **Fig. R20**, no newly emerged monomer-related vinyl resonances are observed after heating. This result provides no evidence for further polymerization of PED under the perovskite annealing condition.

Taking together, these results indicate that the polymer interlayers are largely preserved during the 150 °C perovskite annealing step. Therefore, the improved device performance is not attributed to uncontrolled in situ polymerization or thermal conversion during annealing. Instead, it originates from the preformed ultrathin polymer interlayer and its interfacial modification effects, including improved buried-interface contact, defect passivation, reinforced interfacial bonding, and suppression of Sn-related ion migration. We have added the FTIR and ^{1}H NMR data to the revised Supplementary Information in **Supplementary Fig. 6** and **Supplementary Fig. 2**. Also we clarified the thermal stability of the polymer interlayers in the revised manuscript.

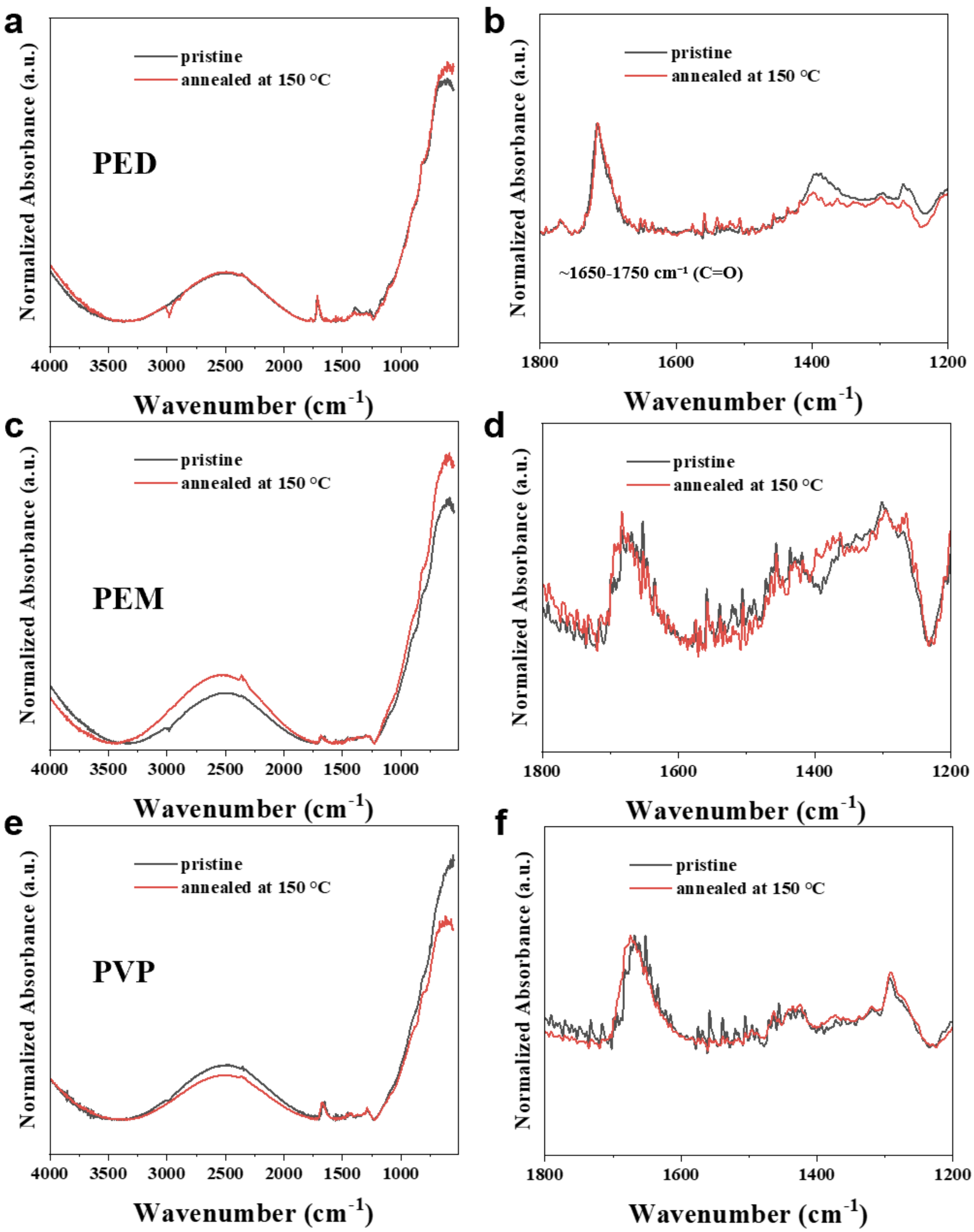

Fig. R19. ATR-FTIR characterization of polymer thermal stability under the perovskite annealing condition. (a), (c), (e) Full-range normalized absorbance FTIR spectra of PED, PEM, and PVP,

respectively, before and after annealing at 150 °C. (b), (d), (f) Corresponding enlarged spectra in the 1800–1200 cm⁻¹ region, covering the carbonyl-related and adjacent fingerprint regions. The spectra were baseline-corrected and normalized for comparison of peak positions and characteristic vibrational features.

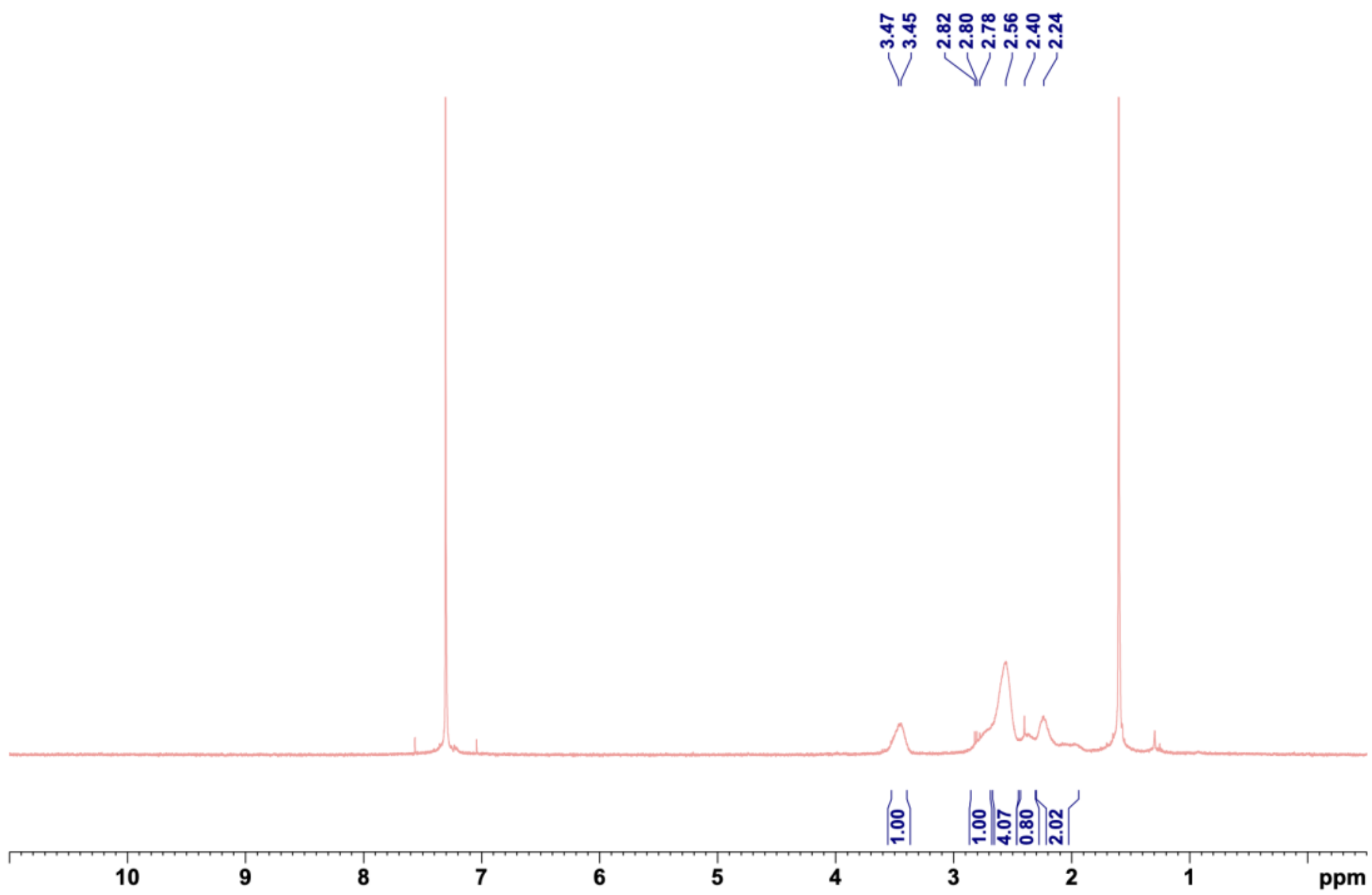

Fig. R20. ^{1}H NMR of PED after annealing at 150°C for 13 minutes.

<u>Revised parts in the manuscript:</u>

In page 13 of the revised manuscript:

This spin-coating condition was selected based on spin-speed-dependent device-performance screening using PED as the representative polymer interlayer (**Supplementary Fig. 43**). Additional ATR-FTIR and ^{1}H NMR characterizations indicate that the polymer interlayers remain largely stable under the 150 °C perovskite annealing condition, with no obvious evidence of thermal decomposition or further polymerization (**Supplementary Figs. 2** and **6**).

In page 16 of the revised manuscript:

FTIR spectra were collected in ATR mode using a Thermo Scientific Nicolet Summit X FTIR spectrometer equipped with an Everest ATR accessory and a ZnSe ATR crystal. The polymer-

coated side was placed in contact with the ATR crystal during measurement. The spectra were plotted as normalized absorbance to compare peak positions and characteristic vibrational features. For thermal-stability characterization, polymer powders were heated at 150 °C for 13 min, matching the perovskite annealing condition used in device fabrication.

5.Please replace the term “bottom interface” with “buried interface”, which is more accurate in this context.

Our response:

We thank the reviewer for this precise suggestion. We completely agree that "buried interface" is the more accurate and standard terminology in this context. Accordingly, we have replaced "bottom interface" with "buried interface" throughout the revised manuscript and supplementary information.

6.The authors conclude that “these results confirm that the bottom interface is significantly more sensitive to light-induced degradation than the top surface”. However, no justification is provided for this conclusion beyond optical microscope observations. Please provide a thorough explanation to support this claim.

Our response:

We thank the reviewer for this helpful comment. We agree that optical microscopy alone is insufficient to conclusively establish that the buried interface is more sensitive to light-induced degradation than the top surface. Therefore, in the revised manuscript, we have softened the wording and replaced the previous statement that “these results confirm that the bottom interface is significantly more sensitive to light-induced degradation than the top surface” with a more conservative description. We now refer to the buried SnO_2/perovskite interface as a major degradation-sensitive region under light aging.

To provide additional justification, we added depth-profile XPS measurements of fresh and aged devices in **Fig. R3** and **Fig. R4**. Compared with the fresh device, the aged device shows pronounced Pb/I/N compositional decoupling near the buried SnO_2/perovskite interface. In part of the buried-interface region, the N-containing organic component is depleted relative to Pb/I-containing inorganic species, suggesting preferential loss or redistribution of the organic

perovskite components during aging. In addition, the aged device exhibits increased overlap between Sn-related signals and perovskite-related Pb/I signals near the buried interface, consistent with Sn-related migration or intermixing from SnO_2 into the perovskite side. These depth-resolved results provide chemical evidence that the buried interface undergoes more pronounced compositional redistribution than the top interface under the same aging condition.

This interpretation is also consistent with our other interface-focused characterizations, including post-aging bottom-side XPS, STEM-EDS, ICP-AES, and Sn K-edge XANES analyses, which collectively support Sn-related ion migration from SnO_2 as an important degradation pathway. Importantly, we do not claim that degradation at the top surface or in the bulk is absent. Rather, the revised manuscript now states that the buried SnO_2/perovskite interface is more strongly affected under the tested light-aging condition and represents a major degradation-sensitive region in these n–i–p devices. We have added the XPS depth-profiling results to the **Supplementary Fig.9** and **Supplementary Fig.10.**

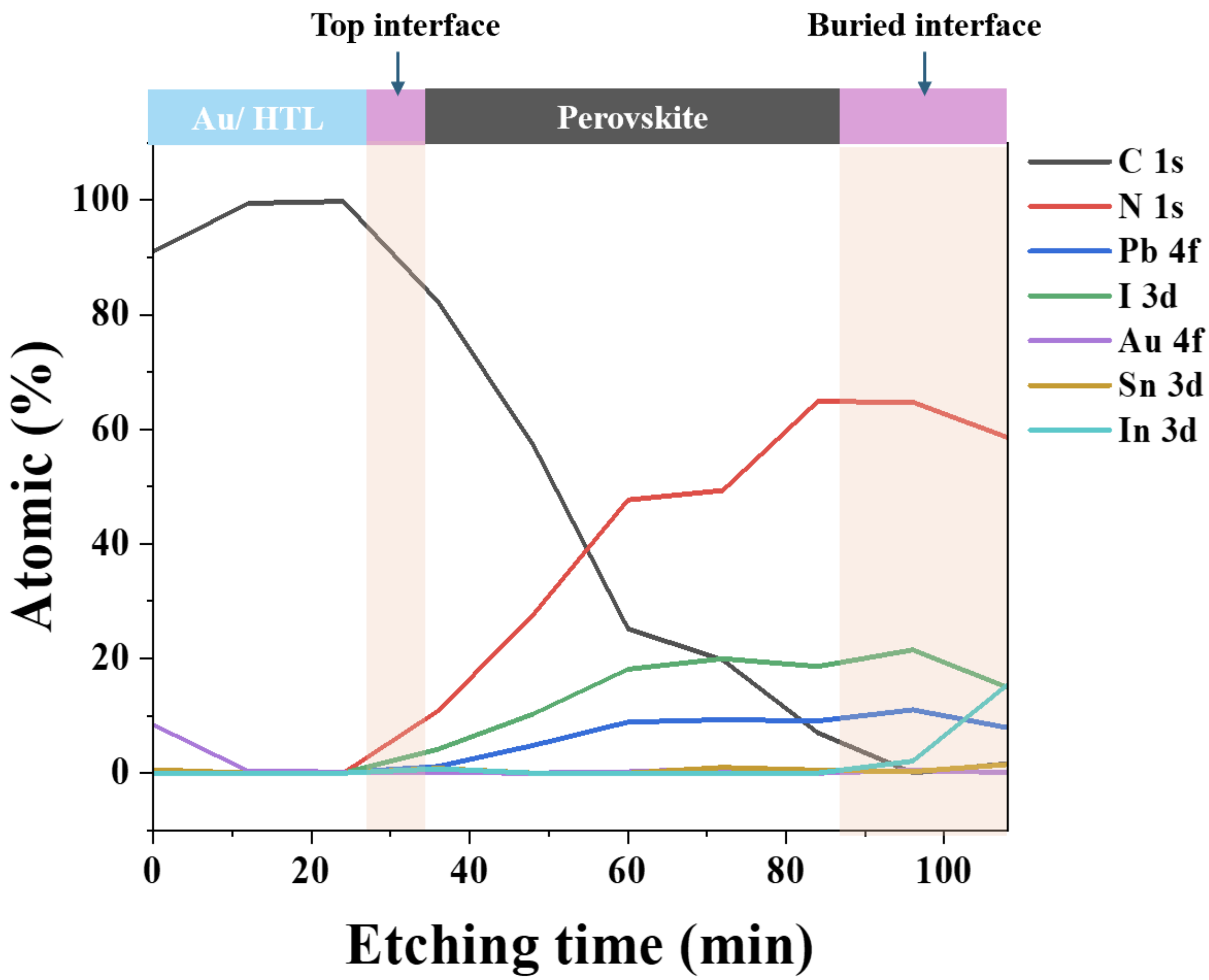

Fig. R3. Interface-focused XPS depth profile of the fresh device.

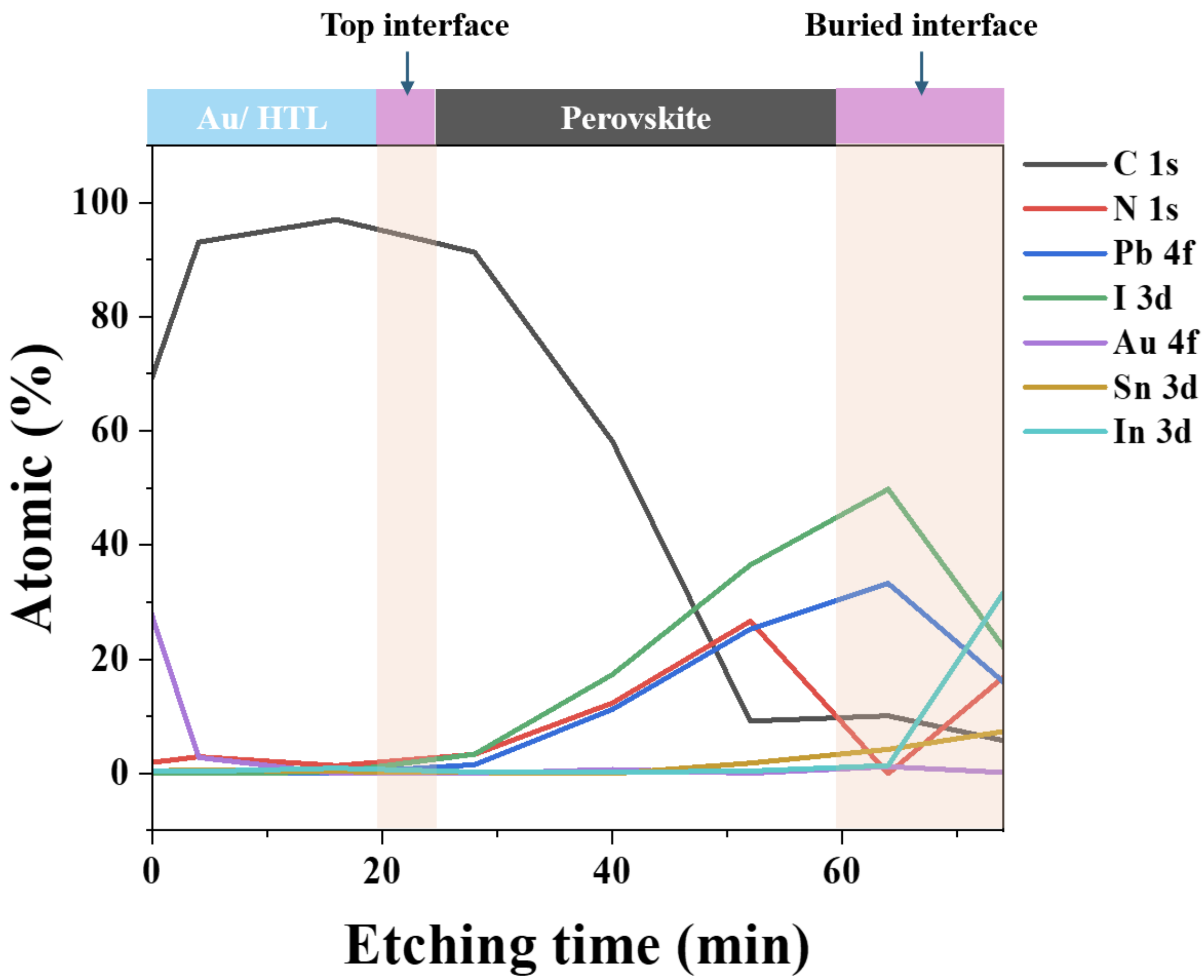

Fig. R4. Interface-focused XPS depth profile of the aged device (aging condition: encapsulated device under one-sun illumination for 170 hours).

Revised parts in the manuscript:

In page 5 of the revised manuscript:

Optical microscopy (OM) images revealed a more pronounced color change at the buried side than at the top surface after aging (**Supplementary Fig. 14**), suggesting stronger degradation near the buried interface. This observation is further supported by depth-profile XPS of fresh and aged devices. Compared with the fresh device, the aged device shows pronounced Pb/I/N compositional decoupling near the buried SnO_2/perovskite interface, where the N-containing organic component is depleted relative to Pb/I-containing inorganic species in part of the buried-interface region (**Supplementary Figs. 9, 10**).

7.Please improve Figure S7 by adding a scale bar, clearly defining the measurement range, and experimental conditions, as the current presentation is disorganized. Please label the conditions and elements in Figures 3a and 3b for greater clarity.

Our response:

We sincerely thank the reviewer for pointing out these oversights.

Supplementary Fig.12 actually displays the buried interface of the fresh devices. It was mistakenly referenced in the wrong section of the manuscript due to a typographical error. We have now relocated it to the appropriate section and revised the figures according to your helpful suggestion.

Additionally, we have updated **Figures 3a and 3b** and incorporated more detailed experimental conditions into the revised manuscript to further improve clarity.

Revised parts in the manuscript:

In page 21 of the revised SI:

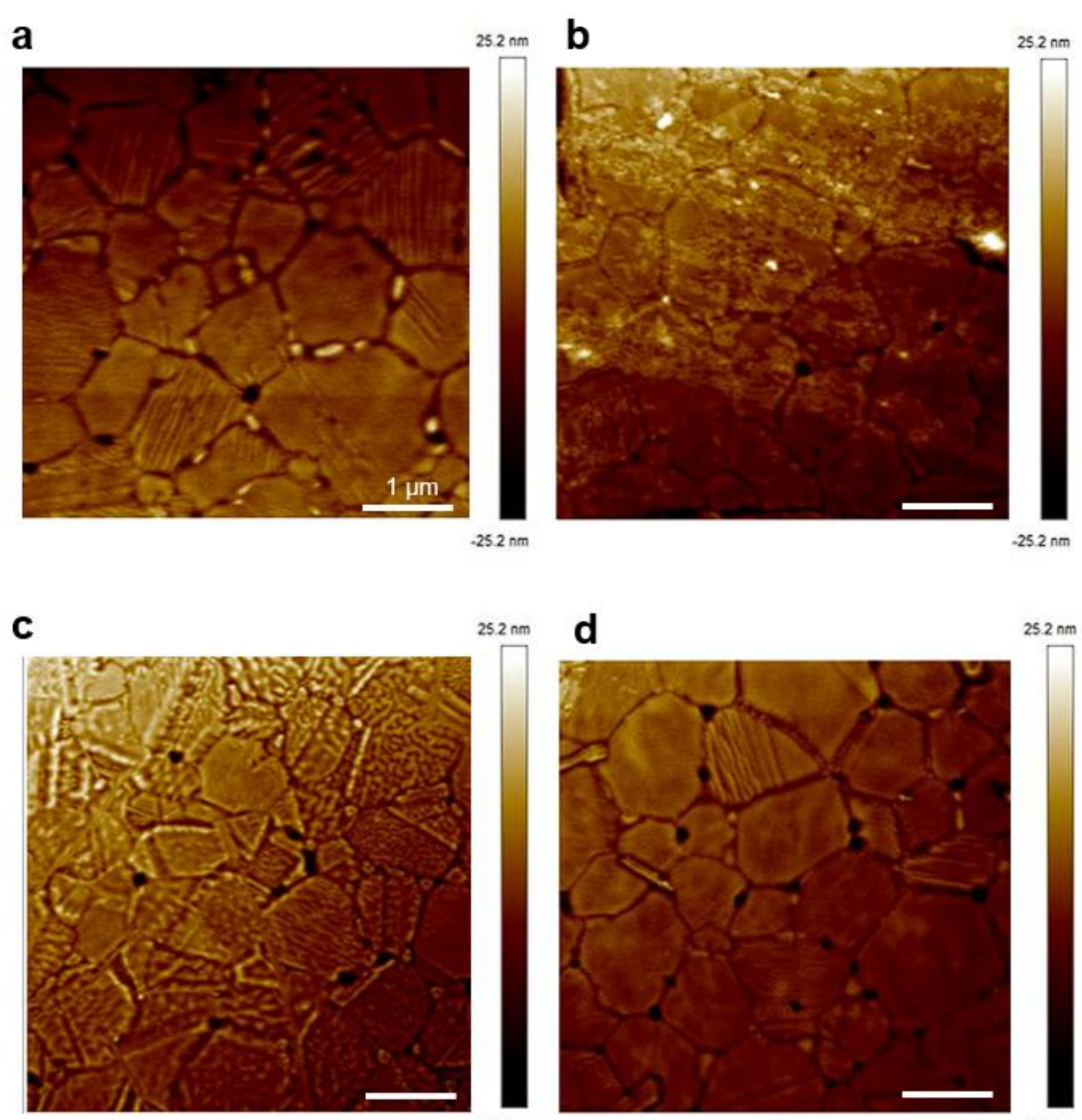

Supplementary Fig. 12. AFM images for buried interface peeled from fresh device of (a) REF (b) with PED interlayer (c) with PEM interlayer(d)with PVP interlayer.

In page 4 of the revised manuscript:

Cross-sectional scanning electron microscopy (SEM) images of the buried interface (**Fig. 1c to f**) revealed that the reference perovskite films contained numerous voids and grooves along grain boundaries. In contrast, perovskite films incorporating polymeric interlayers exhibited denser microstructures with significantly reduced grain boundary grooves, as further confirmed by atomic force microscopy (AFM) (**Supplementary Fig.12**).

In page 5 of the revised manuscript:

The perovskite layers were peeled off after operation under maximum power point tracking (MPPT) for 200 hours. SEM analysis (**Fig. 2a**) showed that the aged perovskite bottom surfaces of reference devices developed pronounced grain boundary grooves and voids, consistent with structural degradation. In contrast, films incorporating polymeric interlayers exhibited smoother, denser morphologies~~, as further confirmed by atomic force microscopy (AFM) (**Supplementary Fig.9**).~~

In **Figure 3a** and **3b** of the revised manuscript:

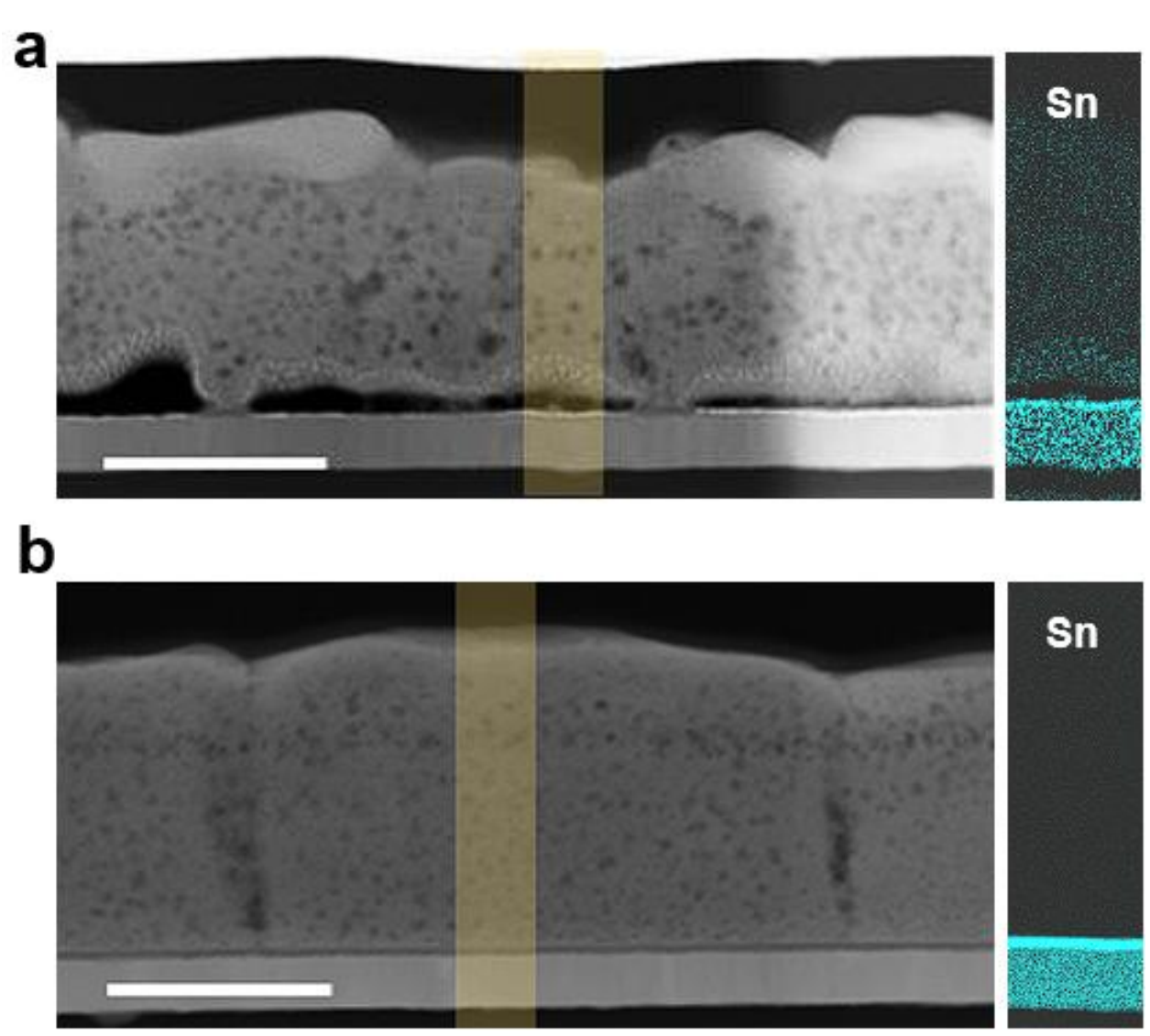

(a) cross-sectional STEM image (scale bar: 500 nm) and tin EDS elemental map of the highlighted area in the reference sample after MPPT aging for 1000 hours. (b) cross-sectional STEM image and tin EDS elemental map of the highlighted area in the PED-treated sample after MPPT aging for 1000 hours.

8.How can the pronounced increase in hysteresis for PED-modified devices be reconciled with the claim that PED effectively suppresses ion migration? Please address this apparent contradiction.

Our response:

We thank the reviewer for pointing out this important issue. We agree that the champion PED device shown in the original *J–V* curve appears to exhibit scan-direction-dependent behavior. However, the J–V curves in Fig. 4a were originally presented to show the champion device performance, rather than to provide a statistical comparison of hysteresis. Since J–V hysteresis in perovskite solar cells can vary from device to device, a single champion curve is not sufficient to quantitatively evaluate hysteresis across different interlayer conditions.

To address this concern, we have now added statistical forward- and reverse-scan J–V analysis based on 20 devices for each condition. As shown in **Fig. R7a**, the PED-modified devices exhibit consistently high PCEs in both reverse and forward scans. More importantly, the hysteresis-index distribution calculated from the same devices shows that PED-modified devices have the lowest average hysteresis index among all device groups, including the reference, PEM-, and PVP-modified devices (**Fig. R7b** and **Table R2**). The hysteresis index was calculated as $HI = (PCE_{\text{reverse}} - PCE_{\text{forward}})/PCE_{\text{reverse}} \times 100\%$). These statistical data indicate that the apparent hysteresis observed in one champion PED device does not represent the overall device trend.

We also note that *J–V* hysteresis is not a direct one-to-one measure of a single ion-migration process. It is affected by multiple coupled factors, including ion migration, trap filling/detrapping, interfacial charge accumulation, capacitive effects, and scan-rate-dependent transient responses. Therefore, the suppression of Sn-related ion migration by PED should be evaluated together with more direct ion-migration and interfacial-degradation evidence. In this work, PED-induced suppression of ion migration is supported by temperature-dependent conductivity, resistive-switching measurements, STEM/EDS, ICP-AES, XPS, and XANES analyses. The newly added statistical hysteresis analysis is consistent with these independent measurements and further confirms that PED does not aggravate J–V hysteresis at the device-statistics level.

We have revised the manuscript and Supplementary Information accordingly by adding the statistical forward/reverse *J–V* data, hysteresis-index distribution, and the definition of the hysteresis index to the **Supplementary Fig. 33** and **Supplementary Table 6**.

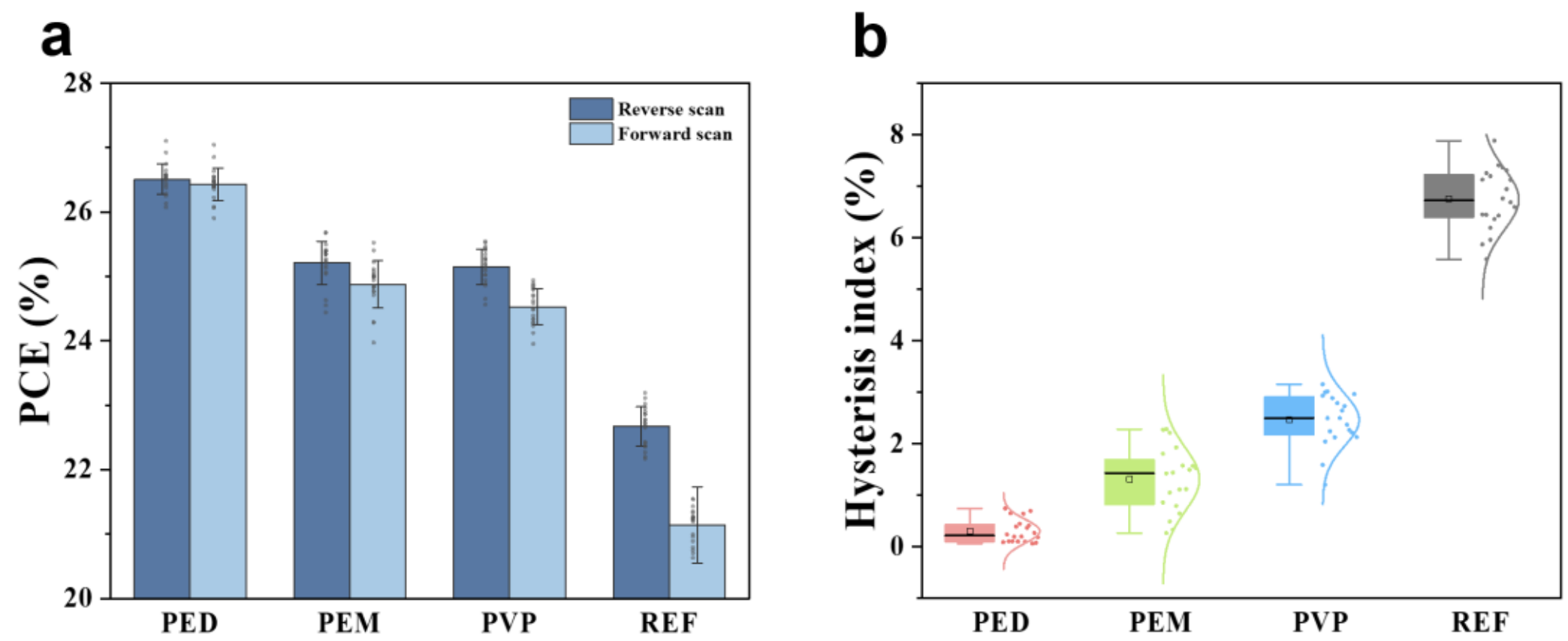

Fig. R7. (a) Statistical comparison of reverse- and forward-scan PCEs for PED-, PEM-, PVP-modified and reference devices. Bars represent mean values, error bars represent standard deviation, and overlaid points represent individual devices (n = 20). (b) Distribution of hysteresis index calculated from the same devices using $HI = (PCE_{\text{reverse}} - PCE_{\text{forward}})/PCE_{\text{reverse}} \times 100\%$. Box-plots show the median and interquartile range, and overlaid points represent individual devices.

Table R2. Statistical summary of forward/reverse J–V measurements and hysteresis indices.

Condtion	Reverse PCE (%)	Forward PCE (%)	Hysteresis Index
PED	26.51±0.24	26.43±0.25	0.302±0.228
PEM	25.21±0.34	24.88±0.37	1.309±0.622
PVP	25.15±0.27	24.53±0.28	2.465±0.505
REF	22.67±0.31	21.14±0.59	6.747±0.594

9.Why does the EQE show a pronounced enhancement in the 350–400 nm region, indicating improved generation and extraction of photogenerated carriers near the bottom interface? Please provide a mechanistic explanation and relevant literature citations.

Our response:

We greatly appreciate the reviewer for pointing out this. The pronounced enhancement of the EQE in the 350–400 nm region provides direct optical evidence for the improved bottom SnO_2/perovskite interface. According to the Beer-Lambert law, the light intensity decays exponentially with penetration depth (**Fig.R21b**):

$$A = log_{10}\frac{I_0}{I_t} = \alpha \cdot l \cdot c,$$

Where A is the absorbance, I_0 is the intensity of incident light, I_t is the intensity of transmitted light, α is the absorption coefficient, l is the thickness of the absorbing medium, c is the concentration of the absorbing substance.

Because perovskite materials possess an exceptionally high absorption coefficient (α) in the short-wavelength region (**Fig.R21a**), photons in the 350–400 nm range exhibit a very shallow penetration depth and are almost entirely absorbed within the first few tens of nanometers adjacent to the buried interface[12].

Consequently, the photogenerated carriers in this specific wavelength band are extremely sensitive to the buried interfacial quality. In the pristine device, energy extraction barriers and deep-level traps at the bare SnO_2 surface cause severe non-radiative recombination of these shallowly generated carriers, leading to a restricted short-wavelength EQE[13]. The introduction of the polymer interlayers effectively passivates these interfacial defects and improves the charge extraction at the buried interface. This effectively suppresses interfacial non-radiative recombination and facilitates the efficient collection of the charge carriers generated near the buried interface, directly resulting in the observed EQE enhancement.

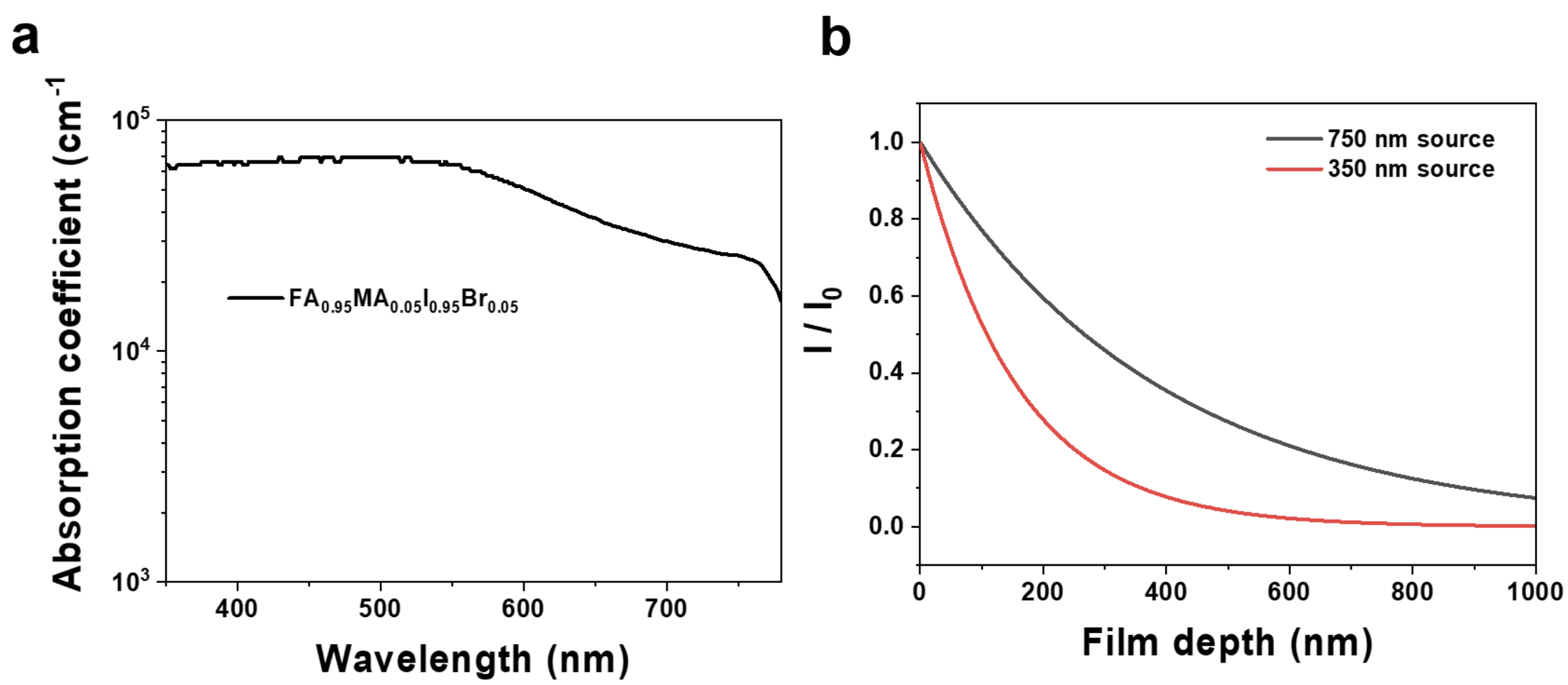

Fig. R21. Absorption spectra of perovskite film. (a) Absorption coefficient of $FA_{0.95}MA_{0.05}I_{0.95}Br_{0.05}$. (b) Depth dependent intensity attenuation of incident light (I_0) with different light source wavelength.

Revised parts in the manuscript:

In page 9 of the revised manuscript:

While the EQE spectra displayed negligible differences above 500 nm, the PED-modified device exhibited a distinctly higher EQE in the 350-400 nm region, indicating improved photocarrier generation and extraction near the buried interface. As short-wavelength photons possess an exceptionally large absorption coefficient and a shallow penetration depth in perovskites (**Supplementary Fig. 8**)[39], they are predominantly absorbed adjacent to the bottom heterojunction. Therefore, this localized EQE enhancement directly confirms that the polymer interlayer successfully suppresses interfacial non-radiative recombination and facilitates efficient charge collection[40].

10.The entire study focuses on a polymer interlayer deposited on SnO2. Why did the authors characterize the energy levels of polymer-doped SnO2 using UPS, rather than measuring the energy levels of the polymer interlayer alone? This approach is inappropriate and reinforces concerns raised in Comment 1 regarding whether these polymers function as n-type semiconductors. Please address this issue critically and revise accordingly.

Our response:

We thank the reviewer for this important comment. We agree that the role of the polymer interlayers and the purpose of the UPS measurements should be clarified more explicitly. First, we would like to clarify that the polymers in this work are not designed or claimed to function as independent n-type semiconductors or standalone electron-transport layers. Instead, they are used as polymeric interfacial modifiers on SnO_2 to regulate buried-interface chemistry, improve perovskite crystallization, and suppress ion migration. Therefore, the most device-relevant electronic structure is not the intrinsic energy level of an isolated polymer film, but the effective surface energetics of the SnO_2/polymer stack that directly contacts the perovskite layer in the actual device.

For this reason, the UPS measurements in the original manuscript were performed on SnO_2/polymer stacks rather than on isolated polymer films. These measurements reflect the device-relevant buried-interface energetics after polymer modification of SnO_2. To further address the reviewer's concern, we have now additionally measured UPS spectra of polymer-only films and added this data to **Fig. R22** and **Table. R5**. The UPS-derived apparent HOMO levels of polymer-only PED, PEM, and PVP films are approximately −7.37, −7.13, and −6.22 eV versus vacuum, respectively. These values are different from those obtained from the corresponding SnO_2/polymer stacks, confirming that the effective interfacial energetics are strongly affected by the underlying SnO_2 substrate and polymer–SnO_2 interactions. This comparison supports our use of SnO_2/polymer UPS data as the device-relevant measurement, while polymer-only UPS serves as a reference for the intrinsic polymer films.

We also emphasize that UPS alone is not used here to assign polymers as n-type semiconductors. To evaluate whether the polymer interlayers impede vertical charge transport, we performed sandwich-type conductivity measurements of SnO_2/polymer stacks. This part is previously discussed in the first question. This result indicates that the polymer-modified SnO_2 stacks maintain sufficient vertical charge-transport capability under solar-cell operating conditions, although the polymers themselves are not treated as conventional n-type transport layers.

Accordingly, we have revised the manuscript to clarify that the polymers function primarily as interfacial modifiers rather than standalone charge-transport layers. We have also added polymer-only UPS spectra and the corresponding extracted apparent HOMO levels to the Supplementary Information in **Supplementary Fig. 36** and **Supplementary Table 9**. In addition, we have

replaced the term “polymer-doped SnO_2” with “polymer-modified SnO_2” or “SnO_2/polymer stack”. We also revised the UPS figure captions from “Fermi edge” to “valence/HOMO onset region” for greater accuracy.

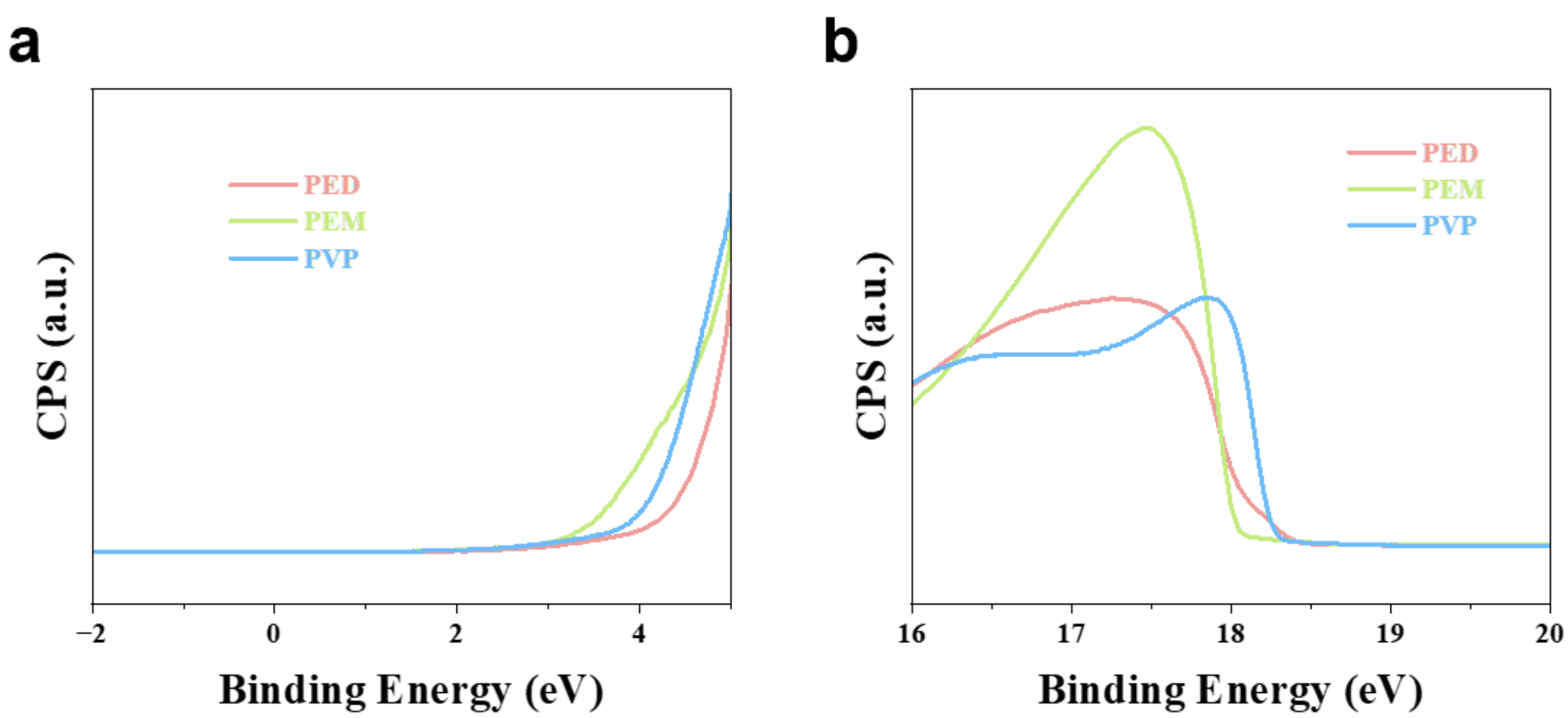

Fig. R22. UPS spectra of polymer only films: (a) HOMO onset region near the Fermi level and (b) secondary electron cutoff region.

Table R5. Calculated HOMO for polymer from UPS results.

Condtion	PED	PEM	PVP
Cut-off Energy (eV)	18.13	18.02	18.26
Work function Φ (eV)	3.08	3.19	2.95
HOMO Onset (eV)	4.29	3.94	3.27
Calculated HOMO level vs vacuum (eV)	-7.37	-7.13	-6.22

Revised parts in the manuscript:

In page 9 of the revised manuscript:

To gain further insight into the origin of the improved device efficiency, the interfacial energy level alignment was examined using ultraviolet photoelectron spectroscopy (UPS) and Kelvin probe force microscopy (KPFM). Since the polymer layers function as interfacial modifiers rather than standalone charge-transport layers, UPS measurements were performed on SnO_2/polymer stacks to probe the device-relevant surface energetics at the buried interface. Polymer-only UPS spectra were also measured as references and are provided in **Supplementary Fig. 36**.

11.The line widths throughout the manuscript are inconsistent. Please unify them for improved presentation and readability.

Our response:

We greatly appreciate the reviewer for pointing out this. We have unified the line widths in the figure throughout the manuscript and SI.

Revised parts in the manuscript:

We have unified the line width in **Figure 1b**, **Figure 2b**, **Figure 2c, Figure 3c, Figure 3e, Figure 3g, Figure 4c, Supplementary Fug.11 and Supplementary Fig.24.**

12.The sources of the starting materials used for polymer synthesis, including the grade, specification, and supplier of AIBN, are not reported in the Experimental Section.

Our response:

We greatly appreciate the reviewer for pointing out this. We have updated the missing information for the starting materials in the experimental section.

Revised parts in the manuscript:

In page 11 of the revised manuscript:

Dimethylsulfoxide (DMSO, 99.7%), isopropanol (IPA, 99.7%), dimethylformamide (DMF, 99.8%), acetone (99.9%), chlorobenzene (99.8%), acetonitrile (99.8%), 4-*tert*-butyl pyridine (98%), lithium bis(trifluoromethanesulfonyl)imide (99.95%), 2,2′-Azobis(2-methylpropionitrile) (AIBN, 98%) and PVP10 (average Mw~10000) were all purchased from Sigma-Aldrich. Lead iodide beads (PbI_2, 99.999%) and SnO_2 colloidal solution (15% in water) were purchased from Alfa Aesar. Formamidine iodide (FAI), methylammonium bromine (MABr) and

methylammonium iodide (MAI) were purchased from GreatCell Solar. Spiro-MeOTAD (99.8%) and methylammonium chloride (MACl) were purchased from Xi'an Polymer Light Technology. 1-ethenylpyrrolidine-2,5-dione and 1-ethenyl-3-methylpyrrolidin-2-one were purchased from Enamine.

Reference

1. Hao, M. & Zhou, Y. Grain-boundary grooves in perovskite solar cells. *Joule* **8**, 913–921 (2024).
2. Xiao, T., *et al.* Elimination of grain surface concavities for improved perovskite thin-film interfaces. *Nature Energy* **9**, 999–1010 (2024).
3. Hao, M., *et al.* Flattening Grain-Boundary Grooves for Perovskite Solar Cells with High Optomechanical Reliability. *Advanced Materials* **35**, 2211155 https://doi.org/https://doi.org/10.1002/adma.202211155 (2023).
4. Wei, J., *et al.* SnO2-in-Polymer Matrix for High-Efficiency Perovskite Solar Cells with Improved Reproducibility and Stability. *Advanced Materials* **30**, 1805153 https://doi.org/https://doi.org/10.1002/adma.201805153 (2018).
5. Bi, D., *et al.* Polymer-templated nucleation and crystal growth of perovskite films for solar cells with efficiency greater than 21%. *Nature Energy* **1**, 1–5 (2016).
6. Zhao, Y., *et al.* A Polymerization-Assisted Grain Growth Strategy for Efficient and Stable Perovskite Solar Cells. *Advanced Materials* **32**, 1907769 https://doi.org/https://doi.org/10.1002/adma.201907769 (2020).
7. Lee, J.-W., Kim, H.-S. & Park, N.-G. Lewis acid–base adduct approach for high efficiency perovskite solar cells. *Accounts of chemical research* **49**, 311–319 (2016).
8. Xing, Y., *et al.* Polymer Lewis base for improving the charge transfer in tin–lead mixed perovskite solar cells. *Nanomaterials* **14**, 437 (2024).
9. Li, X., *et al.* Defect passivation effect of chemical groups on perovskite solar cells. *ACS applied materials & interfaces* **14**, 34161–34170 (2021).
10. Voit, B. Principles of polymerization. By George Odian.). Wiley Online Library (2002).
11. Otsu, T., Matsumoto, A., Kubota, T. & Mori, S. Reactivity in radical polymerization of N-substituted maleimides and thermal stability of the resulting polymers. *Polymer Bulletin* **23**, 43–50 (1990).

12. Lin, Q., Armin, A., Nagiri, R. C. R., Burn, P. L. & Meredith, P. Electro-optics of perovskite solar cells. *Nature Photonics* **9**, 106–112 (2015).
13. Min, H., *et al.* Perovskite solar cells with atomically coherent interlayers on SnO2 electrodes. *Nature* **598**, 444–450 (2021).

Closing statement

We sincerely thank the editor and reviewers again for their constructive comments and suggestions. We have carefully addressed all comments and substantially revised the manuscript, Supplementary Information, figures, and Methods accordingly. We believe that the revised manuscript is significantly strengthened in terms of mechanistic evidence, polymer-interlayer characterization, device-performance analysis, stability evaluation, and experimental clarity. We hope that the revised manuscript now meets the expectations of the editor and reviewers and will be suitable for further consideration in *Nature Communications*.

A point-by-point response to reviewers' comments:

Reviewer 1:

All of my concerns have been well addressed by the authors. I recommend this manuscript for publication.

Our response:

Thank you very much for your positive assessment of our revised manuscript. We sincerely appreciate your constructive comments and helpful suggestions throughout the review process, which have helped us improve the quality and clarity of the manuscript.

Reviewer 2:

The authors have adequately addressed all reviewer comments, and the manuscript is now suitable for acceptance.

Our response:

Thank you very much for your positive assessment of our revised manuscript. We greatly appreciate your constructive comments and suggestions, which have helped us further improve the manuscript.

Reviewer 3:

In the revised version of the manuscript entitled "Mitigating Tin Ion Migration and Reinforcing the Buried Interface via Synergistic Polymer-Modified Tin Oxide," the authors have satisfactorily addressed my comments, as well as those raised by the other reviewers. The revisions are thorough, and the changes made to the manuscript are appropriate and well justified.

Our response:

Thank you very much for your careful evaluation of our revised manuscript and for your positive assessment. We sincerely appreciate your thoughtful and constructive comments throughout the review process, which have helped us substantially improve the clarity and quality of the manuscript.

Reviewer 4:

I have thoroughly checked the authors' point-by-point responses to all reviewer comments. Every concern I raised, alongside those from the other referees, has been comprehensively and satisfactorily resolved. No further amendments are needed, and the manuscript is suitable for publication in Nature Communications in its current version.

Our response:

Thank you very much for your thorough evaluation of our responses and revised manuscript. We sincerely appreciate your constructive comments and valuable suggestions throughout the review process, which have helped us improve the manuscript. We are grateful for your positive assessment and recommendation for publication.
